# PRMT5-mediated arginine methylation of FXR1 is essential for RNA binding in cancer cells

**DOI:** 10.1093/nar/gkae319

**Published:** 2024-05-06

**Authors:** Anitha Vijayakumar, Mrinmoyee Majumder, Shasha Yin, Charles Brobbey, Joseph Karam, Breege Howley, Philip H Howe, Stefano Berto, Lalima K Madan, Wenjian Gan, Viswanathan Palanisamy

**Affiliations:** Department of Biochemistry and Molecular Biology, Medical University of South Carolina, Charleston, SC 29425, USA; Division of Molecular Medicine, Department of Internal Medicine, University of New Mexico, Albuquerque, NM 87131, USA; Department of Biochemistry and Molecular Biology, Medical University of South Carolina, Charleston, SC 29425, USA; Department of Biochemistry and Molecular Biology, Medical University of South Carolina, Charleston, SC 29425, USA; Department of Biochemistry and Molecular Biology, Medical University of South Carolina, Charleston, SC 29425, USA; Department of Biochemistry and Molecular Biology, Medical University of South Carolina, Charleston, SC 29425, USA; Department of Biochemistry and Molecular Biology, Medical University of South Carolina, Charleston, SC 29425, USA; Department of Biochemistry and Molecular Biology, Medical University of South Carolina, Charleston, SC 29425, USA; Department of Neuroscience, Medical University of South Carolina, Charleston, SC 29425, USA; Department of Cell and Molecular Pharmacology and Experimental Therapeutics, Medical University of South Carolina, Charleston, SC 29425, USA; Department of Biochemistry and Molecular Biology, Medical University of South Carolina, Charleston, SC 29425, USA; Department of Biochemistry and Molecular Biology, Medical University of South Carolina, Charleston, SC 29425, USA; Division of Molecular Medicine, Department of Internal Medicine, University of New Mexico, Albuquerque, NM 87131, USA

## Abstract

Emerging evidence indicates that arginine methylation promotes the stability of arginine-glycine-rich (RGG) motif-containing RNA-binding proteins (RBPs) and regulates gene expression. Here, we report that post-translational modification of FXR1 enhances the binding with mRNAs and is involved in cancer cell growth and proliferation. Independent point mutations in arginine residues of FXR1’s nuclear export signal (R386 and R388) and RGG (R453, R455 and R459) domains prevent it from binding to RNAs that form G-quadruplex (G4) RNA structures. Disruption of G4-RNA structures by lithium chloride failed to bind with FXR1, indicating its preference for G4-RNA structure containing mRNAs. Furthermore, loss-of-function of PRMT5 inhibited FXR1 methylation both *in vivo* and *in vitro*, affecting FXR1 protein stability, inhibiting RNA-binding activity and cancer cell growth and proliferation. Finally, the enhanced crosslinking and immunoprecipitation (eCLIP) analyses reveal that FXR1 binds with the G4-enriched mRNA targets such as AHNAK, MAP1B, AHNAK2, HUWE1, DYNC1H1 and UBR4 and controls its mRNA expression in cancer cells. Our findings suggest that PRMT5-mediated FXR1 methylation is required for RNA/G4-RNA binding, which promotes gene expression in cancer cells. Thus, FXR1’s structural characteristics and affinity for RNAs preferentially G4 regions provide new insights into the molecular mechanism of FXR1 in oral cancer cells.

## Graphical Abstract

**Graphical Abstract:**
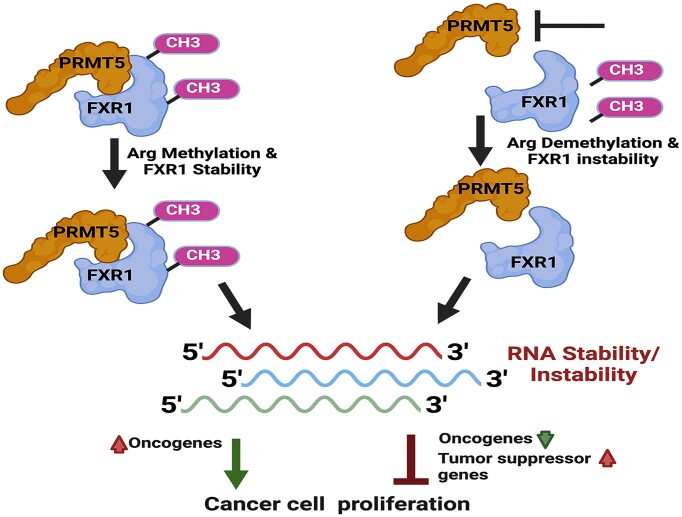

## Introduction

Dysregulated gene expression is a hallmark of cancer, and post-transcriptional gene regulation (PTR) contributes significantly to activating oncogenes and reducing tumor suppressor expression (1,2). The changes in PTR have gained considerable attention for their regulatory roles in biologically significant *cis-* and trans-factors, such as 5′- and 3′-untranslated regions (UTRs) of mRNAs and RNA-binding proteins (RBPs), respectively (3). RBPs regulate critical cellular processes, including transcription, mRNA turnover, and translation (4). However, aberrant expression of RBPs contributes to neoplasia, including head and neck oral squamous cell carcinomas (5,6). Although significant progress has been achieved in understanding RBP-mediated gene regulation (7,8), and cancer-promoting activity, the molecular basis of dysregulated expression of RBPs has yet to be studied. RBP, Fragile X mental retardation protein-1 (FXR1), is a chromosome 3q amplification gene overexpressed in multiple cancers and exerts oncogenic signaling to promote tumorigenesis (9–16). Our published findings indicate that FXR1 helps cancer cells bypass cellular senescence by stabilizing the non-coding telomerase RNA component (TERC) and destabilizing CDKN1A (p21) to promote cell growth (16). Furthermore, our findings also demonstrated that FXR1 targets p21 mRNA destabilization by recruiting miR-301a-3p in both oral and lung cancer cells (17). Although FXR1, its downstream targets, and p53/p21 pathway-mediated cellular senescence are well studied in oral and lung cancer cells, it remains unclear how elevated FXR1 protein enhances malignant transformation in cancer cells. As most RBPs undergo post-translational modifications (PTM) such as phosphorylation, acetylation, methylation, and sumoylation to regulate gene expression in cancer cells (18), here, we set out to study the impact of PTM on FXR1 and its regulatory effects on its RNA targets. Based on the observation and unproven hypothesis that FXR1 is targeted by protein methyltransferases (19), we focused on identifying and characterizing enzymes that methylate FXR1 at the post-translational level and report the functional interactions between FXR1 and methyltransferases.

For the past 30 years, several attempts have been made to understand the biological functions of Fragile-X mental retardation (FXR) proteins in Fragile-X syndrome (20). Still, a significant knowledge gap exists in appreciating the role of the FXR family of proteins in cancer cell structure, function, protein modifications, and RNA metabolism (21). The FXR family members FMRP and FXR1 contain the arginine/glycine-rich (RGG) protein domain, but FXR2 lacks the RGG domain. However, all three FXR families of proteins have K-homology domains, which are ubiquitous throughout eukaryotes (22). FXR1 contains highly conserved arginine residues in its C-terminal nuclear export signal (NES) and the RGG domain. About 0.5–1% of the total arginine residues in the human proteome are methylated and have a slow turnover rate, which will likely confer long-lasting functional properties to the target proteins (23,24). Adding a methyl group(s) to the arginine residues helps the proteins to interact with other proteins and nucleic acids (25). The protein arginine methyltransferases termed PRMTs (PRMT1, 3, 4 [CARM1], 5, 6 and 8), and other probable methyltransferases (PRMT2, 7, 9) are responsible for protein methylation (26). Although the RGG domain functions are relatively known, its biological significance is bypassed in the FXR family of proteins that regulate all levels of RNA metabolism (27–29). It was envisioned that the FXR1 RGG domain could be a target of arginine methyltransferases for methylation (30). However, the specific arginine methyltransferase responsible for the methylation of FXR1 has never been identified. Methylation of FMRP and FXR1 occurs mainly within their RGG box, which may influence their RNA-binding and protein-protein interactions (19). Hence, in this research, we investigated the effect of arginine methylation on FXR1’s RNA binding capacity including its specificity towards guanine rich regions in cancer cells.

FXR1 is known to be involved in mRNA transport, translational control, and mRNA binding via adenylate-uridylate-rich (AU-rich) elements (ARE) (31,32), and G-quartet (G4) RNA structures (33,34). Previous studies have shown that FXR1 undergoes distinct PTM (35,36). However, the enzyme responsible for FXR1’s methylation and how methylated FXR1 impacts RNA binding and alters their expression in cancer cells are unclear. For the first time, here we report, FXR1 is arginine methylated and the functional consequence of methylation relating to RNA binding activity in cancer cells. This study shows that PRMT5 interacts with FXR1 and methylates arginine at positions 386, 388, 453, 455 and 459. Interestingly, both R388 and R455 of FXR1 are necessary to bind to RNAs, with a predilection for G4-RNAs. As a result, we argue that FXR1 methylation increases its G4-RNA-binding capacity, which promotes cancer cell growth and proliferation. Furthermore, the FXR1 mRNA targets identified by nhanced crosslinking and immunoprecipitation (eCLIP)-seq had a greater binding affinity for the G4-rich sequences of top genes such AHNAK, AHNAK2, UBR4, MAP1B, DYNC1H1 and HUWE1. Studies have found that these targets have many functions in various malignancies (37–41). In addition, TCGA database analysis of HNSCC revealed amplification of these RNA targets, implying carcinogenic involvement. However, further study is required to unravel the molecular mechanism by which FXR1 regulates each of its mRNA targets to promote cancer growth. Interestingly, both genetic and small molecule PRMT5 inhibition failed to methylate recombinant as well as the endogenous FXR1, resulting in protein instability and downregulation of FXR1 target mRNA levels in HNSCC cells. Our findings explain one of the molecular mechanisms of FXR1’s reported tumorigenic role in HNSCC and lay the groundwork for future research into how targeting the interface between FXR1 and PRMT5 can affect gene expression and aid in the development of novel therapies.

## Materials and methods

### Cell lines, reagents, plasmids and antibodies

HNSCC cell lines UMSCC11A, -74A and -74B were obtained from the University of Michigan, and SCC4 (#CRL-1624), SCC25 (#CRL-1628) and Cal27 (#CRL-2095) were obtained from ATCC. Lung cancer cell line A549 was also obtained from ATCC. Cell lines UMSCC74B and Cal27, and A549 were routinely grown in Dulbecco's modified Eagle medium (DMEM) containing 10% fetal bovine serum (FBS) with 100 U/ml penicillin-streptomycin (P/S). UMSCC11A and -74A were grown in DMEM containing 10% FBS, 100 U/ml P/S, and 1X non-essential amino acids. SCC4 and SCC25 cell lines were grown in DMEM: F12 (1:1) containing 400 ng/ml hydrocortisone, 10% FBS, and 100 U/ml P/S. A549 was grown in F-12K medium containing 10% FBS and 100 U/ml P/S. shRNA constructs for FXR1 (TRCN0000158932) (16,17) were obtained from Sigma Mission. PRMT5 shRNA was obtained from Santa Cruz biotechnologies (SC41073-SH). Flag-PRMT5 and Flag-MEP50 were generated by cloning the corresponding cDNA into the pRK5-Flag vector (37). HA-PRMT5 was constructed by cloning the corresponding cDNA into the pRK5-HA vector (37). Myc-FXR1 was constructed by cloning the corresponding FXR1 (>NM_005087.4) into the pCDNA3.0 with C-terminal Myc-tag (35). GST-FXR1 was created by cloning (S382-P476) of FXR1 (>NM_005087.4) in the C-terminus of GST gene in pGEX-6P-1 plasmid between EcoR1 and NotI with an intervening stop codon. Single guide RNAs (sgRNA) for PRMT5 were designed at https://www.synthego.com and were cloned into lentiCRISPR v2 vector (Addgene, #52961) (42,43).

### Antibodies

From Cell Signaling Technology, FXR1 (#12295, used predominantly for western blot), Myc-tag (9B11) (#2276), E-Cadherin (24E10) (#3195), N-Cadherin (D4R1H) (#13116), Symmetric Di-Methyl Arginine Motif [sdme-RG] MultiMab™ Rabbit mAb mix (#13222), Asymmetric Di-Methyl Arginine Motif [adme-R] MultiMab™ Rabbit mAb mix (#13522), CD3 (#78588S); From EMD Millipore, FXR1 (#05-1529, used for IP and RNA-IP); From Abcam, FXR1 (#ab50841 for IHC and multiplex); BD Pharmingen, p21, (#556431); From Proteintech, GAPDH (10494-1-AP), Histone H3 (17168-1-AP), GST-tag (10000-0-AP), PRMT5 (18436-1-AP), PRMT1 (11279-1-AP), HA-tag (51064-2-AP), and β-Actin (60008-1-Ig). Horseradish peroxidase-conjugated anti-mouse (NA931) and anti-rabbit (NA934) immunoglobulin Gs were procured from GE Healthcare Biosciences (Uppsala, Sweden). Normal mouse (sc-2025) and rabbit (sc-2027) IgGs were obtained from Santa Cruz Biotechnology. Protein A/G plus (sc-2003) beads were purchased from Santa Cruz Biotechnology. GSK3326593 (PRMT5) and GSK3368712 (PRMT1) inhibitors were obtained from GlaxoSmithKline (GSK) with a material transfer agreement (MTA). The protein thermal shift assay dye was procured from applied biosystems.

### Senescence staining

SA-β-gal activity is determined using X-gal (5-bromo-4-chloro-3-indolyl β-d-galactoside) staining at pH 6.0 according to the manufacturer's instructions (9860, Cell Signaling Technology). A549 cells were transiently infected with Control shRNA and FXR1 shRNA for 72 h and were treated with TGFβ as described in the results section.

### Immunoblot analysis

Cells were lysed using RIPA buffer, supplemented with 1× protease inhibitor cocktail and PMSF, equal amount of proteins were separated using SDS-PAGE. Proteins were transferred to the PVDF membrane, blocked in 5% skimmed milk, and incubated with primary antibodies at 4°C overnight. Membranes were washed three times with 1× Tris-buffered saline-0.1% Tween-20 and incubated with secondary antibody for 1 h at room temperature. Proteins were visualized using substrates Clarity or Clarity Max (Biorad# 1705060 and 1705062), followed by Biorad Image Lab.

### Polysome profiling

A549 cells were treated with TGFβ for 48 h, cells were lysed in TMK100 lysis buffer, and the supernatant was layered onto a 10–50% sucrose gradient and centrifuged at 151 000 × g at 4°C for 3 h. Polysome fractions were collected using a fraction collector with continuous absorbance monitoring at 254 nM. RNAs were extracted with Trizol (Invitrogen) and reverse-transcribed to cDNAs using SuperScript III Reverse Transcriptase. PCR was performed using primers listed below: FXR1: 5′- CCCTAATTACACCTCCGGTTATG-3′ and 5′-TCTCCTGCCAATGACCAATC-3′; β-Actin: 5′- GGACCTGACTGACTACCTCAT-3′ and 5′-CGTAGCACAGCTTCTCCTTAAT-3′. Two percent agarose gel was utilized to resolve the PCR products. Band quantification was performed using Quantity One (Bio-Rad Laboratories, Inc.).

### RNA extraction and qRT-PCR

Total RNA is prepared from HNSCC cell lines using Trizol (Ambion) or RNeasy mini kit (QIAGEN) by following the manufacturer's protocol. qRT-PCR for all m/RNA targets is performed using an Applied Biosystems StepOne Plus system or quantstudio 6.0 pro with the SYBR green master mix RT-PCR kit (SA Biosciences) as described (44). Primer sequences are provided in Supplementary Table S1.

### RNA-seq mapping and quantification

Reads were aligned to the human hg38 reference genome using STAR (v2.7.10a) (45). Genecode annotation for hg38 (version 37) was used as reference alignment annotation and downstream quantification. BAM files were filtered for uniquely mapped reads using custom bash scripts. Quality metrics were calculated using Picard tool (http://broadinstitute.github.io/picard/) and summarized using MultiQC (46). Gene level expression quantification was calculated using FeatureCounts (v2.0.1) (47). Counts were calculated based on protein-coding genes from the annotation file.

### Differential gene expression analysis and functional enrichment

Low-expressed genes were filtered using a per case-control approach with RPKM ≥0.5 as a filter to keep genes. Differential Expression was performed in R using DESeq2 (48). We estimated log_2_ fold changes, P values, and FDR (Benjamini-Hochberg correction). We used FDR <0.05 and abs (log_2_(fold change)) ≥0.5 thresholds to define differentially expressed genes. Custom R codes were used to visualize the data. The functional annotation was performed using the R package clusterProfiler (49) using the GO database. GSEA analysis was performed using the R package fgsea. A Benjamini–Hochberg FDR was applied as a correction for multiple comparisons. Significant categories were filtered for FDR <0.05.

### Transduction (shRNA or sgRNA)

Specific shRNA and control shRNA plasmids or sgRNA and controls were used for the preparation of individual lentiviral particles. Cells were transduced with the lentiviral particles at an MOI (multiplicity of infection) of 25–50 in a medium supplemented with 8 μg/ml polybrene (16) and incubated for 72 h. mRNA levels and the protein expression were analyzed by qRT-PCR and immunoblot respectively.

### Purification of GST-tagged FXR1 proteins from bacteria

50 ml of log phase culture of *E. coli* BL21(DE3) cells containing the pGEX-6P-1-FXR1 plasmid was grown at 37°C in Luria Broth (LB) containing 100 μg/ml carbenicillin. The bacteria were induced to express human truncated FXR1 protein by adding isopropyl β-d-1-thiolgalactopyranoside (IPTG) to a final concentration of 25 uM and incubated for 4h. Cells were harvested by centrifugation at 2500 × g for 10 min at 4°C, resuspended in 10 ml lysis buffer (50 mM HEPES pH 7.9, 150 mM KCl, 1 mM MgCl2, 0.1% Triton-X 100, 0.1 mM phenylmethylsulfonylfluoride (PMSF), and Complete Protease Inhibitor Cocktail (Fisher#P178430) and lysed via sonication on ice (Fisher Scientific Sonic Dismembrator Model 100; three 10 s pulses at level 7). Debris was pelleted via centrifugation at 11 000 × g for 20 minutes at 4°C, and supernatants were added to glutathione sepharose beads for 3 h at 4°C. Beads were rocked with lysates for 1 h at 4°C, then washed 5 times with 2 ml of lysis buffer. GST-FXR1 protein was eluted by adding 0.1 ml of lysis buffer containing 50 mM reduced glutathione and a batch elution method. Eluted samples were dialyzed into a lysis buffer containing 10% glycerol and stored at −80°C.

### *In vitro* methylation assays

PRMT5 *in vitro* methylation assays were performed as previously described (50). Briefly, 5 μg of recombinant GST-FXR1 proteins (WT and mutants) purified from bacterial cells were incubated with immunoprecipitated HA-PRMT5 in the presence of 1 μl of adenosyl-l-methionine, S- [methyl-^3^H] (1 mCi/ml stock solution, Perkin Elmer). The reactions were performed in the methylation buffer (50 mM Tris pH 8.5, 20 mM KCl, 10 mM MgCl_2_, 1 mM β-mercaptoethanol, and 100 mM sucrose) at 30°C for 1 h and stopped by adding 3 × SDS loading buffer and was resolved by SDS-PAGE. The separated samples were then transferred from the gel to a polyvinylidene difluoride membrane, which was then sprayed with EN^3^HANCE (Perkin Elmer) and exposed to X-ray film.

### Immunoprecipitation of FXR1 from UMSCC74B cells

Endogenous FXR1 was purified from control and PRMT5 inhibitor treated 74B cells (2 × 10^6^ cells). For immunoprecipitation all steps were carried out at 4°C. The cells were washed with ice-cold 1X PBS buffer followed by cell lysis using 1× cell lysis buffer containing 20 mM Tris (pH 7.5), 150 mM NaCl, 1 mM EDTA, 1 mM EGTA, 1% Triton X-100, 2.5 mM sodium pyrophosphate, 1 mM β-glycerophosphate, 1 mM Na_3_VO_4_, 1 μg/ml Leupeptin and 1 mM PMSF. Lysate was incubated overnight with IP specific FXR1 or IgG anitibody followed by incubation with Dynabeads for 2 h with gentle rotation. After centrifugation, lysate was removed and beads were washed three times with 1× PBS. FXR1 was purified from the antibody-bead complex using glycine buffer (pH 2.0) and the pH of the elute was adjusted to 7.5 using Tris–HCl (pH 7.5). The protein fractions were analyzed by CBB staining and immunoblot.

### Structural modelling of G4-RNA binding regions of FXR1

FXR1 region S382-P476 was modelled using Phyre2(46) and Alphafold (51) servers. As this region was seen to be completely unfolded, two peptide regions corresponding to regions 382–395 and 450–463 were separately used to thread on the FMRP peptide as seen in the PDB structure 5DE5 (in complex with G4-RNA). Mutagenesis and minimization was accomplished in Chimera (52). All models were minimized by using 1000 cycles of Steepest-descent minimization followed by 50 cycles of conjugate-gradient minimization. All atoms were kept unfixed to allow for free movement. Residue properties were kept in accordance with atom parameters defined by the AMBER ff14SB force field. Finally, hydrogen bonding interaction between G4-RNA and FXR1 peptides was mapped using the generate protocol of PDBsum1(53) hosted by EMBL-EBI. Hydrogen bonds are predicted in accordance with HBPLUS hydrogen bonding potentials developed by McDonald and Thronton (54). Figures were generated using PyMOL.

### Electrophoretic mobility shift assay (EMSA)

Recombinant or endogenous FXR1 protein was assembled onto 30-mer RNA. 0.5 pmol of [y-32P] ATP or 5′ ATTO™ 550 labeled RNA was mock-treated or mixed with recombinant truncated FXR1 (WT or mutants) protein(s) and incubated at room temperature (∼25°C) for 20 min. Reactions were carried out in the final volume of 10 μl of 1X buffer containing 50 mM Tris–HCl pH 7.4, 1 mM MgCl_2_, 0.1 mM EDTA, 150 mM KCl or 150 mM LiCl_2_, 1 mM dithiothreitol (DTT) with 1 U/ul of Murine RNase Inhibitor (NEB), and 100 ug/ml BSA. After incubation, the samples were loaded onto 12% nondenaturing polyacrylamide gel containing 0.5X TBE (Tris–Cl, pH 8.0, Boric acid, EDTA). The electrophoresis was performed at room temperature in 0.5× TBE for 4 h at 125 V. The RNA distribution or shift was visualized by autoradiography after gel drying or imaging at Alexa 546 nm at fluorescence excitation.

### Protein thermal shift assay

FXR1 protein stability in presence of different EMSA buffers were tested using the Protein thermal shift assay dye from applied biosystems. Each reaction was carried out in the final volume of 20 μl and the FXR1 protein melt curve was obtained in quant studio 6.0 pro using the parameters specified by the manufacturers. The raw data was analyzed to determine the normalized fluorescence value for the denatured protein using the protein thermal shift software.

### eCLIP and data analysis

The eCLIP studies were performed by Eclipse Bioinnovations Inc., according to the published single-end eCLIP protocol (55). Approximately 20 million UMSCC 74B cells for two biological replicates were ultraviolet crosslinked at 400 mJ cm^−2^ with 254-nm radiation, cells were scrapped, washed twice with ice cold 1× PBS and stored at –80ºC until it was sent out to Eclipse Bioinnovation Inc. The RBP IP was done using eClip validated FXR1 rabbit monoclonal antibody and the library was prepared according to the published method (55). Libraries generated using the eCLIP-seq method are sequenced using standard SE50 or SE75 conditions on the Illumina HiSeq 2500 platform in standard single-end formats and peaks were compared with the size matched input (smI) and positive control. Peaks were called using the standard eCLIP processing protocol 0.2, which is available at: https://github.com/YeoLab/eclip.

### Immunofluorescence

Optimized multiplex immunofluorescence was performed using the OPAL™ multiplexing method. OPAL™ is based on Tyramide Signal Amplification (TSA) using the Roche Ventana Discovery Ultra Automated Research Stainer (Roche Diagnostics, Indianapolis, IN). Tissues were stained with antibodies against [DAPI, CD3 (1:100), FXR1 (1:100), and PRMT5 (1:100)], and the fluorescence signals were generated using the following fluorophores: [OPAL dyes, Marker + Dye Pairing, Dilution used] (Akoya Biosciences, Marlborough, MA). Slides were imaged at 20X magnification using the Vectra® Polaris™ Automated Quantitative Pathology Imaging System (Akoya Biosciences, Marlborough, MA) and analyzed using inForm® Tissue Analysis Software (v[2.6.0], Akoya Biosciences, Marlborough, MA).

### Cell viability and colony formation

Cell viability rate upon UMSCC74B or A549 cells treated with GSK3326593 (GSK593) and GSK3368712 (GSK712) alone or in combination for 72 h is determined using MTT cell proliferation assays (Invitrogen). Briefly, 5 × 103 cells were seeded into each well of a 96-well plate (well area = 0.32 cm^2^). On the next day, cells were treated with 2 μM of each drug alone or in combination every 24 h, and the medium was replaced with an experimental medium (100 μl). MTT solution was prepared fresh (5 mg/ml in H2O), filtered through a 0.22-μm filter, and kept for 5 min at 37°C. The MTT solution (10 μl) was added to each well post-treatment, and plates were incubated in the dark for 4 h at 37°C. The reaction was stopped using MTT stop solution (10% SDS in 1N HCl) and further incubated overnight at 37°C. The following day the absorbance was measured at A570 nm using a plate reader (Bio-Rad).

### Statistical analysis

Data are expressed as the mean ± the standard deviation. Two-sample t-tests with equal variances are used to assess differences between means. Results with *P*-values <0.05 are considered significant.

## Results

### TGFβ-induced FXR1 undergoes post-translational modification in cancer cells

**Figure 1.**
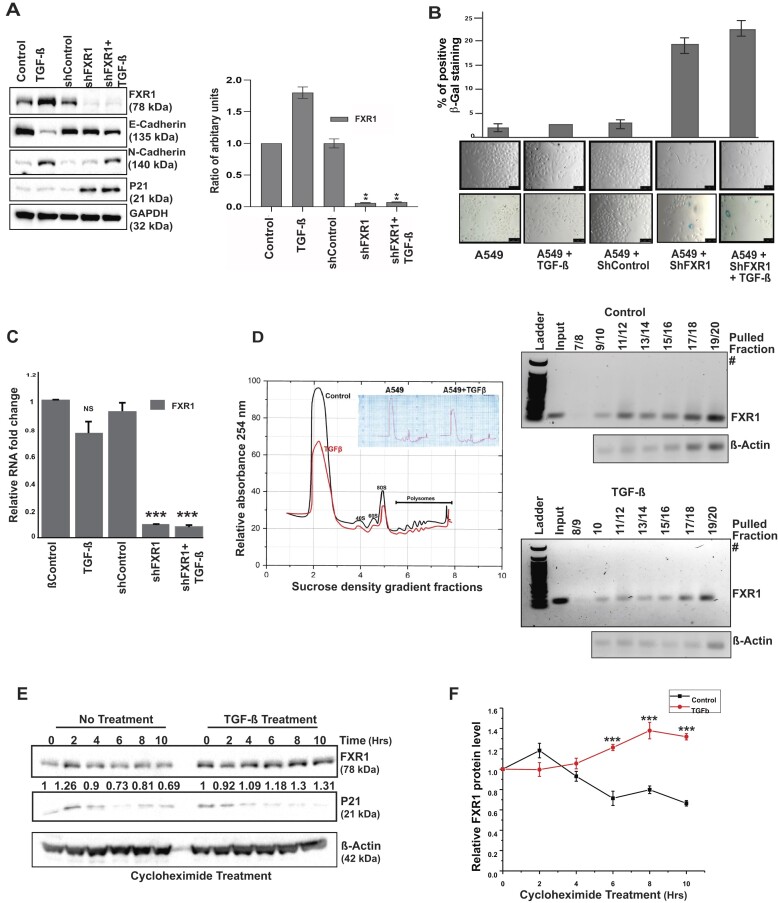
TGFβ-induced FXR1 undergoes post-translational modification in cancer cells. (**A**) Western Blot analyses show protein regulation by TGF-β treatment (48 h) on A549 cells. GAPDH serves as a loading control. The bar graph on the right side depicts the quantitative value of FXR1 in panel-A western blot. *N* = 3. (**B**) Analyses of cell morphology (upper panel) and β-Gal staining (lower panel) of the A549 cells treated with TGF-β and shRNA. The upper panel depicts the quantitative pixel values of β-gal positive cells, an indicator of cellular senescence. (**C**) qRT-PCR of the samples mentioned above (A and B) show that TGF-β only affects the FXR1 protein and does not affect its RNA level. *N* = 3. ***P < 0.0005. (**D**) Polysome profiling of A549 cells with TGF-β treatment compared to control. DNA gel shows the RT-PCR products from serial polysome fractions from control and treated TGF-β samples and analyzed for FXR1 expression in each pulled polysome fraction. (**E**) A549 cells were pretreated with TGF-β or control diluent for 48 h, followed by 5 μM cycloheximide treatment for 0 to 10 h to block protein synthesis. After the treatment, the cells were harvested at the indicated time points and immunoblotted for FXR1, P21 and β-actin (loading control). (**F**) Quantitative analyses of FXR1 protein levels in control and TGFβ treated A549 cells followed by cycloheximide treatment. The results plotted here represent the mean ± SEM of three independent experiments. All the data were defined as mean ± SD and were analyzed by Student's *t*-test (*n* = 3). ***P < 0.0005.

Our previous findings demonstrated that overexpressed FXR1 in metastatic oral cancer cells (UMSCC-74A, -74B) and lung adenocarcinoma A549 cells contribute to tumor growth and proliferation (16,17). Silencing FXR1 promotes cellular senescence by activating CDKN1A (p21) and downregulating TERC RNA in both oral and lung A549 cells (16). Hence, to determine the molecular basis of high FXR1 protein levels in cancer cells, we used A549 lung cancer cells, which show metastatic phenotype under the treatment of cytokine transforming growth factor-β (TGFβ) (56). The TGFβ-signaling mediated epithelial-to-mesenchymal transition (EMT) is a hallmark of tissue fibrosis, tumor invasiveness, and metastasis (57). Therefore, to study whether EMT plays a role in high FXR1 protein levels, we used A549 cells and tested their expression under TGFβ. As shown in Figure 1A, TGFβ induced the expression of FXR1 protein with reduced E-cadherin and increased N-cadherin levels (EMT markers). The right panel shows the FXR1 protein quantification. Although FXR1 knockdown (KD) alone showed no changes in the E-cadherin and N-cadherin levels, the addition of TGFβ in FXR1 KD cells facilitated a moderate decrease in E-cadherin and an increase in N-cadherin levels compared to only TGFβ treated cells. This observation is further confirmed by cell morphology changes, in which TGFβ-induced cells exhibit a mesenchymal phenotype and silencing of FXR1 induces senescence (Figure 1B, top panel shows quantitation of senescence). However, the changes in E- and N-cadherin levels from TGFβ treated FXR1 KD cells (Figure 1A) may signify the changes occurring only in quiescent cells. Next, we tested whether TGFβ-induced FXR1 protein levels are mediated through transcriptional activation of the FXR1 transcript levels. Surprisingly, no difference in mRNA levels of FXR1 was observed in TGFβ-induced cells (Figure 1C). Hence, we tested whether TGFβ influences the mRNA translation of FXR1 using a polysome gradient assay. The TGFβ-induced A549 cells showed no change in mRNA translation of FXR1 compared to untreated cells (Figure 1D). These data indicate that high expression of FXR1 in the presence of TGFβ might be associated with post-translational modification (PTM) that may contribute to its protein stability. Therefore, we tested FXR1 protein stability by treating the cells with the protein synthesis inhibitor cycloheximide. As shown in Figure 1E and F, the TGFβ treated cells showed increased FXR1 protein stability compared to untreated cells, implying that FXR1 may undergo PTM in TGFβ-treated cells. The findings indicate that the molecular basis for overexpressed FXR1 levels in cancer cells is possibly due to PTM, which could influence its oncogenic function.

### PRMT5-mediated arginine methylation promotes the PTM of FXR1

**Figure 2.**
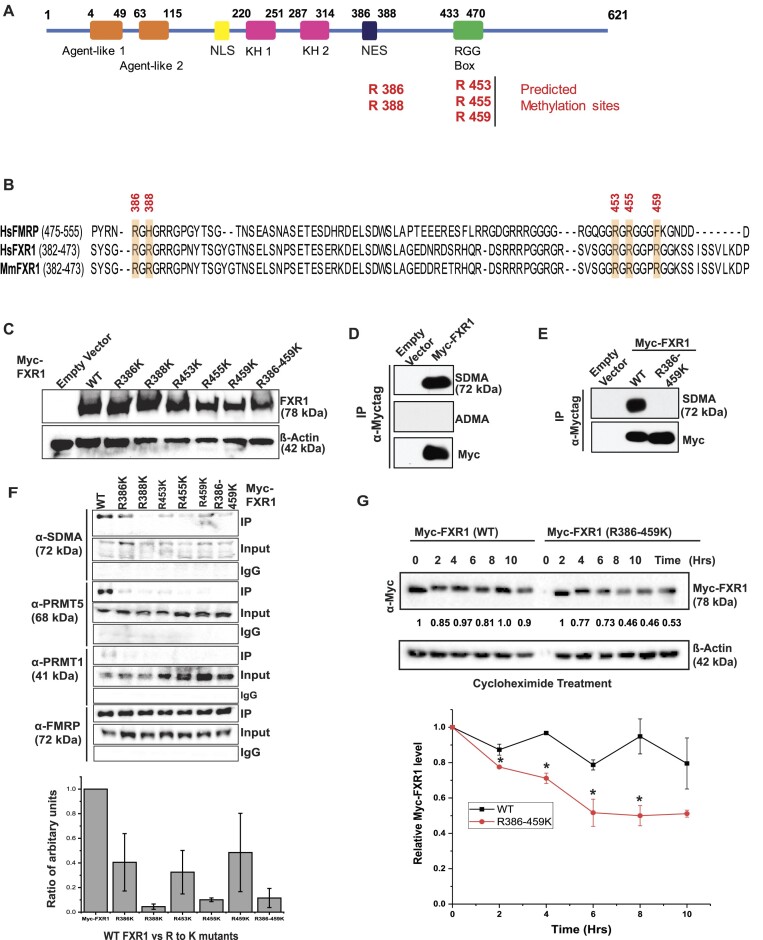
PRMT5-mediated arginine methylation promotes PTM of FXR1. (**A**) The protein structure of FXR1 protein has regions marked for its different domains. The C-terminal arginine-glycine-glycine (RGG) RNA-binding domain has the methylated arginine (R) residues marked in the illustration. (**B**) Multiple sequence alignment of the C-terminus of human and mouse FXR1 and FMRP proteins is shown. Secondary structural elements are marked above the sequences, with α-helices depicted as cylinders and β-strands as arrows. The R residues potentially methylated inside the cell have been chosen for the mutation to lysine (K) and are highlighted (yellow). The FXR1 residue numbers are given above the sequence. The numbers in parentheses indicate the length of the sequences shown. (**C**) Immunoblot analyses of WT and mutant Myc-FXR1 protein expressions in HEK293T cells are shown. β-Actin serves as a loading control. (**D**) HEK293 cells expressing empty vector and Myc-tag FXR1 (WT) were used for IP with Myc-tag antibody and probed for SDMA, ADMA and Myc-tag antibodies. The empty vector serves as a control for Myc-FXR1. (**E**) HEK293 cells expressing empty vector, Myc-tag FXR1 (WT), and mutant (R386-459K) were used for IP with Myc-tag antibody and probed for SDMA and Myc-tag antibodies. (**F**) HEK293 cells expressing empty vector, Myc-tag FXR1 (WT), and mutants R386K, R388K, R453K, R455K and R459K were used for IP with Myc-tag antibody and probed for SDMA, PRMT5, PRMT1 and FMRP (positive control). The bottom panel depicts the quantitative value of WT and RGG mutants FXR1 protein interaction with PRMT5. *N* = 3. (**G**) A549 cells stably expressing Myc-tag FXR1 (WT) and mutant (R386-459K) were treated with 5 μM cycloheximide treatment for 0 to 10 h to block protein synthesis. After the treatment, the cells were harvested at the indicated time points and immunoblotted for FXR1 and β-actin (loading control). The bottom graph shows the relative FXR1 protein levels with time after cycloheximide treatment. All the data were defined as mean ± SD and were analyzed by Student's *t*-test (*n* = 3). *P < 0.05.

The TGFβ-induced PTM of FXR1 may be carried out by phosphorylation, acetylation, or arginine methylation to promote protein stability of the RGG domain-containing proteins (58). Hence, we determined whether specific arginine methylation carried out by protein methyltransferases targets FXR1 and promotes its stability in cancer cells. We used PhosphoSitePlus (phosphosite.org) amino acid predictions and selected the methylation sites on specific arginine residues of FXR1. Based on the C-terminal NES and RGG domain amino acid sequences, we chose arginine amino acids 386, 388, 453, 455 and 459 (Figure 2A) and studied their methylation status and interactions with different methyltransferases. The preferred amino acids are highly conserved between humans and mice and moderately conserved in a known FXR family member, FMRP (Figure 2B), suggesting that these conserved amino acids may play a role in the biological function of FXR1 in cancer cells by contributing to its stability. To determine the arginine methyltransferase that methylates FXR1, we generated Myc-tagged FXR1 with Arg (R) to Lys (K) mutation of the above residues. We expressed and confirmed the wild-type and R mutant constructs (individually and together) in the human embryonic kidney (HEK) 293T cells (Figure 2C). PRMT1 is the primary type I enzyme responsible for approximately 80% of total arginine methylation (asymmetric dimethylarginine [ADMA]), whereas PRMT5 is the dominant type II enzyme that generates symmetric dimethylarginine (SDMA) (59). The expression of both PRMT5 and PRMT1 has been tested in multiple head and neck squamous cell carcinoma (HNSCC) and A549 cells where OHKC (immortalized normal oral keratinocytes) and DOK (dysplastic oral keratinocytes) cells serve as normal and dysplastic cell lines (Supplementary Figure S1A). PRMT5 is predominantly expressed across all the cell lines compared to PRMT1. We also found that the levels of FXR1 and PRMT5 increased with TGFβ treatment (Supplementary Figure S1B). Hence, we tested the methylation status of wild-type (WT) and mutant FXR1. The cellular lysates from HEK 293T cells transfected with an empty vector and a plasmid expressing Myc-FXR1 (WT) were immunoprecipitated using a c-Myc antibody, separated by sodium dodecyl-sulfate polyacrylamide gel electrophoresis (SDS-PAGE), and immunoblotted for both ADMA and SDMA (Figure 2D). An antibody specific to ADMA failed to detect methylated FXR1, however an antibody against SDMA detect the WT-FXR1 indicated that FXR1 is symmetrically dimethylated at Arg residues. HEK293T cells expressing WT and R386/459K FXR1 were subjected to immunoprecipitation (IP) with Myc-antibody and probed for anti-SDMA antibody. As shown in Figure 2E, the SDMA antibody only reacted to the WT and failed to detect any methylation on FXR1 (R386-459K), confirming the symmetrical dimethylation of these arginine residues in FXR1. Hence, to ensure PRMT5 interacts with methyl Arg residues of FXR1, both WT and R386-459K independently expressing cell lysates were subjected to IP and probed for SDMA, PRMT5, PRMT1 and a positive control FMRP (which interacts with FXR1 through N-terminal Tudor domains) (60). As shown in the figure, WT FXR1 interacts with SDMA antibody and PRMT5 through the c-terminal NES/RGG box; however, it failed to establish a strong interaction with PRMT1. This finding indicates that PRMT5 targets Arg residues of FXR1 and methylates them. More importantly, Arg residues of R388, R455 and a complete mutation of Arg residues failed to interact with PRMT5, suggesting that these two Arg residues are likely targeted by PRMT5 (Figure 2F and the bottom graph). The direct protein-protein interaction between FXR1 and PRMT5 was further confirmed using overexpressed HA-tagged PRMT5 IP lysates probed for both SDMA and FXR1 in HEK293T cells (Supplementary Figure S1C). Finally, we carried out the cycloheximide assay to ensure Arg residues are essential for FXR1 protein stability. Both Myc-tagged stably expressed WT and R386-459K proteins in A549 cells were treated with cycloheximide for designated times (up to 10 h) and tested for FXR1 levels by probing with Myc-Ab. As indicated in Figure 2G, after 10 hours, the WT FXR1 level is comparable to its initial time. In contrast, the mutant protein level after 10 hours was reduced to ∼50% compared to the initial time. This observation indicates that arginine residues at positions R386, 388, 453, 455 and R459 may be essential for FXR1 protein stability, individually or collectively. Thus, these observations demonstrated that PRMT5 interacts with FXR1 and promotes its stability in cancer cells.

### Silencing PRMT5 reduces FXR1 and cell growth in HNSCC cells

**Figure 3.**
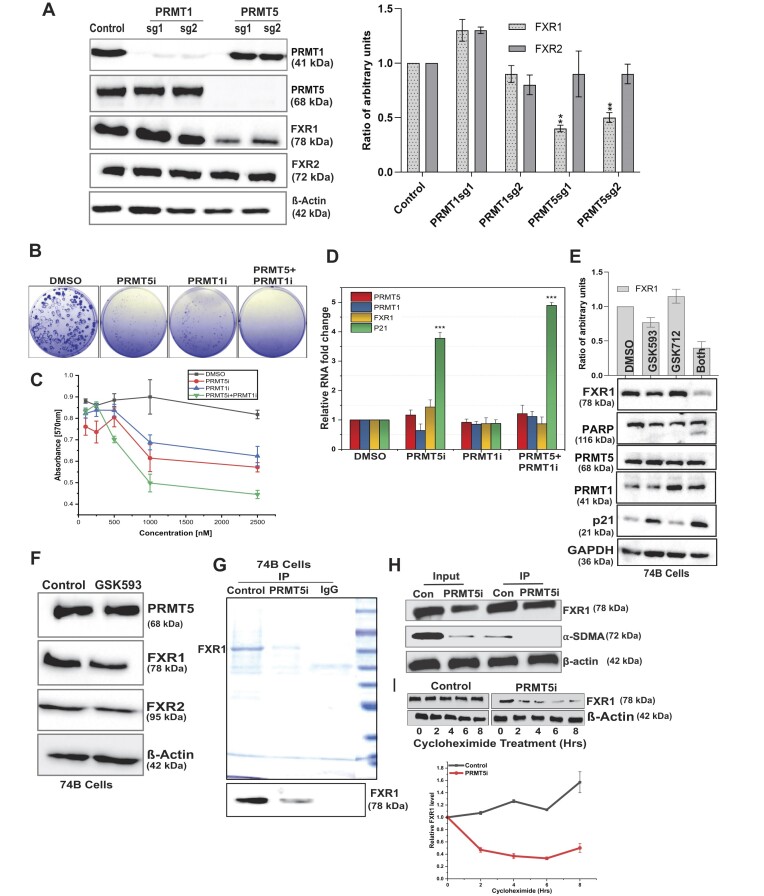
Genetic and small-molecule inhibition of PRMT5 reduces FXR1 and cell growth in HNSCC cells. (**A**) The immunoblot shows two independent guide RNA-mediated knock out (KO) of PRMT1 and PRMT5 in UMSCC74B oral cancer cells. β-Actin serves as a loading control. Quantitative protein levels of FXR1 and FXR2 from three independent experiments are shown as a bar graph (right panel). (**B**) The panel depicts the colony-forming efficiency from clonogenicity assays of UMSCC74B cells treated with indicated drugs and DMSO for 72 h. (**C**) MTT analysis of cell viability in UMSCC74B cells treated with indicated drugs and DMSO for 72 h. Data presented as the mean ± SD of three independent experiments. (**D**) UMSCC74B cells were treated with PRMT5i and PRMT1i (1.5 μM) for 72 h. RNA extraction followed by qRT-PCR was done to determine the relative mRNA levels of FXR1, PRMT5, PRMT1 and p21. All the data were defined as mean ± SD and were analyzed by Student's *t*-test (*n* = 3). ***P < 0.0005. (**E**) Immunoblot analysis of cell extracts obtained from UMSCC74B cells treated with PRMT5i and PRMT1i for 72 h. GAPDH serves as a loading control. The upper bar graph shows the quantitative analyses of FXR1 expression upon treatment. (**F**) Immunoblot analyses of FXR1, comparing FXR2 and PRMT5 levels in UMSCC74B cells upon PRMT5i treatment for 72 h. β-actin served as a loading control. (**G**) Endogenous FXR1 was purified from UMSCC74B control and PRMT5i (2 μM) treated cells using FXR1 specific antibody and mouse IgG (negative control) antibody. Purified protein fractions were analyzed by 10% SDS-PAGE followed by CBB staining. The bottom panel represents the immunoblot confirmation of FXR1 protein obtained from IP. (**H**) Estimating methylation status of endogenous FXR1 purified from UMSCC74B cells treated with PRMT5i (2 μM). Immunoblot was probed with FXR1 and SDMA antibody, a marker of protein methylation. (**I**) UMSCC74B cells were treated with and without PRMT5i for 72 h, followed by treatment with 5 μM cycloheximide for 0 to 8 h to block protein synthesis. After the treatment, the cells were harvested at the indicated time points and immunoblotted for FXR1 and β-actin (loading control). The bottom graph shows the relative FXR1 protein levels with time after cycloheximide treatment. *N* = 3. All the data were defined as mean ± SD and were analyzed by Student's *t*-test *P < 0.05.

PRMT5 is the primary enzyme responsible for arginine SDMA of target proteins and it prefers the consensus arginine- and glycine-rich regions known as RGG/RG motifs (61). PRMT5 targets numerous RGG domain-containing proteins, and inhibiting PRMT5 decreases target protein levels via demethylation (61,62). However, PRMT1 has been shown to carry out protein methylation without PRMT5, indicating a redundancy in the activation of protein methylation by these two methyltransferases (63). As a result, we investigated whether silencing PRMT5 and PRMT1 affected FXR1, FXR2 and FMRP levels in oral and lung cancer cells. As shown in Figure 3A and the right graph panel, we used two guide RNAs (CRISPR/Cas9) to knockout PRMT1 and PRMT5 in oral cancer cells (lung cancer cells, Supplementary Figure S2A), and only PRMT5 deletion reduced FXR1 levels but not FXR2 (which lacks the RGG domain), as previously described (16). Interestingly, PRMT1-silenced cells did not change the protein levels of FXR1 or FXR2, indicating that FXR1 may be a direct substrate of PRMT5 in oral cancer cells. Furthermore, we could not detect the protein FMRP (data not shown), which is not expressed in oral or lung cancer cells.

Based on the effect that PRMT5 had on FXR1 levels, we wanted to see if inhibiting PRMT5 demethylated FXR1 and regulated its actions in cancer cells. GlaxoSmithKline (GSK) has found that both the PRMT1 inhibitor GSK3368712 (GSK712) and the PRMT5 inhibitor GSK3326593 (GSK593) have anti-tumor effects in a variety of cancer cell lines, with the exception of HNSCC (64). To investigate the efficacy of PRMT5 inhibition, we treated oral and lung cancer cells with single and combined PRMT5 and PRMT1 inhibitors (PRMT5/1i). The combination treatment with PRMT5/1i resulted in considerably reduced colony formation (Figure 3B and S2B) and cell growth (Figures 3C and S2C) in both cell lines. Next, we investigated the capacity of PRMT5/1i to inhibit FXR1 mRNA transcript and protein levels in cancer cells. Following the treatment described above, the mRNA and protein levels were measured in the UMSCC74B cells. In addition, we also measured the p21 levels because, FXR1 silencing was already known to regulate p21 mRNA levels (16). PRMT5i treatment had little effect on FXR1 mRNA, but it elevated p21 levels significantly in oral cancer cells (Figure 3D). This finding suggests that FXR1 remains unchanged at the mRNA level. However, demethylation by PRMT5i could affect FXR1 protein and increase p21 levels. In addition, we checked the protein levels of FXR1 and p21 to ensure the inhibitor's effectiveness. As Figures 3E and F indicated, PRMT5 inhibition affected FXR1 but not FXR2 protein levels. Interestingly, a significant rise in p21 levels was also found in PRMT5-inhibited cells, implying that unmethylated FXR1 may be dormant in both oral and lung cancer (Figure S2D and S2E) cells. Interestingly, inhibiting PRMT1 and PRMT5 increased PARP cleavage, which can be attributed to the cell death as demonstrated by the inability to form colonies (Figure 3B). Next, to confirm our observation that inhibiting PRMT5 methyltransferase activity reduces Arg methylation and destabilizes FXR1, we employed endogenous FXR1 isolated from control and PRMT5i-treated UMSCC74B cells (Figure 3G). The purified fraction was tested with the SDMA antibody, which is a marker for PRMT5 activity. Our findings demonstrated that the inhibitory action of PRMT5 failed to methylate FXR1 *in vivo* (Figure 3H). To investigate the effect of demethylation on FXR1 protein stability, we treated the cells with cycloheximide in PRMT5 inhibited UMSCC74B cells. The time-dependent experiment demonstrated that FXR1 protein stability is significantly reduced in PRMT5 inhibited cells, in which FXR1 is demethylated (Figure 3I and bottom panel). In addition, we wished to test whether silencing the activity of PRMT5 alters the localization of FXR1 in cancer cells. As shown in Supplementary Figure S2F, there is no change in FXR1 distribution in the cells under PRMT5 silencing condition, demonstrating that demethylation of FXR1 did not alter its cellular localization. These findings clearly showed that FXR1 is dependent on PRMT5 for its methylation and stability, and that reducing FXR1 methylation promotes p21 levels and preventing the cancer cell growth.

### Arginine amino acids are essential for FXR1 to bind to G4-RNA sequences

**Figure 4.**
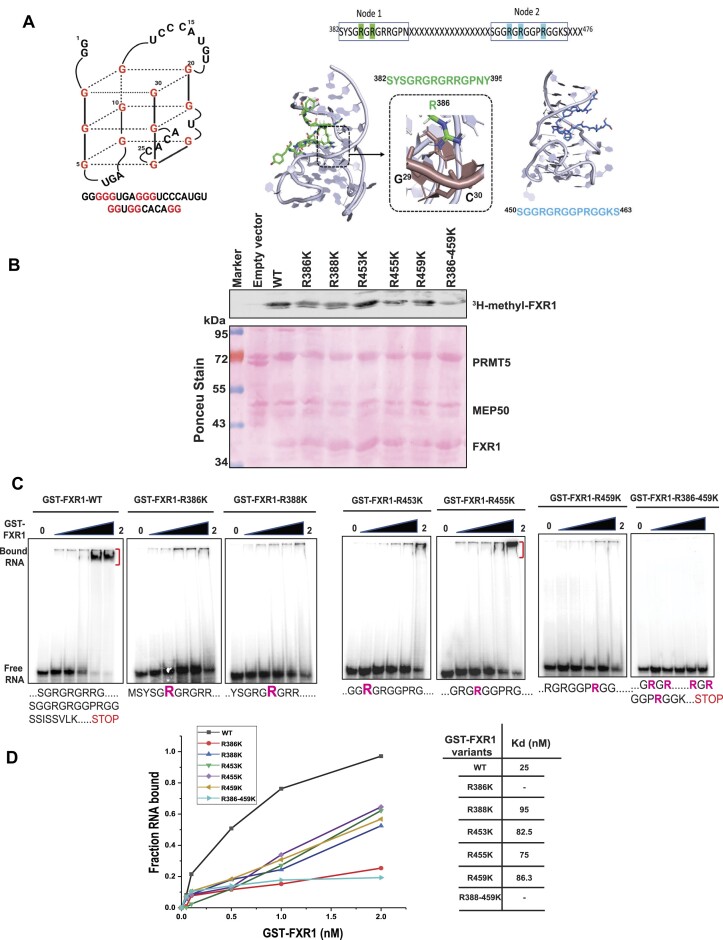
Arginine residue in the NES and RGG domain of FXR1 are essential to bind with G4-RNA sequences. (**A**) The sequence and plausible structure of a 30-mer RNA is used for EMSA assays. The energy-minimized model of FXR1 region 382–395 is threaded on the structure of FMR1 with G4-RNA. When threaded in either direction, R386 makes strong hydrogen bonds with G4-RNA nucleotides and backbone phosphates. Node assembly to investigate G4-RNA binding of FXR1 region 382–476. Peptides from regions 382–395 and 450–463 were used to model them with G4-RNA. Interacting arginine residues that show sensitivity to methylation are highlighted. (**B**) *In vitro* methylation assay was performed with recombinant GST-FXR1 protein purified from bacterial cells and Myc beads bound with PRMT5/MEP50. The methylation assay was carried out in the presence of ^3^H-SAM. The binding was performed at 4°C for 4 h, incubated with or without PIP3 (20 μM), and subjected to immunoblot analyses. PRMT5.MEP50 proteins were purified from HEK293 cells. The Ponceau stain below serves as a loading control for the immunoblot above. (**C**) EMSA with 5′-labeled 30-mer RNA, recombinant FXR1 (S382-P476) WT, and respective arginine mutant proteins. 0.5 pmol of [y-32P] ATP-labeled RNA was mock-treated or mixed with increasing concentrations of recombinant WT and mutant FXR1 proteins and incubated at 25°C for 20 min. Free RNA and RNP complexes are shown in the figure. (**D**) The binding curves and affinity constants are shown for each recombinant protein-RNA complex.

**Figure 5.**
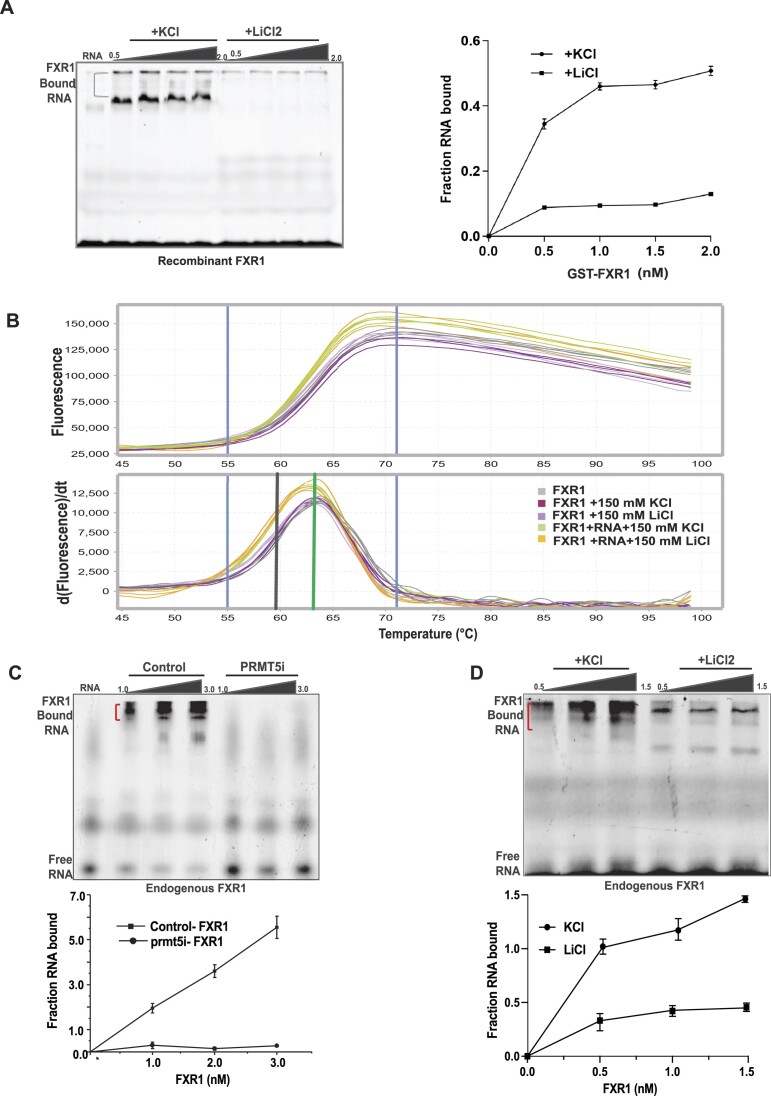
PRMT5-dependent FXR1 methylation is required for G4-RNA binding in HNSCC. (**A**) EMSA was performed as mentioned above with 5′ ATTO 550 labeled 30-mer RNA using recombinant WT FXR1 protein in EMSA buffer containing 150 mM KCl/LiCl_2._ The RNA-protein interaction was analyzed using 10% native PAGE gel and visualized using typhoon FLA 7000 at 546 nm. The right panel shows the binding curves of EMSA. B. Protein thermal shift assay was used to screen for the effect of KCL/LiCl_2_ on FXR1 using Sypro Orange. Data from protein thermal shift software show the Boltzmann (upper) and derivative (lower) melt profiles of FXR1 with or without different buffers (KCL/LiCl_2_)_,_ and with RNA (sample used for EMSA). Data were collected as mentioned in the methods. The median derivative T_m_ and Boltzmann derivative *T*_m_ are represented in black and green vertical lines, respectively. (**C**) EMSA was performed as indicated above with endogenous FXR1 from UMSCC74B cells with and without PRMT5 inhibitor treatment. The bottom panel represents the binding curves of EMSA. (**D**) EMSA was performed as indicated in above in a buffer containing 150 mM KCl/ LiCl_2._ The bottom panel represents the binding curves of EMSA.

Previous studies have shown that arginine residues in the FMRP RGG box are required for G-quadruplex (G4) RNA binding (19,65,66). As a result, we investigated whether arginine residues in FXR1 have a similar role in binding to the p21 mRNA fragment that contains a canonical G4-RNA sequence. The protein structure of FXR1 is less well-established than that of the FMRP C-terminal domain secondary structure (65). It is also unclear how FXR1 identifies G4-RNAs and which amino acids are required for binding to G4-RNAs. To assess the relevance of these arginine residues in FXR1-G4-RNA binding, we created a 30 nucleotide RNA (sequence excised from human P21 3′UTR, seg1 (17)) with a G4-RNA motif (Figure 4A). We have previously demonstrated that FXR1 binds to G4-enriched fragment of the p21 3′UTR (16,17). To analyze the structural workings of various arginine binding capacities, we modeled FXR1 S382-P476 using the Phyre248 and Alphafold49 servers (53). Because this region lacked any secondary structural elements, we identified two nodes for threading into G4-RNA-bound structures using the FMR1 peptide as a template (PDB ID:5DE5) (67). Here, Node1 is defined between amino acids 382- 395 (contains R386 and R388), and Node2 entails amino acids 450–463 (includes R453, R455 and R459) (Figure 4A). Our modeling analysis showed that Node1 formed a complex with G4-RNA using R386 when threaded in either direction (from N to C terminus, Figure 4A or C to N terminus, Supplementary Figure S3A). Specifically, R386 formed stable hydrogen bonds with G29, C30, C5, and G7 when threaded from the N to C terminus and the C to N terminus, respectively (Figure 4A). In comparison, Node2 could only be threaded from the N to C terminus, where C to N terminus threading was disallowed due to stearic clashes of the peptide with the G4-RNA. Hence, modeling studies indicate that these two nodes are the predominant interactors of G4-RNA. Finally, we sought to determine whether FXR1 arginine amino acids are critical for binding with G4-RNA. To begin, we cloned a protein sequence comprising FXR1’s NES and RGG (S382-P476) domains in the pGEX-6P1 vector, then altered the arginine residues (R to K) and purified it using the GST-affinity purification technique (Supplementary Figure S3B). The *in vitro* methylation analysis showed that PRMT5 successfully methylated WT FXR1 but failed to adequately methylate the arginine mutants R386, R388, R45’, R459 and R386-459K (Figure 4B), demonstrating that PRMT5 methylates arginine at these specific positions on FXR1. Further, the recombinant WT and arginine mutant FXR1 proteins were subjected to an electrophoretic mobility shift assay (EMSA) with a radiolabeled 30-mer/ fluorescently labeled G4-RNA substrate. The resulting EMSA studies showed that WT FXR1 binds with G4-RNA at a dissociation constant (Kd) of 25 nM; however, most R to K (arginine to lysine) mutants of FXR1 bind poorly with G4-RNA with high *K*_d_ and the R386K, and R386/459K fails to interact with the G4-RNA (Figure 4C and D). To validate the specificity of FXR1 to the G4 region, we used LiCl2 instead of KCl as a metal ion in the EMSA buffer and examined the binding. It has been shown that potassium stabilizes the G4-RNA over lithium (68). As shown in Figure 5A (right panel, binding curve), potassium ions enhance G4-RNA binding to FXR1. Our results showed that lithium failed to retain the G4-RNA structure and could not bind to FXR1, indicating that FXR1 prefers G4-RNA configurations. However, it is critical to demonstrate that LiCl2 does not affect FXR1 protein stability and merely destabilizes the G4 structure. As a result, we performed the protein thermal shift assay (PTSA) as described in the experimental methods. We observed that LiCl2 had no negative influence on protein stability and maintained the same melting temperature as the sample buffer containing KCl. In addition, the same trend was observed when we used the samples with RNA between different buffers (Figure 5B). To validate our *in vitro* observation, we conducted the EMSA with endogenous FXR1 protein purified from UMSCC74B control and PRMT5i cells using FXR1 specific antibody. As shown in Figure 5C and the bottom panel binding curve, the endogenous FXR1 exhibited a similar binding affinity to G4-RNA in control FXR1 whereas the binding was not significant in the FXR1 purified from PRMT5i cells. Furthermore, the endogenous FXR1 lost the RNA binding when we used LiCl2 as previously seen with the recombinant protein (Figure 5D and bottom graph). Thus, our findings provide compelling evidence that FXR1 preferentially binds to G4-RNA via its selective arginine residues.

### The RNA-binding landscape of FXR1 demonstrates its possible role in RNA regulation

**Figure 6.**
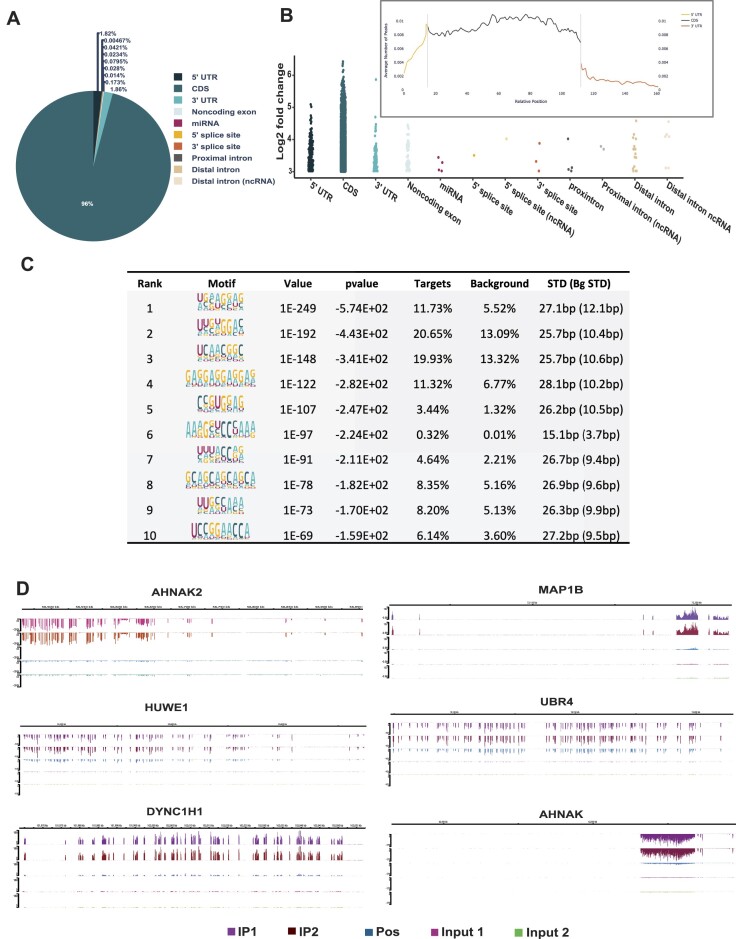
RNA binding landscape of FXR1 by eCLIP and RNA seq. (**A**) The pie chart depicts the distribution of the FXR1 eCLIP peaks in the human genome analyzed from two biological replicates. UTR-untranslated region; CDS coding sequence. The data was considered with the cut-off values of peak log_2_ fold enrichment ≥3 and *P*-value ≤0.001. (**B**) The binned FXR1 eCLIP peak coverage across all expressed genes in UMSCC74B cells. The inset represents the metagene plots of the normalized average number of peaks mapped to specific genomic regions. The 5′UTR, CDS and 3′UTR of each gene are split into 13, 100 and 70 bins, respectively. (**C**) Top ten most significantly enriched *de novo* sequence motifs in the FXR1-binding peaks using HOMER12. The percentage of peaks containing the discovered motifs and the p-values of the motifs calculated by a binomial test against the random genomic background was shown. (**D**) Integrated genome viewer (IGV) browser tracks the FXR1’s eCLIP peaks of top targets (based on pvalue and log_2_ fold change) spanning the genomic loci of AHNAK2, MAP1B, HUWE1, UBR4, DYNC1HI and AHNAK. Detailed information about all significantly enriched eCLIP peaks can be found in Supplementary Data-1.

In our recent findings (16,17), we demonstrated that FXR1 binds to the G4-specific region of p21 and degrades the mRNA in an miR301a-3p-dependent manner. In addition to our findings, others have found that FXR1 targets multiple mRNAs, including p21, in mouse C2C12 cells (69). Hence, we decided to determine the global analysis of FXR1-associated transcripts using enhanced crosslinking and immunoprecipitation (eCLIP) (55). As described, the UMSCC74B cells were subjected to UV-cross linking and IP with FXR1 for eCLIP analysis. The eCLIP followed by RNA-seq analysis (GEO: GSE252916, reviewer token- kfwrseckrlepjet), data show that FXR1 binds to diverse locations (5′ and 3′ UTR, coding and intergenic RNA regions) of several target RNAs, accounting for 21000 reproducible peaks in both biological replicates (Supplementary Data 1). Further analysis revealed that 96% of FXR1 binding peaks were matched to coding sequences (Figure 6A). However, FXR1 has also displayed a high RNA binding preference for 5′, coding, and 3′ UTR sequences (Figure 6B and the inset). Hence, both 5′ and 3′ UTR sequences were taken for further analysis due to their role in mRNA turnover and translation functions. We focused on 3′UTR sequences over 5′UTR due to their direct role in RNA turnover functions. Our data indicate that 1.86% of eCLIP peaks was also mapped on the 3′ UTR, that are highly enriched with top targets such as MAP1B, HUWE1, DYNC1H1, AHNAK2, AHNAK and UBR4. The FXR1 binding RNA sequence motifs were identified using HOMER12 de novo motif analysis (http://homer.ucsd.edu/homer/motif/). Based on their *P*-value, the resulting motif analysis indicates that the most enriched peaks displayed high G-rich sequences (Figure 6C and Supplementary Data 2). Based on their G4-rich sequences and binding preference to top targets, we mapped the FXR1 binding to the respective mRNA targets using the hg19 genome browser as indicated by eCLIP analysis. As shown in Figure 6D, FXR1 IP samples showed significant enrichment of target mRNAs compared to input samples, indicating that FXR1 preferentially binds to selective regions of mRNAs. Next, we intended to determine whether the enriched mRNAs contain canonical G4-RNA sequences in their 3′UTR (Supplementary Data 3). We used a G4 mapper (70) to map the potential G4 sequences in the most enriched peaks for the top FXR1 RNA targets. Surprisingly, most of the FXR1’s identified RNA targets contain numerous G4 sequences spanning from the 5′UTR to the 3′UTR (Supplementary Data 4). Altogether, the findings from this eCLIP analysis further confirm our earlier *in vitro* and *in vivo* investigations, indicating FXR1 has a relatively higher affinity for binding towards G4-RNA sequences in the mRNA. Moreover, the gene ontology (GO) enrichment analysis revealed that FXR1 interacting mRNA encoding proteins are associated with cell cycle, phosphatidylinositol signaling, ubiquitin-mediated proteolysis, and nucleocytoplasmic transport (Supplementary Data 5). These findings suggest that the FXR1-RNA network-associated biological processes facilitate cancer cell growth and proliferation.

### Multifaceted gene regulatory roles of FXR1 in HNSCC cells

**Figure 7.**
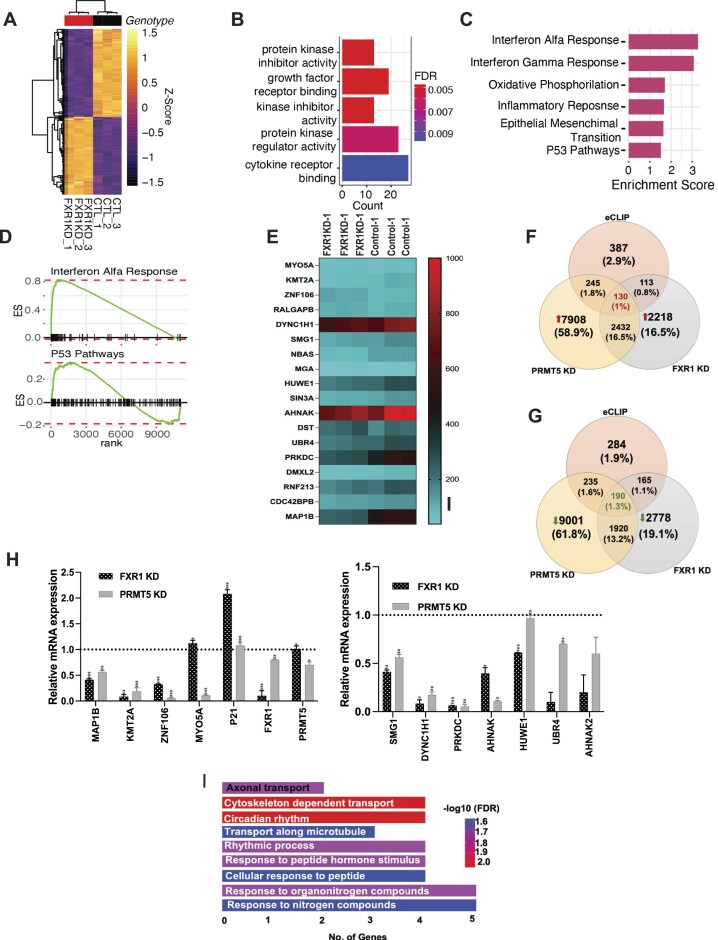
FXR1 and PRMT5-dependent altered gene signatures in HNSCC cells. (**A**) Heat map of significantly differentially expressed genes identified between FXR1 KD and control samples. Rows show *Z* scores of normalized, log2-transformed values from differentially expressed genes (FDR < 0.05). Dendrograms depict Pearson correlation clustering of samples. (**B**) Bar plot representing the functional enrichment of FXR D1 DEGs of the top 6 genes ontology biological process (BP). The X-axis corresponds to the number of genes in the functional ontology. The Y-axis shows the top 5 functional ontologies ranked by significance. Gradient color depicts the FDR value (red = most significant, blue = least significant). (**C**) Bar plot representing the functional enrichment of FXR D1 DEGs of the top 6 hallmark gene set from MSigDB database (FDR < 0.05). The X-axis corresponds to the normalized enrichment score based on GSEA analysis. (**D**) Graphical representation of the rank-ordered gene lists for Interferon Alfa Response (NES = 3.29, FDR = 1.24e-27) and P53 Pathways (NES = 1.50, FDR = 1.27e-02) hallmark gene sets. (**E**) Heat map for the top FXR1 eCLIP RNA targets shows differential expression profile in UMSCC74B control and FXR1 KD cells. (**F**) Venn diagram represents the FXR1 eCLIP targets commonly up-regulated in both FXR1 KD and PRMT5 KD conditions. (**G**) Venn diagram represents the FXR1 eCLIP targets commonly down-regulated in both FXR1 KD and PRMT5 KD conditions. (**H**) Quantitative real-time PCR validation of top eCLIP targets having the highest fold-change and *P*-values compared to the size-matched input. The results plotted here represent the mean ± SEM of three independent experiments. All the data were defined as mean ± SD and were analyzed by Student's *t*-test (*n* = 3). ***P < 0.0005. (**I**) The bar graph represents the GO enrichment analyses of the top eighteen FXR1 eCLIP targets.

To interrogate the oncogenic functions and gene signatures essential for cancer cell growth and proliferation, we performed an RNA-seq by silencing FXR1 and PRMT5 separately using shRNAs and analyzed the high-throughput sequencing data. The silencing effect of *shPRMT5* was confirmed using immunoblot (Supplementary Figure S4A). For this analysis, we used total RNA isolated from the WT, FXR1 KD and PRMT5 KD cells, and subjected them to bulk RNA sequencing analysis (FXR1:GSE212760, reviewer token-ypqfmuiapxetdyh, PRMT5: GSE256352, reviewer token-mdapmcyijzgjdud). Bioinformatics analyses identified several differentially expressed genes based on a threshold of *q* ≤ 0.05 (FDR 5%) for statistical significance and a log-fold expression change with an absolute value of at least 1. Principal Component Analyses (PCA) plot depicts the gene expression variance that is exhibited between KD samples of FXR1 and PRMT5 (Supplementary Figure S4B). The heat map of differentially expressed genes (DEGs) identified in the KD and control samples is depicted in Figure 7A, and S4C showed PRMT5’s DEGs. The next bar chart and the dot plot depicts the functional enrichment of DEGs from diverse biological processes in FXR1 KD (Figure 7B) and PRMT5 KD respectively (Supplementary Figure S4D, upregulated pathways S4E down regulated pathways). The x-axis corresponds to the number of genes in the functional ontology. The functional enrichment of FXR1 DEGs indicated top 6 hallmark gene sets obtained from the MSigDB database (Figure 7C), demonstrating its biological importance relating to interferon pathways. More importantly, Gene Set Enrichment Analysis (GSEA) predictions, and we identified 22 pathways that FXR1 significantly impacts. The GSEA pathway further shows that several cancer pathways are negatively affected, and anti-cancer pathways are positively regulated. Graphical representation of the rank-ordered gene lists for Interferon Alfa Response and P53 Pathways hallmark gene sets (Figure 7D). The heat-map of FXR1 KD RNA seq depicts the expression levels of various top eCLIP targets according to the highest fold change and pvalue (Figure 7E). While analyzing the RNA-seq data of FXR1 knockdown, we observed changes in multiple pathways associated with cancer. However, examined the significance of these findings concerning the eCLIP targets of FXR1. Next, to investigate the expression of regulated mRNAs (DEGs) connected with FXR1 (eClip) under FXR1 or PRMT5 KD circumstances, we identified the mRNAs that are present in all three conditions. Specifically, 130 genes showed increased expression (Figure 7F) and 190 genes showed decreased expression (Figure 7G). The GO enrichment of FXR1 eCLIP target expression that is altered under FXR1 and PRMT5 KD conditions is found to be mostly enhanced in nucleic acid binding, and helicase activities and reduced in enzyme binding and regulatory activity (Supplementary Figure S4F and S4G). To validate the changes in FXR1-related transcripts under both KD conditions, we examined the expression of important gene targets that are tightly bound to FXR1. According to the data presented in Figure 7H, the qRT-PCR validation of selective FXR1 targets showed a predominant decrease in expression in both FXR1 and PRMT5 KD cells. Surprisingly, TCGA database analyses of HNSCC patient tissues have revealed the FXR1 top targets are altered at the mRNA level, indicating the targets may exert an oncogenic role in HNSCC (Supplementary Figure S4H). Moreover, the GO enrichment analyses revealed that the 18 highest-ranking mRNA targets of FXR1 are majorly involved in nitrogen metabolism, microtubule formation, axonal control, and cell proliferation (Figure 7I). This suggests that FXR1 can bind to and stabilize these transcripts, hence possibly promoting the growth and proliferation of cancer cells. The results further indicate that the FXR1-PRMT5 axis could have a significant impact on the development of cancer through the control of the above-mentioned biological process.

### Overexpressed PRMT5 and FXR1 predict poor patient outcomes and show clinical significance

**Figure 8.**
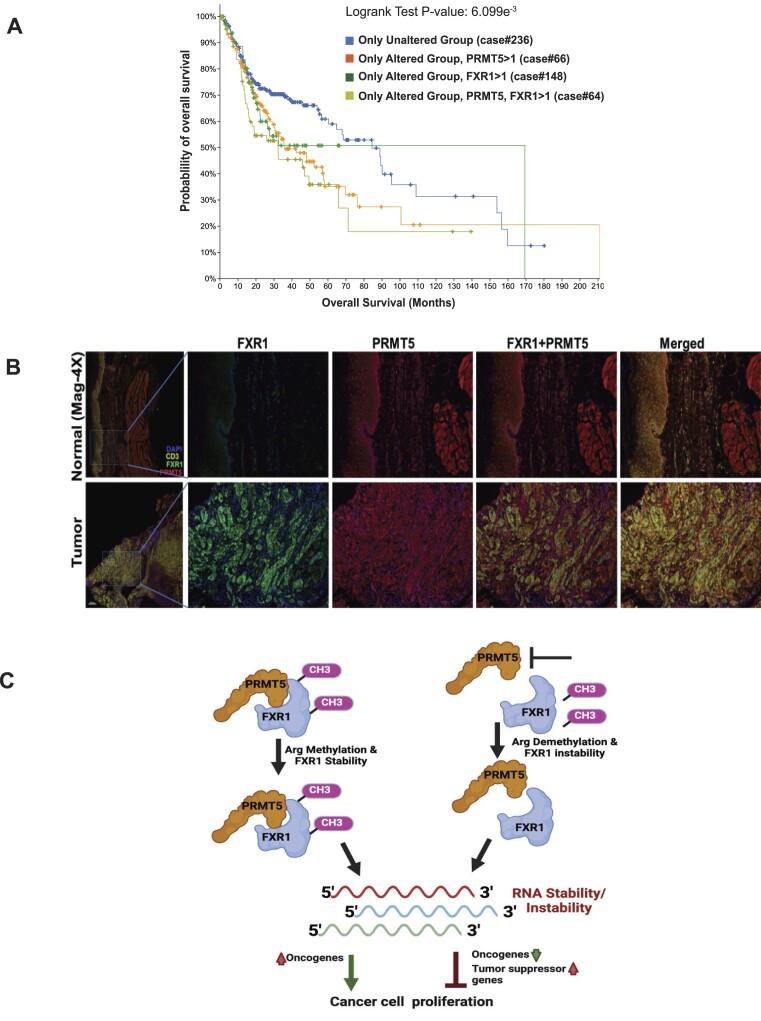
PRMT5-dependent FXR1 preferentially targets oncogenes and alters its expression in HNSCC. (**A**) Kaplan–Meier plots of overall survival of stage HNSCC patients (*n* = 522) stratified by FXR1 and PRMT5 mRNA expression (SD > 1). The log-rank *P* value and the number of cases per group are shown. (**B**) Optimized multiplex immunofluorescence showing the expression of FXR1 and PRMT5 in human HNSCC tumor and normal adjacent tissue samples. DAPI and CD3 staining was done for the nucleus and tumor markers. (**C**) Model represents the methylation dependent regulation of FXR1 and its RNA targets to promote or inhibit the tumor cell proliferation.

Others have reported that PRMT5 is overexpressed in HNSCC (71), and inhibition of PRMT5 by EPZ015666 (GSK3235025) reduces H3K4me3-mediated Twist1 transcription and suppresses the carcinogenesis and metastasis of HNSCC (72). PRMT5 (73) and FXR1 (14,16,21) are overexpressed in multiple cancers, but combinatorial expression changes in cancers have never been reported. In addition, we tested the mRNA level changes of PRMT5 and FXR1 in The Cancer Genome Atlas (TCGA) HNSCC and lung adenocarcinoma data sets. As shown in the survival plot the overexpressed PRMT5 and FXR1 (SD > 1) alone (Supplementary Figure S5A and S5B) or in combination (Figure 8A), lead to poor patient survival in HNSCC and lung cancer patients. FXR1 protein is overexpressed in oral tumors compared to normal tissue and colocalized with PRMT5, demonstrating that both proteins contribute to an oncogenic phenotype (Figure 8B). Hence, targeting PRMT5 to modulate FXR1 functions is significant and may provide a unique anti-tumor response for HNSCC and lung adenocarcinoma patients.

## Discussion

The results of our study have revealed that FXR1 is a target of PRMT5 for arginine methylation. Furthermore, our data indicate that arginine methylation occurs explicitly in the NES and RGG box domains of FXR1 in cancer cells. Chromosome 3q amplification in lung and oral cancer patients leads to an increase in FXR1 mRNA levels and exert oncogenic properties (14,16). This study has identified and added a new feature that FXR1 protein undergoes post-translational modification by PRMT5-mediated arginine methylation, which enhances the stability of FXR1 protein (Figure 1). Our findings also show that PRMT5 directly adds a dimethyl group to FXR1 arginine residues in cancer cells. Based on the FXR1-PRMT5 protein-protein interaction and methylation status, the residues R388K and R455K demonstrated a lack of interaction with PRMT5 compared to WT, demonstrating that these residues might have a strong preference to get methylated by PRMT5. The improved stability of FXR1 protein may be attributed to the arginine residues R388 and R455, which exhibited robust interactions with PRMT5 (Figure 2A). Moreover, we have also demonstrated that FXR1 demethylation through inhibition of PRMT5 affected the protein stability and reduced the cancer cell proliferation (Figure 3I).

Post-translational modifications, including arginine methylation, regulate protein functions and this modification requires approximately 12 ATPs to add a single methyl group to a protein (78). Methyl groups added to the amino groups of amino acid side chains often increase steric hindrance and reduce hydrogen bonds by replacing the amino hydrogens (79). For example, hnRNP A1 is methylated by PRMT5 on two residues, R218 and R225, which facilitates the interaction of hnRNP A1 with IRES RNA to promote IRES-dependent translation (82). Arginine methylation of different proteins, including FXR1 family protein, FMRP, affects protein–RNA interactions, protein localization, and protein-protein interactions (25). Studies have shown that the RGG box of FMRP, is known for recognizing G-quadruplex RNAs (81) and arginine residues are highly favored when it comes to RNA binding (80). Moreover, published findings showed that the folding of G4-RNAs *in vitro* is similar to *in vivo* conditions (83). For example, the sequences we used from p21 3′-UTR are folded as a G4 (Figure 4B) to bind with FXR1 properly. Additionally, the studies have indicated that G4-RNA must be efficiently folded to interact with protein FMRP (84). Due to the close proximity of FXR1 arginine residues spanning NES and RGG motifs, there is a likelihood that PRMT5 methylates multiple arginine residues at a given time and alter the protein stability and function of FXR1. Further, this methylation also facilitates FXR1 to bind with G4-RNAs and control their expression through a potentially novel mechanism, which requires further exploration. Based on our biochemical structure prediction, we have used a 30-base RNA that forms a G4 structure to show the binding affinity of FXR1 arginine residues. Both *in vitro* and *in vivo* assays show that arginine residues present in the NES (R386 and R388) and RGG domain (R453, R455, R459) of FXR1 are essential for binding with G4-RNAs (Figures 4 and 5). Subsequent *in vitro* binding experiments using arginine mutants demonstrated that changes in arginine residues of FXR1 lead to decreased affinity for G4-RNA. Interestingly, the binding study employing the endogenous FXR1 further validated our *in vitro* observations and confirmed the interplay between FXR1 and PRMT5 that is vital for G4-RNA binding by FXR1 (Figure 5). To further prove our claim that FXR1 prefers G4-RNAs, we used LiCl2 to destabilize the G4-RNAs and see the effect through binding studies. It has been shown that structural analysis of G4-RNA with various metal ions favors potassium as a stabilizing agent over lithium (68) (Figure 4). Interestingly, in the presence of potassium FXR1 strongly interact with G4-RNA, but lithium destabilizes the G4-RNA structure and disrupts the binding with FXR1 (Figure 5), suggesting that FXR1 may prefer a noncanonical G4-structure to interact with the RNA. Previous findings from the Darnell laboratory also stated that FMRP binds with G4-RNAs and represses mRNA translation in neuronal cells (74). Thus, methylation of the arginine residues can either help increase or decrease the RNA binding capacity of the methylated protein.

Our published findings show that FXR1 specifically targets the G4-rich regions of p21 mRNA and TERC long non-coding RNA to control their expression in oral cancer cells (16). Deleting the G4-region of p21 mRNA specifically did not interact with FXR1 in cancer cells, indicating that FXR1 prefers G4-sequences in the 3′UTR to regulate the expression of target genes. FXR1 facilitates the degradation of p21 mRNA at the molecular level by enlisting miR-133a-3p and PNPase to induce instability (17). The mechanism by which FXR1 binds to and stabilizes TERC RNA through interaction with the G4-region is not well understood. TERC RNA may not have miRNA binding sites, hence FXR1 interaction could potentially enhance TERC stability rather than destabilize it. Darnell group showed that FMRP interacts with the coding region of many mRNAs associated with autism spectrum disorders (75). Interestingly, FMRP is known to interact with G4-RNA sequences located at the 3′UTR, influencing the localization and translation of target mRNAs (77). It is also vital to show in this study that FXR1 prefers the G4-mRNAs in head and neck cancer cells, mostly localized in the cytoplasm. Nevertheless, our eCLIP data clearly demonstrate that FXR1 interacts with and regulates the target mRNAs both in a positive and negative manner in cancer cells (Figure 6). Utilizing the eCLIP analysis and FXR1 KD gene signature analysis, we have successfully demonstrated that the differential gene expression is mediated by FXR1. According to the eCLIP motif analysis, FXR1 can bind to both G- and U-rich sequences. The FXR1 target mRNA encoding proteins include AHNAK, AHNAK2, MAP1B, HUWE1, and DYNC1H1, as depicted in Figure 6, enriched with G4-sequences. Our data also show that FXR1 targets the coding regions, 5′UTR, and 3′UTR of key genes involved in microtubule filaments, potentially linked to cancer progression (Figure 7). For instance, MAP1B, a microtubule filament protein, the prominent target of FXR1, is also targeted by FMRP and is associated with autistic spectrum disorder and autophagy (76). Therefore, establishing the connection between FXR1 and the microtubule-associated gene network would reveal the crucial role of FXR1 in cancer cells. Further experimental strategies are needed to determine if FXR1 binds to non-G4 RNAs and acts as a repressor or promoter of their mRNA turnover and translation in cancer cells.

Our RNA-seq and eCLIP analysis showed that silencing FXR1 can have both cancer positive and negative effects on gene expression, suggesting that the recognition of G4-region may influence mRNA turnover regulation. The contrasting roles of FXR1 in mRNA stability and destabilization considering the G4-structural features need to be investigated further in cancer cells. Together, our results show that arginine methylation may influence its target mRNAs having preference towards G4 enriched sequences to regulate its gene expression in cancer cells. FXR1 shows high methylation levels and can have more preference to bind G4-RNAs containing regulatory signals for generating proteins that are crucial for encouraging tumor growth. Thus, the current results indicate a straightforward function of FXR1 in cancer cells that may pave the way for targeting the NES/RGG box for therapeutic intervention to elucidate the regulation of tumor suppressors in cancer cells.

Collectively, our data unambiguously demonstrated the molecular interaction between PRMT5 and FXR1 by the impartial techniques. As demonstrated in Figure 8, head and neck tumors have limited survival and poor outcomes due to the overexpression of FXR1 and PRMT5. The rationale behind integrating FXR1 and PRMT5 inhibitors to improve clinical outcomes is presented in our work. More importantly, as our model illustrates (Figure 8C), we showed that PRMT5-activated FXR1 is intricate in controlling the mRNA expression of its targets, playing both tumor-activating and tumor-suppressive roles. Therefore, further research is required to fully comprehend FXR1’s involvement in mRNA synthesis and turnover in cancer cells, leading to cancer growth and proliferation.

## Supplementary Material

gkae319_Supplemental_Files

## Data Availability

The data underlying this article are available in the Gene Expression Omnibus, and can be accessed under accession codes GSE252916, GSE212760 and GSE256352. Further data is available in ModelArchive at https://modelarchive.org/doi/10.5452/ma-epklf.
