# Supplementary material for: PRMT5-mediated arginine methylation of FXR1 is essential for RNA binding in cancer cells
Source: Nucleic Acids Res. 2024 May 6;52(12):7225–44. doi: 10.1093/nar/gkae319 (PMC11229354; doi:10.1093/nar/gkae319)
Supplement: gkae319_Supplemental_Files [file gkae319_supplemental_files.zip › Supp. data 3.docx]

**AHNAK2**

00001 AGTTGGGATC TTCGCTTCTG GGCCAGTTGG GAGAGCGTCT GTAGCTTCCT TGTGTCCGGC CGTCAGCTGC AGCCC**GGGG**A GCCA**GG**TGCA GAAAC**GG**AAG
000101 ATGACCACTC TGTGACTGAA GGGCCTGC**GG** ATGAG**GG**CAT TCGACCAC**GG** CCGCAG**GG**GT CTTCACCTGT CTACGAATAC ACGACTGAGG CTGCCGACTT
000201 T**GG**ACTCCA**G G**AAGACGCCC CC**GG**CA**GG**CA A**GG**TTCTGCT **GG**GAGAC**GG**A GATCCT**GG**TG GAAGCGAGAT TCA**GGGG**ACT CAC**GG**ACATT TTTCA**GG**ATG
000301 AGTCGTCCAG A**GG**CTGTCCA **GG**A**GG**CAACA GA**GG**TGACGC TGAAGACAGA **GG**T**GG**A**GG**CA **GG**AGCCAGTG GCTACAGTGT CACA**GG**T**GG**T **GGGG**ACCAGG
000401 GGATCTTCGT CAAGCAAGTG CTGAAGGACT CCTCAGCCGC CAAGCTTTTT AACTTGAGAG AAGGGGATCA GCTGCTCAGT ACAACCGTGT TCTTTGAAAA
000501 CATAAAATAT GAAGATGCTC TCAAAATCCT TCAATATTCA GAGCCGTACA AGGTTCAGTT CAAAATCAGA CGGCAGCTCC CTGCCCCACA GGATGAAGAG
000601 TGGGCTTCCA GCGATGCCCA GCAC**GG**CCCA CA**GG**GCAA**GG** AGAA**GG**AGGA CACGGATGTT GCTGATGGGT GCAGAGAGAC CCCCACGAAA ACTCT**GG**AA**G**
000701 **G**AGAT**GGGG**A CCAAGAGAGA CTCATCTCCA AACCAAG**GG**T GG**GG**AGA**GG**C A**GG**CAGAGCC AGAGGGAGAG GCTCTCTTGG CCAAAATTTC AATCCATAAA
000801 GAGCAAGC**GG** G**GG**CCG**GG**AC CCCAGA**GG**TC ACACAGCTCG TCAGAGGCCT ACGAACCTAG GGACGCACAT GACGTGTCCC CTACAAGCAC AGACACAGAG
000901 GCCCAGCTCA CGGTGGAGCG CCAAGAGCAG AAGGCA**GG**GC CG**GG**CAGCCA GA**GG**AGGC**GG** AAGTTCCTCA ACCTCAGATT CA**GG**ACA**GG**C TC**GG**GACA**GG**
001001 GCCCTTCATC GACAGGACAG CCAGGCAGG**G GG**TTCCAGAG T**GGG**GT**GGG**C CGTGCT**GGG**G TCCT**GG**AAGA GTT**GGGG**CCC TG**GG**GTGATA GCCTCGAGGA
001101 GACTGGGGCT GCCACA**GG**CA GCA**GG**AGAGA **GG**AGAG**GG**CA GAACAGGATC GAGAAGTGAT GCCTGCTCAG AGCATGCCAT TGCCCACAGA GCTCGGTGAC
001201 CCTAGACTTT GCGAGGGAAC CCCTCAGGAA GGG**GG**ACTCA G**GG**CAGCCA**G G**CTCCAT**GG**A AAGACCCT**GG** AGG**GG**CA**GG**C ACA**GG**AGACA GCAGTGGCCC
001301 AGAGGAAGCC CAGGGCCCAG CCAACTCCTG GAATGAGCC**G G**GAG**GG**TGAA **GG**CGA**GG**GAC TGCAGAGCCT GGAAATCGGG ATCGCCAGAC TGTCCTTGAG
001401 AGACACAACC GAAGGAGGCA CACAGATTGG CCCACCAGAA ATTAGGGTGC GAGTACACGA TTTAAAGACA CCAAAATTTG CATTTTCCAC AGAAAAAGAG
001501 CCAGAAAGAG AAAGGCGCCT TAGTACCCCA CAGCGAGGGA AGAGACAGGA TGCGTCCTCA AAAGCGGGTA CTGGCCTGAA G**GG**TGAGGA**G G**TGGAA**GG**AG
001601 CCG**GG**TGGAT GCCGGGCAGG GAACCAACCA CACATGCAGA AGCACAGGGG GATGAA**GG**AG AT**GG**AGA**GG**A A**GG**ACTACAG AGGACAAGGA TCACTGA**GG**A
001701 ACA**GG**ACAA**G G**GCAG**GG**AAG ACACAGAAGG ACAGATAAGA ATGCCCAAGT TCAAGATACC CTCCTTAGGA TGGTCGCCAA GCAAGCACAC AAAGACAGGC
001801 AGAGAAAAAG CCACAGAAGA CACAGAGCA**G G**GAA**GG**GAA**G G**AGA**GG**CCAC AGCAACAGCT GATAGAAGAG AACAGAGACG CACAGAGGAA GGATTAAAAG
001901 ACAAAGAAGA CAGTGACTCA ATGACAAACA CAACAAAAAT ACAACTAATA CACGATGAAA AACGCTTAAA AAAGGAACAA ATTCTGACAG AAAAGGAAGT
002001 GGCCACCAAA GACAGCAAGT TCAAAATGCC CAAGTTCAAG ATGCCATTGT TCGGGGCGTC AGCCCCAGGC AAGTCCAT**GG** A**GG**CCTC**GG**T **GG**ATGTGTCT
002101 GCGCCGAAGG TGGAGGCCGA CGTGAGCCTC CTCTCCATGC AGGGGGACCT CAAGACCACT GACCTCAGCG TCCAGACCCC TTCCGCTGAC CT**GG**A**GG**TCC
002201 A**GG**AT**GG**CCA AGTGGATGTG AAACTTCCGG AGGGCCCCCT GCCCGAGGGA GCCAGCCTCA AAGGGCACCT GCCCAAGGTG CAGAGGCCCA GTTTGAAGAT
002301 GCCCAAAGTG GACCTCAAGG GCCCCAAGCT **GG**ACCTGAAA **GG**CCCCAA**GG** C**GG**AAGTGAC AGCCCCCGAT GTGAAGATGT CTCTGTCCAG CAT**GG**A**GG**T**G**
002401 **G**ACGTCCA**GG** CCCCGAGAGC AAAGCTGGAT GGTGCGC**GG**C T**GG**A**GG**G**GG**A CCTGTCCCTG GCCGACAAGG AGGTGACTGC CAAAGACAGC AAGTTCAAAA
002501 TGCCCAAGTT CAAGATGCCA TCATTC**GGGG** TGTC**GG**CCCC A**GG**CAAGTCC AT**GG**A**GG**ACT C**GG**T**GG**ATGT GTCTGCGCCG AAGGTGGAGG CCGACGTGAG
002601 CCTCTCCTCC ATGCAGGGGG ACCTCAAGGC CACTGACCTC AGCATTCAGC CCCCTTCCGC TGACCT**GG**A**G G**TCCA**GG**CT**G G**CCAAGTGGA TGTGAAACTT
002701 CC**GG**AG**GG**CC CTGTGCCCGA **GG**GAGCC**GG**C CCCAAAGTGC ACCTGCCCAA AGTGGAGATG CCCAGTTTCA AGATGCCCAA AGTGGACCTC AAGGGCCCCC
002801 AGATAGATGT TAAGGGCCCC AAGCT**GG**ACC TGAAA**GG**CCC CAA**GG**C**GG**AA GTGACAGCCC CCGAT**GG**CGA **GG**TGTCTCTG CCCAGCAT**GG** A**GG**T**GG**ATGT
002901 CCA**GG**CCCAG AA**GG**CCAAGC T**GG**ATGGTGC GT**GG**CT**GG**A**G G**G**GG**ACCTGT CCCTGGCCGA CAAGGACGTG ACTGCCAAAG ACAGCAAGTT CAAAATGCCC
003001 AAGTTCAAGA TGCCGTCGTT C**GGGG**TATC**G G**CCCCA**GG**GA AGTCCATCAA GGCCTT**GG**T**G G**ATGTGTCTG CACCCAA**GG**T GGA**GG**CCGAC CTGAGTCTCC
003101 CCTCCATGCA GGGGGACCTG AAGACCACTG ACCTCAGCAT TCAGCCTGCT TCTACTGACC TGAA**GG**TCCA **GG**CTGACCA**G G**T**GG**ATGTGA AGCTCCC**GG**A
003201 G**GG**CCACCTG CCCGA**GG**GAG CT**GG**CCTTAA AGGGCACTTG CCCAAGGTGG AGATGCCCAG TTTCAAGATG CCCAAAGT**GG** CCCTCAA**GG**G CCCCCA**GG**T**G**
003301 **G**ACGTCAAGG GCCCCAAGCT GGACCTGAAA AGCCCCAA**GG** C**GG**AAGTCAC AGCCCCTGAT GT**GG**A**GG**TGT CTCTGCCCAG CGT**GG**AGGT**G G**ACGTCGA**GG**
003401 CCCCG**GG**AGC CAAGCTGGAC AGTGCGC**GG**C T**GG**A**GG**G**GG**A ACTGTCCCTG GCCGACAAGG ATGTGACTGC CAAAGACAGC AGGTTCAAAA TGCCCAAGTT
003501 CAAGATGCCA TCGTTCGGGG CGTCAGCCCC A**GG**CAAGTCC ATCGA**GG**CCT C**GG**T**GG**ATGT GTCTGCACCC AAAGTGGAGG CCGACGTGAG TCTCCCCTCC
003601 ATGCAGGGGG ACCTCAAGAC CACTGACCTC AGCATTCAGC CCCCTTCCGC TGACCT**GG**A**G G**TCCACGCT**G G**CCA**GG**TGGA CGTGAAGCTC CT**GG**AG**GG**CC
003701 ACGTGCCTGA **GG**GAGCC**GG**C TTCAAAGGGC ACCTGCCCAA GGTGCAGATG CCTAGTTTGA AGATGCCCAA AGTGGACCTC AAG**GG**CCCCC A**GG**T**GG**AAGT
003801 CA**GG**GGCCCC AAGCTGGACC TGAAA**GG**TCA TAA**GG**CAGA**G G**TGAC**GG**CCC ACGAAGTGGC TGTGTCTCTG CCCAGTGT**GG** AGGT**GG**ACAT GCA**GG**CCCC**G**
003901 **G**GAGCCAAGT TGGATGGCGC ACAGCT**GG**AC GG**GG**ACCTGT CCCT**GG**CTGA CAA**GG**ACGTG ACTGCCAAAG ACAGCAAGTT CAAAATGCCC AAGTTCAAGA
004001 TGCCGTCGTT CGGGGTGTCT GCCCCAGGCA AGTCCATTGA GGCCTCCGT**G G**ACCTGTCTG CACCCAA**GG**T **GG**A**GG**CCGAC ATGAGCCTCC CCTCCATGCA
004101 GGGGGACCTC AAGACCACTG ACCTCAGCAT TCAGCCCCCT TCCACTGACC T**GG**AGCTCCA **GG**CT**GG**CCAA TT**GG**ACGTGA AACTCCCAGA GGGCCCCGTG
004201 CCCGAGGGAG CCGGCCTCAA AGGGCACCTG CCCAAGCTGC AGATGCCCAG TTTCAAGGTG CCCAAAGTGG ACCTCAAGGG CCCTGAAATA GACATCAAGG
004301 GCCCCAAGCT GGACCTAAAA GACCCCAA**GG** T**GG**AAGTGAC AGCCCCTGAT GT**GG**A**GG**TTT CTCTGCCCAG CGT**GG**AGGT**G G**ATGTCGA**GG** CCCCA**GG**AGC
004401 CAAGCTGGAT GGT**GG**AC**GG**C T**GG**A**GG**AGGA CATGTCCCTG GCCGACAAGG ACTTGACTAC CAAAGACAGC AAGTTCAAAA TGCCCAAGTT CAAGATGCCG
004501 TCGTTCGGGG TGTCTGCCCC AGGCAAGTCC ATCGAGGCCT CAGT**GG**ATGT GTCTGCGCCG AA**GG**T**GG**A**GG** CCGACGTGAG CCTCCCCTCC ATGCAGGGGG
004601 ACCTCAAGGC CACTGACCTG AGCATACAGC CCCCTTCTGC TGACCT**GG**A**G G**TCCA**GG**CT**G G**CCAAGTGGA CGTGAAACTC CCAGAGGGCC CTGTGTCCGA
004701 GGGAGCCGGC CTCAAAGGGC ACCTGCCCAA AGTGCAGATG CCCAGTTTCA AGATGCCCAA AGTGGACCTC AAGGGGCCCC AGATAGATGT TAAGGGCCCC
004801 AAGCT**GG**ACC TGAAA**GG**CCC CAA**GG**T**GG**AA GTGACAGCCC CCGATGTGAA GATGTCTCTG TCCAGCAT**GG** A**GG**T**GG**ACGT CCA**GG**CCCCG AGAGCAAAGC
004901 TGGATGGTGC GCAGCT**GG**AG GG**GG**ACCTGT CCCT**GG**CCGA CAA**GG**CGGTG ACTGCCAAAG ACAGCAAGTT CAAAATGCCC AAGTTCAAGA TGCCATCATT
005001 T**GGGG**TGTC**G G**CCCCA**GG**CA AGTCCATCGA GGCCTC**GG**T**G G**ATGTGTCTG AGCCGAA**GG**T **GG**AAGCTGAT GTGAGCCTCC CCTCCATGCA GGGGGACCTG
005101 AAGACCACTG ACCTCAGCAT TCAGTCCCCT TCCGCCGACC T**GG**A**GG**TCCA **GG**CT**GG**CCAA GTGAACGTGA AACTCCC**GG**A G**GG**CCCCCTT CCCGA**GG**GAG
005201 CC**GG**CTTCAA AGGGCACCTC CCCAAGGTGC AGATGCCCAG TTTGAAGATG CCCAAAGTGG CCCTCAAGGG CCCCCAGATG GACGTCAAGG GCCCCAAGCT
005301 GGACCTGAAA **GG**CCCCAA**GG** CGGA**GG**TGAT **GG**CCCCCGAC GTGGAGGTGT CTCTGCCCAG CGT**GG**AGGT**G G**ACGTCGA**GG** CTCCA**GG**AGC CAAGCT**GG**AC
005401 AGTGTGC**GG**C T**GG**AG**GG**TGA CCTGTCCCTG GCCGACAAGG ATGTGACTGC CAAAGACAGC AAGTTCAAAA TGCCCAAGTT CAAGATGCCG TCGTTCGGGG
005501 TGTCTGCCCC A**GG**CAAGTCC ATCGA**GG**CCT C**GG**T**GG**ATGT GTCTGCGCCG AAGGTGGAGG CCGAAGTGAG CCTCCCCTCC ATGCAGGGGG ACCTCAAGAC
005601 CACGGACCTC TGCATTCCGC TCCCTTCTGC AGACCT**GG**T**G G**TCCA**GG**CT**G G**CCAAGTGGA CATGAAGCTC CC**GG**AGGGCC A**GG**TGCCCGA **GG**GAGCC**GG**C
005701 CTCAAAGGGC ACTTGCCCAA GGTGGATATG CCCAGTTTCA AGATGCCCAA AGTGGACCTC AAGGGCCCCC AGACAGATGT TAAGGGCGCC AAGCT**GG**ACC
005801 TGAAA**GG**CCC CAA**GG**C**GG**AA GTGACAGCCC CCGATGTCGA GGTGTCTCTG CCCAGCATGG AGGT**GG**ATGT CCA**GG**CCCAG AA**GG**CTAAGC T**GG**AT**GG**TGC
005901 GC**GG**CT**GG**AG **GG**AGACCTGT CCCTGGCCGA CAAGGACATG ACTGCCAAAG ACAGCAAGTT CAAAATGCCC AAATTCAAGA TGCCGTCGTT CG**GG**GTATC**G**
006001 **G**CCCCA**GG**GA **GG**TCCATCGA GGCCTC**GG**T**G G**ATGTGCCTG CACCCAA**GG**T GGA**GG**CCGAC GTGAGTCTCC CCTCCATGCA GGGGGACCTG AAGACCACTG
006101 ACCTCAGCAT TCAGCCCCCT TCTGCCGACC TGAA**GG**TCCA GACT**GG**CCA**G G**T**GG**ATGTGA AGCTCCC**GG**A G**GG**CCACGTG CCCGA**GG**GAG CT**GG**CCTCAA
006201 AGGGCACCTG CCCAAGGTGG AGATGCCCAG TTTGAAGATG CCCAAAGT**GG** ACCTCAA**GG**G CCCCCA**GG**T**G G**ACATCAAGG GCCCCAAACT GGACCTAAAA
006301 GACCCCAAGG TGGAAATGAG AGTCCCCGAT GTCGAGGTGT CTCTGCCCAG CAT**GG**A**GG**T**G G**ACGTCCA**GG** CCCCAAGAGC CAAGCTGGAT AGTGCGCATC
006401 TGCA**GG**G**GG**A CCTGACCCT**G G**CCAACAA**GG** ACCTGACTAC CAAAGACAGC AAGTTCAAAA TGCCCAAGTT CAAGATGCCG TCGTTTGGGG TGTCTGCCCC
006501 A**GG**CAAGTCC ATCGA**GG**CCT C**GG**T**GG**ATGT GTCTCCACCC AAGGTGGAGG CCGACATGAG TCTCCCCTCC ATGCAGGGGG ACCTCAAGAC CACTGACCTC
006601 AGCATTCAGC CCCTTTCCGC CGACGTGAA**G G**TCCA**GG**CT**G G**CCA**GG**TGGA CGTGAAACTC CT**GG**AG**GG**CC CTGTGCCCGA **GG**AAGTC**GG**C CTCAAAGGGC
006701 ACCTGCCCAA GCTGCAGATG CCCAGTTTCA AGGTGCCCAA AGTGGACCTC AAGGGCCCCG AAATAGACAT CAAGGGCCCC AAGCTGGACC TAAAAGACCC
006801 CAA**GG**T**GG**AA GTGACAGCCC CTGATGT**GG**A **GG**TGTCTCTG CCCAGCGT**GG** AGGT**GG**ACGT CAA**GG**CCCCA **GG**AGCCAAGC TGGATGGTGC GC**GG**CT**GG**A**G**
006901 **G**G**GG**ACATGT CCCTGGCCGA CAAGGACGTG ACTGCCAAAG ACAGCAAGTT CAAAATGCCC AAGTTCAAGA TGCTGTCGTT TGGGGTGTCT GCCCTTGGCA
007001 AGTCCATCGA GGCCTCAGC**G G**ATGTGTCTG CGTTGAA**GG**T **GG**A**GG**CCGAC GTGAGCCTCC CCTCCATGCA GGGGGACCTC AAGACCACTG ACCTCAGCGT
007101 TCAGCCCCCT TCCGCTGACC T**GG**A**GG**TCCA **GG**CT**GG**CCAA GTGGATGTGA AACTCCCAGA GGGCCCCGTG CC**GG**AG**GG**AG CC**GG**CCTCAA A**GG**GCACCTG
007201 CCCAAGCTGC AGATGCCCAG TTTCAAGATG CCCAAAGTAG ATCTCAAGGG CCCCCAGATA GATGTCAAGG GCCCCAAGCT GGACCTGAAA GGCCCCAAGA
007301 C**GG**ACGTGAT **GG**CCCCCGAC GT**GG**A**GG**TGT CTCAGCCCAG CGT**GG**AGGT**G G**ATGTCGA**GG** CCCC**GG**GAGC CAAGCTGGAT GGTGCGT**GG**C T**GG**A**GG**G**GG**A
007401 CCTGTCTGTG GCGGACAAGG ATGTGACTAC CAAAGACAGC AGGTTCAAAA TTCCCAAGTT CAAGATGCCG TCATTCGGGG TGTCTGCCCC A**GG**CAAGTCC
007501 ATCGA**GG**CCT C**GG**T**GG**ATGT GTCTGCGCCG AA**GG**T**GG**A**GG** CCGAC**GG**GAG CCTCTCCTCC ATGCAGGGGG ACCTCAAGGC CACTGACCTC AGCATTCAGC
007601 CCCCTTCCGC TGACCT**GG**A**G G**TCCA**GG**CT**G G**CCAAGTGGA CGTGAAACTC CCAGAGGGCC CTGTGCC**GG**A G**GG**AGCC**GG**C CTCAAA**GG**GC ACCTGCCCAA
007701 GGTGCAGATG CCCAGTTTCA AGATGCCTGA AATGGACCTC AAGGGCCCCC AGCTAGATGT CAAGGGCCCC AAGCT**GG**ACC TGAAA**GG**CCC CAA**GG**C**GG**AA
007801 GTGACAGCCC CCGATGTGGA GATGTCTCTG TCCAGCAT**GG** A**GG**T**GG**ACGT CCA**GG**CCCCG AGAGCAAAGC TGGATGGTGC GC**GG**CT**GG**A**G G**G**GG**ACCTGT
007901 CCCTGGCCGA CAAGGGTGTG ACAGCCAAAG ATAGCAAGTT CAAAATGCCC AAGTTCAAGA TGCCATCATT CA**GG**GTGTC**G G**CCCCA**GG**CG AGTCCATCGA
008001 **GG**CGTT**GG**T**G G**ATGTGTCTG AGCTGAA**GG**T **GG**AAGCCGAC ATGAGCCTCC CCTCCATGCA AGGGGACCTT AAGACCACTG ACATCAGCAT TCAGCCCCCC
008101 TCTGCCCAAC TGGA**GG**TCCA **GG**CT**GG**CCA**G G**TGGATGTGA AACTCCCAGA GGGCCACGTT CCCGAGGGAG CCGGCCTCAA AGGGCACCTG CCCAAGCTGC
008201 AGATGCCCAG TTTCAAGATG CCTGAAGTGG ACCTCAAGGG CCCCCAGATA GATGTTAAGG GCCCCAACGT **GG**ACCTGAAA **GG**CCCCAA**GG** C**GG**AAGTGAC
008301 AGCCCCCGAT GTGAAGATGT CTCTGTCCAG CAT**GG**A**GG**T**G G**ACGTCCA**GG** CCCCGAGAGC AAAGCTGGAT GGTGCGC**GG**C T**GG**A**GG**G**GG**A CCTGTCCCTG
008401 GCCGACAAGG GCATGACAGC CAAAGACAGC AAGTTCAAAA TGCCCAAGTT CAAGATGCCG TCATTC**GGGG** TGTC**GG**CCCC A**GG**CAAGTCC ATCGAGGCCT
008501 C**GG**T**GG**ATGT GTCTGAGCTG AA**GG**T**GG**AAG CTGACGGGAG CTTCCCCTCC ATGCAAGGGG ATCTTAAGAC CACTGACATC CGCATTCAGC CCCCCTCCGC
008601 CCAACTGGAG GTCCA**GG**CT**G G**CCA**GG**T**GG**A CGTGAAACTC CCAGAGGGCC ACGTTCCCGA GGGAGCCGGC CTCAAAGGGC ACCTGCCCAA GGTGCAGATG
008701 CCCAGTTTCA AGATGCCCAA AGTGGATCTC AAGGGCCCCC AGATAGACGT CAAGGGCCCC AAGCTGGACC TGAAA**GG**CCC CAA**GG**CGGA**G G**TGAC**GG**CCC
008801 CCGACGTGGA GGTGTCTCTG CCCAGCGT**GG** A**GG**T**GG**ACGT CGA**GG**CCCCG AGAGCAAAGC T**GG**AT**GG**TGC AC**GG**CT**GG**AG GGTGACCTGT CCCTGGCCGA
008901 CAAGGATGTG ACTGCCAAAG ACAGCAAGTT CAAAATGCCC AAGTTCAAGA TGCCGTCGTT CGGGGTGTCT GCCCCA**GG**CA AGTCCATTGA **GG**TCTC**GG**T**G**
009001 **G**ATGTGTCTG CGCCGAAGGT GGAGGCCGAA GTGAGCCTCC CCTCCATGCA GGGGGACCTG AAGACCACTG ACATCAGCAT TGAGCCCCCC TCTGCCCAAC
009101 TGGAGGTCCA **GG**CT**GG**CCA**G G**T**GG**ACCTGA AGCTCCCAGA GGGCCACGTT CCCGAGGGAG CTGGCCTCAA AGGGCACCTG CCCAAGTTGC AGATGCCCAG
009201 TTTCAAGATG CCCAAAGTAG ATCGCAAGGG ACCCCAGATA GATGTCAAGG GCCCCAAGCT GGACCTGAAA GGCCCGAAGA C**GG**ACGTGAC **GG**CCCCCGAC
009301 GT**GG**A**GG**TGT CTCAGCCC**GG** CAT**GG**A**GG**T**G G**ATGTCGA**GG** CCCCA**GG**AGC CAAGTT**GG**AT **GG**TGCAC**GG**C T**GG**A**GG**G**GG**A CCTGTCCCTG GCCGACAAGG
009401 ATGTGACTGC CAAAGACAGC AAGTTCAAAA TGCCCAAGTT CAAGATGCCG TCGTTCGGGG TGTCTGCCCC A**GG**CAAGTCC ATTGA**GG**TCT T**GG**T**GG**ATGT
009501 GTCTGCGCCA AAGGTGGAGG CCGACCTGAG CCTCCCCTCC ATGCAGGGGG ACCTGAAGAA CACTGACATC AGCATTGAGC CCCCCTCTGC CCAACTGGAG
009601 GTCCA**GG**CT**G G**CCA**GG**T**GG**A CGTGAAGCTC CCAGAGGGCC ACGTTCTCGA GGGAGCTGGC CTCAAAGGGC ACCTGCCCAA GTTGCAGATG CCCAGTTTCA
009701 AGATGCCCAA AGTAGATCGC AAGGGCCCCC AGATAGACAT CAAGGGCCCC AAGCTGGACC TGAAAGGCCC GAAGAT**GG**AT GTGAC**GG**CCC CCGACGT**GG**A
009801 **GG**TGTCTCAG CCCAGCAT**GG** AGGT**GG**ACGT CGA**GG**CCCCA **GG**AGCCAAGT TGGATGGTGC AC**GG**CT**GG**A**G G**G**GG**ACCTGT CCCTGGCCGA CAAGGATGTG
009901 ACTGCCAAAG ACAGCAAGTT CAAAATGCCC AAATTCAAGA TGCCGTCGTA CAGGGCGTCT GCCCCA**GG**CA AGTCCATCCA **GG**CCTC**GG**T**G G**ATGTGTCTG
010001 CGCCGAAGGC GGAGGCCGAC GTGAGCCTCC CCTCCATGCA GGGGGACCTC AAGACCACTG ACCTCAGCAT TCAGCTCCCT TCTGTGGACC TGGAGGTCCA
010101 **GG**CT**GG**CCA**G G**T**GG**ACGTGA AGCTCCC**GG**A G**GG**CCACGTG CCCGA**GG**GAG CT**GG**CCTCAA AGGGCACCTG CCCAAGGTGG AGATGCCCAG TTTCAAGATG
010201 CCCAAAGTGG ACCTCAAGAG CCCCCA**GG**T**G G**ACATCAAG**G G**CCCCAAGCT **GG**ACCTAAAA GTCCCCAA**GG** C**GG**AAGTGAC AGTCCCTGAT GT**GG**A**GG**TGT
010301 CTCTGCCCAG CGT**GG**A**GG**T**G G**ACGTCCA**GG** CCCCGAGAGC CAAGCTGGAT GGTGCGC**GG**C T**GG**A**GG**G**GG**A CCTGTCCCTG GCTGAAAAGG ATGTGACTGC
010401 CAAAGACAGC AAGTTCAAAA TGCCCAAGTT CAAGATGCCC TCCTTCGG**GG** TGTC**GG**CCCC A**GG**CA**GG**TCC ATCGAGGCCT CGCT**GG**ATGT GTCTGCGCCG
010501 AA**GG**T**GG**A**GG** CCGACGTGAG CCTCTCCTCC ATGCAGGGGG ACCTCAAGGC CACTGACCTC AGCATTCAGC CCCCTTCCGC TGACCTGGAG GTCCAGGCTG
010601 TCCAAGT**GG**A TGT**GG**AACTC CT**GG**AG**GG**CC CCGTGCCCGA GGGAGCCGGC CTCAAAGGGC ACCTGCCCAA AGTGGAGATG CCCAGTTTAA AGACGCCCAA
010701 AGTGGACCTC AAGGGCCCCC AGATAGATGT TAAGGGCCCC AAGCTGGACC TGAAAGGCCC CAAGGCAGAA GTGAGAGTCC CCGATGTCGA GGTGTCTCTG
010801 CCCAGCGTGG AGGT**GG**ATGT CCA**GG**CCCCG AA**GG**CCAAGC T**GG**ATGCT**GG** GC**GG**CT**GG**AG **GG**AGACCTGT CCCTGGCTGA CAAGGACGTG ACTGCCAAAG
010901 ACAGCAAGTT CAAAATGCCC AAATTCAAGA TGCCGTCATT CAGGGTATC**G G**CCCCAG**GG**A AGTCCAT**GG**A GGCCTC**GG**TG GATGTGTCTG CACCCAAGGT
011001 GGAAGCCGAT GTGAGTCTCC CCTCCATGCA GGGGGACCTG AAGACCACTG ACCTCAGCAT TCAGCCCCCT TCTGCCGACC TGAA**GG**TCCA **GG**CT**GG**CCAG
011101 AT**GG**ATGTGA AGCTCCC**GG**A GGGCCA**GG**TG CCCGA**GG**GAG CC**GG**CCTCAA AGAGCACCTG CCCAAGGTGG AGATGCCCAG TTTGAAGATG CCCAAAGTGG
011201 ACCTCAAG**GG** CCCCCA**GG**T**G G**ACATCAA**GG** GCCCCAAGCT GGACCTAAAA GTCTCCAA**GG** C**GG**AAGTCAC AGCCCCTGAT GT**GG**A**GG**TGT CTCTGCCCAG
011301 CGT**GG**A**GG**T**G G**ACGTCCA**GG** CCCCAAGAGC CAAACTGGAT AGTGCACAGC T**GG**AGGG**GG**A CCTGTCCCT**G G**CCGACAA**GG** ATGTGACTGC CAAAGACAGC
011401 AAATTCAAAA TGCCCAAGTT CAAGATGCCG TCATTTGGGG TGTCTGCCCC AGGCAAGTCC ATTGAGGCCT C**GG**TGCACGT GTCTGCACCC AA**GG**T**GG**A**GG**
011501 CCGATGTGAG TCTCCCCTCC ATGCAGGGGG ACCTCAAGAC CACTGACCTC AGCATTCAGC CCCATTCTGC CGACCTGACG GTCCAAGCTC GCCAGGTGGA
011601 CATGAAACTC CT**GG**AG**GG**CC ACGTGCCCGA **GG**AAGCC**GG**C CTCAAAGGAC ACCTGCCCAA GGTGCAGATG CCCAGTTTCA AGATGCCCAA AGTCGACCTC
011701 AAGGGCCCTG AAATAGACAT CAAGGGCCCC AAGCTGGACC TAAAAGACCC CAA**GG**T**GG**AA GTGACAGCCC CTGATGT**GG**A **GG**TTTCTCTG CCCAGCGT**GG**
011801 AGGT**GG**ACGT CGA**GG**CCCCA **GG**AGCCAAGC TGGATGGTGC GC**GG**CT**GG**A**G G**G**GG**ACCTGT CCCTGGCCGA CAAGGACATG ACGGCCAAAG ACAGCAAGTT
011901 CAAAATGCCC AAGTTCAAGA TGCCGTCGTT CGGGGTGTCT GCCCCA**GG**CA AGTCCAT**GG**A **GG**CATCAGT**G G**ATGTGACCG CGCCAAAGGT GGAGGCCGAC
012001 GTGAGCCTCC CTTCCATGCA GGGGGACCTC AAGGCCACTG ACCTCAGCGT TCAGCCCCCT TCCGCTGACC T**GG**A**GG**TCCA **GG**CT**GG**CCAA GTGGACGTGA
012101 AACTCCCAGA GGGCCCCGTG CCCGAGGGAG CCAGCCTCAA AGGGCACCTG CCCAAGGTGC AGATGCCCAG TTTCAAGATG CCCAAAGTGG ACCTCAAGGG
012201 CCCCCAGATA GATGTTAAGG GCCCCAAGCT **GG**ACCTGAAA **GG**CCCCAA**GG** C**GG**AAGTGAC AGCCCCTGAT GTGAAGATGT CTCTGTCCAG CAT**GG**A**GG**T**G**
012301 **G**ACGTCCA**GG** CCCCGAGAGC AAAGCTGGAT GGTGTGCAGC T**GG**AGGG**GG**A CCTGTCCCT**G G**CCGACAA**GG** ATGTGACTGC CAAAGACAGC AAGTTCAAAA
012401 TGCCCAAGTT CAAGATGCCA TCATTC**GG**GG TGTC**GG**CCCC A**GG**CAAGTCC AT**GG**AGGCGT CCGTGGATGT GTCTGAGCTG AAGGCGAAAG CCGACGTGAG
012501 CCTCCCCTCC ATGCAGGGGG ACCTCAAGAC CACTGACCTC AGCATTCAGT CCCCTTCCGC CGACCT**GG**A**G G**TCCA**GG**CT**G G**CCAAGTGGA CGTGAAACTC
012601 CC**GG**AG**GG**CC CCCTGCCCAA **GG**GAGCC**GG**C CTCAAAGGGC ACCTCCCCAA GGTGCAGATG CCCTGTTTGA AGATGCCCAA AGTGGCCCTC AAG**GG**CCCCC
012701 A**GG**T**GG**ATGT CAA**GG**GCCCC AAGCT**GG**ACC TGAAA**GG**CCC CAA**GG**C**GG**AT GTGATGACCC CCGTCGTGGA GGTGTCTCTG CCCAGCAT**GG** AGGT**GG**ACGT
012801 CGA**GG**CCCCG **GG**AGCCAAGC T**GG**ACAGTGT GC**GG**CT**GG**AG **GG**TGACCTGT CCCTAGCCGA CAAGGACATG ACTGCCAAAG ACAGCAAGTT CAAAATGCCC
012901 AAGTTCAAGA TGCCGTCGTT CGGGGTGTCT GCCCCAGGCA AGTCCATCGA GGCCTCGTT**G G**ATGTGTCTG CGCTGAA**GG**T **GG**A**GG**CTGAC GTGAGCCTCC
013001 CCTCCATGCA GGGGGACCTG AAGACCACTC ACCTCAGCAT TCAGCCCCCT TCCGCTGATC T**GG**A**GG**TCCA **GG**CT**GG**CCAA GAGGATGTGA AACTCCCAGA
013101 GGGCCCTGTG CATGAGGGAG CCGGCCTCAA AGGGCACCTG CCGAAGCTGC AGATGCCCAG TTTCAAGGTA CCCAAAGTGG ACCTCAAGGG TCCCCAGATA
013201 GACGTTAATG TCCCCAAGCT **GG**ACCTGAAA **GG**CCCCAA**GG** TGGA**GG**TGAC GTCCCCCAAC CTGGACGTGT CTCTGCCCAG CAT**GG**A**GG**T**G G**ACATCCAAG
013301 CCCCA**GG**AGC CAAGCTGGAC AGTACGC**GG**C T**GG**A**GG**G**GG**A CCTGTCCCTG GCTGACAAGG ACGTGACTGC CAAAGACAGC AAGTTCAAAA TGCCCAAGTT
013401 CAAGATGCCA TCCTTTGGGA TGTTGTCCCC A**GG**CAAGTCC ATCGA**GG**TCT C**GG**T**GG**ATGT GTCTGCGCCA AAGATGGAGG CCGACATGAG CATTCCCTCC
013501 ATGCAGGGGG ACCTCAAGAC CACTGACCTC CGCATTCAGG CCCCTTCCGC CGACCTGGAG GTCCA**GG**CT**G G**CCA**GG**T**GG**A CTTGAAACTT CCAGAAGGCC
013601 ACATGCCCGA GGTAGCC**GG**C CTCAAAG**GG**C ACCTGCCCAA **GG**T**GG**AGATG CCCAGTTTCA AGATGCCCAA AGTGGACCTC AA**GG**GCCCCC A**GG**T**GG**ACGT
013701 CAA**GG**GCCCC AAGCTGGACC TGAAA**GG**CCC AAA**GG**CAGA**G G**TGAT**GG**CCC CCGATGTGGA GGTGTCTCTG CCCAGCGT**GG** AGAC**GG**ATGT CCA**GG**CCCCA
013801 **GG**ATCCATGC T**GG**AT**GG**TGC GC**GG**CTTGA**G G**GGGACCTGT CCCTGGCCCA CGAGGATGTA GCTGGGAAAG ACAGTAAGTT TCAAGGACCA AAACTGAGCA
013901 CGTCTGGTTT TGAATGGTCG TCAAAGAAAG TTTCCATGTC TTCCTCTGAA ATCGAAGGAA ATGTTACATT CCATGAGAAG ACTTCCACAT TTCCCATTGT
014001 GGAATCTGTT GTTCATGAAG GTGATCTTCA TGATCCATCT CGCGATGGTA ACTTGG**GG**CT TGCTGTT**GG**A GAAGTT**GG**AA T**GG**ATTCGAA GTTTAAGAAA
014101 CTGCATTTTA AAGTGCCCAA AGTTTCATTT TCTTCTACCA AAACTCCTAA AGATAGTTTA GTCCCAGGTG CAAAGTCTAG CATAGGTCTT TCCACGATTC
014201 CTTTATCATC TTCAGAATGC TCAAGTTTTG AATTACAACA GGTTTCGGCT TGTTCAGAGC CATCCATGCA GATGCCTAAG GTGGGTTTTG CTGGGTTTCC
014301 ATCATCCCGG CTTGATCTCA CTGGTCCTCA CTTTGAATCT TCTATTCTCT CTCCCTGTGA GGATGTTACA CTTACAAAAT ACCAGGTGAC TGTTCCCAGA
014401 GCTGCCTTGG CCCCTGAGCT TGCTCTGGAA ATTCCTTCTG GGTCTCAGGC TGATATTCCT CTTCCCAAGA CAGAGTGCTC CACTGACCTG CAGCCTCCAG
014501 AGGGAGTTCC AACATCTCAA GCTGAGAGTC ACTCTGGCCC ACTGAATTCC ATGATTCCTG TTTCTCTTGG TCAGGTATCT TTTCCTAAAT TCTATAAACC
014601 AAAGTTTGTG TTTTCAGTCC CCCAAATGGC AGTTCCTGAG GGAGACCTAC ATGCAGCAGT GGGTGCCCCA GTCATGTCTC CTCTTAGCCC TGGAGAAAGA
014701 GTGCAGTGCC CCTTGCCAAG CACCCAGCTG CCATCCCCAG GCACCTGTGT GTCTCAGGGC CCAGAAGAGC TTGTGGCCTC CTTGCAGACA TCAGTAGTGG
014801 CCCCTGGAGA AGCCCCTTCT GAAGATGCTG ACCACGAAGG GAAAGGGAGT CCCTTGAAAA TGCCTAAGAT TAAGCTTCCA TCATTTAGGT GGTCCCCGAA
014901 GAA**GG**AAACA **GG**GCCAAA**GG** T**GG**ACCCAGA ATGCAGCGTG GAGGACTCAA AACTCAGCCT GGTTTTAGAC AAGGATGAAG TGGCCCCGCA GTCTGCCATC
015001 CACATGGATC TGCCTCCTGA GA**GG**GAT**GG**A GAGAA**GG**GGA **GG**AGCACAAA GCCTGGCTTT GCCATGCCAA AACTTGCACT TCCCAAAATG AAGGCTTCTA
015101 AGAGTGGGGT CAGCCTGCCA CAGAGAGACG TGGATCCTTC CCTTTCTAGT GCCACAGCAG GGGGTAGCTT TCAAGACACA GAAAAGGCCA GCAGTGACGG
015201 T**GG**TAG**GG**GA **GG**ACTT**GG**TG CAACAGCAAG TGCCACAGGA AGTGAGGGTG TGAACCTCCA CCGGCCACAG GTCCACATTC CCAGTTTGGG CTTTGCCAAA
015301 CCTGATCTCA GATCCTCCAA **GG**CCAA**GG**T**G G**A**GG**TGAGCC AGCCTGAAGC TGACCTGCCT CTTCCCAAAC ATGATCTGTC TACCGAAGGT GACAGCAGA**G**
015401 **G**ATGT**GG**GCT C**GGGG**ATGTC CCAGTGAGCC AGCCTTGTGG GGAGGGGATA GCCCCCACAC CTGAAGATCC CCTCCAGCCA TCCTGTAGAA AACCAGATGC
015501 TGAAGTCCTC ACAGTGGAAA GCCCAGAGGA GGAAGCCATG ACCAAGTACT CGCAGGAAAG CTGGTTTAAA ATGCCCAAGT TCCGCATGCC CAGCCTTAGG
015601 CGCTCTTTCA **GG**GACAGA**GG** CGGGGCT**GG**A AAGCT**GG**AAG T**GG**CTCAGAC ACA**GG**CACC**G G**CAGCAACA**G G**GGGTGAAGC AGCAGCTAAA GTCAAAGAGT
015701 TCCTTGTTTC TGGGTCAAAC GTGGAGGCAG CTATGTCCCT ACAGCTCCCA GAGGCAGATG CAGAAGTGAC AGCTTCTGAG AGCAAATCAT CCACAGATAT
015801 TCTAAGGTGT GATCTTGACA GCACAGGCTT GAAGCTGCAC CTCTCCACTG CTGGGATGAC T**GGGG**ATGAG CTTTCCACTT CTGA**GG**TCA**G G**ATCCATCCA
015901 TCCAAAGGAC CTCTCCCTTT TCAGATGCCT GGCATGAGGC TTCCAGAAAC CCAGGTTCTT CCAGGAGAAA TAGATGAGAC TCCTCTTTCC AAGCCAGGAC
016001 ATGACCTTGC CAGCATGGAG GATAAAACAG AGAAATGGTC TTCCCAGCCT GAAGGTCCAC TTAAATTGAA AGCTTCAAGT ACTGATATGC CATCCCAGAT
016101 TTCTGTGGTT AATGTGGATC AACTGTGGGA AGATTCTGTC CTAACTGTCA AATTCCCCAA ATTAATGGTA CCAAGGTTCT CCTTCCCTGC CCCCAGCTCA
016201 GAGGATGATG TGTTCATCCC CACTGTGAGG GAAGTGCAGT GTCCAGAGGC CAATATTGAT ACAGCCCTTT GTAA**GG**AAAG TCC**GGGG**CTC T**GG**GGAGCCA
016301 GCATCCTGAA **GG**CA**GG**TGCT **GGGG**TCCCTG GGGAGCAGCC TGTGGACCTT AACCTGCCTT TGGAAGCTCC CCCAATTTCA AAGGTCAGAG TGCATATTCA
016401 GGGTGCTCAG GTTGAAAGTC AAGAGGTCAC TATACACAGC ATAGTGACAC CAGAGTTTGT AGATCTCTCA GTACCCAGGA CTTTTTCCAC TCAGATTGTG
016501 CGGGAATCAG AGATCCCCAC GTCAGAGATT CAAACACCTT CGTACGGATT TTCCTTATTA AAAGTGAAAA TCCCAGAGCC CCACACGCAG GCTAGAGTGT
016601 ACACAACAAT GACTCAACAC TCTA**GG**ACTC A**GG**AG**GG**CAC AGAAGA**GG**CT CCCATACAAG CCACCCCAGG AGTAGACTCC ATTTCTGGAG ATCTCCAGCC
016701 TGACACTGGA GAACCATTTG AGATGATCTC TTCCAGCGTC AATGTACTGG GACAGCAAAC ACTCACATTT GAAGTTCCTT CTGGCCACCA GCTTGCAGAC
016801 AGCTGTTCAG ATGAGGAGCC AGCAGAAATT CTTGAGTTTC CCCCTGATGA TAGCCAAGAG GCAACCACAC CACTGGCAGA TGAAGGCAGG GCTCCAAAAG
016901 ACAAACCAGA AAGTAAAAAA TCTGGTCTGC TCTGGTTTTG GCTTCCAAAC ATTGGGTTTT CCTCTTCTGT TGATGAGACA GGTGTTGATT CCAAAAATGA
017001 CGTCCAGAGA TCTGCTCCCA TTCAAACACA GCCTGAGGCA CGACCAGAGG CAGAACTGCC TAAAAAACA**G G**AGAA**GG**CA**G G**CT**GG**TTCCG ATTTCCCAAA
017101 TTAGGGTTCT CCTCATCTCC TACCAAGAAA AGCAAAAGCA CCGAAGATGG GGCAGAGCTG GAAGAACAAA AACTTCAAGA AGAAACAATC ACGTTTTTTG
017201 ATGCCCGAGA AAGTTTCTCC CCTGAAGAGA AGGAAGAGGG TGAACTGATC G**GG**CCTGT**GG** GCACT**GG**GCT **GG**ACTCCAGA GTGATGGTGA CATCCGCGGC
017301 AAGAACAGAG TTAATCCTGC CCGAGCAGGA CAGAAAAGCT GACGATGAAA GCAAA**GG**GTC A**GG**CCTG**GG**A CCAAATGAA**G G**CTGAGAGGT ATGGCTCATC
017401 AGTACAAGAG AGATGCAAAA AACTAAGTTG GAAAGTAAAG GCTACACACA CATATGGAGC ACCCCATCCC ACAGCACATT ACATCCACCT CACTTCACAG
017501 AACGGAGAAC AGAGCAGAAA TGACCAGAAC ACCTTTGTCA CCATCACACA GCCCTCCTAA AATGGAACCA AAGCTTCCCA GCTCCCTCAA AGCTTTGGAT
017601 GCAAAGAAGG CACCCTGACT TCCACAAGAC ACCAGAATTC ACAC**GG**TACT CAGA**GG**CACT GCT**GGGG**AAG TTTGTTGGTC TTTATTAGAT AAATTTCCAG
017701 AGACCTGTCC ATAATACCCA ACAGAACATG ACTGTTTCTT TGAGGAAAGG GTTATAATGT CTGTGGTGTA CAAGTCGTTT TTGGTATAAC TTCTTTCCTG
017801 CTGCTGCTGC TTCCCGGCAA ACATAGTTTT CCTATTTCAG GCAGAGTGCG GTATATTCCA GGAAACACTG TTTCCTACTC ACTTAGCTTA CTTCTTTGTT
017901 GAATGCCTCA CTAATGGCAA GTTTCAAGAT GTTTTGGGTG ACAATGCACA CATGCTG**GG**C AAAA**GG**GTGA T**GG**CCAGT**GG** CTGGCAGCTG GGCCAGCAGA
018001 AGCTAGGACA TCTGTGAGTT GTCATTCTCA TCTATCCATG TCCACTGGCC TGCCAGCATC CGCCAGTGCC TTGCCAGTGT GCACGGTCCC ACACTGTGGC
018101 CCCTGAGTCC CCTAATGTAC ACGCTGCAGC CAGAATGCAG ATGGAGCT**GG** CTT**GG**CTGTT CCCT**GG**ATG**G G**CAATAAAGA AAGTGCTGCA TCCCA

**MAP1B**

000001 ATGGCATCTT GGTGCACGTC TGTGCCATTC CTCTCAATAT TTCCAAAGAT GGACAAAAGA GAACATCGTT AGTTGAATTC CTGGGCAAAC TGGTCTGAAG
000101 ATTGACTT**GG** AA**GG**AGT**GG**A **GG**ACAAAAGA TCCTTCATCA CCGAAGTGAC GTTTTAGAAA CAGTGGTCCT GATCAACCCT TCTGATGAAG CAGTCAGCAC
000201 CGAGGTGCGC TTAATGATCA CTGATGCTGC CCGACACAAG CTGCTCGTGC TGACCGGGCA GTGCTTTGAA AATACCGGAG AGCTCATTCT CCAGTCCGGC
000301 TCTTTCTCCT TCCAGAACTT CATAGAGATT TTCACCGATC AAGAGATCGG GGAGTTACTA AGCACCACCC ATCCTGCCAA CAAAGCCAGC TTAACCCTGT
000401 TCTGTCCTGA AGAAGGGGAC TGGAAGAACT CCAATCTTGA CAGACACAAT CTCCAAGACT TCATCAATAT TAAACTCAAT TCAGCTTCTA TCTTGCCAGA
000501 AATGGAAGGA CTTTCTGAGT TTACCGAGTA TCTCTCAGAA TCAGTGGAAG TCCCATCTCC CTTTGACATC TTGGAACCTC CCACATCGGG TGGATTTCTG
000601 AAGCTCTCCA AGCCCTGCTG TTATATTTTT CCA**GG**A**GG**GA **GGGG**CGATTC TGCCTTGTTT GCAGTGAATG GTTTCAATAT GCTCATCAAT GGCGGATCAG
000701 AGAGAAAATC CTGCTTCTGG AAGCTCATCC GACACTTAGA CCGAGTGGAC TCCATCCTGC TCACCCACAT TGGGGATGAC AATTTGCCTG GAATAAACAG
000801 CATGTTACAG CGGAAAATTG CAGAGCTCGA GGAAGAACAG TCCCAGGGCT CCACCACAAA TAGTGACTGG ATGAAAAACC TCATCTCCCC TGACTTAGGA
000901 GTTGTATTTC TCAATGTACC TGAAAATCTC AAAAATCCAG AGCCAAACAT CAAGATGAAG AGAAGCATAG AAGAAGCCTG CTTCACTCTC CAGTACCTAA
001001 ACAAATTGTC CATGAAACCA GAACCTCTGT TTAGAAGTGT AGGCAATACT ATTGATCCTG TCATTCTTTT CCAAAAAATG GGAGTAGGTA AACTTGAGAT
001101 GTATGTGCTT AATCCAGTCA AGAGCAGCAA GGAAATGCAG TATTTTATGC AGCAGTGGAC TGGTACCAAC AAAGACAAGG CTGAATTCAT TCTGCCTAAT
001201 GGTCAAGAAG TAGATCTCCC GATTTCCTAC TTAACTTCAG TCTCATCTTT GATTGTGTGG CATCCAGCAA ACCCTGCGGA GAAAATCATC CGAGTCCTGT
001301 TTCCTGGGAA CAGCACCCAG TACAACATCC TGGAAGGGTT GGAAAAGCTC AAACATCTAG ACTTTCTGAA GCAGCCACT**G G**CCACCCAAA A**GG**ATCTCAC
001401 T**GG**CCA**GG**TG CCCACTCCTG TGGTGAAACA AACAAAACTG AAACAGAGGG CTGATAGCCG AGAAAGTCTG AAGCCAGCCG CAAAACCACT TCCTAGCAAA
001501 TCCGTGCGCA AGGAGTCAAA AGAAGAAACC CCTGAGGTCA CAAAAGTGAA TCACGTGGAA AAGCCACCCA AAGTTGAAAG CAAAGAAAAG GTAATGGTGA
001601 AAAAAGACAA GCCAATAAAA ACAGAGACCA AACCTTCAGT GACTGAAAAG GAGGTTCCCA GCAAAGAAGA GCCATCTCCA GTGAAAGCCG AGGTGGCTGA
001701 GAAGCAAGCC ACAGATGTCA AACCCAAAGC TGCCAA**GG**AG AAGAC**GG**TGA AAAA**GG**AAAC AAA**GG**TAAAG CCTGAAGACA AGAAAGAGGA GAAAGAAAAG
001801 CCAAAGAAAG AAGTGGCTAA AAAGGAGGAC AAAACACCTA TCAAGAA**GG**A **GG**AAAAACCA AAAAA**GG**AAG A**GG**TGAAAAA AGAAGTCAAA AAAGAGATCA
001901 AGAAAGAAGA GAAAAAAGAA CCCAAGAAAG AGGTTAAGAA AGAAACACCG CCAAAGGAAG TCAAGAAGGA AGTTAAGAAG GAAGAGAAGA AGGAAGTGAA
002001 AAAGGAAGAA AAGGAACCCA AAAAAGAAAT TAAGAAGCTC CCTAAAGACG CAAAGAAATC ATCTACTCCT CTGTCTGAAG CAAAAAAACC AGCTGCTTTA
002101 AAACCAAAAG TACCCAAGAA GGAAGAGTCT GTCAAGAAAG ATTCTGTTGC TGCC**GG**AAAG CCAAA**GG**AGA A**GGGG**AAAAT AAAAGTCATT AAGAA**GG**AA**G**
002201 **G**CAA**GG**CCGC AGA**GG**CTGTC GCTGCAGCTG TCGGCACTGG AGCCACCACA GCAGCTGTCA T**GG**C**GG**CAGC T**GG**AATAGCA GCCATT**GG**CC CTGCCAAAGA
002301 ACTCGAAGCT GAGAGGTCCC TTATGTCATC TCCTGAGGAT CTAACCAAGG ACTTTGAAGA GTTAAAGGCT GAAGAGGTCG ATGTAACAAA GGACATCAAG
002401 CCTCAGCTGG AGCTAATCGA AGACGAAGAG AAACTGAAGG AAACTGAGCC AGTCGAAGCC TACGTCATCC AGAAGGAGAG AGAAGTCACC AAAGGTCCTG
002501 CCGAGTCCCC TGATGAG**GG**A ATCACTACCA CTGAA**GGGG**A **GG**GCGAATGT GAACAGACAC CTGAGGAGCT GGAGCCCGTC GAGAAGCAGG GAGTAGACGA
002601 CATTGAAAAA TTTGAAGATG AAGGAGCCGG TTTTGAAGAA TCTTCAGAGA CTGGAGACTA TGAAGAGAA**G G**CAGAAACTG A**GG**A**GG**CTGA **GG**AGCCAGAA
002701 GA**GG**AT**GGGG** A**GG**AACACGT ATGTGTGAGC GCCTCCAAGC ACAGCCCCAC TGA**GG**ATGA**G G**AAAGTGCCA A**GG**CGGA**GG**C TGATGCATAC ATCAG**GG**AGA
002801 AGAG**GG**AGTC TGT**GG**CCAGT GG**GG**ATGACC GAGCCGAAGA AGACAT**GG**AT GA**GG**CCATTG AGAAA**GG**AGA **GG**CTGAACAA TCTGAAGA**GG** A**GG**CTGATGA
002901 **GG**A**GG**ACAAA GCTGAAGATG CCAGAGA**GG**A **GG**AATATGAG CC**GG**AAAAAA T**GG**AAGCTGA AGACTATGTG ATGGCTGT**GG** TCGACAA**GG**C TGCAGA**GG**CT
003001 GGT**GG**TGCCG AGGAGCAGTA TGGATTCCTC ACCACACCAA CCAAGCAACT AGGAGCCCAG TCTCCTGGCC GAGAACCTGC ATCTTCAATT CATGATGAGA
003101 CTTTACCTGG AGGCTCAGAG AGCGAGGCCA CCGCTTCTGA TGAGGAGAAT CGAGAAGACC AGCCTGAGGA ATTCACTGCC ACCTCTGGCT ACACTCAGTC
003201 TACTATTGAG ATATCCAGTG AGCCCACCCC CATGGATGAG ATGTCTACCC CTCGAGACGT GATGAGTGAT GAGACCAACA ATGAAGAGAC GGAGTCCCCT
003301 TCTCAGGAAT TCGTAAATAT CACCAAATAT GAATCTTCAT TGTATTCTCA GGAATACTCT AAACCTGCTG ATGTTACACC GCTCAACGGA TTTTCTGAAG
003401 GATCAAAAAC AGATGCCACT GATGGCAAGG ATTACAATGC TTCAGCCTCT ACCATATCAC CACCCTCTTC CATGGAGGAA GACAAATTCA GCAGATCTGC
003501 TTTACGTGAT GCTTACTGCT CTGAAGTGAA AGCCAGCACC ACTTTGGACA TCAAAGATAG CATCTCAGCT GTTTCAAGTG AAAAGGTCAG CCCATCGAAG
003601 AGCCCGTCCC TGAGTCCATC TCCACCATCA CCCTTAGAAA AGACCCCCCT GGGTGAACGT AGTGTGAACT TCTCTCTGAC GCCCAATGAG ATTAAAGTCT
003701 CTGCAGAGGC AGAAGTAGCC CCGGTGTCTC CTGAGGTGAC CCAAGAAGTA GTTGAAGAAC ATTGTGCTAG TCCTGAGGAC AAGACTCTGG AAGTGGTGTC
003801 ACCATCTCAG TCCGTGACTG GCAGTGCTGG TCACACACCT TACTATCAAT CTCCTACTGA CGAGAAATCC AGTCATCTCC CTACAGAAGT CATTGAAAAA
003901 CCACCAGCAG TTCCAGTGAG TTTTGAATTC AGTGATGCCA AAGATGAGAA TGAAAGGGCT TCAGTAAGCC CCATGGATGA GCCCGTGCCT GACTCAGAGT
004001 CTCCTATTGA AAAAGTTTTG TCTCCTTTAC GCAGCCCGCC CCTCATTGGA TCCGAGTCTG CTTATGAAAG TTTTCTAAGT GCTGATGACA AGGCTTCTGG
004101 CAGAGGTGCC GAAAGTCCTT TTGAAGAAAA GAGTGGAAAA CAAGGCTCTC CAGACCAAGT AAGTCCAGTT TCTGAAATGA CTTCTACTAG TCTTTACCAA
004201 GACAAACAGG AAGGGAAAAG CACAGACTTT GCACCAATAA AAGAAGACTT TGGCCAAGAA AAGAAAACTG ATGATGTTGA AGCCATGAGT TCTCAACCAG
004301 CACT**GG**CTCT **GG**ATGAAA**GG** AAATTA**GG**AG ATGTTTCTCC CACACAAATA GATGTCAGTC AGTTTGGATC TTTTAAAGAA GACACTAAGA TGTCCATTTC
004401 TGAAGGTACT GTCTCAGACA AGTCAGCTAC TCCTGTTGAT GAGGGCGTAG CAGAAGACAC GTACTCTCAT ATGGAGGGTG TGGCCTCAGT GTCCACAGCC
004501 TCAGTGGCTA CGAGCTCATT TCCAGAGCCA ACAACAGATG ATGTGTCTCC ATCTCTGCAT GCTGAGGTTG GCTCCCCACA TTCCACAGAA GTAGATGACT
004601 CCCTTTCAGT GTCTGTTGTG CAAACACCTA CCACATTCCA GGAAACAGAA ATGTCTCCAT CTAAAGAAGA ATGCCCAAGA CCGATGTCAA TTTCTCCACC
004701 AGATTTCTCC CCTAAAACTG CAAAGTCCAG GACACCCGTT CAAGATCACA GATCTGAACA GTCCTCAATG TCTATTGAAT TTGGCCAAGA ATCTCCTGAG
004801 CAATCCCTTG CTATGGACTT CAGTCGACAG TCTCCAGATC ACCCTACAGT GGGTGCAGGC GTGCTTCACA TCACTGAAAA TGGGCCAACT GAAGTGGACT
004901 ACAGTCCTTC TGACATGCAG GACTCCAGTT TATCACATAA GATACCACCT ATGGAGGAGC CGTCCTACAC CCAAGATAAT GATCTTTCTG AGCTCATCTC
005001 AGTATCTCAG GTAGAGGCCT CCCCGTCCAC CTCTTCTGCT CATACCCCTT CTCAGATCGC TTCTCCTCTC CAAGAAGATA CTCTATCCGA TGTTGCTCCT
005101 CCCAGAGATA TGTCCTTATA TGCCTCACTC ACCTCTGAAA AAGTGCAAAG TCTGGAAGGA GAGAAGCTCT CTCCAAAATC TGATATCTCT CCACTCACCC
005201 CACGAGAGTC CTCTCCTTTA TATTCACCTA CTTTTTCAGA TTCTACCTCT GCAGTCAAAG AGAAAACAGC AACTTGCCAC AGTTCCTCTT CTCCACCAAT
005301 AGATGCAGCA TCCGCAGAGC CCTATGGCTT CCGTGCCTCA GTGTTATTCG ATACAATGCA ACACCATCTA GCCTTGAATA GAGATTTGTC CACACCT**GG**C
005401 CT**GG**AGAA**GG** ACAGT**GG**AGG GAAGACACCT GGTGACTTTA GCTATGCCTA TCAAAAGCCT GAGGAAACAA CCAGGTCCCC AGATGAAGAA GATTATGACT
005501 ATGAGTCTTA TGAGAAGACC ACCCGGACCT CAGATGTGGG TGGCTATTAC TATGAGAAGA TAGAGAGAAC CACAAAATCT CCAAGTGACA GTGGCTACTC
005601 CTATGAGACC ATTGGGAAAA CTACCAAGAC CCCTGAAGAT GGTGACTATT CCTATGAAAT TATTGAGAAG ACCACACGGA CCCCTGAAGA GGGTGGGTAC
005701 TCATATGACA TAAGTGAAAA GACCACCAGC CCCCCCGAAG TGAGTGGTTA CAGCTATGAA AAGACTGAGA GGTCTAGAAG GCTTCTGGAT GACATCAGCA
005801 ATGGCTATGA TGACTCTGA**G G**AT**GG**T**GG**CC ACACACTT**GG** GGACCCCAGC TACTCTTATG AAACCACTGA GAAAATTACC AGTTTCCCTG AGTCTGAAGG
005901 TTATTCCTAT GAGACATCTA CAAAGACAAC ACGAACCCCT GATACTTCCA CATACTGTTA CGAGACTGCA GAGAAAATCA CTAGAACCCC TCAGGCATCC
006001 ACATATTCCT ACGAGACTTC AGACCTATGC TACACTGCAG AAAAGAAGTC CCCCTCAGAA GCCCGTCAGG ATGTCGATTT ATGCCTCGTG TCCTCTTGTG
006101 AATACAAGCA CCCCAAGACA GAGCTTTCAC CCTCTTTCAT TAATCCCAAT CCTCTTGAGT GGTTTGCCAG TGAAGAACCC ACTGAAGAAT CTGAAAAGCC
006201 CCTCACTCAA TCA**GG**G**GG**AG CCCCACCGCC TCCA**GG**A**GG**A AAGCAACAGG GCCGACAGTG TGATGAAACC CCTCCCACCT CAGTCAGCGA GTCAGCCCCA
006301 TCCCAGACCG ACTCTGATGT TCCCCCGGAG ACTGAAGAGT GCCCCTCCAT CACGGCCGAT GCCAATATCG ACTCTGAAGA CGAGTCGGAA ACCATCCCCA
006401 CAGACAAAAC TGTCACGTAC AAACACATGG ACCCACCTCC AGCTCCCGTG CAAGACCGCA GCCCTTCGCC ACGCCACCCT GATGTGTCCA T**GG**T**GG**ACCC
006501 AGA**GG**CCTT**G G**CCATTGAGC AGAACCTGGG CAAAGCTCTA AAGAAAGATC TGAAAGAGAA GACCAAAACC AAAAAGCCAG GTACAAAGAC CAAGTCATCT
006601 TCACCTGTCA AAAAGAGTGA TGGGAAGTCT AAGCCCTTGG CAGCTTCACC AAAACCAGCG GGCTTGAAAG AATCCTCGGA TAAAGTGTCC AGGGTGGCTT
006701 CTCCTAAGAA GAAAGAATCT GTGGAAAAGG CAGCAAAACC CACCACCACT CCTGAGGTCA AAGCTGCACG TGGGGAAGAG AAAGACAAGG AGACCAAGAA
006801 TGCTGCCAAT GCCTCTGCAT CCAAGTCGGC CAAGACCGCC ACTGCAGGAC CAGGAACTAC CAAGACGACC AAGTCATCTG CTGTGCCCCC AGGCCTCCCT
006901 GTGTATTTGG ACCTGTGCTA CATTCCTAAC CACAGCAATA GTAAGAATGT TGATGTGGAA TTTTTCAAGA GAGTGCGGTC TTCCTACTAC GTGGTGAGTG
007001 GGAATGACCC TGCTGCTGAG GAGCCCAGCC GGGCTGTCCT GGACGCTTTG TT**GG**AA**GG**AA A**GG**CTCAGT**G G**GGCAGCAAC ATGCAGGTGA CACTGATCCC
007101 AACTCATGAC TCAGAAGTGA TGAGGGAATG GTACCAGGAG ACCCATGAGA AACAGCAAGA TCTCAACATC ATGGTTTTAG CAAGCAGCAG CACAGTGGTT
007201 ATGCAAGATG AATCCTTCCC TGCATGCAAG ATTGAACTGT AAAAACCAAG GCCAGCCACA CCACAGGATC TGAACTTTGT TTCCAGAAAT TCTTCAATTT
007301 GAAATCACCT TTTCTAAAAA GTCAATTCAT CTAGTTAAGT CGCTGAACAA TTACCTGCCA AATGCTATAC TGTGTCATGG TGATGCAAGT CACTAAATTT
007401 CTCAGTTTTT GCTGATTGCT AAGGGAAATA ACAGTATTTC CACAATAGGG TTCAAATTCC TGCAAAATTA CCTACCCCAG TTCATCTCTG CTGAACATTT
007501 GGAAACCATG CACTAGCCAA CCCAACTGAC TTCTGCTAGG TAGAGGCATT TGTCTTAGAG AGAGAGAGAG CGCGGGAGAG AGTGAGAGAG AGTGAGAGCA
007601 CAAAGATAAC GCAGGAGAGA GAGAGAGAAA GAATGAGAAA GAAAAGGAAT GCAAGAGAAG GAGATGTAAT GACAGAGAGT TCTGGTGAGA TACCCAGAGA
007701 GAAAAAGAGA GAGCAG**GG**TG G**GG**TAA**GG**A**G G**AGAAAATAA ACCAACAATT AGGTCTGCAT TTTCTCAGGC AGTAGGCATT CTTTAGTCTA CATAGGCAAA
007801 GTTTTCCATT TTTGTCAGTC TGAGTCATCA AAAAGAGTCT TAATTTTCTA AAACAAGTTG GCTAGAAGAA AGTAAAAAGA ACAACACTTG TTATGAGGGC
007901 ATGTGATATT TTCACATCTT AATTAAGCTC CTTCAGTTTG AAGGCTGCAC ACTGACATAA TGTAGTGAGT GTAGACTGGC CATGCAAGTG GTTTGGGCCC
008001 CATTCAGAAC TCTCAGACTC TAAACACACA AGTAGATTGA TCTAAGGCAT GCTCCCAGCA TTTGTCCACC CACTTAGTCC ACTCTGAGTC GATTAACCTG
008101 CATGCAGCAA CACCCAAGTC CACCCCAATT AACTGAAGCA AATACCAAAG CAGTTGGGAG TACATATGGT AGACAATTTG CCTTAGGAAG TGACTTGAAT
008201 GTACAAAGAT ACTTGATGCA CTTATTTTTT AATGTGAGAC AGCAAGTTTA TAAAACATCC ATATAGGATT ATAGATACTT AAA**GG**AACAC GTG**GG**TGAGC
008301 GTGTGT**GG**G**G G**TACTAGAAG CTGATCTGAT TGGTCCAACA GTTTGATGCT GAGTCATGCG TGTTGAATCC CACTTCAGTG CACCTGTGGC CTCTCAGTCA
008401 AACAAGTTGT GCCTTTCACA GCTTCTTTAC TACTGCAAGT TCAAGACTGA AATGGCTTCT ATGATCAGAA CTG**GG**AAAAC AGTGAATCTT AT**GG**T**GG**AAG
008501 A**GG**TTCTCAG CAAGTGTACA GTATTTACCT TCCTTTGTCT TACATTGGCT TTTTAAATTT TCCATTAATT TCAACATAAT TATGGGAACA AGTGTACAGA
008601 AGAATTTTTT TTTTAAGATA TGTGAGAACT TTTCATAGAT GAACTTTTTA ACAAATGTTT TCATTTACAG GAAATTGCAA AGAAAATTCT CAAGTGATAG
008701 TCTTTTTTTT TAAGTGTTTC GTAAGACAAA AATTGAATAA TGTTTTTTGA AGTTCTGGCA AGATTGAAGT CTGATATTGC AGTAATGATA TTTATTAAAA
008801 ACCCATAACT ACCAGGAATA ATGATACCTC CCACCCCTTG ATTCCCATAA CATAAAAGTG CTACTTGAGA GTGGG**GG**AGA AT**GG**CAT**GG**T A**GG**CTACTTT
008901 TCAGGGCCTT GACAAGTACA TCACCCAGTG GTATCCTACA TACTTCTTTC AAGATCTTCA ACCATGAGGT AAAAGAGCCA AGTTCAAAGA ACCCTAGCAC
009001 AAATTTGCTT TGGGATTTTC TTTTCTGGAA AAAAAAAATA AAAGAAATAG TACATTGAAA ACAAATGAAT TCTCAACTCC TACGGTTCAT GTAGAGTTTA
009101 GAGAAAATTT CCATCATTGT CATCATTGAA CTGTGAACCT GGGAAGCCAG ATCATGATTA ACACTGACAT CAAGTTTCAA GTTGCAGATC AATGCACCCA
009201 GTGTTCAGAT GAGGCAAACT TCTCCGTGAC AACTGTGCTG TGCTCTGTCA CATTACATTT CCTGCAGACT CTAAGATCTA CGGAGTAGAG AACAATGACC
009301 TCATTTTATT TTCTATGTTA GTTATTTATT TCAAAATTAA CATTTTAGTT GATTTTTGTC TGATAAGTCT ATGTTTTGCA CTGCTAACTA TGATGAGGGT
009401 TTAAAAAAAT GCTTCTTCAG GGTCCTTTCA CTGAGGACCT ATGCAGTCTA CTTAATGCTG TGAATTACAT TTTTCAAATG TTTAATTTTT TAAAGAAAAT
009501 TAATATTCTA TTTTTGTTAG GCTTCTCTAG AAATGCAGCT TTTATTTATT ACCCCATTTC TTTCAAGTCC TTGGAAAATA ACATATTAAG GGTACAAGAA
009601 ATTAACACAT GATGGAAAAG TCATTGTGAC GCCAATGAAT TTCATTGAGT ATAAACTCAT CTACTTCAAA TTTATTTTAT AACACAACCT AAGATACTCA
009701 AGATAATTAT TTAATGGTTA GCTCTTAAGT TGAATTGGTC TACATAATGC GTGGGAAGAA AACCAGATTT TTAGCCTTCT TGCCAAATCC AGACCTCTGG
009801 TTGATTTTTC TTTGACAGAA GATGCAAGTT ATTTTCCAAT TTCACAATTA AATGTATTTA ACCTGAACAT TATTTTGCTT TAAAAACTAT AAACATTGTA
009901 GGAGAATTAT AGCCAGTCTT CAGTTATAAC CACTCCACCC TCCTCACTTT CTCTCTCTCT CTCTCTTTTT TTTTTTTTTT TTTTTTGCTA TGGGATTTAA
010001 TGGGAAAAAT ATGTAAAAAC TGTCACTAGT CAGCTGGCTC TTTTTCCTAT GAAATCTATC AGTACCTTTC TCCATCCGTT GTTCTCAATA TGACCACAGA
010101 GCCTGAGTAT ACCAAGAAAA CCAATATTCG CATTACAGGT TGCTCCTGTC CTTCCAGACA CCTTTCCTGC CTGTGTGACT AACCTAATTT TGCTAGTTCC
010201 ATAAATACAC GATTAGTTTA GTAACAGCCA TCACAATGTA CCATGTACAT TCATGGTGAG AGCTAAAGAT CGACACAGAC TTCTAGGAGC TTTGTTCATA
010301 CTGATTACTT AATCCAATTG TATAGATTGA ATATTTGAGT GGAAGGAATT TACACTCTGT TTAAATGATG GGATTCTATC GAGATAGCAC TCATGATCAT
010401 GACCTTTTTG GTAGTATTCT TAAACAAAAT TCTACAGAGA CTAAATGTTA GCGATGATCC TCCATTTTCA ATTTTAACCA ATTCTGTCCC CTTTCTCAAA
010501 ACCCTGAGCC CTGTGCATGC TTTCTCAGTC TTGTGGTGGG ACTGGATACA ATGACTAACT TCCCCTCCTC CCCTCTTTAA ACACCATTTT CCATGGAGTT
010601 CAAAAAAATT TTTTTTCTTA ACGTTACATA TCATAGTGAA TGGTTTCCCC AGTGTATATG AATGTTTTAA GTGTCTCCAA TAGCTTATGC AGTCTAGGAG
010701 CTTTCCAATA CTCATTTAAT TAAGATTTAA TCATTTGCTA ATGGAAATCT TACCACCTTT CATTTTCCCT CTGTTACCAA ATTTCAGCTC TTAGGAGCTG
010801 CTCTACAATT CTGAATTTGC TTTTCTTGCC TCTCTTTAGT CACCTGTCAC AGGAGGTTCC TGCTCAGTAA TGATATTGTG AGTTAGGATA ATAACTTTTT
010901 TTTTTTGTGC TTCAGATTTA GAAGAAAAGA TCCTGTTTCC ATTTGAAAGG AACTGTAAGC TTTTATCTTT TAACCAACTG AACAATACAC CAAAAGCAGC
011001 CTAGGGATGA GCATTTCTTT GAAAGCAATT AGGTTATTCA CCTGGTATTA AAACTATTTA CTGTTAAAAA ATCTGTGACT TCATGAAGTT GATTTTTAAA
011101 GGCAGCATCA AAAACTGAAA AGGAAGGGAA AAAATAGGCA GCTTCTCTGC ACTTGTTTGG AGCTCCCCAA AACA**GG**AGCC AT**GG**AGAAGT **GG**CATCAAGA
011201 CC**GG**GCTGCC CTTTCGAGAA CACCCTGTGG CAGTTCAGAG ACACGCTTTT CCTACACTGC ATGCAGCCCC TCTTTCCAGC ACTGGAAAGA AGTGGTCTTG
011301 AGCCCAGCTG AGAAGCACTT CACACTCCTC TCTCTTGTTC TGAATGGTGT TTGTGTCAGT CTGCAGCTGT GTATGGTATT ATGTCTTATA ATCCTGCATC
011401 ACTTCTATCC TATCCAGTCA TATCTAATGT AGAAAATTAG TTTCCAGTGA AAGTAATATG TAGTGCTTTT ATGATATTTG TGTGCAATAT CCCCTCTTCC
011501 ATTGAGGATA TTTGATGTAA AGGAAAAAAA AAAACTCAGT TCCACAATAA AATACAAAAG TGGCAAAA

**HUWE1**

000001 GACTGAG**GG**C TAGCGA**GG**GG AGCAG**GG**CTG GAGCA**GG**GCT **GG**AGCA**GG**GC T**GG**AGCA**GG**G CTGAAGCAGG GCTGAAGCAG GGCCGCGGAC CCCGCACGCT
000101 CCTGCGGGCC CCGCGGAGCC ATTGC**GG**CCG A**GG**CCTC**GG**C A**GG**CGCCAGC GGAGAGCTAG CCGCATCTTC GG**GG**GCAGCC C**GG**CAGCTGC C**GG**CGGCGC**G**
000201 **G**CGAGAGAGC **GG**CTGACAGA **GGGG**ATGCGA **GG**TCCTCCAG CAGCCTGACC TGAGTGGGTT AGTGATCCAG AGAAACCAGC AGGCCAACTT **GG**TCA**GG**AA**G**
000301 **G**TTC**GG**GAAG CTGTT**GG**AGC AGTGT**GGGG**A ATTTCCCACC A**GG**ATGAGTA TGATTGGCTG TGATTTTAGA TCGTAAAGCT GAAAATTGAA ATCATGAAAG
000401 TAGACAGGAC TAAACTGAAG AAGACACCTA CTGAGGCTCC TGCAGACTGC AGAGCCTTAA TAGACAAACT CAAAGTTTGT AATGATGAGC AACTTCTCTT
000501 GGAACTGCAG CAGATCAAAA CATGGAACAT TGGAAAGTGC GAGTTATATC ACT**GG**GT**GG**A CCTGTT**GG**AC CGCTTCGAT**G G**AATACTGGC AGATGCTGGA
000601 CAGACAGTGG AGAATATGTC ATGGATGCTC GTATGTGATA GGCCAGAAAG AGAGCAACTG AAAATGCTTC TCTTGGCTGT GTTGAACTTC ACAGCCTTGC
000701 TCATTGAGTA CAGCTTTTCC CGGCATCTGT ACAGTTCCAT AGAGCATTTG ACAACTTTAT TGGCTTCCTC TGATATGCAA GTGGTGCTGG CAGTCCTCAA
000801 TCTCCTATAT GTATTTAGCA AAAGATCAAA CTACATCACT CGTCTGGGAT CTGACAAGAG GACCCCGCTG CTAACTC**GG**C TACAACATTT **GG**CAGAGAGC
000901 T**GGGG**T**GG**AA A**GG**AGAAT**GG** CTTT**GG**ACTT GCAGAATGTT GCAGAGACTT GCATATGATG AAATATCCAC CCAGTGCAAC TACACTACAC TTTGAATTCT
001001 ATGCAGATCC T**GGGG**CCGA**G G**TCAAAATTG AGAAAA**GG**AC AACTAGTAAC ACACTACATT ATATTCACAT AGAGCAACTT GACAAGATTT CAGAAAGCCC
001101 TTCTGAAATC ATGGAATCTC TTACCAAAAT GTACAGCATT CCTAAGGATA AGCAGATGCT GTTATTTACA CACATACGAC TGGCCCATGG CTTTTCTAAT
001201 CACAGGAAGC GATTGCAGGC AGTTCAGGCC AGACTGCATG CAATATCTAT ATTAGTGTAT TCCAATGCCT TGCAGGAATC AGCAAACAGT ATCTTGTATA
001301 ATGGCTTGAT AGAGGAGTTG GTAGATGTCC TTCAGATAAC GGATAAGCAG CTTATGGAGA TTAAAGCAGC TTCTTTACGA ACATTAACAT CAATTGTCCA
001401 CTTGGAGAGA ACTCCCAAAC TCAGCAGTAT TATTGACTGT ACTGGAACTG CCTCCTACCA TGGATTTTTG CCAGTGCTTG TAAGGAACTG TATCCAGGCC
001501 ATGATTGATC CTTCCATGGA TCCATACCCT CACCAGTTTG CCACTGCTCT CTTCTCTTTT TTATACCATC TGGCCAGCTA CGATGCT**GG**T **GG**TGAAGCCT
001601 T**GG**TCTCCTG T**GG**AATGATG GAAGCCTTAT TGAAGGTCAT AAAGTTTCTT GGCGATGAAC AGGACCAGAT AACATTTGTC ACCAGAGCCG TCAGAGTGGT
001701 TGACCTTATC ACCAACCTGG ATATGGCAGC TTTTCAATCC CATAGTGGAC TTTCTATCTT CATTTATAGA CTTGAGCATG AAGTAGATTT GTGCCGAAAA
001801 GAATGTCCGT TTGTGATCAA GCCAAAGATC CAGAGACCCA ATACTACACA AGAA**GG**AGA**G G**AAAT**GG**AAA CTGATAT**GG**A TGGAGTCCAG TGTATTCCAC
001901 AACGAGCAGC ACTTCTGAAA TCCATGTTGA ATTTCCTCAA GAAGGCCATC CAAGACCCTG CTTTCTCAGA TGGCATACGA CATGTGATGG ATGGTTCTCT
002001 GCCTACCTCC CTGAAACACA TCATCAGCAA TGCAGAATAC TATGGCCCAT CACTCTTCCT CCTAGCTACT GAAGTGGTGA CTGTGTTTGT ATTTCAAGAA
002101 CCATCACTGC TCTCCTCACT CCAGGACAAT GGATTGACAG ATGTCATGCT GCATGCACTG CTTATCAAAG ATGTTCCTGC TACCCGTGAA GTCCTTGGCT
002201 CCCTCCCAAA TGTATTCAGT GCACTCTGTT TGAATGCCCG AGGTCTTCAG TCTTTTGTTC AGTGTCAGCC TTTTGAACGC CTCTTCAAAG TTCTTCTGTC
002301 TCCAGATTAC CTCCCAGCCA TGC**GG**A**GG**A**G G**AGAAGTTCT GATCCCCTT**G G**GGATACTGC ATCCAACCTG GGGAGTGCTG TCGATGAGCT CATGAGACAT
002401 CAGCCCACCC TTAAAACAGA TGCAACGACT GCCATCATCA AGTTACTTGA AGAAATCTGT AATCTTGGAA GGGACCCCAA ATACATCTGT CAGAAGCCAT
002501 CAATCCAGAA GGCAGATGGC ACTGCCACTG CTCCTCCCCC AAGGTCTAAT CATGCCGCAG AAGAAGCCTC TAGTGA**GG**AT GA**GG**A**GG**AAG A**GG**AAGTACA
002601 GGCCATGCAG AGCTTTAATT CTACCCAGCA AAATGAAACT GAGCCTAATC AGCAGGTTGT TGGTACAGAG GAACGTATTC CTATTCCCCT CATGGATTAC
002701 ATCCTTAATG TGATGAAATT TGTGGAATCT ATTCTGAGCA ACAATACAAC AGATGACCAC TGCCAGGAAT TTGTGAATCA GAAAGGACTG TTGCCTTTGG
002801 TTACCATTTT GGGTCTTCCC AATCTGCCCA TTGACTTTCC CACATCTGCT GCCTGTCAGG CTGTTGCAGG TGTCTGCAAA TCCATATTGA CACTGTCACA
002901 TGAACCCAAA GTCCTTCAAG AGGGTCTCCT TCAGTTGGAC TCCATCCTCT CCTCCCTGGA GCCCTTACAC CGCCCCATTG AATCCCCTGG GGGCTCAGTG
003001 TTGTTGCGAG AACTGGCTTG CGCAGGCAAT GTTGCTGATG CTACCCTCTC AGCCCAGGCC ACACCTCTGC TGCATGCACT CACTGCTGCC CATGCCTACA
003101 TCATGATGTT TGTTCATACT TGCAGAGTTG GACAGAGTGA AATTCGTTCC ATCTCCGTAA ACCAGTGGGG CTCTCAATTG GGTCTGAGTG TTTTGAGCAA
003201 GCTGAGCCAG TTATACTGTT CCCTGGTGTG GGAAAGCACT GTCCTCCTCT CTCTGTGTAC CCCAAACAGC CTACCATCTG GGTGTGAATT TGGCCAGGCA
003301 GATATGCAGA AACT**GG**TTCC AAA**GG**ATGAG AA**GG**CA**GG**TA CGACCCAGGG CGGAAAAAGA TCAGATGG**GG** AACAGGAT**GG** AGCAGCT**GG**A AGTAT**GG**ATG
003401 CTTCTACCCA GGGCTTATTA GAAGGCATTG **GG**CTAGAT**GG** TGACACATT**G G**CTCCCAT**GG** AGACAGATGA ACCTACTGCT TCAGACTCTA AGGGCAAATC
003501 TAAAATCACA CCAGCAATGG CTGCCAGAAT TAAGCAAATC AAGCCTTTGT TATCAGCTTC CTCCAGATTA GGCCGAGCAC TTGCTGAGCT ATTTGGACTT
003601 CTTGTTAAAC TTTGTGTGGG ATCTCCTGTC CGCCAGAGAA GGAGCCATCA TGCTGCCAGC ACCACTACAG CACCGACACC TGCCGCGCGA TCAACAGCCT
003701 CAGCTCTCAC TAAGCTCTTG ACTAAGGGGT TATCTTGGCA GCCCCCACCA TATACACCTA CTCCCCGATT CAGGCTGACA TTCTTCATCT GTTCAGTTGG
003801 TTTCACATCC CCAATGCTGT TTGATGAGAG GAAGTATCCC TACCACCTCA TGCTGCAAAA ATTTCTCTGC TCCGGAGGCC ACAATGCTCT TTTTGAAACT
003901 TTCAACTGGG CTCTGTCCAT GGGAGGTAAA GTTCCTGTTT CTGAGGGATT GGAACACTCA GACTTGCCTG ATGGCACAGG AGAATTCCTA GATGCCT**GG**C
004001 TTATGCT**GG**T **GG**AGAAGAT**G G**TGAATCCCA CCACGGTGCT TGAATCTCCA CATTCGCTGC CTGCCAAATT GCCTGGAGGT GTCCAGAACT TTCCCCAGTT
004101 CAGTGCACTG CGCTTCCTTG TGGTAACTCA GAAAGCAGCC TTTACTTGCA TCAAAAACTT ATGGAACCGG AAACCCCTGA A**GG**TATAT**GG** T**GG**ACGAAT**G**
004201 **G**CTGAATCGA TGCTGGCCAT TCTATGCCAC ATCCTCCGAG GAGAACCTGT GATTCGAGAG AGACTAAGCA AGGAGAAGGA GG**GG**TCTCGA **GG**AGAAGA**GG**
004301 ATACAG**GG**CA AGA**GG**AA**GG**T **GG**CTCCCGCC **GG**GAACCTCA AGTCAACCAG CAACAACTGC AACAGCTCAT GGACAT**GG**GC TTCACAAG**GG** AACATGCAAT
004401 **GG**A**GG**CACTG TTGAACACCA GCACCATGGA GCAGGCCACA GAGTACCTTT TAACCCACCC TCCTCCAATC ATGGGAGGAG TTGTTCGGGA TCTCAGCATG
004501 TCTGAAGAGG ACCAGATGAT GAGAGCAATT GCTATGTCTC T**GG**GACA**GG**A TATTCCAAT**G G**ATCAAAG**GG** CAGAGTCACC TGAGGAAGTT GCTTGCC**GG**A
004601 AGGA**GG**AAGA **GG**AAC**GG**AAA GCTCG**GG**AAA AGCA**GG**AGGA **GG**AAGA**GG**CT AAATGTCTAG AGAAGTTCCA GGATGCTGAC CCGTTGGAAC AAGATGAGCT
004701 CCACACTTTC ACAGATACTA TGTTGCCAGG CTGCTTCCAC CTTCTTGATG AGCTGCCAGA CACAGTATAC CGTGTGTGTG ACCTGATCAT GACAGCAATC
004801 AAACGTAATG GAGCAGATTA TCGTGACATG ATTCTGAAGC AAGTAGTCAA TCAGGTGTGG GAAGCTGCTG ATGTATTGAT CAAAGCTGCT CTTCCCCTGA
004901 CAACAAGTGA CACAAAAACC GTGTCAGAGT GGATAAGTCA GATGGCCACA CTGCCCCAGG CCTCCAATTT GGCTACTAGA ATCTTGCTTT TAACGCTACT
005001 TTTTGAGGAG TTGAAGCTAC CTTGTGCTTG GGTGGTTGAA TCAAGTGGCA TCCTTAATGT CCTAATCAAA CTCTTGGAAG TGGTTCAGCC CTGCCTCCAG
005101 GCAGCCAAGG AGCAGAAGGA AGTCCAGACC CCAAAGTGGA TCACACCAGT GTTGCTCCTG ATTGATTTCT ATGAAAAGAC AGCCATCTCC TCAAAAAGGA
005201 GAGCCCAGAT GACTAAGTAC CTGCAATCCA ACAGCAACAA CTGGCGCTGG TTTGATGATC GCTCTGGGCG TTGGTGTAGT TACAGTGCAA GCAACAATAG
005301 CACTATTGAT TCTGCCTGGA AATCTGGAGA GACAAGCGTG CGATTCACTG CAGGCCGAAG AAGATACACG GTCCAATTCA CTACAAT**GG**T GCA**GG**TTAAT
005401 GA**GG**AAACAG **GG**AACCGACG CCCTGTGATG CTGACTCTCC TCAGGGTACC TCGGCTGAAT AAAAATTCAA AAAACAGCAA TGGACAGGAA CTAGAGAAGA
005501 CGCTGGAAGA AAGCAAAGAA ATGGATATCA AACGTAAAGA AAATAAAGGC AATGATACCC CTTTGGCCCT AGAGAGTACA AACACTGAAA A**GG**AGACAAG
005601 CCT**GG**A**GG**AA ACAAAAATC**G G**GGAGATCCT GATCCAGGGC TTGACAGAAG ATATGGTGAC TGTTTTAATC CGGGCCTGCG TGAGCATGCT GGGAGTCCCT
005701 GTGGACCCAG ATACTTTGCA TGCCACCCTT CGTCTCTGTC TGAGGCTCAC CCGGGACCAC AAATATGCCA TGATGTTTGC AGAACTGAAG AGTACCCGCA
005801 TGATCTTGAA TTTGACCCAG AGCTCAGGCT TCAATGGGTT TACTCCCCTG GTCACCCTTC TCTTAAGACA CATCATTGAG GACCCCTGTA CCCTTCGTCA
005901 TACCATGGAA AAGGTTGTTC GCTCAGCAGC TACAAGTGGA GCTGGTAGCA CTACCTCT**GG** TGTTGTGTCT **GG**CAGCCTC**G G**CTCTC**GG**GA GATCAACTAC
006001 ATCCTTCGTG TCCTTGGGCC AGCCGCATGC CGCAATCCAG ACATATTCAC AGAAGTGGCC AACTGCTGTA TCCGCATCGC CCTTCCTGCC CCTCGAGGCT
006101 CAGGAACTGC TTCAGATGAT GAATTTGAGA ATCTTAGAAT TAAAGGCCCT AATGCTGTAC AGCTGGTGAA GACCACCCCT TTGAAGCCCT CACCTCTGCC
006201 TGTCATCCCT GATACTATCA AGGAAGTGAT CTATGATATG CTGAATGCTC TGGCTGCATA CCATGCTCCA GAGGAAGCAG ATAAATCTGA TCCTAAACCT
006301 **GGGG**TTATGA CCCAAGA**GG**T T**GG**CCAGCTC CTGCAAGACA TGGGTGATGA TGTATACCAG CAGTACCGGT CACTTACGCG TCAGAGCAGT GACTTTGATA
006401 CGCAGTCAGG TTTTTCCATT AATAGTCAGG TCTTTGCTGC AGATGGTGCC TCCACTGAGA CTTCCGCATC TGGGACCTCC CAAGGAGAGG CTTCAACTCC
006501 AGAGGAGTCT CGAGATGGGA AGAAAGATAA AGAAGG**GG**AC CG**GG**CCTCTG A**GG**AA**GG**CAA ACAGAAAGGC AAGGGCAGCA AACCTTTAAT GCCTACCTCC
006601 ACTATCCTTC GTCTTCT**GG**C AGAGTT**GG**TG A**GG**TCCTATG TT**GG**TATTGC TACCCTGATT GCCAACTACA GCTACACTGT GGGCCAGTCT GAACTGATCA
006701 AAGAGGACTG CAGTGTGCTA GCTTTTGTTC TGGACCACCT GCTCCCACAT ACCCAGAATG CAGAAGACAA GGACACCCCT GCCTTGGCCC GCCTGTTCCT
006801 CGCAAGCCTG GCTGCTGCA**G G**GAGT**GG**CAC AGATGCCCA**G G**T**GG**CCCTAG TGAATGAAGT AAAAGCAGCC CTT**GG**ACG**GG** CACT**GG**CTAT **GG**CTGAGAGT
006901 ACAGAGAAAC ATGCCAGGCT TCAGGCAGTG ATGTGTATCA TCAGTACTAT CATGGAGTCC TGCCCCTCCA CCTCCAGCTT CTACAGCAGT GCCACAGCGA
007001 AGACCCAGCA CAATGGCATG AACAACATCA TTCGGCTTTT CCTGAAGAAG GGACTGGTTA ATGACCTGGC CAGAGTACCT CACAGCTTAG ACCTGTCCAG
007101 TCCCAACATG GCCAACACAG TCAATGCTGC TCTGAAGCCT TTGGAAACAC TTTCCCGGAT TGTGAACCAG CCCAGTAGCC TTTTTGGCAG CAAGAGTGCT
007201 TCTAGCAAGA ACAAGTCTGA GCAGGATGCC CAAGGAGCCT CTCAAGATTC CAGTAGCAAC CAGCA**GG**ACC CA**GG**CGAGCC T**GGGG**AAGCA GAAGTGCAGG
007301 AGGAGGATCA TGATGTCACT CAGACAGAGG T**GG**CAGATGG **GG**ATATCAT**G G**ATGGGGA**GG** CTGAAACCGA CTCAGTGGTG ATTGCTGGGC AGCCTGAGGT
007401 GCTCAGTTCA CAAGAGATGC AGGTTGAGAA TGAGCTGGAG GACCTGATAG ATGAGTTGCT TGAGAG**GG**AT **GG**C**GG**ATCT**G G**GAACAGTAC AATTATAGTG
007501 AGCAGAAGTG GAGAGGATGA ATCACAAGAG GACGTGCTGA TGGATGAAGC TCCTTCCAAC CTCAGCCAAG CTTCCACCTT GCAGGCCAAC CGAGAAGATT
007601 CCATGAATAT CCT**GG**ACCCT GA**GG**ATGA**GG** A**GG**AGCACAC TCA**GG**AAGA**G G**ACAGCAGT**G G**CAGTAACGA **GG**ATGAGGAT GATAGTCAGG ATGAAGA**GG**A
007701 **GG**A**GG**A**GG**AG GAAGATGAGG AAGATGATCA **GG**A**GG**ATGAT GAA**GG**TGAAG A**GG**GAGATGA AGACGATGAC GACGAT**GG**CT CTGAGAT**GG**A ATT**GG**ATGA**G**
007801 **G**ATTATCCTG ATATGAACGC TTCTCCCTTG GTCCGATTTG AGCGCTTTGA CCGGGAGGAT GATCTCATCA TTGAGTTTGA CAACATGTTC TCCAGTGCTA
007901 CAGACATCCC CCCATCCCCA GGAAATATCC CTACCACCCA TCCACTGATG GTGCGCCATG CAGACCACAG TTCTCTGACA CTGGGCAGTG GCTCTTCAAC
008001 AACTCGTCTC ACCCAG**GG**CA TCG**GG**CGCAG TCAGA**GG**ACC CTAA**GG**CAGC TGACGGCCAA TACTGGCCAC ACCATTCATG TTCACTACCC TGGGAATCGC
008101 CAGCCCAACC CTCCTCTTAT ACTGCAGAGG TTGCTTGGTC CCTCAGCTGC TGCTGACATC CTTCAGCTGA GCAGCAGCCT TCCCCTACAA AGCCGG**GG**TC
008201 G**GG**CCCGCCT CCT**GG**TA**GG**C AACGATGACG TCCACATCAT CGCCCGTTCT GATGATGAGC TGCTGGATGA CTTTTTCCAT GATCAGAGCA CAGCTACCAG
008301 CCAAGCAGGA ACCCTGTCCA GCATCCCCAC AGCCCTGACC CGCTGGACAG AAGAATGCAA AGTTCTCGAT GCTGAGAGCA TGCATGACTG TGTTTCAGTG
008401 GTTAAAGTGT CCATTGTCAA TCACCTGGAA TTCCTGAG**GG** ATGA**GG**AGCT **GG**AAGAAA**GG** CGAGAGAAGC GCAGGAAACA ACTGGCTGAG GAAGAAACAA
008501 AGATAACTGA TAAAGGCAAA GAAGATAAGG AGAACAGGGA TCAGAGTGCA CAGTGTACTG CATCTAAGTC AAATGACTCC ACTGAACAGA ATCTCTCAGA
008601 TGGGACGCCT ATGCCTGACA GCTACCCAAC AACCCCATCT TCAACTGATG CAGCTACATC TGAGTCCAAG GAGACCCTTG GCACTCTGCA ATCCTCACAA
008701 CAGCAACCAA CACTCCCAAC CCCACCAGCT TTGGGAGAGG TTCCTCA**GG**A GCTGCAGTCT CCAGCT**GG**AG AA**GG**G**GG**CAG CTCTACACAG CTATTGATGC
008801 CTGTAGAGCC AGA**GG**AATTG **GG**TCCCACAA **GG**CCAAGT**GG** GGAAGCAGAA ACAACTCAGA TGGAGTTATC CCCAGCTCCC ACTATAACCT CACTTTCCCC
008901 AGAGAGAGCT GAGGATTCTG ATGCACTGAC GGCTGTCAGC AGTCAGCTAG AAGGCTCTCC TATGGATACA AGCAGCCT**GG** CTTCCTGTAC CTTAGA**GG**A**G**
009001 **G**CTGT**GG**GTG ACACTTCAGC AGCTGGCAGT TCTGAGCAGC CCAGAGCAGG CAGCTCCACT CCT**GGGG**ATG CCCCACCAGC TGT**GG**C**GG**AA GTGCAA**GG**CA
009101 **GG**AGTGAT**GG** GTCAGG**GG**AA TCTGCCCAGC CACCTGAGGA CAGCTCCCCA CCTGCATCCT CTGAGAGCTC TTCCACCAGA GATTCTGCCG TGGCCATTTC
009201 TGGAGCAGAT TCCCGAGGAA TCCTAGAAGA GCCGTTGCCT TCAACAAGCA GTGAAGAAGA AGATCCCCTT GCGGGTATCA GTCTCCCTGA AGGTGTGGAC
009301 CCCTCTTTTC TGGCTGCCCT GCCTGATGAC ATCCGTCGGG AAGTTCTACA GAACCAGCTA GGCATTCGTC CACCAACCCG GACTGCCCCC TCCACAAATA
009401 GCTCAGCGCC TGCAGT**GG**T**G GGG**AATCCT**G G**TGTGACTGA AGTGAGCCCT GAGTTTCTGG CTGCCCTGCC TCCAGCCATT CAGGAGGAAG TACTGGCACA
009501 GCAGAGAGCT GAGCAGCAGC GACGAGAACT AGCACAGAAT GCCAGCTCAG ACACCCCTAT GGACCCTGTG ACCTTCATCC AGACTCTGCC CTCAGACCTG
009601 CGCCGTAGTG TCCTAGAGGA TATGGAGGAC AGTGTGTTAG CTGTGATGCC ACCTGACATT GCAGCTGAGG CTCAAGCCCT GAGACGAGAG CAAGAAGCCC
009701 GGCAGCGACA GCTCATGCAT GAGCGTCTGT TTGGGCACAG TAGCACCTCC GCACTCTCTG CTATTCTCCG AAGCCCGGCT TTCACCAGTC GCTTAAGTGG
009801 CAACCGTGGG GTCCAGTATA CTCGCCTTGC TGTGCAGAGA **GG**T**GG**CACCT TCCAGAT**GG**G **GG**GTAGCAGC AGCCATAACA GGCCTTCTGG CAGTAATGTA
009901 GATACTCTCC TCCGCCTCCG AGGACGGCTC CTTCTGGACC ACGAAGCCCT TTCTTGTCTC TTGGTCCTAC TTTTTGTGGA TGAGCCAAAG CTCAATACTA
010001 GCCGTCTACA CCGAGTACTG AGAAATCTCT GCTACCATGC CCAGACCCGC CACTGGGTCA TCCGCAGTCT GCTCTCCATC TTGCAGCGCA GCAGTGAGAG
010101 TGAGCTATGC ATTGAAACAC CCAAACTCAC TACAAGTGAG GAAAAGGGCA AAAAGTCGAG CAAGAGCTGT GGGTCAAGTA GCCATGAGAA CCGTCCCCTG
010201 GACCTGCTAC ACAAGATGGA GTCAAAGAGC TCCAACCAGC TTTCCTGGCT CTCAGTATCC ATGGATGCAG CCCTAGGCTG CAGGACTAAT ATATTTCAGA
010301 TCCAGCGTTC AGGGGGGCGT AAACATACCG AGAAGCATGC AAGCGGTGGC TCCACCGTCC ACATCCATCC CCAAGCTGCT CCTGTTGTCT GCAGACACGT
010401 TTTGGATACA CTCATTCAAT TGGCCAAGGT ATTTCCCAGC CACTTCACAC AGCAGCGGAC CAAAGAAACA AACTGTGAGA GTGATC**GG**GA AA**GGGG**CAAT
010501 AA**GG**CCTGTA GCCCATGCTC CTCACAGTCC TCCAGCAGTG GCATTTGCAC AGACTTCTGG GACTTATTGG TAAAACTGGA CAACATGAAT GTCAGCCGGA
010601 AAGGCAAGAA CTCCGTGAAG TCAGTGCCAG TGAGCGCT**GG** C**GG**TGA**GG**G**G G**AAACCTCTC CATACAGCCT CGAGGCCTCT CCACTGGGGC AGCTCATGAA
010701 CATGTTGTCA CACCCAGTCA TCCGCCGGAG CTCTCTCTTA ACTGAGAAAC TCCTCAGACT CCTTTCTCTC ATCTCAATTG CTCTCCCAGA AAACAAGGTG
010801 TCAGAAGCAC AGGCTAATTC TGGCAGCGGT GCTTCCTCCA CCACCACTGC CACCTCAACC ACATCTACCA CCACCACCAC TGCCGCCTCC ACCACGCCCA
010901 CACCCCCTAC TGCACCCACC CCTGTCACTT CTGCTCCAGC CCTGGTTGCT GCCACGGCTA TTTCCACCAT TGTCGTAGCT GCTTCGACCA CAGTGACTAC
011001 CCCCACGACT GCTACCACTA CTGTTTCAAT TTCTCCCACT ACTAAGGGCA GCAAATCTCC AGCGAAGGTG AGTGATGGGG GCAGCAGCAG TACAGACTTT
011101 AAGATGGTGT CCTCTGGCCT CACTGAAAAC CAGCTACAGC TCTCTGTAGA GGTGTTGACA TCCCACTCTT GTTCTGAGGA AGGCTTAGAG GATGCAGCCA
011201 ACGTACTACT GCAGCTCTCC C**GGGGGG**ACT CT**GGG**ACCC**G GG**ACACTGTT CTCAAGCTGC TACTGAATGG AGCCCGCCAT CTGGGTTATA CCCTTTGTAA
011301 ACAAATAGGT ACCCTGCTGG CCGAGCTGCG GGAATACAAC CTCGAGCAGC AGCGGCGAGC CCAATGTGAA ACCCTCTCTC CTGATGGCCT GCCTGAGGAG
011401 CAGCCACAGA CCACCAAGCT GAAGGGCAAA ATGCAGAGCA GGTTTGACAT GGCTGAGAAT GTGGTAATTG TGGCATCTCA GAAGCGACCT TTGGGTGGCC
011501 GGGAGCTCCA GCTGCCTTCT ATGTCCATGT TGACATCCAA GACATCTACC CAGAAGTTCT TCTTGAGGGT ACTACAGGTC ATCATCCAGC TCCGGGACGA
011601 CACGCGCCGG GCTAACAAGA AAGCCAAGCA GACA**GG**CAGG CTA**GG**TTCCT CC**GG**TTTA**GG** CTCAGCTAGC AGCATCCAGG CAGCTGTTC**G G**CAGCT**GG**A**G**
011701 **G**CTGA**GG**CTG ATGCCATTAT ACAAAT**GG**TA CGTGA**GG**GTC AAA**GG**GCGC**G G**AGACAGCAA CAAGCAGCAA CGTCGGAGTC TAGCCAGTCA GAGGCGTCTG
011801 TCC**GG**AGGGA **GG**AATCACCC AT**GG**ATGT**GG** ACCAGCCATC TCCCAGTGCT CAAGATACTC AATCCATTGC CTCCGATGGA ACCCCACA**GG** G**GG**AGAA**GG**A
011901 AAA**GG**AAGAA AGACCACCTG AGTTACCCCT GCTCAGCGAG CAGCTGAGTT T**GG**ACGAGCT GT**GG**GACATG CTT**GGGG**AGT GTCTAAAGGA ACTAGAGGAA
012001 TCCCATGACC AGCATGCGGT GCTAGTGCTA CAGCCTGCTG TCGAGGCCTT CTTTCTGGTC CATGCCACAG AGCGGGAGAG CAAGCCTCCT GTCCGAGACA
012101 CCCGTGAGAG CCAGCTGGCA CACATCAAGG ACGAGCCTCC TCCACTCTCC CCTGCCCCCT TAACCCCAGC CACGCCTTCC TCCCTTGACC CATTCTTCTC
012201 CCGGGAGCCC TCATCTATGC ACATCTCCTC AAGCCTGCCC CCTGACACAC AGAAGTTCCT TCGCTTTGCA GAGACTCACC GCACTGTGTT AAACCAGATC
012301 CTACGGCAGT CCACGACCCA CCTTGCTGAT GGGCCTTTTG CTGTCCTGGT AGACTACATT CGTGTCCTCG ACTTTGATGT CAAGCGCAAA TATTTCCGCC
012401 AAGAGCTGGA GCGTTTAGAT GA**GGGG**CTCC **GG**AAAGAAGA CAT**GG**CTGTG CATGTCCGTC GTGACCATGT GTTTGAAGAC TCCTATCGTG AGCTGCATCG
012501 CAAATCCCCC GAAGAAATGA AGAATCGATT GTATATAGTA TTTGAAGGAG AAGAA**GG**GCA **GG**ATGCT**GG**T G**GG**CTCCTGC GGGAGTGGTA TATGATCATC
012601 TCTCGAGAGA TGTTTAACCC TATGTATGCC TTGTTCCGTA CCTCACCTGG TGATCGAGTC ACCTACACCA TCAATCCATC TTCCCACTGC AACCCCAACC
012701 ACCTCAGCTA CTTCAAGTTT GTCGGACGCA TTGTGGCCAA AGCTGTATAT GACAACCGTC TTCTGGAGTG CTACTTTACT CGATCCTTTT ACAAACACAT
012801 CTTGGGCAAG TCAGTCAGAT ATACAGATAT GGAGAGTGAA GATTACCACT TCTACCAAGG TCTGGTTTAT CTGCTGGAAA ATGATGTCTC CACACTAGGC
012901 TATGACCTCA CCTTCAGCAC TGAGGTCCAA GAGTTTGGAG TTTGTGAAGT TCGTGACCTC AAACCCAAT**G GGG**CCAACAT CTT**GG**TAACA GA**GG**AGAATA
013001 AGAAGGAGTA TGTACACCTG GTATGCCAGA TGAGAATGAC AGGAGCCATC CGCAAGCAGT TGGCGGCTTT CTTAGAAGGC TTCTATGAGA TCATTCCAAA
013101 GCGCCTCATT TCCATCTTCA CTGAGCAGGA GTTAGAGCTG CTTATATCAG GACTGCCCAC CATTGACATC GATGATCTGA AATCCAACAC TGAATACCAC
013201 AAGTACCAGT CCAACTCTAT TCAGATCCAG TGGTTCTGGA GAGCATTGCG TTCTTTCGAT CAAGCTGACC GTGCCAAGTT CCTCCAGTTT GTCACAGGTA
013301 CTTCCAAGGT ACCCCTGCAA GGCTTTGCTG CCCTCGAAGG CATGAATGGC ATTCAGAAGT TTCAGATCCA TCGAGATGAC AGGTCCACAG ATCGCCTGCC
013401 TTCAGCTCAC ACATGTTTTA ATCAGCTGGA TCTGCCTGCC TATGAGAGCT TTGAGAAGCT CCGCCACATG CTACTGTTGG CTATCCAGGA GTGCTCTGAA
013501 **GG**CTTT**GG**GC T**GG**CCTAATA A**GG**CCCTGCC CAACTCCGTG GGGTTTTTTT TACCATTGTT GGACCT**GGGG** A**GG**G**GG**GAGT TAAAAAAAGA ACCAGAAAGA
013601 AATTGTCAAA AACCAATAAA TGAAATCCAC CAACTCACCG TGTGTGTCCC AGCTGCCCCA TCTTCCCCAG CGCATACCTG TTCCTCTTCT CATTCTCTCC
013701 CCGCCGCCTG TTTCCTCACC TTCTCTCCCC TTTCCATGCC GTCCATGATC CCCACCCCAT GTGTTTTAAA AAGGCAGTAG CCTTTGCAGG GACCTGTCTG
013801 TCCCAACTGT TTGAACAGTG TGCTCCTCAG ATTCTGTGTT CAGAAGGATT TGCTGCATTG AGACTTGAAA CCTTTGGATA GGGGAAAAAA TTATATATAT
013901 ATATATTTTT TTGTTCTGTT TGCATTTCTT AATTTGTGCT TGGAATGTGT TGATGTGCAC AGCTAATGAT TCAATGCGAG ACAAGATTGG CGTCTGTGTT
014001 GTGGAGGTTT CAAATAAAGA GCACTCTTCA TAACTCACTT TTCACAATGG AGTTTTTTTC AAACTTAAAA AAAAAAACAA AAAACTCTTA AGCACATGCT
014101 A**GG**CATCT**GG** AGAAAAGAT**G G**ATTCTCCA**G G**AAACCATCC CTGCCCTACC CGCTTGCCCA CCTGCCTCCA CCACTGGTGC AACTTCCTCC TTCAGAGTCA
014201 GTTTCTGCAG TCAGGAGAGA TGAGCAGTTC ACCAGGAATT **GG**G**GG**T**GG**G**G G**CATCTGTTT TTTTTTTTTA GAATGGGAGC TGCACTT**GGG G**AT**GG**TGAT**G**
014301 **G**TGTTAGAGA TTGCAGCCCA GGACAAAGCA TCACCTTGCT CAAGGGGAAC AAACTGCAGC AGGTTGTACA AAACATTCAG AGAGCTCACT TGACCCAACC
014401 GAGGGTTTTA CTTGGTGAGC CTTTCTAGTA GTCTTGAGTC TGGGGCTCAG TTTTAGATTT TTATTTCATT AATGTTTGTT TATTTCTAAT AAATTTTAAT
014501 AAGCGAGTAA TTTAACTAGG TCGCCAGAAG ACCATTATTT TTGTTTGTCT ATTTTTGTTG TCGTCACTCC CTCTCTCGTC ATCTGTCTGC CAGTCTGTTC
014601 ACAGATCACC TTGTAGTCCC TTGTGGTTTT TACTCCATCC AGTGCTTAAA GCTGATCTAA GGACTTCGAT GGTTCCAGCC TTCAAAGAAA AAATAATTTA
014701 ATAAAAATTT AGTTAGAAAA AATAACCCCA A

**UBR4**

000001 GCAGTAGTAC GAC**GG**AAGAT **GG**CGACGAGC **GG**CGGCGAAG A**GG**CGGC**GG**C AGC**GG**CTCC**G G**CGCC**GGGG**A CCCC**GG**CAAC **GG**GGGC**GG**AC ACGACCCCG**G**
000101 **G**CT**GG**GA**GG**T **GG**CTGTGCGG CCCCTGCTGT CCGCGTCCTA CTCCGCCTTC GAGATGAAGG AGTTGCCGCA GCTGGTGGCC TCAGTCATCG AGAGTGAATC
000201 AGAAATCCTG CACCATGAGA AGCAGTACGA GCCATTCTAC TCATCTTTTG TTGCACTTTC CACACACTAT ATTACAACAG TTTGCAGTCT CATTCCCCGG
000301 AACCAACTTC AGTCAGTGGC AGCAGCCTGT AAAGTTCTAA TTGAGTTTTC TCTCCTGCGT CTGGAGAATC CAGATGAGGC TTGTGCTGTG TCCCAGAAAC
000401 ACTTGATTCT CCTAATCAAG GGCCTGTGCA CTGGCTGTAG CCGACTAGAT AGAACTGAAA TTATCACATT TACAGCAATG ATGAAATCCG CCAAGCTGCC
000501 CCAAACAGTG AAGACACTTT CAGACGTGGA AGATCAGAAA GAGCTGGCCT CACCAGTAAG CCCTGAGTTG AGGCAAAAGG AGGTACAGAT GAATTTTTTG
000601 AACCAGCTGA CCTCAGTTTT TAACCCTAGA ACTGTAGCAT CACAACCTAT CAGTACACAG ACTCTGGTGG AAGGAGAAAA TGATGAGCAG TCATCTACAG
000701 ATCAAGCCTC AGCTATCAAA ACCAAGAATG TGTTCATAGC TCAGAACGT**G G**CTAGTCTTC AAGAGCTT**GG** T**GG**CTC**GG**AG AAGCTACTGC GTGTATGTTT
000801 GAACCTGCCA TATTTCCTAC GCTATATCAA TCGGTTCCAA GATGCAGTTT TAGCTAATTC CTTCTTCATA ATGCCTGCAA CAGTAGCAGA TGCCACTGCT
000901 GTTCGTAATG GCTTTCATTC ATTGGTGATT GATGTAACTA TGGCATTGGA TACCCTTTCT CTACCTGTGT TGGAACCTCT CAATCCTTCT CGTCTACAAG
001001 ATGTGACAGT CCTCAGCCTA AGTTGTCTGT ATGCAGGTGT GAGTGTGGCA ACGTGCATGG CCATCCTCCA TGTGGGTAGT GCCCAGCAAG TGCGGACAGG
001101 GTCCACGAGC TCCAAAGAAG ATGACTATGA AAGTGACGCA GCTACAATTG TCCAGAAATG TCTCGAAATC TATGACATGA TTGGACAAGC AATCAGCAGT
001201 TCTCGCCGGG CTGGTGGTGA GCACTATCAG AATTTCCAAT TGCTGGGTGC TTGGTGCTTG TTAAACAGCC TTTTCCTCAT ACTGAACCTC AGTCCTACTG
001301 CGTT**GG**CTGA TAA**GGGG**AAA GAGAA**GG**ACC CACTGGCTGC CCTCCGAGTC AGAGACATCC TTTCTCGTAC TAAAGAGGGA GTGGGCTCCC CTAAACTG**GG**
001401 **G**CCTGGAAAA **GGG**CATCA**GG G**ATTT**GGG**GT ACTCTCAGTA ATATTGGCAA ACCATGCCAT CAAACTGCTA ACGTCTCTCT TTCAAGACCT ACAAGTGGA**G**
001501 **G**CCCTTCACA A**GG**GTTG**GG**A GACAGAT**GG**C CCCCCTGCAG CCTTGAGCAT TATGGCCCAG AGCACCTCCA TACAGAGGAT TCAACGGCTG ATTGACTCTG
001601 TCCCACTGAT GAACCTGCTC TTGACGTTAC TTTCAACTTC CTACAGAAA**G G**CATGTGTCC TGCAGC**GG**CA GA**GG**AAG**GG**C TCCATGAGCA GCGATGCCAG
001701 CGCCTCCACC GACTCCAATA CTTACTATGA **GG**ACGATTTC AGTAGCAC**GG** A**GG**A**GG**ACAG CAGCCAAGAC GATGACAGTG AGCCTATTTT **GGGG**CAAT**GG**
001801 TTTGA**GG**AGA CTATTTCTCC CAGTAAAGAG AAAGCAGCAC CTCCGCCTCC TCCCCCACCT CCTCCACTGG AAAGCTCTCC TCGGGTTAAA AGCCCCAGTA
001901 AGCA**GG**CCCC T**GG**TGAGAAG **GG**CAACATTC T**GG**CGAGTCG CAAAGATCCT GAGTTGTTCT TAGGTCTGGC TTCCAACATT TTGAACTTCA TCACCTCTTC
002001 CATGCTGAAC TCTCGGAACA ATTTTATCCG AAACTATCTG AGTGTATCTC TTTCAGAACA CCATATGGCC ACCCTAGCCA GTATCATCAA **GG**AGGT**GG**AC
002101 AAAGAT**GG**AC TCAAG**GG**TTC ATCAGATGAA GAGTTTGCTG CAGCTCTCTA TCACTTCAAC CACTCACTGG TAACCTCTGA CCTTCAGTCA CCTAACCTGC
002201 AGAACACACT GTTGCAGCAG CTA**GG**AGT**GG** CTCCTTTTTC TGA**GG**GCCCT T**GG**CCCTTGT ACATTCACCC TCAAAGCCTC TCTGTGCTTT CACGCCTCCT
002301 GCTCATCTGG CAACATAAAG CCAGTGCTCA AGGTGACCCT GACGTCCCAG AATGCCTTAA AGTTTGGGAC AGGTTTTTGT CTACAATGAA GCAGAATGCC
002401 CTGCAAGGTG TGGTGCCCAG TGAGACAGAG GATCTGAATG TAGAACACCT GCAGATGCTC CTCCTCATTT TCCACAATTT CACCGAGACA GGCCGGCGGG
002501 CCATATTGTC GCTTTTTGTC CAGATCATCC AGGAGTTGAG CGTCAACATG GATGCTCAGA TGCGCTTCGT GCCGCTTATC TTGGCTCGCC TCCTTCTCAT
002601 CTTTGATTAT CTGCTTCATC AGTACTCCAA AGCCCCTGTG TATCTATTTG AGCAGGTACA GCATAACCTG CTAAGTCCTC CCTTT**GG**GTG **GG**CAAGT**GG**A
002701 TCCCA**GG**ACA GCAACAGCCG CCGGGCAACC ACTCCTCTCT ATCATGGATT CAAAGAAGTA GAAGAAAACT GGTCTAAGCA TTTCTCATCA GATGCTGTCC
002801 CACACCCCAG ATTCTACTGT GTCCTGTCCC CAGAAGCCTC AGAGGATGAT TTGAACCGAC TTGATTCTGT GGCATGTGAC GTCCTTTTCT CCAAGCTTGT
002901 CAAGTATGAT GAGCTTTATG CTGCACTGAC AGCCCTGCTT GCAGCTGGGT CCCAGCTTGA TACAGTTAGG AGAAAGGAAA ACAAGAATGT AACAGCCTTG
003001 GAGGCCTGTG CCCTTCAATA TTACTTCTTG ATACTGTGGA GGATCCTAGG AATTTTACCA CCATCAAAGA CTTACATTAA CCAGCTATCC ATGAACTCAC
003101 CTGAGATGAG CGAATGTGAC ATCTTGCACA CTCTGCGATG GTCTTCTCGG CTCCGGATCA GCTCCTATGT CAACTGGATA AAGGATCACC TTATCAAACA
003201 GGGAATGAAG GCTGAGCATG CTAGCTCGCT TCTAGAACTG GCATCCACCA CTAAGTGTAG CTCAGTGAAA TATGATGTTG AAATAGTAGA GGAATACTTC
003301 GCTCGACAGA TCTCATCCTT CTGTAGTATC GACTGTACCA CCATCTTGCA GCTGCATGAA ATTCCCAGTC TGCAGTCCAT CTACACCCTT GATGCCGCGA
003401 TCTCAAAGGT CCAGGTCTCT TTGGATGAGC ATTTTTCTAA GATGGCTGCT GAGACTGATC CTCATAAGTC GTCTGAGATT ACCAAGAACC TACTTCCAGC
003501 CACGCTGCAA CTCATTGACA CCTATGCATC GTTCACCAGA GCCTATTTGC TGCAAAACTT TAATGAAGAG GGAACAACTG AGAAACCTTC CAAGGAGAAA
003601 CTGCAAGGCT TTGCTGCTGT TTT**GG**CTATT **GG**CTCTAGCA **GG**TGCAA**GG**C AAATACTCTG GGTCCGACAC TGGTTCAGAA TTTGCCATCG TCAGTGCAGA
003701 CTGTGTGTGA GTCCTGGAAC AACATCAATA CCAATGAATT TCCCAATATT GGATCCTGGC GCAATGCCTT TGCCAATGAC ACCATCCCTT CAGAGAGTTA
003801 TATTAGTGCA GTGCAGGCTG CACACCTGGG GACTCTCTGT AGCCAAAGTC TGCCCCTGGC TGCTTCCCTG AAGCATACCC TCCTCTCACT GGTCAGGTTG
003901 ACTGGAGATC TTATTGTTTG GTCAGATGAG ATGAACCCAC CACAGGTAAT TCGGACACTG CTACCTCTTC TTTTGGAATC AAGCACTGAG AGTGTTGCCG
004001 AGATCAGTAG CAACTCCCTG GAACGCATCT TGGGCCCTGC TGAGTCTGAT GAGTTCTTGG CTCGTGTTTA TGAGAAGCTG ATCACTGGTT GTTACAACAT
004101 TCTGGCCAAT CATGCAGATC CTAACAGT**GG** ACT**GG**ATGAA TCCATCCT**GG** A**GG**AATGTCT CCAGTACTTG GAAAAGCAGC TGGAAAGTAG CCAGGCTCGT
004201 AAAGCTATGG AGGAGTTTTT CTCTGACAGT GGAGAACTTG TACAGATCAT GATGGCAACA GCCAATGAGA ACCTCTCTGC TAAATTCTGT AACCGAGTTT
004301 TGAAATTCTT CACCAAACTC TTCCAGCTGA CTGAGAAGAG CCCTAACCCG AGCCTGTTGC ATCTCTGT**GG** CTCCCT**GG**CA CAACT**GG**CCT GTGT**GG**AACC
004401 TGTGCGCCTG CAGGCCTGGC TCACCCGCAT GACTACATCG CCCCCAAAAG ATTCTGATCA GCTGGATGTA ATTCAGGAGA ACCGGCAGCT GCTGCAGTTA
004501 CTGACCACAT ACATTGTTCG **GG**AAAACAGC CAAGTT**GGGG** AA**GG**TGTGTG TGCTGTTCTT CTGGGCACCC TGACTCCCAT GGCAACAGAG ATGCT**GG**CCA
004601 AC**GG**TGAT**GG** GACT**GG**CTTC CCTGAACTTA T**GG**TTGTGAT **GG**CCACTCT**G G**CCAGTGCA**G G**TCAAGGTGC TGGTCACCTT CAGCTTCATA ATGCTGCTGT
004701 GGATTGGCTG AGCAGATGCA AGAAATACCT GTCACAGAAG AATGTAGTTG AAAAACTGAA TGCCAATGTA ATGCATGGAA AGCATGTGAT GATCTTGGAG
004801 TGCACATGCC ATATCATGTC TTACTTGGCT GATGTCACGA ATGCCCTGAG CCAGAGTAAT GGTCAAGGCC CAAGTCATCT CTCAGT**GG**AT **GGGG**AAGAGC
004901 **GG**GCCATTGA AGTAGACTCA GACTG**GG**TGG A**GG**AGTT**GG**C GGT**GG**AAGAG GAAGATTCCC AGGCTGAGGA TTCAGATGAA GATTCTCTTT GCAATAAACT
005001 CTGCACTTTT ACGATCACAC AGAAAGAATT CATGAACCAG CATTGGTACC ACTGTCACAC CTGTAAAAT**G G**T**GG**AT**GG**CG T**GG**GTGTCTG CACAGTGTGT
005101 GCTAAGGTGT GCCACAAGGA TCATGAGATT TCCTATGCCA AGTATGGATC CTTCTTCTGT GACTGT**GG**AG CCAA**GG**AAGA T**GG**CAGCTGT TT**GG**CTCTGG
005201 TGAAGAGAAC TCCTAGCAGT GGCATGAGCT CTACCATGAA GGAGTCGGCA TTTCAGAGTG AACCCAGGAT TTCAGAGAGT CTAGTGCGTC ATGCCAGCAC
005301 CTCCTCGCCA GCTGACAAAG CCAAGGTTAC CATCAGTGAT GGAAAGGTTG CTGACGAAGA GAAGCCCAAG AAGAGCAGCC TCTGCCGCAC AGTAGA**GG**GC
005401 TGCC**GG**GA**GG** AATTACAGAA CCA**GG**CCAAT TTCTCCTTCG CTCCTCTCGT GTTAGACATG CTTAATTTCC TTATGGATGC CATTCAGACC AACTTCCAGC
005501 AAGCTTCAGC CGTCGGGAGC AGCAGCCGTG CTCAGCAAGC CCTCAGTGAG CTACACACTG TGGAGAAGGC AGTGGAGATG ACAGACCAGC TGAT**GG**TTCC
005601 CACCTTA**GG**G TCCCA**GG**AA**G G**TGCCTTTGA GAATGTGCGG ATGAATTACA GTGGAGACCA GGGCCAGACC ATCCGGCAGC TGATCAGTGC TCATGTGCTC
005701 AGGCGGGTGG CTATGTGTGT GCTCTCCTCT CCCCATGGGC GCCGCCAACA TTTGGCTGTC AGCCATGAGA AGGGCAAGAT CACCGTTCTG CAGCTCTCTG
005801 CACTCCTGAA GCAAGCAGAT TCCAGCAAAA GGAAGTTAAC TCTGACCCGC TTGGCTTCTG CCCCAGTTCC TTTTACTGTG TTGAGCCTCA CAGGAAATCC
005901 CTGCAAGGAA GACTACTT**GG** C**GG**TTTGT**GG** GCTAAA**GG**AC TGTCATGTGC TCACCTTTAG TAGCTCAGGC TCTGTTTCGG ATCACTT**GG**T TTTGCACCCT
006001 CAGTT**GG**CAA C**GGGG**AACTT CATCATCAAA GCCGTGTGGT TACCTGGTTC ACAGACCGAG TTAGCAATTG TCACCGCAGA CTTTGTTAAG ATTTATGACC
006101 TGTGTGTTGA TGCCTTGAGT CCAACCTTCT ATTTTCTCCT GCCAAGCTCA AAGATAAGAG ATGTTACCTT CCTTTTCAAT GAGGAGGGAA AGAACATCAT
006201 TGTTATAATG TCTTCGGCTG GGTACATCTA TACTCAGCTT ATGGAAGAGG CCAGCAGTGC CCAGCAGGGA CCCTTCTATG TCACTAATGT GTTGGAAATC
006301 AATCATGAGG ACCTGAAGGA CAGTAACAGC CAGGT**GG**CG**G G**C**GG**T**GG**TGT GTCCGTGTAC TACTCCCACG TGTTGCAGAT GTTGTTCTTC AGCTATTGTC
006401 AAGGCAAATC ATTCGCAGCC ACCATCAGCA GGACAACCCT GGAGGTGTTG CAACTCTTCC CCATCAACAT CAAAAGTTCC AATGGTGGCA GTAAGACTTC
006501 TCCTGCTCTT TGCCAGT**GG**T CTGA**GG**TGAT GAACCACCCT **GG**CTT**GG**TGT GCTGTGTCCA GCAAACTACA **GGGG**TGCCGC T**GG**TAGTTAT **GG**TGAAACCA
006601 GACACTTTTC TTATCCAGGA GATTAAGACT CTTCCTGCTA AAGCGAAGAT CCAAGACATG GTTGCTATTA GGCACACGGC CTGCAATGAG CAGCAGCGGA
006701 CAACAATGAT TCTGCTGTGT GAGGATGGCA GCCTGCGCAT TTACATGGCC AACGTGGAGA ACACCTCCTA CTGGCTGCAG CCATCCCTGC AGCCCAGCAG
006801 TGTCATCAGC ATCATGAAGC CTGTTCGAAA GCGCAAAACA GCTACAATCA CAACCCGCAC GTCTAGCCAG GTGACTTTCC CCATTGACTT TTTTGAACAC
006901 AACCAGCAGC TGACAGATGT **GG**AGTTT**GG**T **GG**TAACGACC TCCTACA**GG**T CTATAATGCA CAACAGATAA AACACCGGCT GAATTCCACT GGCATGTATG
007001 TGGCCAACAC CAAGCCCGGA GGCTTCACCA TTGAGATTAG TAACAACAAT AGCACTAT**GG** TGATGACA**GG** CATGC**GG**ATC CAGATT**GG**GA CTCAAGCAAT
007101 AGAACGGGCC CCGTCATATA TCGAGATCTT CGGCAGAACT ATGCAGCTCA ACCTGAGTCG CTCACGCTGG TTTGACTTCC CCTTCACCAG AGAAGAAGCC
007201 CTGCAGGCTG ATAAGAAGCT GAACCTCTTC ATT**GGGG**CCT C**GG**T**GG**ATCC AGCAGGTGTC ACCATGATAG ATGCTGTAAA AATTTAT**GG**C AAGACTAA**GG**
007301 AGCAGTTT**GG** CT**GG**CCTGAT GAGCCCCCAG AAGAATTCCC TTCTGCCTCT GTCAGCAACA TCTGCCCTTC AAATCTGAAC CAGAGCAACG GCACTGGAGA
007401 TAGCGACTCA GCTGCCCCCA CTACGACCAG T**GG**AACTGTC CT**GG**AGA**GG**C T**GG**TTGTGAG TTCTTTAGAA GCCCTGGAAA GCTGCTTTGC CGTTGGCCCA
007501 ATCATCGAGA AGGAGAGAAA CAAGAATGCT GCTCAGGAGC TGGCCACTTT GCTGTTGTCC CTGCCAGCAC CTGCCAGTGT CCAGCAGCAG TCCAAGAGCC
007601 TTCTGGCCAG CCTGCACACC AGCCGCTCGG CCTACCACAG CCACAAGGAT CAGGCCTTGC TGAGCAAAGC TGTGCAGTGT CTCAACACAT CTAGCAAAGA
007701 **GG**GCAA**GG**AT TT**GG**ACCCTG A**GG**TGTTCCA GAGGCTAGTG ATCACAGCTC GCTCCATTGC CATCATGCGC CCCAACAACC TTGTCCACTT TACGGAGTCA
007801 AAGCTGCCCC AGATGGAAAC AGAA**GG**AAT**G G**ATGAA**GG**GA A**GG**AACCGCA GAAGCAGTTG GAAGGAGATT GCTGTAGTTT CATCACCCAG CTTGTGAACC
007901 ACTTCTGGAA ACTCCATGCA TCCAAACCCA AGAATGCCTT CTTGGCACCT GCCTGCCTTC CAGGACTAAC TCATATTGAA GCTACTGTCA ATGCTCTGGT
008001 GGACATCATC CATGGCTACT GTACCTGTGA GCTGGATTGT ATTAACACAG CATCCAAGAT CTACATGCAG ATGCTCTTGT GTCCTGATCC TGCTGTGAGC
008101 TTCTCTTGTA AACAAGCTCT AATTCGAGTC CTAAGGCCCA GGAACAAACG GAGACATGTG ACTTTACCCT CTTCCCCTCG AAGCAACACT CCAATGGGAG
008201 ACAAGGATGA TGATGACGAT GATGATGCAG ATGAGAAAAT GCAGTCATCA GGGATCCCGA ATGGTGGTCA CATCCGTCA**G G**AAAGCCA**GG** AACAGAGTGA
008301 **GG**T**GG**ACCAT GGAGATTTTG AGATGGTGTC TGAGTCGATG GTCCTGGAGA CAGCTGAAAA TGTCAACAAT GGCAACCCCT CTCCCCTGGA **GG**CCCTGCT**G**
008401 **G**CA**GG**CGCAG A**GG**GCTTCCC CCCCATGCTG GACATCCCAC CTGATGCAGA TGACGAGACC ATGGTTGAAC TAGCCATTGC CCTGAGCCTG CAGCAGGACC
008501 AACAAGGCAG CAGCAGCAGT GCCCTGGGCC TGCAGAGCCT GGGACTGTCC GGCCAGGCAC CCAGCTCTTC CTCTCTGGAC GCAGGAACCC TCTCTGACAC
008601 CACAGCATCA GCTCCAGCCT CAGACGACGA GGGCAGTACA GCAGCGACAG ATGGTTCTAC CCTTCGGACC TCTCCTGCTG ACCACGGT**GG** TAGTGT**GG**GC
008701 TC**GG**AGAGCG **GGGG**CAGTGC AGT**GG**ACTCA GT**GG**CT**GG**CG AGCACAGTGT ATCTGGCCGG AGCAGTGCTT ATGGCGATGC TACAGCTGAG G**GG**CATCC**GG**
008801 CT**GG**ACCA**GG** AAGTGTCAGC TCAAGCACTG GAGCCATCAG CACCACCACT GGGCACCAGG A**GG**GAGAT**GG** CTCCGA**GG**GA GAA**GG**AGAAG GAGAAACTGA
008901 AGGAGATGTC CACACTAGCA ACAGGCTGCA CATGGTCCGT CTAATGCTGT TGGAGAGATT ACTGCAGACC CTGCCTCAAT TACGAAACGT TGGCGGTGTC
009001 CGGGCCATCC CATACATGCA GGTCATTCTA ATGCTCACTA CAGATCT**GG**A T**GG**AGAAGAT GAGAAAGACA A**GG**G**GG**CCCT AGACAACCTG CTCTCCCAGC
009101 TTATTGCTGA GTTGGGTATG GATAAAAAGG ATGTCTCCAA GAAGAATGAG CGCAGCGCCC TGAATGAAGT CCATCTGGTA GTAATGAGAC TCCTGAGTGT
009201 CTTCATGTCC CGCACCAAAT CTGGATCCAA GTCTTCCATA TGTGAGTCAT CTTCCCTCAT CTCCAGTGCC ACAGCAGCAG CTCTACTGAG CTCTGGGGCT
009301 GTGGACTACT GCCTGCACGT GCTCAAATCA CTGCTGGAAT ATTGGAAGAG CCAACAGAAT GACGAGGAGC CTGTGGCTAC CAGCCAGTTG CTGAAACCAC
009401 ATACTACCTC CTCCCCACCT GACATGAGCC CATTCTTTCT CCGCCAGTAT GTGAAGGGTC ATGCTGCTGA TGTGTTTGAG GCCTATACTC AGCTTCTAAC
009501 AGAAATGGTA CTGAGGCTTC CTTACCAAAT CAAAAAGATT ACTGACACCA ATTCTCGAAT CCCACCTCCT GTCTTTGACC ACTCGTGGTT TTACTTTCTC
009601 TCCGAGTACC TCATGATCCA GCAGACTCCA TTTGTGCGCC GTCAAGTCCG CAAACTTCTG CTCTTCATCT GTGGATCCAA AGAGAAGTAC CGCCAGCTCC
009701 GGGATTTGCA CACCCTGGAC TCTCACGTGC GTGGGATCAA GAAGCTGCTA GAAGAGCA**GG GG**ATATTCCT CC**GG**GCAAGT GT**GG**TTACAG CCAGCTCAGG
009801 CTCCGCCTTG CAATATGACA CACTCATCAG CCTGATGGAG CACCTGAAAG CCTGTGCAGA GATTGCCGCC CAGCGAACCA TCAACTGGCA GAAATTCTGC
009901 ATCAAAGATG ACTCCGTCCT GTACTTCCTC CTCCAAGTCA GTTTCCTTGT GGATGAGGGC GTGTCCCCAG TGCTGCTGCA ACTGCTCTCC TGTGCTCTGT
010001 GCGGCAGCAA GGTGCTCGCT GCACTGGCAG CCTCTTCGGG ATCCTCCAGT GCTTCTTCCT CCTCAGCCCC TGTGGCTGCC AGTTCTGGAC AAGCCACAAC
010101 ACAGTCCAAG TCTTCCACTA AAAAGAGCAA GAAAGAAGAA AAAGAAAAGG AGAAAGAT**GG** TGAGACCTCT **GG**CAGCCA**GG** A**GG**ACCAGCT GTGCACAGCT
010201 CTGGTGAACC AGCTGAACAA ATTTGCCGAT AAGGAAACCC TGATCCAGTT CCTGCGTTGT TTCCTGTTAG AGTCCAATTC TTCCTCGGTG CGCTGGCAGG
010301 CCCACTGTCT GACACTGCAC ATCTACAGAA ATTCCAGCAA ATCTCAACAG GAGCTCCTGC TAGATCTGAT GTGGTCCATC TGGCCAGAAC TCCCAGCCTA
010401 TGGTCGTAAG GCTGCCCAGT TTGTGGACCT ACTAGGATAT TTCTCCCTGA AAACTCCACA AACAGAGAAG AAGTTGAAGG AGTATTCACA GAAGGCTGTG
010501 GAGATTCTGC GGACTCAAAA CCATATTCTT ACCAACCACC CCAACTCGAA CATTTATAAC ACTTTGTCTG GCTTAGTGGA GTTTGATGGC TATTACCTGG
010601 AGAGCGATCC CTGCCTGGTG TGTAATAACC CGGAAGTACC GTTCTGTTAT ATCAAGCTGT CTTCCATTAA AGTGGACACG CGGTACACCA CCACCCAGCA
010701 GGTTGTGAAG CTCATTGGCA GTCACACCAT CAGCAAAGTG ACAGTGAAAA TCGG**GG**ATCT GAAAC**GG**ACC AAGAT**GG**TGC **GG**ACCATCAA CCTGTATTAT
010801 AACAACCGAA CCGTGCAGGC CATCGTGGAG TTGAAAAACA AGCCAGCTCG CTGGCACAAA GCCAAGAAGG TTCAGCTGAC CCCTGGACAG ACAGAGGTGA
010901 AGATTGACCT GCCGTTGCCC ATTGTGGCCT CCAATCTGAT GATTGAGTTT GCAGACTTCT ATGAAAACTA CCAGGCCTCC ACAGAGACCC TGCAGTGCCC
011001 TCGCTGTAGT GCCTCGGTCC CTGCCAACCC AGGAGTCTGT GGCAACTGTG GAGAGAATGT GTACCAGTGT CACAAATGCA GATCCATCAA CTACGATGAA
011101 AAGGATCCCT TCCTCTGCAA TGCCTGTGGC TTCTGTAAAT ATGCCCGCTT CGACTTCATG CTCTATGCCA AGCCTTGCTG TGCAGTGGAT CCCATTGAGA
011201 ATGAAGAAGA CCGGAAGAAG GCTGTATCCA ACATCAATAC ACTTTTGGAC AAAGCTGATC GAGTGTATCA TCAGCTGATG GGACACCGGC CACAGCTGGA
011301 GAACCTGCTC TGCAAAGTGA ATGAGGCAGC TCCAGAAAAG CCACA**GG**ATG ACTCA**GG**AAC AGCA**GG**G**GG**C ATCAGCTCCA CTTCTGCCAG TGTGAATCGT
011401 TACATCCTGC AGTTGGCTCA GGAGTATTGT GGAGACTGCA AGAACTCTTT TGATGAACTC TCCAAAATCA TCCAGAAAGT CTTTGCTTCG CGCAAAGAGT
011501 TGTTGGAATA TGACCTACAG CAGAGGGAAG CAGCCACTAA ATCATCCCGG ACCTCCGTGC AGCCCACATT CACTGCCAGC CAGTACCGTG CCTTATCCGT
011601 CCTGGGCTGT GGCCACACAT CCTCCACCAA GTGCTATGGC TGCGCCTCGG CTGTCACAGA ACATTGTATC ACACTACTTC GGGCCCTGGC CACCAACCCA
011701 GCCTTGAGGC ACATCCTTGT CTCCCAGGGC CTTATCCGGG AGCTCTTTGA TTATAATCTT CGCCGAG**GG**G CTGC**GG**CCAT GC**GG**GAGGA**G G**TCCGCCAGC
011801 TCATGTGCCT CCTAACTCGA GACAACCCAG AAGCCACCCA ACAGATGAAT GACCTGATTA TTGGCAAGGT CTCCACAGCC CTGAAGGGCC ACTGGGCCAA
011901 CCCCGATCTG GCAAGTAGCC TGCAGTATGA AATGCTGCTG CTGACGGATT CTATCTCCAA **GG**A**GG**ACAGC TGCT**GG**GAGC TCC**GG**TTACG CTGTGCTCTC
012001 AGCCTTTTCC TCATGGCTGT GAACATTAAG ACTCCTGTGG TGGTTGAAAA CATTACCCTC ATGTGCCTGA GGATCTTGCA GAAGCTGATA AAACCACCTG
012101 CTCCCACTAG CAAGAAGAAC AAGGATGTCC CCGTTGAGGC CCTCACCACG GTGAAGCCAT ACTGCAATGA GATCCATGCC CAGGCTCAAC TGTGGCTCAA
012201 GAGAGACCCC AAGGCATCCT ATGATGCCTG GAAGAAGTGT CTTCCTATCA GAGGGATAGA TGGCAATGGG AAAGCCCCCA GCAAATCAGA GCTCCGCCAT
012301 CTCTATTTGA CTGAGAAGTA TGTGTGGAGG TGGAAACAGT TCCTGAGTCG TC**GG**G**GG**AAG A**GG**ACCTCCC CCTT**GG**ATCT CAAACTGGGG CATAACAACT
012401 GGCTGCGACA AGTGCTTTTC ACTCCAGCAA CGCAGGCCGC ACGGCAGGCA GCCTGTACCA TTGTGGAAGC TCTAGCCACC ATTCCCAGCC GCAAGCAGCA
012501 GGTCCTGGAC CTGCTTACCA GTTACCTGGA TGAGCTGAGC ATAGCTGGGG AGTGTGCAGC TGAGTACCTG GCTCTCTACC AGAAGCTCAT CACTTCTGCG
012601 CACT**GG**AAAG TCTACTT**GG**C AGCTC**GGGG**A GTCCTACCCT ATGTGGGCAA CCTCATCACC AAGGAAATAG CTCGTCTGCT **GG**CCCT**GG**A**G G**A**GG**CTACCC
012701 TGAGTACCGA TCTGCAGCAG GGTTATGCCC TTAAAAGTCT CACAGGCCTT CTCTCCTCCT TTGTTGAGGT GGAATCCATC AAAAGACATT TTAAAAGTCG
012801 CTTGGTGGGT ACTGTGCTGA ATGGATACCT GTGCTTGC**GG** AAGCT**GG**T**GG** TGCAGA**GG**AC CAAGCTGATC GATGAGACGC A**GG**ACATGCT GCT**GG**AGATG
012901 CT**GG**A**GG**ACA TGACCACAGG TACAGAATCA GAAACCAAGG CCTTCATGGC TGTGTGCATT GAGACAGCCA AGCGCTACAA TCTGGATGAC TACCGGACCC
013001 CGGTGTTCAT CTTCGAGAGG CTCTGCAGCA TCATTTATCC TGAGGAGAAT GAAGTCACTG AGTTCTTTGT GACCCTGGAG AAGGATCCCC AACAAGAAGA
013101 CTTCTTACAG GGCAGGATGC CTGGGAACCC GTATAGCAGC AATGAGCCAG GCATCGGGCC GCTGATGAGG GATATAAAGA ACAAGATTTG CCAGGACTGT
013201 GACTTAGT**GG** CCCTCCT**GG**A AGATGACAGT **GG**CAT**GG**AGC TTCTAGTGAA CAATAAAATC ATTAGTTTGG ACCTTCCTGT GGCTGAAGTT TACAAGAAAG
013301 TCTGGTGTAC CACGAATGAG GGAGAGCCCA TGAGGATTGT TTATCGTATG C**GG**G**GG**CTGC TG**GG**CGATGC CACAGA**GG**AG TTCATTGAGT CCCTGGACTC
013401 TACTACAGAT GAAGAAGAAG ATGAAGAAGA AGTGTATAAA ATGGCT**GG**TG TGAT**GG**CCCA GTGT**GG**GGGC CT**GG**AATGCA TGCTTAACAG ACTCGCAGGG
013501 ATCAGAGATT TCAAGCAGGG ACGCCACCTT CTAACAGTGC TACTGAAATT GTTCAGTTAC TGCGTGAAGG TGAAAGTCAA CCGGCAGCAA CTGGTCAAAC
013601 TGGAAATGAA CACCTTGAAC GTCATGCTGG GGACCCTAAA CCTGGCCCTT GTAGCTGAAC AAGAAAGCAA **GG**ACAGT**GG**G **GG**TGCAGCTG T**GG**CTGAGCA
013701 GGTGCTTAGC ATCATGGAGA TCATTCTAGA TGAGTCCAAT GCTGAGCCCC TGAGTGAGGA CAAGGGCAAC CTCCTCCTGA CAGGTGACAA GGATCAACTG
013801 GTGATGCTCT TGGACCAGAT CAACAGCACC TTTGTTCGCT CCAACCCCAG TGTGCTCCAG GGCCTGCTTC GCATCATCCC GTACCTTTCC TTT**GG**AGA**GG**
013901 T**GG**AGAAAAT GCAGATCTT**G G**TGGAGCGAT TCAAACCATA CTGCAACTTT GATAAATATG ATGAAGATCA CAGTGGTGAT GATAAAGTCT TCCTGGACTG
014001 CTTCTGTAAA ATAGCTGCTG GCATCAAGAA CAACAGCAAT GGGCACCAGC TGAAGGATCT GATTCTCCAG AAGGGGATCA CCCAGAATGC ACTTGACTAC
014101 ATGAAAAAGC ACATCCCTAG CGCCAAGAAT TTGGATGCCG ACATCTGGAA AAAGTTTTTG TCTCGCCCAG CCTTGCCATT TATCCTAA**GG** CTGCTTC**GGG**
014201 **G**CCT**GG**CCAT CCAGCACCCT GGCACCCAGG TTCTGATTGG AACTGATTCC ATCCCGAACC TGCATAAGCT **GG**AGCA**GG**TG TCCAGTGATG A**GG**GCATT**GG**
014301 GACCTTGGCA GAGAACCTGC TGGAAGCCCT GCGGGAACAC CCTGACGTAA ACAAGAAGAT TGACGCAGCC CGCAGGGAGA CCCGGGCAGA GAAGAAGCGC
014401 ATGGCCAT**GG** CAATGA**GG**CA GAA**GG**CCCTG **GG**CACCCTGG GCATGACGAC AAATGAAAAG GGCCAGGTCG TGACCAAGAC AGCACTCCTG AAGCAGATGG
014501 AAGAGCTGAT CGAGGAGCCT GGCCTCACGT GCTGCATCTG CAGGGAGGGA TACAAGTTCC AGCCCACAAA GGTCCTGGGC ATTTATACCT TCACGAAGCG
014601 **GG**TAGCCTT**G G**A**GG**AGAT**GG** AGAATAAGCC CCGGAAACAG CAGGGCTACA GCACCGTGTC CCACTTCAAC ATTGTGCACT ACGACTGCCA TCTGGCTGCC
014701 GTCA**GG**TT**GG** CTCGA**GG**CC**G G**GAAGAGTGG GAGAGTGCCG CCCTGCAGAA TGCCAACACC AAGTGCAACG **GG**CTCCTTCC **GG**TCT**GGGG**A CCTCATGTCC
014801 CTGAATCAGC TTTTGCCACT TGCTTGGCAA GACACAACAC TTACCTCCAG GAATGTACAG GCCAGCGGGA GCCCACGTAT CAGCTCAACA TCCATGACAT
014901 CAAACTGCTC TTCCTGCGCT TCGCCATGGA GCAGTCGTTC AGCGCAGACA CT**GG**C**GG**G**GG** C**GG**CCGGGAG AGCAACATCC ACCTGATCCC GTACATCATT
015001 CACACTGTGC TTTACGTCCT GAACACAACC CGAGCAACTT CCCGAGAAGA GAAGAACCTC CAA**GG**CTTTC T**GG**AACAGCC CAA**GG**AGAAG T**GG**GTGGAGA
015101 GTGCCTTTGA AGTGGACGGG CCCTACTATT TCACAGTCTT GGCCCTTCAC ATCCTGCCCC CTGAGCAGTG GAGAGCCACA CGTGT**GG**AAA TCTTGC**GG**A**G**
015201 **G**CTGTT**GG**TG ACCTCGCA**GG** CTCG**GG**CAGT **GG**CTCCA**GG**T GGAGCCACCA GGCTGACAGA TAAGGCAGTG AAGGACTATT CCGCTTACCG TTCTTCCCTT
015301 CTCTTTTGGG CCCTCGTCGA TCTCATTTAC AACATGTTTA AGAAGGTGCC TACCAGTAAC ACAGAGGGAG GCTGGTCCTG CTCTCTCGCT GAGTACATCC
015401 GCCACAACGA CATGCCCATC TACGAAGCTG CCGACAAAGC CCTGAAAACC TTCCAGGAGG AGTTCATGCC AGTGGAGACC TTCTCAGAGT TCCTCGATGT
015501 GGCCGGTCTT TTATCAGAAA TCACCGATCC AGAGAGCTTC CTGAAGGACC TGTTGAACTC AGTCCCCTGA CCACCACACA GCAGCTGCGG CGGCGAAGAC
015601 GAAGCTGGCT TGCCTTCCAC CCTCTGTTCT CCCTCCTTGT GCATTAAGTT CCCTCCGCGG GATGCTGCAT TGTTACCCCG CCCTCCCCTC TCTCATTTTT
015701 CTT**GG**TGT**GG** CTT**GGGG**TTT TTAGGCTTCC TGTTTTATCT CGTGTGTGTG GTGCACCAGC TATGAGGTTG TCTGTAACCC AAGCCATCAA AGGGCCTGTA
015801 CATACCTAGG AGCCATGAGT TGTCCCGGCC AGCTTCATAC TTGAGTGTGC ACATCTTGAG AAATAAACAA GTGACTTAAC ACACATTGAA AA

**DYNC1H1**

000001 AGTCTGCGGT G**GG**CTAGC**GG** ACGGTCC**GG**C TTCC**GG**CGGC CGTTTCTGTC TCTTGCTGGC TGTCTCGCTG AGTCGCGGCC GCCTTCTCAT CGCTCCTGGA
000101 AGGTCCCGAG CGCGACACCA TGTCGGAGCC C**GG**G**GG**C**GG**C **GG**CGGCGA**GG** ACGGCTC**GG**C CGGATT**GG**AA GTGTC**GG**CCG TGCAGAATGT GGCGGACGTG
000201 TCGGTGCTGC AGAAGCACCT GCGCAAGCTG GTGCCGCTGC TGCT**GG**A**GG**A C**GG**C**GG**CGA**G G**CGCC**GG**CCG CGCT**GG**AGGC **GG**CGCTGGAG GAGAAGAGCG
000301 CCCTGGAGCA GATGCGCAAG TTCCTTTC**GG** ACCCGCA**GG**T CCACAC**GG**TG CTGGT**GG**AGC GCTCCACGCT CAAAGAGGAC GTCGGTGATG AAGGAGAAGA
000401 AGAAAAAGAA TTCATTTCCT ATAACATCAA CATAGACATT CATTATGGGG TTAAATCCAA TAGCTTGGCA TTCATTAAAC GTACTCCCGT GATTGATGCA
000501 GATAAACCCG TGTCTTCTCA GCTCCGGGTC CTTACACTCA GTGAAGACTC GCCCTACGAA ACTTTGCATT CTTTCATTAG CAATGCAGTG GCTCCTTTTT
000601 TTAAGTCCTA CATTAGAGAG TCT**GG**CAA**GG** CAGACA**GG**GA T**GG**TGATAAA ATGGCTCCTT CAGTTGAAAA GAAGATTGCA GAACTCGAAA TGGGACTCCT
000701 TCACTTGCAG CAAAATATTG AAATTCCGGA GATCAGCCTG CCGATTCATC CAATGATCAC AAATGTTGCA AAACAGTGTT ATGAGCGTGG AGAAAAGCCA
000801 AAAGTTACAG ACTTTGGTGA TAAGGTTGAA GACCCAACAT TTCTTAATCA GTTACAATCT GGAGTTAACC GCTGGATCCG AGAAATTCAA AAAGTGACCA
000901 AACTGGATCG AGATCCTGCA TCAGGAACTG CCTTACAGGA AATTAGTTTT TGGCTAAACT TGGAACGTGC GTTATACCGC ATCCAGGAGA AACGGGAGAG
001001 CCCGGAAGTT CTCCTGACTC TGGATATCTT GAAACATGGC AAGCGCTTCC ATGCCACCGT CAGTTTTGAC ACTGACACAG GTCTAAAACA GGCTTTGGAA
001101 ACTGTGAATG ACTACAATCC TCTGATGAAA GATTTCCCTC TGAATGATTT GCTGTCTGCC ACGGAGCTGG ACAAAATAAG ACAGGCGCTT GTTGCCATTT
001201 TCACACATTT GAGAAAGATC CGAAACACAA AATATCCTAT TCAGAG**GG**CA CTGCGTTT**GG** T**GG**A**GG**CAAT TTCAAGAGAC TTGAGTTCTC AATTACTCAA
001301 AGTATTGGGC ACTAGGAAAT TGATGCATGT TGCTTATGAA GAATTTGAAA AAGTTATGGT AGCATGCTTT GAAGTTTTTC AGACTTGGGA TGATGAGTAT
001401 GAGAAACTTC AGGTATTGTT GAGAGACATC GTCAAAAGAA AAAGGGAAGA AAATCTGAAG ATGGTGTGGC GTATCAACCC TGCCCACAGG AAGCTGCAGG
001501 CCCGCCTTGA CCAGATGAGA AAATTTAGAC GCCAGCATGA ACAGCTAAGA GCTGTTATCG TCAG**GG**TCCT GA**GG**CCACA**G G**TCAC**GG**CAG TTGCACAACA
001601 GAATCAAGGA GAGGTCCCTG AACCCCAAGA TATGAAAGTG GCTGAGGTTC TCTTTGATGC TGCAGATGCA AATGCCATTG AGGAAGTAAA CCTTGCTTAT
001701 GAGAACGTCA A**GG**AAGT**GG**A T**GG**ACT**GG**AT GTTTCCAAAG A**GG**GCAC**GG**A AGCCT**GG**GA**G G**CTGCTATGA AGAGGTACGA TGAGAGGATC GACAGAGTGG
001801 AGACCCGGAT CACCGCTCGC CTTCGGGATC AGCTTGGCAC AGCCAAGAAT GCCAACGAGA TGTTTAGGAT TTTCTCCAGG TTTAATGCAC TGTTTGTCAG
001901 GCCTCACATC CGTGGGGCCA TTCGCGAATA CCAGACCCAG CTGATCCAGC GCGTGAAAGA TGACATTGAG TCTCTTCACG ACAAGTTCAA GGTCCAGTAC
002001 CCACAGAGTC AGGCTTGTAA GATGAGTCAC GTTCGTGACT TGCCCCCTGT GTCAGGGTCT ATCATCTGGG CTAAACAGAT CGACA**GG**CAG CTGAC**GG**CCT
002101 ACATGAAGC**G G**GT**GG**AAGAT GTCCTT**GG**CA AG**GG**CTG**GG**A GAATCACGT**G G**A**GGGG**CAGA AGCTGAAGCA **GG**AT**GG**AGAC AGCTTCCGCA TGAAGCTCAA
002201 CACGCA**GG**AG ATCTTTGATG ACT**GG**GCAA**G G**AA**GG**TGCAG CAGCGCAACC TCGGTGTCTC GGGGCGCATT TTCACCATCG AAAGTACTCG **GG**TTC**GGGG**C
002301 CGAACT**GG**AA ATGTGCTTAA GCTGAAAGTT AACTTTCTTC CTGAGATTAT CACACTATCC AAAGAAGTCC GGAACCTCAA ATGGCTTGGT TTCCGCGTCC
002401 CACTGGCGAT TGTGAACAAA GCCCATCAAG CAAACCAGCT TTACCCGTTT GCCATCTCAC TGATCGAGAG CGTTCGTACC TATGAACGGA CCTGCGAGAA
002501 **GG**T**GG**A**GG**AG C**GG**AACACCA TTTCCCTTTT **GG**TGGCT**GG**C TTGAAAAA**GG** AAGTGCA**GG**C CCTGATCGCA GAAGGCATTG CGTTGGTGTG GGAGTCCTAC
002601 AAACTTGACC CATATGTACA GCGCTTAGCA GAGACTGTCT TCAACTTCCA AGAAAAGGTG GATGATCTGC TGATCATTGA AGAAAAAATA GACCTAGAAG
002701 TCCGTTCCTT GGAAACTTGT ATGTATGACC ATAAGACATT CTCGGAAATC TTGAACAGAG TCCAGAAAGC AGTGGATGAC TTAAATCTGC ACTCCTATTC
002801 CAATTTGCCC ATCTGGGTCA ACAAGCTTGA CATGGAGATT GAAAGAATAT TGGGCGTCCG TCTGCAAGCT GGCCTGAGAG CTTGGACGCA GGTTCTTCTT
002901 GGACAAGCTG AAGATAAAGC AGAAGTTGAC ATGGACACAG ATGCTCCACA AGTTAGTCAC AAGCCTGGTG GAGAGCCAAA GATCAAAAAT GTCGTTCATG
003001 AGCTAAGAAT AACCAATCAG GTAATCTACT TGAATCCACC AATTGAAGAG TGCAGATACA AGCTGTATCA GGAAATGTTT GCCTGGAAGA TGGTTGTACT
003101 GTCTCTCCCC A**GG**ATCCAGA GTCAGA**GG**TA CCA**GG**TG**GG**T GTACATTACG AATTGACTGA GGAAGAGAAA TTCTATCGGA ATGCTTTAAC ACGGATGCCT
003201 GATGGCCCTG TTGCCCTGGA AGAGTCGTAT TCTGCTGTCA TGGGCATTGT ATCTGAAGTT GAACAGTATG TCAAGGTTTG GCTTCAGTAT CAGTGTTTAT
003301 GGGATATGCA AGCTGAAAAC ATCTATAACA GACTTGGAGA AGATCTCAAC AAATGGCAGG CTCTCCT**GG**T CCAAATAA**GG** AA**GG**CCAGA**G G**AACCTTTGA
003401 CAATGCAGAA ACCAAGAAAG AGTTTGGACC AGTAGTTATA GATTATGGCA AGGTACAATC TAAGGTGAAC TTGAAATATG ACTCTT**GG**CA TAA**GG**A**GG**TT
003501 CTTAGCAAAT TT**GG**GCAGAT GCTAGGATCA AACATGACGG AATTCCATTC CCAGATCTCA AAGTCCCGCC AAGAGTTGGA GCAGCACTCA GTAGACACGG
003601 CCAGCACCTC CGATGCAGTG ACCTTCATCA CCTATGTGCA GTCTTTGAAA CGGAAGATCA AGCAGTTTGA GAAGCAAGTT GAGCTCTACC GCAATGGCCA
003701 GCGCTTACTG GAAAAGCAAA GGTTCCAGTT CCCACCTTCC TGGCTTTATA TTGACAACAT CGAGGGAGAG TGGGGAGCCT TCAATGACAT CATGCGGCGA
003801 AAGGACTCTG CCATTCAGCA GCAGGTGGCA AACCTGCAAA TGAAGATTGT CCA**GG**A**GG**AT CG**GG**CCGT**GG** AAAGCCGCAC CACCGACCTG CTGACTGACT
003901 GGGAGAAGAC CAAGCCTGTC ACGGGCAACC TTCGCCCAGA AGAGGCACTT CAGGCTCTCA CCATATATGA GG**GG**AAGTTT **GG**TA**GG**CTGA A**GG**ACGACAG
004001 AGAGAAGTGT GCAAA**GG**CCA A**GG**A**GG**CGCT **GG**AATTGACA GATACTGGGC TTCTCAGTGG CAGTGAAGAG CGCGTGCAGG TGGCCTTAGA AGAATTACAG
004101 GACCTCAAA**G G**CGTTT**GG**TC AGAACTTTCT AA**GG**TTT**GG**G AGCAAATCGA TCAGATGAAG GAGCAACCCT GGGTTTCAGT ACAGCCTCGA AAGCTTCGAC
004201 AAAATTTGGA TGCCCTCCTG AACCAGCTGA AAAGCTTCCC TGCCCGGTTG CGACAGTATG CGTCCTATGA GTTTGTTCAG AGGCTTCTGA AAGGTTACAT
004301 GAAGATAAAT ATGCTGGTGA TTGAACTGAA ATCCGAAGCA CTTAAAGACC GCCATTGGAA ACAGCTCATG AAAAGGCTTC ACGTTAATTG GGTTGTTTCT
004401 GAGCTAACCC TTGGCCAAAT CTGGGATGTT GACTTGCAGA AAAATGAAGC GATTGTCAAG GATGTACTGC TTGT**GG**CACA AG**GG**GAGAT**G G**CTTT**GG**AAG
004501 AATTTTTGAA GCAGATAAGA GAAGTGTGGA ATACTTATGA ACTAGACTTG GTTAATTATC AGAACAAGTG CCGCTTGATC CGTGGCTGGG ATGACCTCTT
004601 CAACAAGGTC AAAGAACACA TCAACAGCGT CTCGGCCATG AAGCTCTCTC CGTATTACAA GGTTTTTGAA GAGGATGCTC TCAGCTGGGA AGATAAGCTG
004701 AACAGGATCA TGGCTCTCTT TGATGTGTGG ATTGATGTGC AGAGGC**GG**TG **GG**TCTACCT**G G**AA**GG**TATCT TCACAGGCAG TGCAGATATC AAGCACCTGC
004801 TGCCAGTGGA AACCCAGCGG TTTCAGAGCA TCAGCACTGA GTTTTTGGCT CTAATGAAAA AAGTGTCCAA GTCTCCCCTT GTTATGGATG TTCTGAACAT
004901 CCAG**GG**AGTA CAGA**GG**TCTC T**GG**AAAGATT **GG**CAGACCTG CTAGGAAAGA TCCAGAAAGC ATTGGGAGAA TATCTGGAAA GAGAGCGGTC ATCTTTCCCC
005001 AGGTTCTATT TTGTGGGTGA TGAAGATTTG CTTGAAATCA TTGGAAACAG CAAGAATGTC GCTAAATTAC AGAAACACTT CAAGAAGATG TTTGCTGGAG
005101 TTTCGAGCAT CATCCTGAAC GAGGATAACT CTGTTGTTTT GGGTATTTCA TCTCG**GG**AA**G G**AGA**GG**A**GG**T TATGTTTAAA ACTCCTGTGT CAATTACTGA
005201 ACATCCCAAA ATCAATGAGT GGCTCACATT GGTAGAAAAG GAGATGAGAG TCACCCTGGC CAAACTGCTT GCTGAGTCTG TTACGGAAGT TGAGATTTTT
005301 GGTAAAGCAA CTTCAATTGA CCCAAATACC TACATCACTT GGATTGATAA ATACCAGGCC CAGCTTGTGG TTTTGTCAGC CCAGATAGCC TGGTCTGAGA
005401 ACGTGGAGAC CGCACTGAGC AGCATG**GG**C**G G**A**GG**T**GG**AGA TGCCGCGCCC TTGCACTCTG TGCTGAGCAA TGTGGAGGTC ACCCTCAATG TGTTAGCAGA
005501 CTCTGTCCTC ATGGAGCAGC CCCCACTCCG AAGGCGGAAG CTAGAACACT TGATTACAGA GTTGGTTCAC CAGAGAGATG TTACAAGGTC CTTGATCAAA
005601 AGCAAGATTG ACAACGCCAA ATCTTTTGAA TGGCTCAGCC AGATGCGATT TTACTTTGAC CCTAAGCAAA CTGATGTGTT ACAGCAGTTG TCAATTCAAA
005701 TGGCAAATGC CAAATTTAAC TATGGCTTTG AGTACCTGGG TGTTCAGGAC AAACTGGTCC AGACCCCCCT CACTGACCGC TGCTATTTGA CAATGACACA
005801 AGCCTTGGA**G G**CCA**GG**CT**GG** GG**GG**TTCCCC ATTTGGACCT GCTGGAACTG GGAAAACAGA GTCTGTCAAA GCTCTTGGCC ATCAGCTTGG ACGGTTTGTT
005901 TTAGTTTTCA ACTGTGATGA AACCTTTGAT TTCCA**GG**CAA T**GG**GCC**GG**AT CTTTGT**GG**GC CTTTGCCA**GG** TG**GG**TGCCT**G GGG**CTGCTTT GACGAGTTCA
006001 ACCGCCT**GG**A **GG**AGC**GG**ATG CTCTC**GG**CTG TGTCCCAGCA GGTGCAGTGC ATACAGGAAG CACTGCGTGA ACATTCCAAC CCCAACTACG ACAAGACCTC
006101 TGCCCCCATT ACTTGTGAGC TGCTGAACAA ACAAGTCAAG GTGAGCCCGG ACATGGCCAT CTTCATCACC ATGAACCCTG GCTACGCGGG CCGGTCTAAC
006201 CTTCCTGACA ACTTGAAGAA GCTGTTCCGG AGCTTGGCCA TGACCAAGCC CGACCGGCAG TTAATCGCCC AGGTCATGCT GTACTCACAG GGTTTCCGCA
006301 CTGCTGAAGT GCTTGCCAAC AAAATCGTCC CGTTTTTTAA ACTATGCGAT GAGCAGCTCT CTTCCCAAAG CCATTATGAC TTCGGTCTTC GGGCTTTGAA
006401 GAGTGTGCTG GTGAGTGCAG GCAATGTGAA GAGAGAGAGA ATCCAGAAGA TAAAGAG**GG**A GAAAGA**GG**AA CGA**GGGG**AAG CAGTTGATGA AGGAGAAATT
006501 GCTGAAAATC TCCCTGAACA AGAGATTCTG ATACAGAGCG TCTGTGAGAC GAT**GG**TGCCA AAGCT**GG**T**GG** CAGA**GG**ACAT CCCGCTGCTC TTCAGCCTCC
006601 TGTCGGACGT GTTCCCTGGA GTCCAGTATC ACAGGGGTGA GATGACTGCC CTTCGAGAGG AGCTGAAGAA AGTGTGTCAG GAGATGTATT TGACATATGG
006701 AGATGGAGAA GAAGTT**GG**T**G G**AATGTG**GG**T TGAAAA**GG**TT CTCCAGCTCT ATCAGATCAC CCAGATCAAT CATGGCCTGA TGATGGTG**GG** GCCCTC**GG**GA
006801 AGTG**GG**AAGA GCAT**GG**CCTG GCGTGTCCTG CTGAAGGCAT TGGAGAGACT CGAG**GG**TGT**G G**AA**GG**TGT**GG** CCCATATCAT CGACCCCAAG GCCATCAGCA
006901 AAGACCACCT CTAC**GG**AACC CT**GG**ACCCCA ACACCA**GG**GA AT**GG**ACAGAT GGGCTCTTCA CACACGTGCT GAGAAAGATC ATCGACAGCG TGAGAGGCGA
007001 GCTGCAGAAG CGCCAGTGGA TCGTCTTCGA TGGCGATGTG GATCCAGAGT GGGTTGAGAA CTTGAACTCA GTGCTGGATG ACAATAAGCT CCTAACTTTG
007101 CCCAATGGAG AGCGCCTCAG TCTTCCACCC AATGTGAGAA TAATGTTTGA GGTACAGGAC TTGAAATACG CGACCTTGGC CACAGTGTCG CGCTGC**GG**CA
007201 T**GG**TCT**GG**TT CAGTGA**GG**AT GTGCTGAGCA CCGACATGAT CTTCAACAAC TTCCTGGCCA GGCTGCGCAG CATCCCGCT**G G**ATGAAGG**GG** AGGATGA**GG**C
007301 ACAGC**GG**C**GG** CGTAAG**GG**CA AAGA**GG**ATGA GG**GG**GAGGAG GCCGCTTCCC CCATGCTGCA GATCCAAAGA GATGCAGCTA CGATCATGCA ACCGTACTTC
007401 ACGTCCAACG GCCTGGTCAC CAAGGCGCTA GAGCACGCCT TCCAGCTGGA GCACATCATG GACCTAACAC GCCTGCGCTG CCTGGGCTCG CTCTTCTCCA
007501 TGCTGCACCA GGCCTGCCGC AACGTGGCGC AGTATAACGC CAACCATCCC GACTTCCCCA TGCAGATCGA GCAGCTGGAG CGCTACATTC AGCGATATCT
007601 GGTTTATGCC ATACTCTGGT CCCTGTCTGG AGACAGCCGG CTAAAAATGA GAGCAGAGCT GGGTGAATAC ATCAGAAGAA TCACGACCGT GCCTCTGCCC
007701 ACTGCGCCCA ACATACCCAT TATCGATTAT GAGGTGTCCA TCAGC**GG**AGA AT**GG**TCTCCG T**GG**CAGGCCA A**GG**TGCCTCA GATTGAAGTG GAGACGCACA
007801 AGGTGGCAGC CCCTGATGTC GTCGTGCCAA CGCTGGACAC AGTCCGCCAC GAAGCCCTCT TGTACACTTG GCTGGCCGAA CACAAGCCCC T**GG**TCTTGTG
007901 T**GG**CCCTCCT **GG**GTCT**GG**CA AGACCATGAC ACTCTTCAGC GCCCTCCGGG CCTTGCCTGA CAT**GG**A**GG**T**G G**T**GG**GTCTCA ACTTCTCCAG TGCTACTACT
008001 CCAGAGCTGC TTCTGAAGAC TTTTGATCAC TACTGCGAGT ACAGGCGCAC ACCTAAT**GGG G**T**GG**TTTT**GG** CTCCTGTTCA ACTTGGAAAG TGGCTGGTGT
008101 TGTTCTGTGA TGAAATCAAC TTGCCAGATA TGGATAAATA TGGGACCCAG AGGGTCATAT CCTTCATCAG ACAGAT**GG**T**G G**AGCAC**GG**A**G G**CTTTTACCG
008201 TACCTCAGAT CAAACATGGG TGAAGCTGGA GAGAATCCAG TTTGTTGGGG CTTGTAATCC CCCCACAGAC CCTGGAAGAA AGCCCCTCTC ACACAGGTTC
008301 CTGCGCCACG TGCCTGTCGT GTATGTGGAT TACCCGGGCC CCGCCTCCCT CACACAGATC TACGGCACCT TCAACCGCGC CATGCTGAGG CTCATTCCAT
008401 CCCTGCGGAC GTATGCAGAG CCGCTCACTG CTGCCATGGT GGAGTTCTAC ACCATGTCTC AGGAGAGATT CACCCAGGAT ACACAACCTC ACTATATCTA
008501 TTCACCCCGT GAAATGACTA GGTGGGTGAG AGGCATCTTT GAAGCGCTGA GACCTCTGGA GACCCTGCCT GTTGAAGGCC TCATTCGGAT TTGGGCACAT
008601 GAAGCTCTGC GTCTCTTCCA AGATAGACTC GTAGA**GG**ATG A**GG**AGA**GG**CG TT**GG**ACTGAT GAGAACATCG ACACGGTTGC TCTGAAGCAC TTCCCTAACA
008701 TCGACAGAGA GAAGGCAATG AGCCGACCCA TCTTGTACAG CAACTGGCTG TCAAAGGATT ACATCCCAGT AGACCAAGAA GAGTTAAGAG ATTATGTCAA
008801 AGCTAGGCTG AAGGTCTTTT ATGAAGAAGA ACTTGATGTT CCGCTGGTGC TGTTTAATGA AGTCCTAGAC CACGTGCTGA GGATTGACAG AATATTCCGT
008901 CAACCTCAAG GCCACTTGCT TCTGATTGGT GTTAGTGGAG CAGGAAAAAC TACCCTGTCT CGTTTCGTCG CCTGGATGAA CGGTTTGAGT GTGTACCAGA
009001 TTAA**GG**TCCA TA**GG**AAGTAC ACA**GGGG**AAG ACTTTGATGA AGATCTACGG ACAGTGTTGA GACGTTCTGG CTGTAAAAAT GAAAAGATAG CATTTATAAT
009101 GGATGAATCT AATGTGTTAG ATTCTGGATT CCTGGAGCGA ATGAATACCC TTCT**GG**CCAA T**GG**AGA**GG**TG CCT**GG**TCTCT TTGAAGGAGA CGAGTATGCC
009201 ACCTTGATGA CGCAGTGCAA AGA**GG**G**GG**CA CAGAA**GG**AA**G G**CCTGATGCT GGACTCGCAC GAGGAGCTCT ACAAGTGGTT CACTAGCCAG GTTATCCGCA
009301 ACCTCCACGT CGTGTTCACC ATGAACCCGT CCTC**GG**AG**GG** ACTCAA**GG**AC CG**GG**CAGCTA CATCACCAGC ACTTTTCAAC A**GG**TGTGTGT TGAATT**GG**TT
009401 T**GG**AGACT**GG** TCCACCGAAG CACTGTATCA GGTTGGCAAA GAATTCACAA GTAAGATGGA TCTGGAGAAG CCAAATTACA TCGTGCCTGA TTACATGCCA
009501 GTTGTGTATG ATAAGCTGCC GCAGCCACCA TCCCATCGGG AAGCCATTGT GAACAGCTGT GTGTTTGTTC ATCAGACTCT TCACCAGGCG AATGCTC**GG**C
009601 TAGCAAAGCG A**GG**C**GG**CAGA ACGAT**GG**CCA TCACCCCTCG CCACTACCTG GACTTCATCA ATCACTATGC CAACCTGTTC CACGAGAAGC GGAGCGAGCT
009701 GGAGGAGCAG CAGATGCACT TGAACGTGGG GCTCAGGAAG ATCAAAGAGA CAGTCGACCA GGTAGAAGAA CTGCGTCGTG ACTTGAGGAT AAAGAGCCAA
009801 GAGCTGGAGG TGAAGAATGC AGCAGCCAAT GACAAGCTGA AAAAGATGGT GAAAGACCAG CAGGAGGCTG AAAAGAAGAA GGTTATGAGC CAAGAAATCC
009901 AGGAACAGCT GCATAAGCAG CAGGAGGTAA TTGCAGACAA ACAGATGAGT GTCAAAGAAG ATCTTGATAA GGTGGAACCT GCCGTCATTG AGGCCCAGAA
010001 TGCTGTGAAG TCGATCAAGA AGCAGCACCT **GG**T**GG**A**GG**TG A**GG**TCCATGG CCAACCCTCC TGCTGCTGTG AAGCTGGCGC TGGAGTCCAT CTGCCTGCTG
010101 CTGGGGGAAA GCACCACAGA CTGGAAGCAG ATCCGCTCCA TCATCATGCG GGAGAACTTC ATCCCCACCA TCGTCAACTT CTCTGCAGAG GAGATCAGTG
010201 ACGCCATAAG GGAGAAGATG AAGAAAAATT ACATGTCCAA TCCAAGTTAC AATTATGAAA TTGTGAATCG GGCTTCCCT**G G**CTTGC**GG**CC CTAT**GG**TGAA
010301 AT**GG**GCAATT GCACAGCTTA ACTATGCAGA CATGTTAAAG AGAGTGGAGC CCCTACGCAA TGAGCTGCAG AAGCTGGAAG ATGACGCCAA **GG**ACAACCAG
010401 CAGAA**GG**CCA ACGA**GG**T**GG**A GCAGATGATC CGAGACCTGG AAGCCAGCAT CGCCCGCTAC AAGGAGGAAT ACGCCGTCCT GATCTCAGA**G G**CCCA**GG**CCA
010501 TCAA**GG**CAGA CCT**GG**CAGCT GTCGAGGCAA AAGTAAACCG GAGCACTGCT CTTCTGAAGA GCTTGTCTGC TGAACGTGAA CGATGGGAAA AAACAAGTGA
010601 AACTTTCAAA AACCAGATGT CCACCATTGC TGGGGACTGT CTCTTGTCAG CTGCGTTCAT TGCCTACGCG GGTTACTTTG ACCAGCAGAT GCGTCAGAAC
010701 TTGTTCACTA CCTGGTCCCA TCACCTACAG CAAGCCAACA TCCAGTTCCG TACAGATATT GCCAGGACGG AATACCTTTC CAATGCTGAT GAGCGTCTTC
010801 GCTGGCAGGC CAGCTCCTTG CCTGCTGATG ACCTTTGCAC AGAAAATGCC ATCATGCTGA AACGATTCAA TAGGTATCCG CTGATCATTG ACCCCTCTGG
010901 ACAGGCCACA GAATTCATTA TGAATGAATA TAAGGATCGT AAGATCACAC GGACCAGCTT CCTGGATGAC GCCTTCAGAA AGAACTTAGA GAGTGCACTG
011001 AGATTC**GG**TA ACCCCCTTCT **GG**TCCA**GG**AT GT**GG**AAAGCT ACGATCCAGT TTTGAACCCG GTGCTGAACC GTGAAGTGCG GCGAACAG**GG** G**GG**AGAGTGC
011101 TGATCACTCT C**GGGG**ACCAG GACATAGACC TGTCGCCATC GTTTGTCATC TTCCTGTCCA CCCGGGATCC AACTGTCGAG TTCCCACCAG ATCTCTGTTC
011201 CCGGGTTACT TTTGTAAACT TCACAGTTAC CCGTAGCAGT TTACAAAGCC AGTGTCTAAA TGAAGTACTT AAAGCAGAAA GACCTGATGT GGACGAGAAA
011301 CGATCTGATC TTCTTAAACT TCAAGGGGAA TTTCAGCTCC GTTTGCGTCA GCTGGAAAAA TCTCTACTAC AAGCTCTGAA CGAGGTGAAA GGGCGCATTT
011401 TGGATGACGA CACGATCATA ACCACTCTGG AGAACCTGAA GAGAGA**GG**CT GCAGA**GG**TCA CCA**GG**AAAGT TGA**GG**AGAC**G G**ACATTGTCA TGCA**GG**A**GG**T
011501 **GG**AGACCGTG TCCCAGCAGT ACCTCCCGCT CTCCACCGCC TGCAGCAGCA TCTACTTCAC CATGGAGTCC CTCAAGCAGA TACACTTCTT GTACCAGTAC
011601 TCCCTCCAGT TTTTCCTGGA CATTTATCAC AACGTCCTAT ACGAGAACCC GAACCTGAAG GGTGTCACCG ACCACACACA GCGCCTGTCC ATTATAACAA
011701 AGGACCTCTT CCA**GG**T**GG**CG TTTAACCGAG T**GG**CTCGA**GG** CATGCTGCAT CAGGACCACA TTACCTTTGC CATGCTGCTG GCAAGAATCA AACTGAAGGG
011801 CACCGTGGGG GAGCCCACCT ACGATGCAGA ATTCCAGCAC TTCTTGAGAG GAAATGAGAT TGTCCTGAGT GCTGGCTCCA CCCCCAGGAT CCAGGGCCTG
011901 ACTGTGGAGC A**GG**C**GG**A**GG**C **GG**TGGTGAGG CTGAGCTGCC TTCCCGCGTT TAAGGACTTG ATTGCAAAGG TTCAGGCAGA CGAGCAATTT **GG**CATCT**GG**C
012001 T**GG**ACAGCAG CTCCCC**GG**AG CAGACTGTGC CCTACCTCTG GAGTGAAGAA ACACCTGCAA CACCCATTGG CCAGGCCATC CACCGCCTGC TCCTGATCCA
012101 GGCTTTCCGG CCCGATCGCC TGTTGGCCAT GGCCCACATG TTTGTTTCAA CAAACCTTGG GGAGTCTTTC ATGTCCATCA TGGAGCAGCC GCTCGACCTG
012201 ACCCACATTG TGGGCACAGA GGTGAAGCCC AACACTCCTG TCTTAATGTG CTCTGTGCCT GGTTATGATG CCAGTGGACA TGTCGAGGAC CTTGCAGCCG
012301 AGCAGAACAC GCAGATCACT TCAATTGCAA TCGGCTCTGC AGAAGGCTTT AACCAAGCAG ATAAGGCAAT AAACACCGCT GTAAAGTCGG GCAGGTGGGT
012401 GATGCTGAAG AATGTGCATC T**GG**CCCCA**GG** GT**GG**CTGATG CAGCT**GG**AGA AGAAGTTGCA TTCCCTGCAG CCGCATGCCT GCTTCCGACT CTTCCTCACC
012501 ATGGAGATCA ACCCCAAGGT GCCTGTGAAT CTGCTCCGTG CGGGCCGCAT CTTTGTGTTC GAGCCACCGC CA**GGGG**TGAA **GG**CCAACATG CTGA**GG**ACGT
012601 TCAGCAGCAT TCCCGTCTCA CGGATATGCA AGTCTCCCAA CGAGCGTGCC CGCTTGTACT TCCTGCTGGC CTGGTTTCAT GCGATCATCC AAGAACGCTT
012701 ACGATACGCA CCACT**GGGG**T **GG**TCAAAGAA GTATGAATTT **GG**AGAGTCTG ACCTGC**GG**TC AGCTTGCGAT AC**GG**T**GG**ACA CGT**GG**CT**GG**A TGACAC**GG**CC
012801 AA**GG**GCA**GG**C AGAACATCTC ACCGGATAAG ATCCCGTGGT CTGCACTAAA GACCTTAAT**G G**CCCAGTCCA TTTAT**GG**CG**G G**CGCGT**GG**AC AACGAGTTTG
012901 ACCAGCGTCT GCTCAACACC TTCCTGGAGC GCCTGTTCAC AACCAGGAGT TTCGACAGTG AGTTTAAGCT GGCATGCAAG GTCGACGGAC ATAAAGACAT
013001 TCAAATGCCA GAT**GG**CATCA **GG**CGAGA**GG**A GTTTGTGCAG T**GG**GTGGAGT TGCTCCCCGA CACCCAGACG CCCTCCTGGC TGGGCCTGCC CAACAACGCC
013101 GAGAGAGTCC TCCTTACCAC ACAGGGTGTG GACATGATCA GTAAAATGCT GAAGATGCAG ATGTT**GG**A**GG** ATGA**GG**ACGA CCT**GG**CCTAC GCAGAGACTG
013201 AGAAGAAGAC GAGGACAGAC TCCACGTCCG ACGGGCGCCC TGCCTGGATG CGGACACTGC ACACCACCGC GTCCAACTGG CTGCACCTCA TCCCCCAGAC
013301 GCTGAGCCAC CTCAAGCGCA CCGTGGAGAA TATCAAGGAT CCTTTGTTCA GGTTCTTTGA GAGAGAAGTG AAGATGGGCG CAAAGCTGCT TCAGGACGTT
013401 CGCCAGGACC TTGCAGATGT CGTCCAGGTG TGCGAAGGAA AGAAGAAGCA GACCAACTAC TTGCGCACGC TGATCAACGA GCTAGTGAAA GGGATCTTGC
013501 CTCGGAGCTG GTCCCACTAC ACGGTGCCTG CCGGCATGAC CGTCATCCAG TGGGTGTCCG ACTTCAGCGA GAGGATCAAA CAGCTGCAGA ACATCTCACT
013601 **GG**CAGCTGCA TCT**GG**T**GG**CG CCAA**GG**AGCT AAAGAACATC CACGTGTGCC TGGGTGGCCT GTTCGTGCCT GAGGCGTACA TCACTGCCAC CA**GG**CAGTAT
013701 GT**GG**CCCA**GG** CCAACAGCT**G G**TCCCTGGAG GAGCTCTGCC TGGAAGTCAA CGTCACCACC TCACAGGGCG CCACCCTTGA CGCTTGCAGC TTC**GG**AGTCA
013801 CG**GG**TTTGAA ACTTCAA**GGG G**CCACGTGCA ACAACAACAA GCTGTCACTG TCCAATGCCA TCTCAACCGC CCTTCCCCTG ACGCAGCTGC GCTGGGTCAA
013901 GCAGACAAAC ACCGAGAAGA AGGCCAGTGT GGTAACCTTA CCTGTCTACC TGAACTTCAC CCGTGCAGAC CTCATCTTCA CCGTGGACTT CGAAATTGCT
014001 ACAAA**GG**A**GG** ATCCTCGCAG CTTCTACGAG C**GGGG**TGTCG CAGTCTTGTG CACAGAGTAA ACTTTTCTAG CTGCCCCTTT CTGTAATAGT GAAAGTTGGT
014101 ATTTAACATT TATTCATTTT TAAAATATTT GGAAGGTCTG AGCTTGTGAA AAGAAAGT**GG** TT**GG**TCTGA**G G**TT**GG**AGGAA GCTGAATGGA ATCTGAC**GG**T
014201 TG**GG**AGT**GG**T **GG**AAATTGGA AGGATACCAG GA**GG**TATTT**G G**GAA**GG**CCAA T**GG**CGTGGCT CCTTTGAGGA AATAAAACAC TAAGCATGAG CCGGCTCCGC
014301 CTCTTCTGTC TCCGCTTTCA TCCCAGGGCA CAGAGCCTTG CCTTCCATGC TGCCCAGGGA GGGCAGCCCA CGGCAGCCAT GCCCCTCCCC ACCTCGCTTT
014401 CATCATGAGC TCGCTCCCGA GCGGCCACAG CACTCATGAA TGAAGACCTT GGGGCCCTTC ACAGACACAG ATGCAGCCAG CTGTGGCTCT GAA**GG**CCCTG
014501 **GG**GCCCG**GG**C ACCAT**GG**TTC ACACCTTTAA TCGCAGCACT TTGGGAGTCT GGGAGTTAAA GACCAGCCTC GGCAACATAG TGAGACCCCG TCTCTACA**GG**
014601 AAATTAAATC A**GG**TGT**GG**T**G G**TGCATGCCT GTAGTCCCAG CTACTTGAG**G G**CTGA**GG**TG**G G**A**GG**ATCACC CAAGCCCAAG AGGTCGAGGC TGCAGTGAGC
014701 TGTGATCTCA CCACTGCACT CCAGCCTGGG TGACAGAGCA AGACCCTGTC TCAAAAAAAA AAAAAGCTGG GCGTGGTGGC TCATGCCTGT AATCCCAGCA
014801 CTTT**GG**GA**GG** CCGA**GG**CG**GG** C**GG**ATCACCA AATTAGCC**GG** GCAT**GG**T**GG**C ACATGCCTGT AATCCCAGCT ACTC**GG**GA**GG** CTGA**GG**CA**GG** AGAATTGCTT
014901 GAACCTG**GG**A **GG**C**GG**A**GG**TT GCAGTGAGCT GAAAAAAAAA AAGGCAGCCC CCAGCCGCTT GTGTTCTTGA CCAG**GG**CCCC A**GG**ACTT**GG**C TCCTCCAGAC
015001 AA**GG**GAGTTT TGTGCTGTAG ATGAGGGAGT TGCCCATCGC CGCCCTAGCA AGTCCATTCC CACACGACCT TTCCAGTGGT GATGATGACA GTGGCCCATA
015101 CAGCTGACTG TTTGCATCTC ACGTTCACAT TGCTAGAGGT GATGGGTGTG CTACACCCGT GGAAACAGGC TTCTGGCATC TCAGTGTCTT TATTTTATTT
015201 TTGAGATGGA GTTTCGCTCT TGTTGCCCAG GCTGGAGTGC AGTGGCGTGA TCTCAGCTCA CTGCAACCTC CACCTACCGG GTTCAAGTGA TCCTCCTGCC
015301 TCGGCCTCCT GAATAGCTGG GATTACAGGC ATGCACCACC ATGCCCAGCT AATTACTATA TTTTTAGTAG AGACAGGGCT TCACCGTGTT **GG**CCA**GG**CT**G**
015401 **G**TCTCAAACT CCT**GG**CCTCA GCCAGTGATC CTCCTGCCTC AGCCTCCCAA AGTGCTGAGA TGACAGGCAT GAGCCACTGC ACCTGGCCAA ATTTTTGTAT
015501 TTTTTGTAGA GATGGAATCT CGCTATTTTC CCT**GG**GCT**GG** TCTCAAACTC CT**GG**CATTAA **GG**GCTCCTCC TATCTCAGCC TCTCAAAGTG CCGGGGTTCT
015601 AGGTGTGAGT CACCGAGTTG AGCCCCTAAA CACATGTTTT TCTTTTTAGA GACAG**GG**TCT CACTC**GG**TTG CCCA**GG**CT**GG** AGTGCAGTGG CGTGACCATG
015701 GCTCACTGCA GCCTCAACCT CCCGGGCTCA AGTGATCCTC CCACCTCAGC CTTCTGAGCA GCTGGGACCA CAGACACACA CCACCATGTC GGCTAATTTT
015801 TGTATCTTTT GTAGAGACAA GGTTTCACCA TGTTGCTCGG GCTGGTCTCG AACTCCTGAG CTCAAGTGGT CCTCCCACCT TGGCCTCCCA AAATGCTGGG
015901 ATTACAAGTG TGAGCCACCA CACCCAGCCC CTAAAAACAT TTATGTGCAT CGACATCAGC TTTGATCAGA AGAGCCCCTG CTCCTCTTGG GCTGGGACCC
016001 CTTCCTGGAC TGAGTCTGCT CACCTTGGAT TAGGCCACCT ACTGCTTCTT CCTCTTCCTT TTCTCAGGCT CTGCAAGGAA GAGCTCCAGC TTCCTCTTGG
016101 AGAAGGAGAT TCTGGGCCCC TTTTCTCTCT CCCAAACCTA GGTGGTGGCC ATGCCCCTCG AGCTCTGCTT GGCACGTGTC TGCCAGTCTC A**GG**G**GG**CTCC
016201 AT**GG**GAGTGA **GG**AAG**GG**CTC GGC**GG**CCCTG **GG**GGTCT**GG**T CCTCCTCCGC CATCCTCGAG TCCTCGGGCT ACGTGCCCTC ATCTTT**GG**CT TCCA**GG**GACA
016301 CG**GG**CCCAGC T**GG**CCACTTC TATGACAAGA ATGTGGGTTT TTTGTGACAG GGCGTCTACT ATATGAACTT CCTCTGGACT CTGACTTCAG TGTGTAGGAA
016401 GCCACCTTAC AGCTCATGTC ACCCAGAGAA CAGTCGTTGC AGCTCCAGTT GGAATGACTG GGGGTGTCTT CCGAATCGCC ATCATAAAGA TCTAAAAGCT
016501 GAGGACAAAA GTGTTTCTCA GTCCAAGAGC AGTTCTGCGA GGCGGAATCA AGTCTAACAT GCTCGCATGC GCTGACTCTT CCTCCCGTCA CTGATGCTGG
016601 TTTTTGCAGG CTCTGCCTTG CAAATGAATT TTTCTTTCTT TTTTTTTTTT TTTTTGAGAC GGAATCTTGC TCTGTCGCCC AAGCTGGAGT GCAGTGGCGC
016701 AATCTGGGCT CACTGCAACC TCCACCTCCC GGATTCAAGC GATTCTCCTG CCTCAGCCTC CTGAGTAGCT GGGACTACAG GCGCCCGCTA ATTTTTGTAT
016801 TTTTAGTAGA GACGG**GG**TTT CACCATATT**G G**TCA**GG**CT**GG** TCTTGAACTC CTGACTCAGG TGATCCACCC GCCTCAGCCT CCCAAGGTGC TGGGATTACA
016901 GGTGTGAGCC ACCACGCCTG GCCGAATTTT TATTTGTTTT TTGTTTGTTT TTGTTTTTTT TAAAAAACTG AGTCTCGCAC TGTTGTCCA**G G**CT**GG**AGTGC
017001 AGT**GG**CGCGA TCTC**GG**CTCA CTGCAAGCTC CGCCTCCCAG GTTCACGCCA TTCTCCTGCC TCAGCCTCCC AAGTAGCTGG GACTACAGGC GCCCACCACC
017101 AAGCCCGGCT AATTTTTTGG ATTTTTAGTA GAGACAGGGT TTCACTGTGT TAGCCAGGAT GGTCTCGCTC TCCTGACCTC GTGATCCACC CGCCTCCCAA
017201 AGTGCTGAGA TTACAGGCGT GAGCCACCAC ACTCGGCCTC TTTGTTTGTT TTTTTTTTTT TTTGAGACAG TCTGGCTCTG TCACTGAGGC TGGAGTGCAG
017301 TGATGCAACC TCATCTCACT GCAGCCTAGA CCTCCTGGGC TTAAGTGATC CTCCCACCTC AGCCTCCCCA GACTACAGGT GCACACCACC ACGCCCGGCT
017401 AATTTTTGTA TTTTTTGTAG AGATGGAGTT TCGCCATGTT GCCCAGGATG GCCTTGAACT CCAGGGCTCA AGCAATCCAC CCACCTCAAC CTCCCAAAGT
017501 GCTGGGATTA CAGGCATGAG CCATTGTGCC CAGCTGCAAA TGAATTTTTA AAAATGTGTT AGATCAATAT TCATATCACC CAAAAACAGC CTGAGAGCCC
017601 GCCACCACTG GGGACTCAGC CTGAGCCTTG TTCCCAGAAA GCCCTCAACG CAGTGCTG**GG** CACAGACCT**G G**GA**GG**CTT**GG** CTCAAACCGC CCTGCCCTCC
017701 GTTGGCAGGG CAGAGCTCCG TCTTTTCCTC TCAACAGCTC TTCCAAA**GG**G CAA**GG**CAGCA TTTTCCT**GG**T GAG**GG**GCAAG CGAAGCTGAG GCCAGACCCA
017801 CCCCCGAGTC TTCCCTCTGC CACATCCAG**G G**CCTGAAG**GG** CGTTCT**GGGG** AGCCTCCACC TTCACACCGA A**GG**CCGCCAA **GG**T**GG**TT**GG**C AAGAAGCTGA
017901 AATGGGCGTT TAAGTCCGAA CCTTGTTTTC AAAGGTGCCC ATTGCCTGTT CTCAAAGCAA TGCACCTGAG AAGCAGTGGC AGGTGGCACA GCCCACCCCC
018001 AACCGCCAGG GCCACCCAAA CACCAGTTAC AACACCATCA CCAACAGAGC CTTTGCAGAA CTTTTTTTTT TTTTTTTTTT TTTGAGACGG AGTCTTGCTC
018101 TGTCGCCCA**G G**CT**GG**AGTGC AAT**GG**TGTGA TCTT**GG**CTGA CTGCAACCTC CGCCTCCCAA GTTCAAATGA TTCTCCTACC TCAGCCTCCC AAGCAGCTGG
018201 GACTACAGGC ACCCACCACC ATGCCCGGCT AATTTTTCTA TTTTTAGTAG AGACAGGGTT TTGCCATGTT GCCCAGGCTG GTCTCGAACT CCTGACCTCA
018301 GGTGATCTGC CCACCTTGGC CTCCCAAAGT GCTGGGATTA CAGGCATGAG CCACCACTCC TGGCCCTCTT GTATTTTTTT GTAGAGACAG GGTTTTGCCA
018401 TGTTGCCCAG GCTGGTCTCC TGGGCTCAAG TGATCCACCC ACCTTGGTCT CCCAAAGTGC TAGGATTATG GGCCTGAGCC ACCCCACCCG GCCCAACCTT
018501 ATTATTACTG AAGTCGCCTT GTTACACACA AACATCTACA GTGCCTCCTG GCCACTCCTT CTGGACTTTT CTTTCCTAGC CCA**GG**TCAAA GT**GG**GCCTTC
018601 G**GG**AGGACCC T**GG**AGGAGAA GCCCCCTCCT CTTCACTTCC ACCCTCTCTC TTCCACCCCT GGAAGTCAGC TTTCTGACCT CAGAATCCCA GCTATCTTCT
018701 GGACTCAGGT GACAAGTCAC CTAAGTGACA GATCCAGCAT GCACTTTATG ACCATCACTC TGCTCCTCCT GCATCAGCGC CCCACCCAAC AGAGCCGACC
018801 AGGCTGGGAA CACCGAGACC GAGAGCGCGT GGCTGTGTCT ACAAAGGCCT CCTCTTTGAG GGAGACACGC GA**GG**CAC**GG**G T**GGGG**CGATG AGACAGGATC
018901 GGCCGGTCCT TGACAGTCTC TGAAGCTGGG AGAACATGAC CCCATGCTCT CCTGTTGGAG TCACCACCTC TTTGCCAACA GCCACCAAAC CTCCACCTCC
019001 AATTAAGCTC CACCCCCATG TCCCCACTGC CCTCCGGACA TCTGTTCAGC ACCCCCCTCG ACCTGGCCAA GGCTCCACGC ACCGCCCTGG TTTCCCGCTC
019101 TGCCCAGCCC CCCGCTGAAG TCTCTAATGG GTTCTTCCGA TTCCACCTGT CCAAGGTAAA ACCCTGAGGT TGTCCCTCCT CCGC**GG**CACC TCCA**GG**TATC
019201 T**GG**TTGCTCA **GG**CTGAAATG TTTCCCAAGG CCCTGGCTCC TCCTCCTTCC AAACCCCGCA GGCAGCTGAT TAGCAAACTC TGACTCTAAC CTCAAAACAT
019301 GTCCAGAATT CAGCCACATC TCCCCACTTC CCCGCCTGGC CCTGGGCCCC GCTATTCCAT CTCAATCTCT GCCCTGGCCC CCAGCCCAGC ATCCACTCTC
019401 AGACCAGAGC AATGCTTGTG AACTCCGAAA ATCTGAGACA GGTCTCAGTT AATTTAGAAA AGATATTTGT CAAGGTTGAG GACGCGCACC CATGACACAG
019501 CCTCA**GG**A**GG** TCCTGACT**GG** GG**GG**AGAGAG ACCCTCTCAT ATTGTTTTAT ACTCAGTACC TGTTTTAAGA AAAAAACGAA GTGAAATCAA AGACA**GG**CAG
019601 CCC**GG**CGCCA **GG**CCCAAAAC CA**GG**CCTGGG CCTGCCTGGC CTAAACCTAG TCGTTAAAAA TCAGCTCATG ACTTAGAACC CGATGTTACC CATAGATTTC
019701 AGGCATTGTA TGGAAGAACA TCGTGAGACT CCCTGCTCTG TTCTGTTTCA CTCTGACTAC CAGTGCATGA AACCCCTGTC ACGTATCCCC CAGACTGCTC
019801 AATCAATCAC GACCCTTTCA CGTAAAATCT TTAGTGTTGT GAGCCCTTAA AAGGGACAGA AATTGTGCAC TT**GGGG**AGCT G**GG**ATTTTAA GAT**GG**TAACT
019901 TGCCGATGCT CCCAGCTGAA TAAAGCCCTT CCTTCTACAA

**AHNAK**

000001 AGAGCCTGGC CCGGCGCCAG CAGCCTGA**GG** AATGT**GG**TGG TT**GG**AGCC**GG** TGACTAATTC AAACCAGAAC TCAGGGAGGA GTGGTTGTGA CCGAGATTCC
000101 CGACGAGAGA GACTGAGGGG AAGAGA**GG**AA **GG**AG**GG**GC**GG** GCTCCTGGCA AGGCATTCGC TCCTGAGCGG AATCCTGCAA AGAT**GG**AGAA **GG**A**GG**AGACA
000201 ACCC**GG**GAGC TGCTGCTGCC CAACT**GG**CAG **GG**TAGT**GG**CT CCCAC**GG**GCT GACCATCGCC CAGAG**GG**ACG AC**GG**CGTCTT TGTGCA**GG**A**G G**TGACGCAGA
000301 ACTCCCCTGC GGCCCGCACT **GG**GGT**GG**TCA A**GG**AGG**GG**GA CCAGATTGTG GGTGCCACCA TCTACTTTGA CAACCTGCAG TCGGGTGAGG TGACCCAGCT
000401 GCTGAACACC AT**GGGG**CACC ACAC**GG**TG**GG** CCTGAAGCTG CACCGCAAGG GGGACCGCTC TCCCGAGCCT GGCCAGACCT GGACCCGTGA AGTCTTCAGC
000501 TCCTGCAGCT CTGAAGT**GG**T TCTGAGC**GGG G**ATGATGA**GG** AGTACCAGCG CATCTACACC ACGAAGATCA AGCCACGGCT GAAGTC**GG**AA GAT**GG**AGTGG
000601 AA**GG**AGACCT C**GG**GGAGACC CAGAGCCGTA CCATCACAGT GACCAGAAGG GTCACGGCCT ACACTGT**GG**A TGTGACT**GG**C CGGGAA**GG**AG CCAA**GG**ACAT
000701 AGACATCAGT AGCCCTGAAT TCAAGATCAA GATTCCAAGA CATGAACTGA CTGAAATCTC CAATGTGGAT GTGGAGACCC AGTCTGGGAA GACCGTGATC
000801 AGACTGCCCT CGGGCTCGGG GGCAGCCTCT CCGACA**GG**CT CTGCTGT**GG**A TATCCGAGCA **GGGG**CCATTT CTGCTTCAGG ACCAGAGCTC CAAGGTGCTG
000901 GCCACTCGAA GCTCCAGGTC ACCATGCCTG **GG**ATAAA**GG**T GGGA**GG**CTCA **GG**TGTCAATG TCAATGCAAA GGGCTTGGAC TT**GG**GT**GG**CA GA**GG**AGG**GG**T
001001 CCAAGTTCCA GCAGTGGACA TTTCATCTTC TCTTG**GG**GGT AG**GG**CAGTAG A**GG**TACAG**GG** CCCATCTCTG GAGAGTGGTG ATCATGGCAA AATTAAATTT
001101 CCCACCATGA AAGTGCCGAA ATTTGGTGTC TCAACAG**GG**C GTGAG**GG**CCA GACACCAAA**G G**CAG**GG**CTGA GGGTTTCTGC ACCTGAAGTC TCTGTGG**GG**C
001201 ACAA**GG**GC**GG** CAAGCCA**GG**C TTGACTATCC AAGCCCCTCA GCTGGAAGTC AGTGTGCCCT CTGCCAATAT TGAG**GG**CCTT GA**GGGG**AAGC TGAA**GG**GCCC
001301 CCAAATCACT GGGCCATCAC TTGAGGGTGA CCTAGGCCTG AAAGGTGCCA AGCCACA**GGG G**CACATT**GG**G GT**GG**ATGCCT CTGCTCCCCA AATT**GG**G**GG**T
001401 AGCATCACT**G G**CCCCAGTGT **GG**AAGTTCA**G G**CCCCTGACA TTGATGTTCA **GGGG**CCT**GG**G AGCAAACTGA ATGTGCCCAA GATGAAAGTC CCCAAGTTCT
001501 CTGTATCA**GG** TGCAAA**GG**GA GA**GG**AAACTG **GG**ATTGATGT GACACTGCCT ACA**GG**TGAAG TGACTGTTCC T**GGGG**TCTCT **GG**GGATGTCA GCCTGCCTGA
001601 GATTGCTACT **GG**TG**GG**CT**GG** AA**GG**AAAGAT GAAAGGTACT AAAGTGAAGA CTCCTGAAAT GATTATTCAG AAACCTAAAA TCTCCATGCA GGATGTGGAT
001701 CTGAGCCTTG GGTCTCCTAA ACTGAAAGGA GATATTAAGG TTTCTGCTCC T**GGGG**TGCAA **GG**TGATGTTA AA**GG**CCCTCA AGTGGCACTT AAAGGCTCCA
001801 GAGTGGACAT AGAGACACCA AACCTAGAG**G G**AACCTTGAC A**GG**CCCTA**GG** CTT**GG**CAGTC CTTCCGGGAA AACCGGAACC TGTAGGATCT CTATGTCAGA
001901 AGTAGACTTA AATGTGGCCG CACCTAAAGT GAAAGGGGGT GTAGATGTCA CACTCCCCAG AGTAGAAGGG AAAGTCAAAG TCCCTGAAGT TGATGTCAGA
002001 GGCCCCAAAG TGGATGTCAG TGCCCCAGAT GTCGAAGCGC ATGGCCCAGA ATGGAACCTG AAAATGCCCA AGATGAAAAT GCCCACGTTC AGCACTCCA**G**
002101 **G**AGCCAAA**GG GG**AA**GG**TCCA GATGTTCATA TGACTCTACC CAAAGGAGAT ATCAGTATTT CAGGGCCCAA GGTCAATGTG GAAGCCCCAG ATGTCAACTT
002201 **GG**AG**GG**TCT**G G**GG**GG**AAAAC TTAAAGGCCC CGATGTTAAG CTGCCTGATA TGAGTGTCAA GACACCAAAG ATCTCCATGC CTGATGTAGA TTTGCACGTG
002301 AAAGGTACAA AGGTGAAGGG AGAGTATGAT GTAACTGTAC CAAAGCT**GG**A A**GG**AGAACTC AAA**GG**CCCAA AAGT**GG**ACAT TGATGCCCCA GATGTGGATG
002401 TTCATGGCCC AGACTGGCAC TTGAAGATGC CCAAGATGAA AATGCCCAAA TTCAGTGTGC CAGGGTTCAA AGCAGAGGGC CCAGAAGTGG ATGTGAACCT
002501 GCCCAAGGCT GATGTGGACA TTTCCGGGCC CAAGATAGAT GTTACTGCTC CTGATGTGAG CATTGAGGAA CCAGAAGGGA AATTGAAAGG GCCCAAGTTT
002601 AAGATGCCTG AGATGAACAT CAAAGTCCCC AAGATCTCCA TGCCTGATGT GGACTTACAT CTGAAAGGCC CTAACGTAAA GGGAGAATAT GATGTCACAA
002701 TGCCAAAGGT TGAAAGTGAG ATTAAAGTTC CTGATGTTGA ACTTAAAAGT GCCAAAATGG ACATTGATGT CCCAGATGT**G G**A**GG**TTCAA**G G**CCCAGACT**G**
002801 **G**CACCTGAAG ATGCCCAAGA TGAAAATGCC CAAGTTCAGC ATGCCTGGCT TCAAAGCAGA GGGCCCAGAA GTGGATGTGA ACCTGCCCAA **GG**CTGATGT**G**
002901 **G**ACATCTCA**G G**ACCCAA**GG**T GGGTGTTGAA GTTCCAGATG TGAATATTGA AGGACCTGAA GGAAAGCTGA AGGGCCCCAA GTTCAAGATG CCAGAGATGA
003001 ATATCAAGGC CCCCAAGATC TCCATGCCTG ATGTGGACTT GCATATGAAA GGTCCTAAAG TAAAGGGAGA ATATGATATG ACAGTGCCAA AGCT**GG**AA**GG**
003101 **GG**ACCTGAAA **GG**CCCAAAAG TAGATGTCAG TGCCCCAGAT GTTGAAATGC AGGGTCCTGA CTGGAACTTG AAGATGCCAA AGATTAAAAT GCCCAAATTT
003201 AGCATGCCCA GCCTCAAAGG AGAGGGGCCA GAATTTGATG TGAACCTGTC CAAAGCGAAT GTGGACATTT CTGCACCAAA AGTAGATACT AATGCTCCAG
003301 ATCTGAGCCT TGAAGGACCT GAAGGGAAGT TGAAAGGCCC GAAGTTTAAG ATGCCTGAGA TGCACTTCAG AGCTCCTAAG ATGTCTTTGC CAGATGTTGA
003401 CCTGGATCTT AAAGGACCCA AAATGAAAGG AAATGTAGAT ATCTCTGCAC CAAAGATAGA GGGTGAAATG CAGGTTCCAG ATGTGGACAT CAGAGGTCCC
003501 AAGGTAGATA TTAAAGCACC AGATGTGGAA **GG**CCAA**GG**CC T**GG**ACT**GG**AG CCTGAAAATA CCCAAGATGA AAATGCCCAA GTTCAGCATG CCCAGCCTCA
003601 AAGGCGAGGG CCCAGAAGTG GATGTGAACT TGCCTAAGGC TGACGTTGTT GTCTCAGGAC CCAAGGTGGA CATCGAAGCC CCAGATGTGA GCCTCGAAGG
003701 TCCAGAAGGG AAGCTGAAGG GTCCCAAGTT TAAGATGCCT GAGATGCATT TCAAGACCCC CAAGATCTCC ATGCCTGATG TGGACTTACA CTTGAAA**GG**C
003801 CCCAAAGTCA AA**GGGG**ATGT **GG**ATGTGTCT GTGCCCAAGG TAGAAGGTGA AATGAAAGTG CCAGATGTTG AAATCAAAGG ACCCAAAATG GACATTGATG
003901 CCCCAGATGT **GG**A**GG**TTCAA **GG**CCCAGACT **GG**CACCTGAA GATGCCCAAG ATGAAAATGC CCAAGTTTAG CATGCCT**GG**C TTCAAA**GG**AG AG**GG**CCGAGA
004001 AGT**GG**ATGTG AACCTGCCCA AGGCTGACAT TGATGTCTCA GGACCCAAGG TGGATGTTGA AGTCCCAGAT GTGAGCCTTG A**GG**GCCC**GG**A A**GG**AAAGCTG
004101 AA**GG**GCCCCA AGTTTAAGAT GCCTGAGATG CACTTCAAGG CCCCCAAGAT CTCCATGCCT GATGTGGACC TGAATCTTAA **GGGG**CCAAAA TTGAA**GG**GAG
004201 ATGT**GG**ATGT GTCCTTGCCT GAGGTAGAAG GTGAAATGAA AGTGCCAGAT GTTGACATTA AAGGGCCCAA AGTTGACATT AGTGCTCCAG ATGTGGATGT
004301 TCATGGCCCA GATTGGCACC TGAAGATGCC CAAGGTGAAA ATGCCCAAGT TCAGCATGCC C**GG**CTTCAAA **GG**AGAG**GG**CC CTGAAGT**GG**A TGTGAAGCTG
004401 CCCAAAGCTG ACGTTGATGT CTCAGGACCC AAAATGGATG CTGAAGTTCC AGATGTGAAT ATTGAAGGTC CAGACGCAAA ACTAAAAGGT CCCAAATTCA
004501 AGATGCCAGA AATGAGTATA AAGCCTCAGA AGATATCCAT ACCAGATGTT GGTTTGCATT TGAAAGGTCC TAAAATGAAA GGAGATTATG ATGTAACAGT
004601 TCCAAAAGTA GAAGGAGAGA TAAAAGCTCC TGATGTTGAC ATCAAAGGCC CCAAAGTTGA TATTAATGCA CCAGATGT**GG** A**GG**TTCAT**GG** CCCAGACT**GG**
004701 CACCTGAAGA TGCCCAAGGT AAAAATGCCC AAGTTCAGCA TGCCT**GG**CTT TAAA**GG**AGAG **GG**CCCAGA**GG** TGGATATGAA CCTGCCCAA**G G**CTGACCTT**G**
004801 **G**TGTTTCA**GG** ACCCAA**GG**TG GACATTGATG TTCCAGATGT GAATCTTGAA GCTCCAGAGG GGAAACTAAA AGGCCCTAAG TTCAAGATGC CAAGCATGAA
004901 TATACAGACG CACAAAATCT CTATGCCTGA TGTTGGACTT AATTTGAAAG CCCCTAAACT GAAAACTGAT GTAGATGTTT CCCTTCCCAA AGTGGAAGGA
005001 GACTTGAAGG GTCCTGAAAT TGATGTGAAA GCCCCTAAGA TGGATGTGAA TGTTGGTGAT ATTGATATTG AAGGTCCAGA AGGGAAGTTG AAGGGCCCCA
005101 AGTTTAAGAT GCCTGAGATG CATTTCAAGG CCCCCAAGAT CTCCATGCCC GATGTGGACT TACACTTGAA A**GG**CCCCAAA GTCAAA**GGGG** ATAT**GG**ATGT
005201 GTCTGTGCCC AAGGTAGAAG GTGAAATGAA AGTGCCAGAT GTTGACATTA AAGGGCCCAA AGTGGACATT GATGCCCCAG ATGTGGAGGT TCACGACCCA
005301 GATTGGCACC TGAAAATGCC CAAGATGAAA ATGCCCAAGT TCAGTATGCC TGGCTTCAAA GCAGAGGGCC CTGAAGTGGA TGTGAATCTG CCAAAGGCTG
005401 ACATTGATGT GTCTGGACCC AGTGTGGACA CTGATGCTCC TGATTTGGAT ATTGAGGGAC CAGAAGGAAA GTTGAAAGGC TCCAAATTTA AGATGCCCAA
005501 GTTGAATATA AAAGCTCCCA AGGTCTCCAT GCCAGATGTG GACTTGAATT TGAAGGGACC CAAACTGAAG GGAGAGATAG ATGCTTCTGT GCCAGAACTG
005601 GAAGGTGATC TCAGAGGGCC GCAAGTTGAT GTCAAA**GG**TC CTTTTGT**GG**A AGC**GG**A**GG**TG CCCGATGTTG ATCTGGAGTG TCCTGATGCA AAGTTGAAAG
005701 GGCCCAAGTT TAAGATGCCT GAGATGCACT TCAAGGCCCC CAAGATCTCC ATGCCTGATG TGGACTTACA CCTGAAAGGC CCCAAAGTCA AAG**GG**GATGC
005801 **GG**ATGTGTC**G G**TGCCAAAAT T**GG**AGGGAGA TTTAACA**GG**C CCCAGTGT**GG** GTGT**GG**A**GG**T GCCTGATGTT GAGCTGGAGT GTCCTGATGC AAAGTTGAAA
005901 GGCCCTAAAT TTAAGATGCC AGACATGCAC TTCAAGGCCC CCAAGATCTC CATGCCTGAT GTGGACTTAC ACTTGAAAGG CCCCAAAGTC AAAG**GG**GATG
006001 T**GG**ATGTGTC **GG**TGCCAAAA TT**GG**AGGGAG ATTTAACA**GG** TCCCAGTGT**G G**GTGT**GG**A**GG** TGCCTGATGT TGAGCTGGAG TGTCCTGATG CAAAGTTGAA
006101 AGGGCCCAAG TTTAAGATGC CTGAGATGCA CTTCAAGACC CCCAAGATCT CCATGCCTGA TGTGGACTTA CACCTGAAA**G G**CCCCAAAGT CAAA**GGGG**AT
006201 AT**GG**ATGTGT CTGTGCCCAA GGTAGAAGGT GAAATGAAAG TGCCAGATGT TGACATCAAA GGACCCAAAA TGGACATTGA TGCCCCAGAT GTGGATGTTC
006301 ATGGCCCAGA CTGGCACCTG AAGATGCCCA AGATGAAAAT GCCCAAGTTC AGCATGCCTG GCTTCAAAGC AGAGGGCCCA GAAGTGGATG TGAACTTGCC
006401 CAAGGCTGAT GTTGTTGTCT CAGGACCCAA GGTGGATGTT GAAGTCCCAG ATGTGAGCCT TGAAGGTCCA GAAGGGAAGC TGAAGGGCCC CAAGCTTAAG
006501 ATGCCTGAGA TGCACTTCAA GGCCCCCAAG ATCTCCATGC CTGATGTGGA CTTACACTTG AAA**GG**CCCCA AAGTCAAA**GG GG**ATGT**GG**AT GTGTCTTTGC
006601 CAAAATT**GG**A G**GG**AGATTTA ACA**GG**CCCCA GTGT**GG**ATGT GGAGGTGCCT GATGTTGAGC TGGAGTGTCC TGATGCAAAG TTGAAAGGGC CCAAGTTTAA
006701 GATGCCTGAG ATGCACTTCA AGACCCCCAA GATCTCCATG CCTGATGTGA ACTTAAACTT GAAA**GG**CCCC AAAGTCAAA**G GGG**ATAT**GG**A TGTGTCTGTT
006801 CCCAAGGTAG AAGGTGAAAT GAAAGTGCCA GATGTTGACA TCAGAGGGCC CAAAGTGGAC ATTGATGCCC CAGATGTGGA TGTTCATGGC CCAGACTGGC
006901 ACCTGAAGAT GCCTAAGATG AAAATGCCCA AGTTCAGCAT GCCT**GG**CTTC AAA**GG**AGAG**G G**CCCAGAAGT **GG**ATGTGAAC TTGCCCAAGG CTGACGTTGA
007001 TGTCTCAGGA CCCAAGGTGG ATGTTGAAGT CCCAGATGTG AGCCTTGAAG GTCCAGAAGG GAAGCTGAAG GGCCCCAAGT TTAAGATGCC TGAGATGCAC
007101 TTCAAGACCC CCAAGATCTC CATGCCTGAT GTTGATTTCA ATTTAAAGGG ACCCAAAATC AAAGGAGATG TTGATGTTTC TGCCCCAAAG CT**GG**AG**GG**AG
007201 AGTTAAAA**GG** TCCAGAATT**G G**ATGTCAAAG GTCCCAAATT AGATGCTGAC ATGCCAGAAG TAGCTGT**GG**A A**GG**CCCAAAT **GG**CAAGT**GG**A AAACTCCTAA
007301 GTTCAAGATG CCAGATATGC ACTTTAAAGC TCCCAAAATC TCTATGCCAG ACCTCGATCT ACACTTGAAG AGCCCCAA**GG** CAAAA**GG**AGA **GG**T**GG**ATGTA
007401 GATGTTCCCA AATT**GG**AAGG **GG**ACCTTAAA G**GG**CCACATG T**GG**ATGTCAG TGGGCCAGAC ATTGACATTG AGGGACCAGA GGGCAAATTG AAAGGCCCTA
007501 AGTTCAAGAT GCCTGATATG CATTTCAAAG CCCCCAATAT TTCTATGCCT GATGTTGATC TAAATCTCAA A**GG**ACCCAAA ATCAA**GG**G**GG** ATGT**GG**ATGT
007601 GTCTGTGCCT GAGGTAGAAG GTAAACTTGA AGTACCAGAT ATGAACATCA GGGGCCCCAA AGTTGATGTA AATGCCCCCG ATGTCCAAGC TCCAGACTGG
007701 CACCTGAAGA TGCCCAAGAT GAAAATGCCC AAGTTCAGCA TGCCTGGCTT CAAAGCAGAG GGCCCTGAAG TAGACGTCAA CTTGCCTAAG GCTGACGTTG
007801 ACATCTCA**GG** ACCCAA**GG**T**G G**ACATTGAA**G G**CCCTGATGT TAATATTGAA GGACCAGAGG GAAAGTTGAA AGGGCCTAAG TTAAAGATGC CAGAGATGAA
007901 CATCAAAGCC CCCAAGATCT CCATGCCTGA CTTTGATTTG CATCTGAAA**G G**TCCCAA**GG**T GAAG**GG**CGAT GT**GG**ATGTTT CTCTGCCCAA AGTGGAAGGT
008001 GACCTCAAGG GCCCCGAAGT TGACATCAAG GGGCCCAAAG TGGATATTAA TGCCCCAGAT GTGGGTGTTC AAGGCCCAGA CTGGCACCTG AAGATGCCCA
008101 AGGTGAAAAT GCCAAAGTTC AGCATGCCT**G G**CTTCAAA**GG** AGAG**GG**CCCA GAT**GG**GGATG TGAAGCTGCC CAAGGCTGAC ATTGATGTCT CAGGACCCAA
008201 AGTGGACATT GAAGGCCCTG ATGTTAACAT TGAAGGACCA GAGGGAAAGT TGAAAGGGCC TAAGTTCAAG ATGCCAGAGA TGAATATCAA AGCCCCCAAG
008301 ATCTCCATGC CTGATATTGA CTTAAACCTG AAAGGACCCA AAGTGAAGGG TGATGTGGAT GTTTCCCTTC CTAAAGTGGA AGGTGACCTC AAGGGCCCAG
008401 AAGTTGACAT CAAGGGCCCA AAAGTGGACA TTGACGCACC TGATGTTGAT GTTCATGGCC CAGACTGGCA CCTAAAGATG CCCAAGATAA AAATGCCCAA
008501 GATCAGCATG CCT**GG**CTTCA AA**GG**AGAA**GG** TCCAGATGT**G G**ACGTGAACC TGCCCAAGGC TGACATTGAT GTCTCAGGAC CGAAAGTGGA TGTTGAATGT
008601 CCCGATGTGA ATATCGAAGG ACCTGAAGGA AAGTGGAAAA GTCCAAAGTT TAAGATGCCA GAGATGCATT TTAAGACTCC AAAGATATCC ATGCCAGATA
008701 TTGACCTGAA TCTCACAGGT CCAAAAATAA AA**GG**AGATGT **GG**ATGTTACA **GG**CCCTAA**GG** TAGAGGGAGA TCTGAAAGGT CCTGAAGTTG ACCTCAAAGG
008801 CCCCAAAGTG GACATTGATG TCCCAGATGT TAATGTTCAG GGTCCAGACT GGCACCTGAA GATGCCCAAG ATGAAAATGC CCAAGTTCAG CATGCCTGGC
008901 TTCAAAGCAG AGGGCCCTGA AGTGGATGTG AACCTGCCCA AGGCTGACGT TGATGTCTCA GGCCCCAAAG TGGACGTTGA AGGCCCTGAT GTTAACATTG
009001 AAGGACCAGA GGGAAAGTTG AAAGGGCCCA AGTTCAAGAT GCCAGAGATG AATATCAAAG CCCCCAAGAT CCCCATGCCT GACTTTGATT TGCATCTGAA
009101 A**GG**TCCCAA**G G**TGAAG**GG**CG ATGT**GG**ATAT TTCTCTGCCC AAAGTGGAAG GTGACCTCAA GGGCCCTGAA GTTGACATCA GGGGTCCCCA AGTGGACATT
009201 GATGTCCC**GG** ATGTG**GG**CGT TCAA**GG**CCCA GACT**GG**CACC TAAAAATGCC CAAAGTGAAA ATGCCCAAAT TCAGCATGCC T**GG**CTTCAAA **GG**AGAG**GG**CC
009301 CAGATGT**GG**A TGTGAACCTG CCCAAGGCTG ACCTTGATGT CTCAGGACCC AAGGTGGACA TTGATGTTCC AGATGTGAAT ATCGAAGGCC CAGAGGGAAA
009401 GTTGAAAGGT CCCAAATTCA AAATGCCTGA GATGAACATC AAAGCCCCCA AGATCTCCAT GCCTGACATT GATCTTAACC TGAAAGGTCC CAAAGTGAAG
009501 GGTGACATGG ATGTGTCTCT GCCAAAAGTG GAAGGTGACA TGAAAGTTCC TGACGTGGAT ATTAAAGGCC CCAAAGTGGA TATTAATGCC CCAGATGTGG
009601 ATGTTCAAGG CCCAGACTGG CACCTGAAGA TGCCTAAAAT AAAAATGCCC AAGATCAGCA TGCCT**GG**CTT CAAA**GG**AGAA **GG**TCCAGAAG T**GG**ACGTGAA
009701 CCTGCCCAAG GCTGACCTTG ACGTCTCAGG ACCCAAGGTG GACGTTGATG TTCCAGATGT GAATATTGAA GGTCCAGATG CGAAACTGAA GGGCCCTAAA
009801 TTCAAGATGC CAGAGATGAA CATCAAAGCT CCTAAAATAT CAATGCCTGA TTTGGACCTC AATCTTAAA**G G**CCCTAAAAT GAAA**GG**AGA**G G**T**GG**ATGTTT
009901 CACTTGCAAA TGTAGAAGGT GATTTGAAAG GACCTGCTCT TGACATAAAA GGCCCAAAGA TAGATGTAGA TGCTCCAGAT ATTGACATTC ATGGCCCAGA
010001 TGCCAAATTA AAAGGTCCAA AACTGAAGAT GCCTGACATG CATGTAAACA TGCCCAAGAT CTCCATGCCA GAAATTGACT TGAATTTGAA AGGCTCAAAG
010101 CTTAAGGGAG ATGTTGATGT CTCTGGGCCC AAGTTGGAAG GTGACATTAA AGCTCCCAGT TTGGATATAA AGGGCCCAGA AGTGGACGTT TCCGGTCCTA
010201 AGCTTAATAT CGAAGGCAAG TCAAAGAAAT CTCGTTTTAA GCTTCCCAAA TTTAATTTTT CGGGCTCTAA AGTTCAGACA CCTGAAGTGG ATGTCAAAGG
010301 TAAAAAGCCA GATATTGACA TAACAGGTCC AAAAGTTGAT ATTAATGCTC CTGATGTCGA GGTCCAAGGA AAAGTGAAAG GATCCAAGTT TAAAATGCCT
010401 TTCCTGAGTA TTTCATCTCC CAAAGTTTCT ATGCCTGACG TGGAGCTAAA TTTGAAAAGT CCCAAAGTCA AAGGAGACTT AGATATTGCA GGTCCCAATT
010501 TAGAAGGTGA CTTTAAAGGC CCCAAAGTGG ATATTAAGGC ACCAGAAGTC AATCTTAATG CACCTGATGT GGATGTTCAT GGTCCAGACT GGAATCTGAA
010601 AATGCCCAAG ATGAAAATGC CCAAATTCAG TGTGTCTGGC TTAAAAGCAG AAGGGCCAGA TGTAGCTGTG GATCTACCAA AAGGAGACAT CAACATAGAG
010701 GGCCCAAGTA TGAACATTGA GGGCCCAGAT CTCAATGT**GG** AA**GG**TCC**GG**A G**GG**AGGCTTG AAAGGTCCCA AATTCAAGAT GCCTGACATG AATATCAAAG
010801 CTCCCAAGAT CTCCATGCCT GACATTGACT TAAACTTGAA A**GG**CCCCAA**G G**TGAAA**GG**TG ATGT**GG**ATAT TTCTCTTCCC AAACTTGAA**G GGG**ATCTGAA
010901 A**GG**GCCAGA**G G**TTGATATCA AAGGCCCTAA AGTGGACATC AATGCCCCAG ATGTGGATGT TCATGGTCCA GACTGGCATC TGAAGATGCC CAAAGTGAAA
011001 ATGCCCAAGT TCAGCATGCC TGGCTTCAAA GGAGAAGGCC CTGAAGTCGA TGTTACCCTC CCTAAAGCTG ACATTGACAT TTCTGGTCCC AATGTAGACG
011101 TTGATGTTCC AGACGTGAAT ATTGAAGGTC CAGATGCAAA GCTGAAGGGC CCCAAGTTCA AGATGCCTGA GATGAACATC AAAGCCCCCA AGATCTCCAT
011201 GCCTGACTTT GACCTGAACT TGAAGGGACC CAAAATGAAG GGTGATGTGG TTGTGTCTTT GCCCAAAGTG GAAGGTGATC TAAAA**GG**CCC TGA**GG**T**GG**AC
011301 ATCAA**GG**GCC CCAAAGTGGA CATTGACACT CCTGACATTA ACATCGAAGG CTCAGAGGGT AAATTCAAGG GACCCAAATT TAAGATACCA GAGATGCACC
011401 TGAAGGCTCC CAAAATATCG ATGCCTGACA TTGATTTAAA CCTGAAGGGC CCCAAAGTCA AGGGCGATGT GGATGTTTCT CTGCCCAAAA TGGAAGGTGA
011501 CCTCAAGGGT CCTGAAGTTG ACATCAAGGG CCCCAAAGTG GACATTAATG CTCCAGATGT TGATGTTCAA GGCCCAGACT GGCACCTGAA GATGCCCAAG
011601 GTGAAAATGC CCAAGTTCAG CATGCCT**GG**C TTCAAA**GG**AG AG**GG**CCCAGA TGT**GG**ATGTG AACCTGCCCA AGGCTGACCT TGATGTCTCA GGACCCAAGG
011701 TGGACATTGA TGTTCCAGAT GTGAATATCG AAGGCCCAGA GGGAAAGTTG AAAGGTCCCA AATTCAAGAT GCCTGAGATG AACATCAAAG CCCCCAAGAT
011801 CTCCATGCCT GACATTGATC TTAACCTGAA AGGACCCAAA GTGAAGGGTG ATATGGATGT GTCTCTGCCA AAAGTGGAAG GTGACATGCA AGTTCCTGAC
011901 TTGGATATTA AAGGCCCCAA AGTGGATATT AATGCCCCAG ATGTGGATGT TCGAGGCCCA GACTGGCACC TGAAGATGCC TAAGATAAAA ATGCCCAAGA
012001 TCAGCATGCC T**GG**CTTCAAA **GG**AGAA**GG**TC CAGAAGT**GG**A TGTGAACCTG CCCAAGGCTG ACCTTGACGT CTCAGGACCC AAGGTGGACG TTGATGTTCC
012101 AGATGTGAAT ATTGAAGGTC CAGATGCGAA ACTGAAGGGC CCTAAATTCA AGATGCCAGA GATGAACATC AAAGCCCCCA AGATCTCCAT GCCTGACTTT
012201 GATTTGCATC TGAAA**GG**CCC TAA**GG**TGAAA **GG**AGATGT**GG** ATGTTTCTCT GCCTAAGATG GAAGGTGATC TAAAGGCCCC TGAAGTTGAC ATCAAGGGCC
012301 CCAAAGTGGA CATTGATGCC CCAGATGTGG ATGTTCATGG CCCAGACTGG CACCTGAAGA TGCCCAAGGT GAAAATGCCC AAATTCAGCA TGCCA**GG**ATT
012401 TAAA**GG**AGAG **GG**CCCAGAAG T**GG**ATGTTAA TTTGCCCAAA GCTGACATTG ATGTCTCAGG ACCCAAAGTG GACATTGACA CTCCTGATAT TGATATTCAT
012501 GGTCCAGAAG GGAAACTGAA GGGCCCCAAA TTTAAAATGC CTGACCTGCA CCTCAAGGCA CCGAAGATCT CTATGCCTGA AGTTGACCTG AATCTGAAAG
012601 GTCCAAAGAT GAAGGGCGAC GTGGACGTTT CTCTGCCCAA AGTGGAAGGC GACCTCAAGG GCCCTGAAGT TGACATCAAG GGCCCCAAAG TGGACATTGA
012701 TGTCCCAGAT GTGGACGTTC AAGGCCCAGA CTGGCACTTA AAAATGCCCA AAGTGAAAAT GCCCAAGTTC AGCATGCCT**G G**CTTCAAA**GG** AGAG**GG**CCCA
012801 GATGT**GG**ATG TGAACCTGCC CAAGGCTGAC CTTGACGTCT CAGGACCCAA GGTGGACATT GATGTTCCTG ATGTGAATAT CGAAGGTCCA GATGCGAAAC
012901 TAAAGGGCCC TAAATTCAAG ATGCCTGAGA TGAACATCAA AGCCCCCAAG ATCTCCATGC CTGACTTTGA TTTGCATCTG AAA**GG**TCCCA A**GG**TGAAG**GG**
013001 TGATGT**GG**AT GTTTCCCTTC CTAAAGTGGA AGGTGACCTC AAGGGCCCAG AAGTTGACAT CAAGGGCCCC AAAGTGGACA TCGATGCCCC TGATGTAGAT
013101 GTTCATGGCC CAGACTGGCA CCTGAAGATG CCCAAGGTGA AAATGCCCAA ATTCAGCATG CCA**GG**ATTCA AA**GG**AGAG**GG** CCCAGATGT**G G**ATGTTACCC
013201 TTCCTAAGGC TGACATTGAG ATTTCTGGCC CCAAAGTGGA CATTGATGCC CCTGATGTCA GTATCGAAGG TCCAGATGCA AAACTCAAGG GTCCAAAGTT
013301 CAAGATGCCA GAGATGAACA TCAAGGCCCC CAAAATCTCC ATGCCTGACA TTGACTTTAA CTTGAAGGGT CCCAAAGTGA AAGGTGATGT GGATGTCTCT
013401 CTGCCCAAAG TGGAAGGTGA TCTCAAGGGC CCTGAAATTG ACATAAAAGG CCCCAGTTTG GACATTGACA CACCTGATGT CAATATTGAA **GG**TCC**GG**AA**G**
013501 **G**AAAATTGAA **GG**GGCCCAAA TTTAAGATGC CTGAGATGAA CATCAAAGCT CCCAAAATCT CTATGCCTGA CTTTGATTTG CACCTGAAA**G G**TCCCAA**GG**T
013601 GAAG**GG**TGAT GT**GG**ATGTTT CACTACCTAA **GG**T**GG**AAAGT GATCTGAAA**G G**GCCAGA**GG**T AGACATTGAA GGTCCTGAAG GGAAGCTCAA AGGTCCCAAG
013701 TTTAAGATGC CTGATGTACA TTTCAAAAGC CCACAAATCT CCATGAGTGA CATTGATTTG AATTTGAAAG GACCTAAGAT AAAAGGAGAT ATGGACATTT
013801 CCGTTCCTAA ACT**GG**AG**GG**A GATCTGAAA**G G**TCCCAAAGT **GG**ATGTCAAA GGCCCTAAAG TGGGCATTGA CACTCCTGAT ATTGACATTC ATGGTCCAGA
013901 AGGGAAACTG AAGGGCCCCA AATTTAAAAT GCCTGACTTA CACCTCAAGG CACCGAAGAT CTCTATGCCT GAAGTTGACC TGAATCTGAA A**GG**TCCAAA**G**
014001 **G**TGAAG**GG**CG ACAT**GG**ACAT TTCTCTGCCC AAAGTGGAAG GCGACCTCAA GGGCCCCGAA GTTGACATCA GGGACCCCAA AGTGGACATT GATGTCCCAG
014101 ATGTGGACGT TCAAGGCCCA GACTGGCACC TAAAAATGCC CAAAGTGAAA ATGCCCAAGT TCAGCATGCC T**GG**CTTCAAA **GG**AGAG**GG**CC CAGATGT**GG**A
014201 TGTGAACCTG CCCAAGGCTG ACATTGATGT CTCAGGACCC AAAGTGGACG TTGATGTTCC TGATGTGAAT ATCGAAGGTC CAGATGCGAA ACTAAAGGGC
014301 CCCAAGTTCA AGATGCCTGA GATGAGCATC AAAGCCCCCA AGATCTCCAT GCCTGATATT GACTTAAACC TGAAAGGACC CAAAGTGAAG GGCGATGTGG
014401 ATGTTACCCT TCCTAAAGTG GAAGGTGACC TCAAGGGCCC AGAAGCTGAC ATCAAGGGCC CAAAAGTGGA CATCAACACC CCTGATGTGG ATGTTCATGG
014501 CCCAGACTGG CACCTGAAGA TGCCCAAGGT GAAAATGCCC AAATTCAGCA TGCCT**GG**CTT CAAA**GG**AGAA **GG**TCCAGATG T**GG**ATGTGAG CCTGCCCAAG
014601 GCCGACATCG ATGTCTCGGG ACCCAAGGTG GACGTTGATA TTCCAGATGT GAATATCGAA GGTCCAGACG CAAAACTGAA GGGCCCCAAG TTCAAGATGC
014701 CTGAAATAAA TATCAAAGCT CCCAAGATCT CCATACCTGA TGTTGACCTG GATTTGAAAG GACCCAAAGT AAAAGGAGAT TTTGATGTGT CTGTCCCTAA
014801 GGTTGAAGGG ACTTTGAAAG GCCCAGAAGT AGATCTTAAA GGTCCACGTC TGGATTTCGA AGGCCCTGAT GCCAAACTCA GTGGCCCATC TTTGAAGATG
014901 CCATCGCTGG AGATATCTGC TCCTAAAGTA ACTGCTCCTG ATGTTGATTT GCATCTCAAG GCACCAAAAA TTGGATTTTC AGGTCCGAAG TTAGAA**GG**T**G**
015001 **G**TGAAGT**GG**A CCTCAA**GG**GA CCCAAAGTTG AAGCTCCAAG CTTAGATGTA CACATGGACA GCCCAGATAT TAACATCGAA GGGCCAGATG TTAAAATCCC
015101 CAAATTTAAG AAACCCAAGT TTGGATTTGG GGCAAAAAGC CCCAAAGCTG ACATCAAGTC ACCTTCACTG GATGTCACTG TTCCTGAGGC AGAGCTGAAC
015201 CTTGAGACTC CTGAAATTAG TGTTGGTGGC AAGGGCAAGA AAAGTAAGTT TAAAATGCCT AAAATTCATA TGAGTGGTCC TAAGATTAAG GCCAAAAAAC
015301 AGGGATTTGA CCTGAATGTT CCTGGGGGTG AAATTGATGC CAGCCTCAAG GCTCCGGATG TAGATGTCAA CATCGCAGGG CCGGATGCTG CACTCAAAGT
015401 CGACGTGAAA TCGCCCAAAA CCAAGAAAAC GATGTTTGGA AAAATGTACT TCCCAGATGT AGAGTTTGAC ATTAAATCAC CTAAATTTAA AGCTGAGGCC
015501 CCTCTCCCTA GCCCCAAACT **GG**AG**GG**TGAA CTCCA**GG**CAC CTGATCT**GG**A ACTTTCTTTG CCAGCGATTC ACGTCGAAGG TCTTGACATC AAGGCGAAGG
015601 CTCCCAAGGT CAAGATGCCA GATGTGGACA TCTCAGTGCC AAAAATAGAG GGTGACCTGA AAGGCCCCAA AGTGCAGGCA AACTTGGGTG CACCTGACAT
015701 CAACATCGAA GGCCTAGATG CTAAAGTCAA AACACCGTCC TTCGGCATTT CTGCCCCTCA AGTCTCCATC CCTGATGTGA ATGTAAACTT GAAAGGACCA
015801 AAGATAAAG**G G**TGATGTCCC CAGCGT**GG**GA CT**GG**AA**GG**AC CAGATGTAGA TCTGCAAGGT CCAGAAGCAA AAATTAAGTT CCCCAAGTTT TCCATGCCCA
015901 AGATCGGCAT CCCAGGTGTG AAAAT**GG**AG**G G**T**GG**G**GG**AGC CGAGGTCCAT GCCCAGCTAC CCTCTCTTGA AGGAGACTTG AGAGGACCAG ATGTTAAGCT
016001 CGAAG**GG**CCC GATGTTTCTC TAAA**GGGG**CC A**GG**AGTAGAC TTGCCTTCAG TGAACCTCTC TATGCCAAAA GTCTCTGGGC CTGACCTTGA TCTGAACTTG
016101 AAAGGACCAA GTTTGAAGGG AGACCTGGAT GCATCTGTTC CCAGCATGAA GGTGCATGCT CCAG**GG**CTCA ACCTCAGT**GG** TGTC**GG**T**GG**C AAAATGCA**GG**
016201 T**GG**GA**GG**AGA C**GG**TGTGAAA GTGCCAGGGA TCGATGCCAC AACAAAGCTT AACGTTGGGG CACCAGATGT GACACTGA**GG GG**ACCAAGCC TGCA**GG**GAGA
016301 TCT**GG**CTGTC TCTGGTGACA TCAAATGCCC TAAAGTATCC GTAGGAGCTC CTGATCTAAG CTTGGAGGCA TCCGAAGGCA GCATTAAACT TCCCAAAATG
016401 AAGCTGCCCC AATTTGGCAT CTCTACTCCG GGGTCCGACT TGCACGTCAA TGCCAA**GGGG** CCACA**GG**TTT CT**GG**CGAACT GAA**GGGG**CCA **GG**TGT**GG**ATG
016501 TGAACCTGAA AGGGCCTCGG ATTTCAGCAC CGAATGTGGA CTTTAACTTG GAAGGACCAA AAGTGAAAG**G G**AGCCTT**GGG G**CCACT**GG**TG AGATCAAAGG
016601 CCCCACTGTC **GG**AGGA**GG**TC TTCCA**GG**CAT T**GG**TGTTCAA GGCCTAGAAG GAAACCTCCA GATGCCTGGA ATTAAGTCCT CTGGATGTGA TGTGAACCTG
016701 CCAGGCGTGA ATGTGAAACT CCCAACTGGG CAGATTTCTG GGCCTGAAAT CAAA**GG**T**GG**T CTGAAA**GG**TT CAGAAGTA**GG** TTTCCATGGG GCTGCTCCTG
016801 ATATCAGTGT GAAGGGGCCT GCCTTTAATA TGGCATCTCC TGAGTCAGAT TTTGGCATCA ACTTGAAGGG CCCAAAAATC AAA**GG**A**GG**TG C**GG**ATGTTTC
016901 A**GG**GGGTGTC AGTGCCCCAG ACATCAGCCT TGGTGAAGGG CATTTGAGTG TTAAA**GG**TTC CGG**GG**GTGAG T**GG**AAG**GG**AC CCCAAGTCTC CTCTGCTCTC
017001 AACTTGGACA CATCTAAGTT TGCTGGGGGC CTTCATTTCT CAGGACCAAA **GG**T**GG**AA**GG**A **GG**TGTGAAAG GA**GG**TCAGAT T**GG**ACTCCA**G G**CTCCTG**GG**C
017101 TGAGTGTGTC TG**GG**CCTCAA **GG**TCACTT**GG** AAAGT**GG**ATC TGGAAAAGTA ACATTCCCTA AAATGAAGAT CCCCAAATTT ACCTTCTCTG GCCGTGAGCT
017201 **GG**TT**GG**CAGA GAAAT**GG**GGG T**GG**ATGTTCA CTTCCCTAAA GCAGAGGCCA GCATCCAAGC T**GG**TGCT**GG**A GAC**GG**CGAGT **GG**GAAGAGTC TGAAGTCAAA
017301 CTGAAAAAGT CCAAGATCAA AATGCCCAAG TTTAATTTTT CCAAACCTAA A**GG**GAAA**GG**T **GG**TGTCACT**G G**CTCACCAGA AGCATCAATT TCTGGGTCCA
017401 AAGGTGACCT GAAAAGTTCA AA**GG**CCAGCC T**GG**GCTCTCT **GG**AAGGAGA**G G**CAGAGGCCG AAGCCTCTTC ACCGAAAGGC AAATTCTCCT TATTTAAAAG
017501 TAAGAAGCCA CGGCACCGCT CAAATTCATT CAGTGATGAA AGAGAGTTCT CTGGACCTTC CACCCCGAC**G G**GGACGCT**GG** AGTTTGAA**GG** TGG**GG**AAGTG
017601 TCTCT**GG**AAG GTG**GG**AAAGT TAAA**GG**GAAA CAC**GG**GAAGC TGAAATTCGG TACCTTT**GG**T **GG**ATT**GGGG**T CAAAGAGCAA AGGTCATTAT GAGGTGACTG
017701 GGAGCGATGA TGAGACAGGC AAGTTACA**GG GG**AGTGG**GG**T GTCCCT**GG**CC TCTAAGAAGT CCCGACTGTC CTCCTCTTCT AGCAATGACA GTG**GG**AATAA
017801 **GG**TT**GG**CATC CAGCTTCCCG A**GG**TGGAGCT GTCAGTTTCC ACAAAGAAAG AGTAGCAGGC CTTTGTATGT GTGTACATAT ATATATATAT AACAAAACAT
017901 CAGCCTTGGG TGGTGTGTTC CTATATAAAC TCCAAAGGGA AACACACCGA CTGCCTCAGC AATCATGCAA AGACCTTGCC TGGCCCGGTG GCAAGCGCTG
018001 AAAAACCGAC CGCCTGTAGG CTCCTGGAAC TATACAGATA GGTAAAGAGT TCCAAGTTCG TCCAGCCCAT GTGCAAAGTC AACAGTATTT GCCTTAAGAT
018101 TTCATATATA TATATTTTTT TGCATTGACT GCTGAGAGCT CCTGTTTACT AAGCAAGCTT TTGTGTTTAT TATCCTCATT TTTACTGAAC ATTGTTAGTT
018201 TTGGGGTAAT GGAAACCCAC TTTTTCATTG TAATGACTTT GGGGGCTTTT GTTAGTAA**GG G**T**GGG**TG**GGG** TGAT**GGG**TTG CAGACGGAGG TCAGGTCTTC
018301 CTCTTTCCTG AGACTGGATC TGTTCAAACA GCAAACGCCC ACAGATGGCC CAGA**GG**TGGT **GG**TAGTCA**GG** GTGTGT**GG**GT GTTTTTAGGG TTCTTTAGTG
018401 TTGTTTCTTT CACCCA**GGGG** T**GG**T**GG**TCCC AGCCAGTTTG GTGCTGACGG TGAGAGGAAA TTAGAATCTG TTTGCAAATT GTCCAACCCA CCCCCTCAAC
018501 ATGAGGGGCT TCCATTTTCT GTGTTTTGTA AGGGAACTGT TTCCTTCATG CCGCCATGTT CCTGATATTA GTTCTGATTT CTTTTTAACA AATGTTATCA
018601 TGATTAAGAA AATTTCCAGC ACTTTAATGG CCAATTAACT GAGAATGTAA GAAAATTGAT GCTGTACAAG GCAAATAAAG CTGTTTATTA ACCTTGA
