# Supplementary material for: PRMT5-mediated arginine methylation of FXR1 is essential for RNA binding in cancer cells
Source: Nucleic Acids Res. 2024 May 6;52(12):7225–44. doi: 10.1093/nar/gkae319 (PMC11229354; doi:10.1093/nar/gkae319)
Supplement: gkae319_Supplemental_Files [file gkae319_supplemental_files.zip › Supplemental data-low size.pdf]

Table S1. Primers used in the study.

**Primer**	**Sequence (5'-3')**
FXR1-F	CCCTAATTACACCTCCGGTTATG
FXR1-R	TCTCCTGCCAATGACCAATC
P21-F	CGGAACAAGGAGTCAGACATT
P21-R	AGTGCCAGGAAAGACAACTAC
GAPDH-F	GGTGGTCTCCTCTGACTTCAACA
GAPDH-R	GTTGCTGTAGCCAAATTCGTTGT
Beta Actin-F	GGACCTGACTGACTACCTCAT
Beta Actin-R	CGTAGCACAGCTTCTCCTTAAT
RPS18-F	CTTTGCCATCACTGCCATTAAG
RPS18-R	ATCACACGTTCCACCTCATC
MycFXR1-R386K-F	CTAGGTCTTATAGCGGAAAAGGCAGAGGTCGTCGGGGA
MycFXR1-R386K-R	TCCCCGACGACCTCTGCCTTTTCCGCTATAAGACCTAG
MycFXR1-R388K-F	CTTATAGCGGAAGAGGCAAAGGTCGTCGGGGACCTAA
MycFXR1-R388K-R	TTAGGTCCCCGACGACCTTTGCCTCTTCCGCTATAAG
MycFXR1-R386/388K-F	GGTTCTAGGTCTTATAGCGGAAAAGGCAAAGGTCGTCGGGGACCTAATTAC
MycFXR1-R386/388K-R	GTAATTAGGTCCCCGACGACCTTTGCCTTTTCCGCTATAAGACCTAGAACC
MycFXR1-R453K-F	GAAGTGTTTCAGGGGGTAAAGGTCGTGGTGGACCACGT
MycFXR1-R453K-R	ACGTGGTCCACCACGACCTTTACCCCCTGAAACACTTC
MycFXR1-R455K-F	AGTGTTTCAGGGGGTCGAGGTAAAGGTGGACCACGTGGTGGCAAA
MycFXR1-R455K-R	TTTGCCACCACGTGGTCCACCTTTACCTCGACCCCCTGAAACACT

MycFXR1-R459K-F	GGTCGAGGTCGTGGTGGACCAAAAGGTGGCAAATCCTCCATCAGT
MycFXR1-R459K-R	ACTGATGGAGGATTTGCCACCTTTTGGTCCACCACGACCTCGACC
MycFXR1-R453/455/459K-F	CAGAAGTGTTTCAGGGGGTAAAGGTAAAGGTGGACCAAAAGGTGGCAAATCCTCCATCAG
MycFXR1-R453/455/459K-R	CTGATGGAGGATTTGCCACCTTTTGGTCCACCTTTACCTTTACCCCCTGAAACACTTCTG
pET28a-FXR1t-F	TCG CGG ATC CGA ATT CAT GTC TTA TAG CGG AAG AGG CAG AG
pET28a-FXR1t-R	GTG CGG CCG CAA GCT TTG ATT ATG GAT TGC TGT CTG GAT CTT TGA G
pET28aFXR1t-R386K-F	TCA TGT CTT ATA GCG GAA AAG GCA GAG GTC GTC GGG G
pET28aFXR1t-R386K-R	CCC CGA CGA CCT CTG CCT TTT CCG CTA TAA GAC ATG A
pGEXFXR1t-R386-388K-F	GAA TTC ATG TCT TAT AGC GGA AAA GGC AAA GGT CGT CGG GGA CCT AAT
pGEXFXR1t-R386-388K-R	ATT AGG TCC CCG ACG ACC TTT GCC TTT TCC GCT ATA AGA CAT GAA TTC
MAP1B- F	CCCTCAGGCATCCACATATTC
MAP1B- R	CAAGAGGACACGAGGCATAAA
KMT2A- F	CTCCTCTCTTCCCTTGGTTTAC
KMT2A- R	CTCTTGTCAGCATCTCGATCTT
ZNF106- F	CACGAGAACGAAGGAACAGTAG
ZNF106-R	GATGGAGACACATGGGAAGATG
MYO5A-F	GAGAGCTGTGGCTGGTTTAT
MYO5A-R	CTCATTCTCATGGCTCCTCTTC
RNF213-F	GCTACGCATCCCTGCTATTT
RNF213-R	AGGTACTGGTCCTTATCCAGAG
PRMT5-F	TATGTGGTACGGCTGCACA3

PRMT5-R	TGGCTGAAGGTGAAACAGG
SMG1-F	TATGGTCGGAAGTCGTTGGG
SMG1-R	TTGGTGGCTAAAGCACGACT
DYNC1H1-F	AGAAGACCAAGCCTGTCACG
DYNC1H1-R	CCTTGGCCTTTGCACACTTC
PRKDC-F	AGCCATTGCCAGAGTACCAC
PRKDC-R	GGATCACTGGAGGTCATGGG
AHNAK-F	GGGAGCGATGATGAGACAGG
AHNAK-R	AAACTGACAGCTCCACCTCG
HUWE1-F	GGAGAGCTAGCCGCATCTTC
HUWE1-R	TAACCCACTCAGGTCAGGCT
UBR4-F	CATCAGCTCCAGCCTCAGAC
UBR4-R	CAGGAGAGGTCCGAAGGGTA
AHNAK2-F	GTAGCTTCCTTGTGTCCGGC
AHNAK2-R	CCCTTCAGTCACAGAGTGGTC

Sup Fig 1

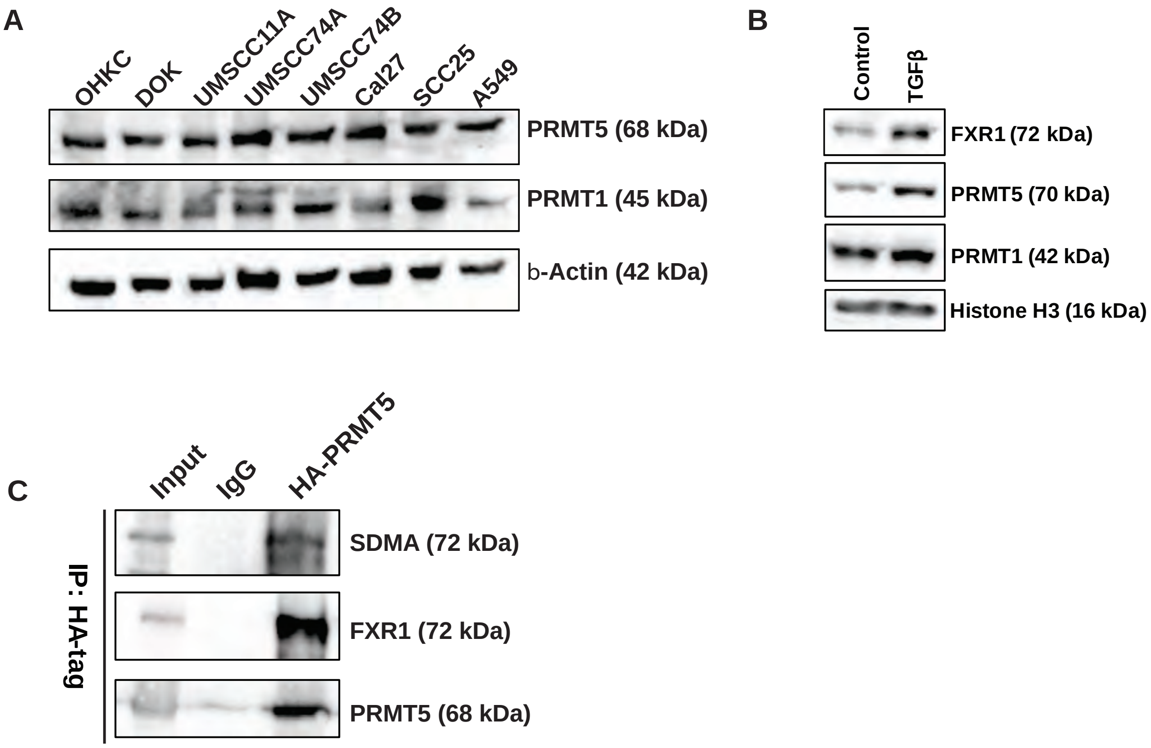
A
OHKC
DOK
UMSCC11A
UMSCC74A
UMSCC74B
Cal27
SCC25
A549
PRMT5 (68 kDa)
PRMT1 (45 kDa)
b-Actin (42 kDa)
B
Control
TGFβ
FXR1 (72 kDa)
PRMT5 (70 kDa)
PRMT1 (42 kDa)
Histone H3 (16 kDa)
C
Input
IgG
HA-PRMT5
IP: HA-tag
SDMA (72 kDa)
FXR1 (72 kDa)
PRMT5 (68 kDa)

Sup Fig 2

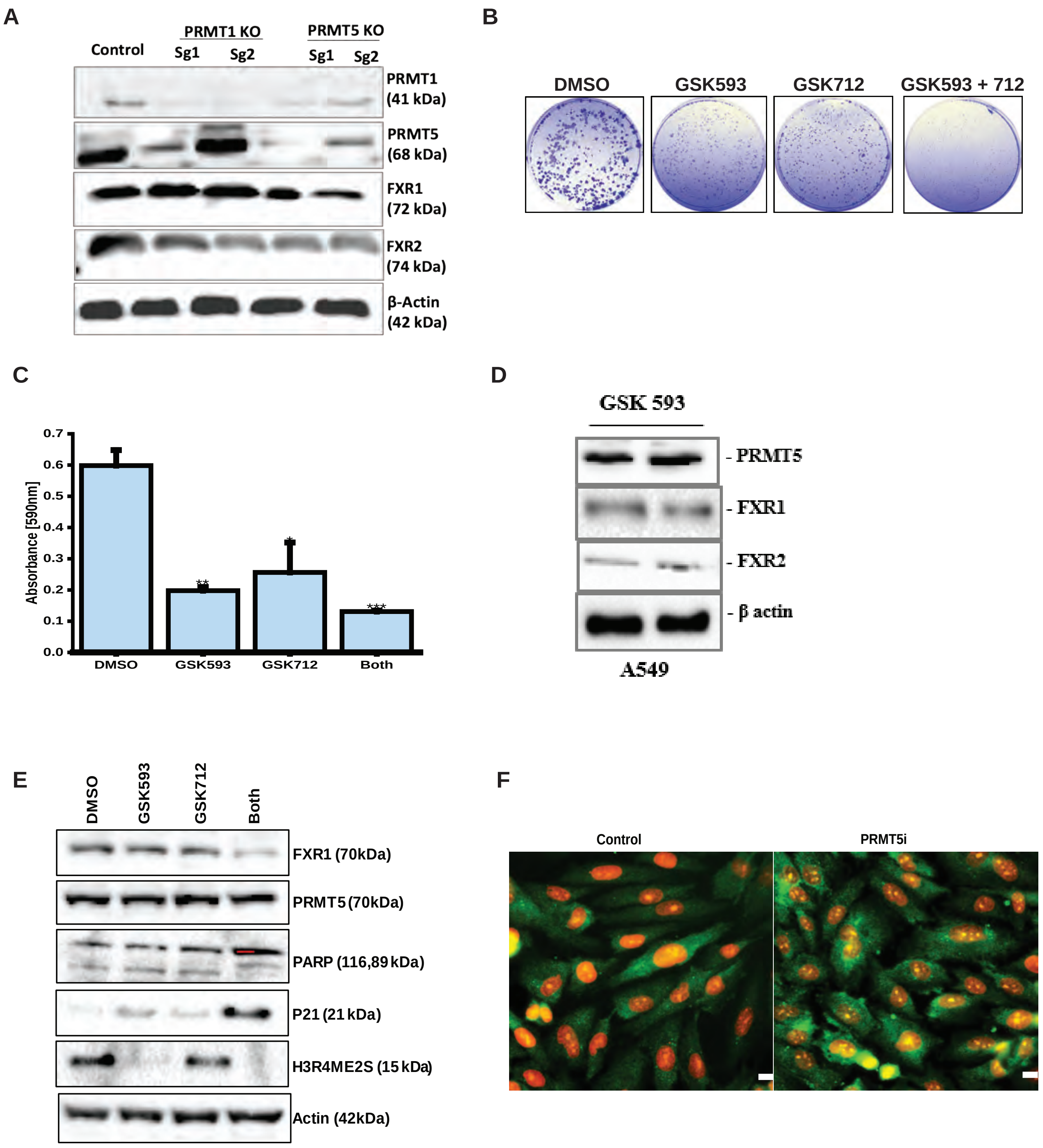

A
Control
PRMT1 KO
Sg1
Sg2
PRMT5 KO
Sg1
Sg2
PRMT1 (41 kDa)
PRMT5 (68 kDa)
FXR1 (72 kDa)
FXR2 (74 kDa)
β-Actin (42 kDa)
B
DMSO
GSK593
GSK712
GSK593 + 712
C
Absorbance [590nm]
0.7
0.6
0.5
0.4
0.3
0.2
0.1
0.0
DMSO
GSK593
GSK712
Both
D
GSK 593
- PRMT5
- FXR1
- FXR2
- β actin
A549
E
DMSO
GSK593
GSK712
Both
FXR1 (70kDa)
PRMT5 (70kDa)
PARP (116,89 kDa)
P21 (21 kDa)
H3R4ME2S (15 kDa)
Actin (42kDa)
F
Control
PRMT5i

Sup Fig 3

A

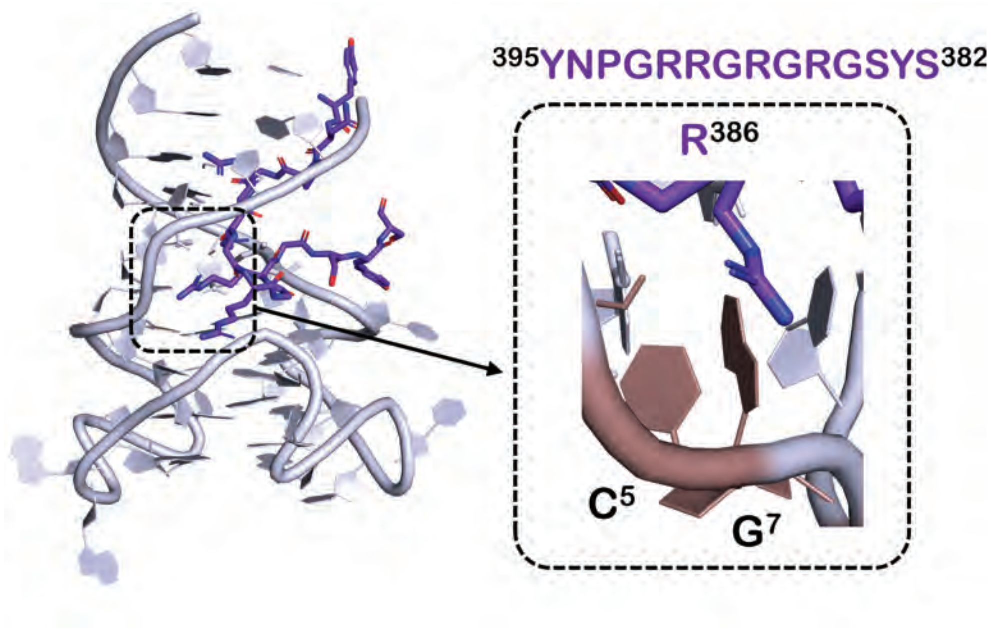
395YNPGRRGRGRGSYS382
R386
C5
G7

B

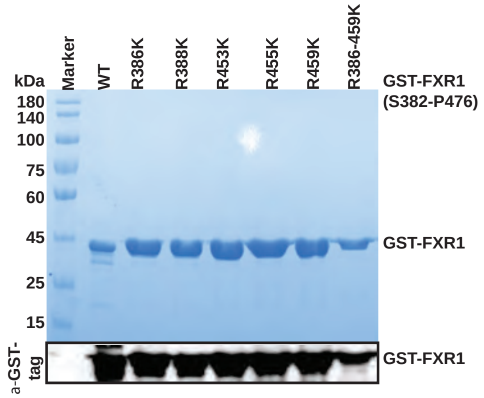
Marker
WT
R386K
R388K
R453K
R455K
R459K
R386-459K
kDa
180
140
100
75
60
45
25
15
GST-FXR1 (S382-P476)
GST-FXR1
a-GST-tag
GST-FXR1

Sup Fig 4

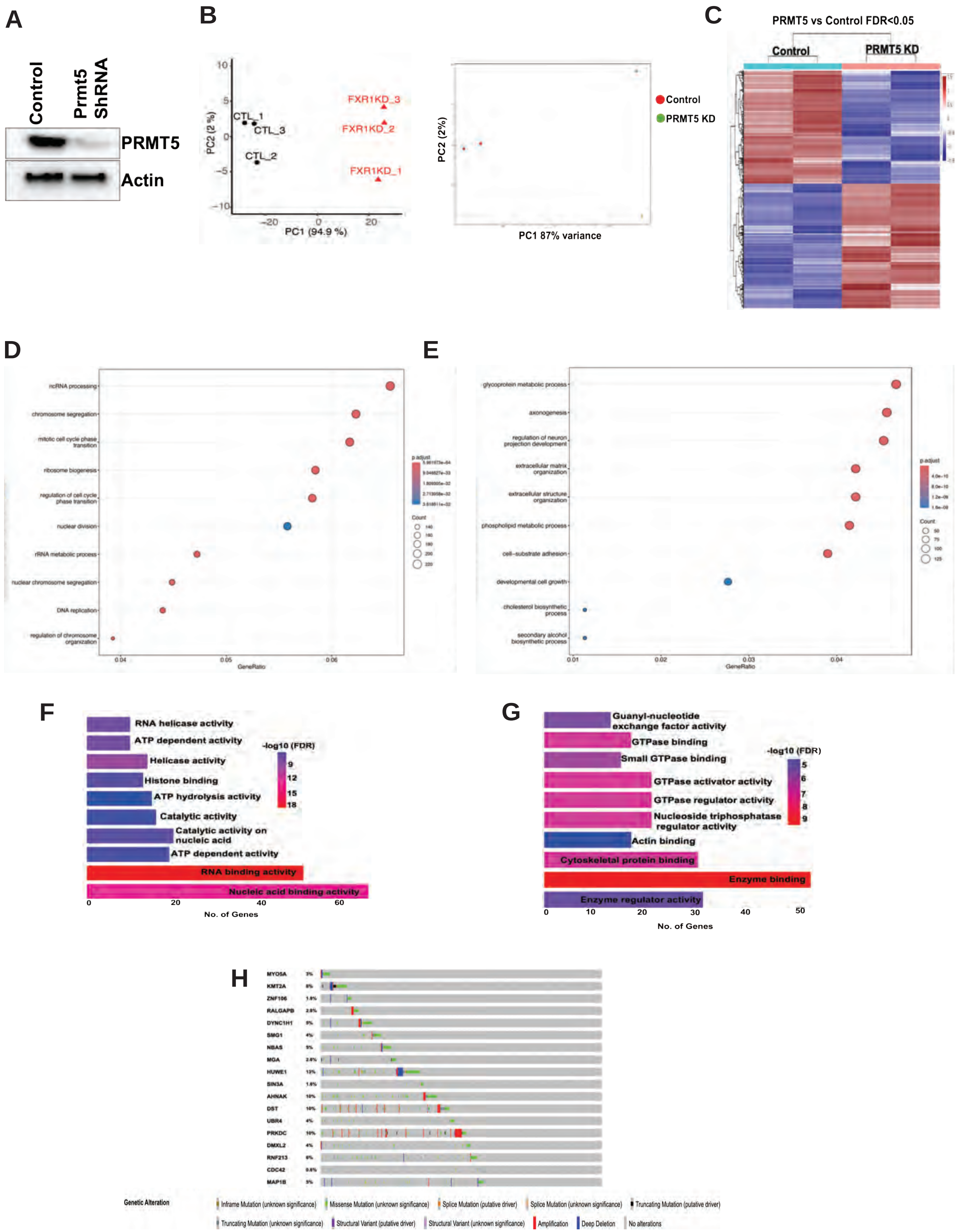
A
Control
Prmt5 ShRNA
PRMT5
Actin
B
CTL_1
CTL_3
CTL_2
FXR1KD_3
FXR1KD_2
FXR1KD_1
PC2 (2 %)
PC1 (94.9 %)
PC2 (2%)
PC1 87% variance
Control
PRMT5 KD
C
PRMT5 vs Control FDR<0.05
Control
PRMT5 KD
D
ncRNA processing
chromosome segregation
mitotic cell cycle phase transition
ribosome biogenesis
regulation of cell cycle phase transition
nuclear division
rRNA metabolic process
nuclear chromosome segregation
DNA replication
regulation of chromosome organization
GeneRatio
p.adjust
Count
E
glycoprotein metabolic process
axonogenesis
regulation of neuron projection development
extracellular matrix organization
extracellular structure organization
phospholipid metabolic process
cell−substrate adhesion
developmental cell growth
cholesterol biosynthetic process
secondary alcohol biosynthetic process
GeneRatio
p.adjust
Count
F
RNA helicase activity
ATP dependent activity
Helicase activity
Histone binding
ATP hydrolysis activity
Catalytic activity
Catalytic activity on nucleic acid
ATP dependent activity
RNA binding activity
Nucleic acid binding activity
-log10 (FDR)
No. of Genes
G
Guanyl-nucleotide exchange factor activity
GTPase binding
Small GTPase binding
GTPase activator activity
GTPase regulator activity
Nucleoside triphosphatase regulator activity
Actin binding
Cytoskeletal protein binding
Enzyme binding
Enzyme regulator activity
-log10 (FDR)
No. of Genes
H
MYO5A
KMT2A
ZNF106
RALGAPB
DYNC1H1
SMG1
NBAS
MGA
HUWE1
SIN3A
AHNAK
DST
UBR4
PRKDC
DMXL2
RNF213
CDC42
MAP1B
Genetic Alteration
Inframe Mutation (unknown significance)
Missense Mutation (unknown significance)
Splice Mutation (putative driver)
Splice Mutation (unknown significance)
Truncating Mutation (putative driver)
Truncating Mutation (unknown significance)
Structural Variant (putative driver)
Structural Variant (unknown significance)
Amplification
Deep Deletion
No alterations

Sup Fig 5

A HNSCC

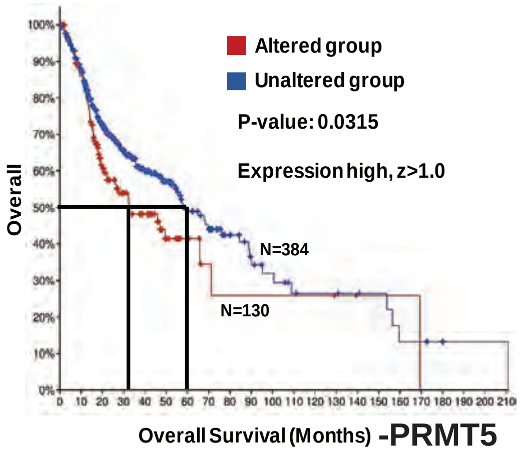

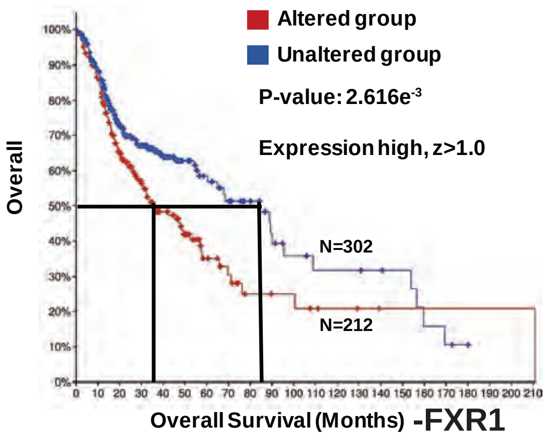

Lung Adenocarcinoma

B

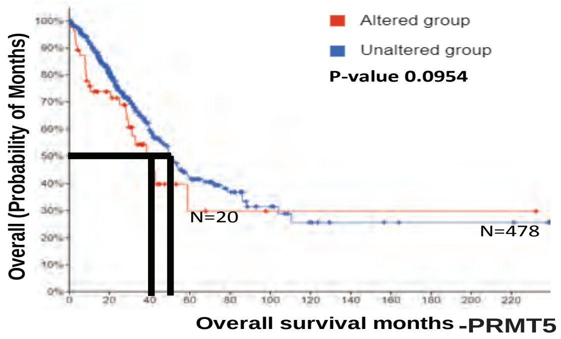

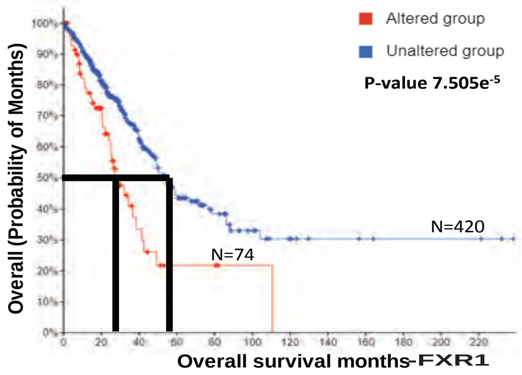

Suppl. Data 3

AHNAK2

00001 AGTTGGGATC TTCGCTTCTG GGCCAGTTGG GAGAGCGTCT GTAGCTTCCT TGTGTCCGGC CGTCAGCTGC AGCCCGGGGA GCCAGGTGCA GAAACGGAAG
000101 ATGACCACTC TGTGACTGAA GGGCCTGCGG ATGAGGGCAT TCGACCACGG CCGCAGGGGT CTTCACCTGT CTACGAATAC ACGACTGAGG CTGCCGACTT
000201 TGGACTCCAG GAAGACGCCC CCGGCAGGCA AGGTTCTGCT GGGAGACGGA GATCCTGGTG GAAGCGAGAT TCAGGGGACT CACGGACATT TTTCAGGATG
000301 AGTCGTCCAG AGGCTGTCCA GGAGGCAACA GAGGTGACGC TGAAGACAGA GGTGGAGGCA GGAGCCAGTG GCTACAGTGT CACAGGTGGT GGGGACCAGG
000401 GGATCTTCGT CAAGCAAGTG CTGAAGGACT CCTCAGCCGC CAAGCTTTTT AACTTGAGAG AAGGGGATCA GCTGCTCAGT ACAACCGTGT TCTTTGAAAA
000501 CATAAAATAT GAAGATGCTC TCAAAATCCT TCAATATTCA GAGCCGTACA AGGTTCAGTT CAAAATCAGA CGGCAGCTCC CTGCCCCACA GGATGAAGAG
000601 TGGGCTTCCA GCGATGCCCA GCACGGCCCA CAGGGCAAGG AGAAGGAGGA CACGGATGTT GCTGATGGGT GCAGAGAGAC CCCCACGAAA ACTCTGGAAG
000701 GAGATGGGGA CCAAGAGAGA CTCATCTCCA AACCAAGGGT GGGGAGAGGC AGGCAGAGCC AGAGGGAGAG GCTCTCTTGG CCAAAATTTC AATCCATAAA
000801 GAGCAAGCGG GGGCCGGGAC CCCAGAGGTC ACACAGCTCG TCAGAGGCCT ACGAACCTAG GGACGCACAT GACGTGTCCC CTACAAGCAC AGACACAGAG
000901 GCCCAGCTCA CGGTGGAGCG CCAAGAGCAG AAGGCAGGGC CGGGCAGCCA GAGGAGGCGG AAGTTCCTCA ACCTCAGATT CAGGACAGGC TCGGGACAGG
001001 GCCCTTCATC GACAGGACAG CCAGGCAGGG GGTTCCAGAG TGGGGTGGGC CGTGCTGGGG TCCTGGAAGA GTTGGGGCCC TGGGGTGATA GCCTCGAGGA
001101 GACTGGGGCT GCCACAGGCA GCAGGAGAGA GGAGAGGGCA GAACAGGATC GAGAAGTGAT GCCTGCTCAG AGCATGCCAT TGCCCACAGA GCTCGGTGAC
001201 CCTAGACTTT GCGAGGGAAC CCCTCAGGAA GGGGGACTCA GGGCAGCCAG GCTCCATGGA AAGACCCTGG AGGGGCAGGC ACAGGAGACA GCAGTGGCCC
001301 AGAGGAAGCC CAGGGCCCAG CCAACTCCTG GAATGAGCCG GGAGGGTGAA GGCGAGGGAC TGCAGAGCCT GGAAATCGGG ATCGCCAGAC TGTCCTTGAG
001401 AGACACAACC GAAGGAGGCA CACAGATTGG CCCACCAGAA ATTAGGGTGC GAGTACACGA TTTAAAGACA CCAAAATTTG CATTTTCCAC AGAAAAAGAG
001501 CCAGAAAGAG AAAGGCGCCT TAGTACCCCA CAGCGAGGGA AGAGACAGGA TGCGTCCTCA AAAGCGGGTA CTGGCCTGAA GGGTGAGGAG GTGGAAGGAG
001601 CCGGGTGGAT GCCGGGCAGG GAACCAACCA CACATGCAGA AGCACAGGGG GATGAAGGAG ATGGAGAGGA AGGACTACAG AGGACAAGGA TCACTGAGGA
001701 ACAGGACAAG GGCAGGGAAG ACACAGAAGG ACAGATAAGA ATGCCCAAGT TCAAGATACC CTCCTTAGGA TGGTCGCCAA GCAAGCACAC AAAGACAGGC
001801 AGAGAAAAAG CCACAGAAGA CACAGAGCAG GGAAGGGAAG GAGAGGCCAC AGCAACAGCT GATAGAAGAG AACAGAGACG CACAGAGGAA GGATTAAAAG
001901 ACAAAGAAGA CAGTGACTCA ATGACAAACA CAACAAAAAT ACAACTAATA CACGATGAAA AACGCTTAAA AAAGGAACAA ATTCTGACAG AAAAGGAAGT
002001 GGCCACCAAA GACAGCAAGT TCAAAATGCC CAAGTTCAAG ATGCCATTGT TCGGGGCGTC AGCCCCAGGC AAGTCCATGG AGGCCTCGGT GGATGTGTCT
002101 GCGCCGAAGG TGGAGGCCGA CGTGAGCCTC CTCTCCATGC AGGGGGACCT CAAGACCACT GACCTCAGCG TCCAGACCCC TTCCGCTGAC CTGGAGGTCC
002201 AGGATGGCCA AGTGGATGTG AAACTTCCGG AGGGCCCCCT GCCCGAGGGA GCCAGCCTCA AAGGGCACCT GCCCAAGGTG CAGAGGCCCA GTTTGAAGAT
002301 GCCCAAAGTG GACCTCAAGG GCCCCAAGCT GGACCTGAAA GGCCCCAAGG CGGAAGTGAC AGCCCCCGAT GTGAAGATGT CTCTGTCCAG CATGGAGGTG
002401 GACGTCCAGG CCCCGAGAGC AAAGCTGGAT GGTGCGCGGC TGGAGGGGGA CCTGTCCCTG GCCGACAAGG AGGTGACTGC CAAAGACAGC AAGTTCAAAA
002501 TGCCCAAGTT CAAGATGCCA TCATTCGGGG TGTCGGCCCC AGGCAAGTCC ATGGAGGACT CGGTGGATGT GTCTGCGCCG AAGGTGGAGG CCGACGTGAG
002601 CCTCTCCTCC ATGCAGGGGG ACCTCAAGGC CACTGACCTC AGCATTCAGC CCCCTTCCGC TGACCTGGAG GTCCAGGCTG GCCAAGTGGA TGTGAAACTT
002701 CCGGAGGGCC CTGTGCCCGA GGGAGCCGGC CCCAAAGTGC ACCTGCCCAA AGTGGAGATG CCCAGTTTCA AGATGCCCAA AGTGGACCTC AAGGGCCCCC
002801 AGATAGATGT TAAGGGCCCC AAGCTGGACC TGAAAGGCCC CAAGGCGGAA GTGACAGCCC CCGATGGCGA GGTGTCTCTG CCCAGCATGG AGGTGGATGT
002901 CCAGGCCCAG AAGGCCAAGC TGGATGGTGC GTGGCTGGAG GGGGACCTGT CCCTGGCCGA CAAGGACGTG ACTGCCAAAG ACAGCAAGTT CAAAATGCCC
003001 AAGTTCAAGA TGCCGTCGTT CGGGGTATCG GCCCCAGGGA AGTCCATCAA GGCCTTGGTG GATGTGTCTG CACCCAAGGT GGAGGCCGAC CTGAGTCTCC
003101 CCTCCATGCA GGGGGACCTG AAGACCACTG ACCTCAGCAT TCAGCCTGCT TCTACTGACC TGAAGGTCCA GGCTGACCAG GTGGATGTGA AGCTCCCGGA
003201 GGGCCACCTG CCCGAGGGAG CTGGCCTTAA AGGGCACTTG CCCAAGGTGG AGATGCCCAG TTTCAAGATG CCCAAAGTGG CCCTCAAGGG CCCCCAGGTG
003301 GACGTCAAGG GCCCCAAGCT GGACCTGAAA AGCCCCAAGG CGGAAGTCAC AGCCCCTGAT GTGGAGGTGT CTCTGCCCAG CGTGGAGGTG GACGTCGAGG
003401 CCCCGGGAGC CAAGCTGGAC AGTGCGCGGC TGGAGGGGGA ACTGTCCCTG GCCGACAAGG ATGTGACTGC CAAAGACAGC AGGTTCAAAA TGCCCAAGTT
003501 CAAGATGCCA TCGTTCGGGG CGTCAGCCCC AGGCAAGTCC ATCGAGGCCT CGGTGGATGT GTCTGCACCC AAAGTGGAGG CCGACGTGAG TCTCCCCTCC
003601 ATGCAGGGGG ACCTCAAGAC CACTGACCTC AGCATTCAGC CCCCTTCCGC TGACCTGGAG GTCCACGCTG GCCAGGTGGA CGTGAAGCTC CTGGAGGGCC
003701 ACGTGCCTGA GGGAGCCGGC TTCAAAGGGC ACCTGCCCAA GGTGCAGATG CCTAGTTTGA AGATGCCCAA AGTGGACCTC AAGGGCCCCC AGGTGGAAGT
003801 CAGGGGCCCC AAGCTGGACC TGAAAGGTCA TAAGGCAGAG GTGACGGCCC ACGAAGTGGC TGTGTCTCTG CCCAGTGTGG AGGTGGACAT GCAGGCCCCG
003901 GGAGCCAAGT TGGATGGCGC ACAGCTGGAC GGGGACCTGT CCCTGGCTGA CAAGGACGTG ACTGCCAAAG ACAGCAAGTT CAAAATGCCC AAGTTCAAGA
004001 TGCCGTCGTT CGGGGTGTCT GCCCCAGGCA AGTCCATTGA GGCCTCCGTG GACCTGTCTG CACCCAAGGT GGAGGCCGAC ATGAGCCTCC CCTCCATGCA
004101 GGGGGACCTC AAGACCACTG ACCTCAGCAT TCAGCCCCCT TCCACTGACC TGGAGCTCCA GGCTGGCCAA TTGGACGTGA AACTCCCAGA GGGCCCCGTG
004201 CCCGAGGGAG CCGGCCTCAA AGGGCACCTG CCCAAGCTGC AGATGCCCAG TTTCAAGGTG CCCAAAGTGG ACCTCAAGGG CCCTGAAATA GACATCAAGG
004301 GCCCCAAGCT GGACCTAAAA GACCCCAAGG TGGAAGTGAC AGCCCCTGAT GTGGAGGTTT CTCTGCCCAG CGTGGAGGTG GATGTCGAGG CCCCAGGAGC
004401 CAAGCTGGAT GGTGGACGGC TGGAGGAGGA CATGTCCCTG GCCGACAAGG ACTTGACTAC CAAAGACAGC AAGTTCAAAA TGCCCAAGTT CAAGATGCCG
004501 TCGTTCGGGG TGTCTGCCCC AGGCAAGTCC ATCGAGGCCT CAGTGGATGT GTCTGCGCCG AAGGTGGAGG CCGACGTGAG CCTCCCCTCC ATGCAGGGGG
004601 ACCTCAAGGC CACTGACCTG AGCATACAGC CCCCTTCTGC TGACCTGGAG GTCCAGGCTG GCCAAGTGGA CGTGAAACTC CCAGAGGGCC CTGTGTCCGA
004701 GGGAGCCGGC CTCAAAGGGC ACCTGCCCAA AGTGCAGATG CCCAGTTTCA AGATGCCCAA AGTGGACCTC AAGGGGCCCC AGATAGATGT TAAGGGCCCC
004801 AAGCTGGACC TGAAAGGCCC CAAGGTGGAA GTGACAGCCC CCGATGTGAA GATGTCTCTG TCCAGCATGG AGGTGGACGT CCAGGCCCCG AGAGCAAAGC
004901 TGGATGGTGC GCAGCTGGAG GGGGACCTGT CCCTGGCCGA CAAGGCGGTG ACTGCCAAAG ACAGCAAGTT CAAAATGCCC AAGTTCAAGA TGCCATCATT
005001 TGGGGTGTCG GCCCCAGGCA AGTCCATCGA GGCCTCGGTG GATGTGTCTG AGCCGAAGGT GGAAGCTGAT GTGAGCCTCC CCTCCATGCA GGGGGACCTG
005101 AAGACCACTG ACCTCAGCAT TCAGTCCCCT TCCGCCGACC TGGAGGTCCA GGCTGGCCAA GTGAACGTGA AACTCCCGGA GGGCCCCCTT CCCGAGGGAG
005201 CCGGCTTCAA AGGGCACCTC CCCAAGGTGC AGATGCCCAG TTTGAAGATG CCCAAAGTGG CCCTCAAGGG CCCCCAGATG GACGTCAAGG GCCCCAAGCT
005301 GGACCTGAAA GGCCCCAAGG CGGAGGTGAT GGCCCCCGAC GTGGAGGTGT CTCTGCCCAG CGTGGAGGTG GACGTCGAGG CTCCAGGAGC CAAGCTGGAC
005401 AGTGTGCGGC TGGAGGGTGA CCTGTCCCTG GCCGACAAGG ATGTGACTGC CAAAGACAGC AAGTTCAAAA TGCCCAAGTT CAAGATGCCG TCGTTCGGGG
005501 TGTCTGCCCC AGGCAAGTCC ATCGAGGCCT CGGTGGATGT GTCTGCGCCG AAGGTGGAGG CCGAAGTGAG CCTCCCCTCC ATGCAGGGGG ACCTCAAGAC
005601 CACGGACCTC TGCATTCCGC TCCCTTCTGC AGACCTGGTG GTCCAGGCTG GCCAAGTGGA CATGAAGCTC CCGGAGGGCC AGGTGCCCGA GGGAGCCGGC
005701 CTCAAAGGGC ACTTGCCCAA GGTGGATATG CCCAGTTTCA AGATGCCCAA AGTGGACCTC AAGGGCCCCC AGACAGATGT TAAGGGCGCC AAGCTGGACC
005801 TGAAAGGCCC CAAGGCGGAA GTGACAGCCC CCGATGTCGA GGTGTCTCTG CCCAGCATGG AGGTGGATGT CCAGGCCCAG AAGGCTAAGC TGGATGGTGC
005901 GCGGCTGGAG GGAGACCTGT CCCTGGCCGA CAAGGACATG ACTGCCAAAG ACAGCAAGTT CAAAATGCCC AAATTCAAGA TGCCGTCGTT CGGGGTATCG
006001 GCCCCAGGGA GGTCCATCGA GGCCTCGGTG GATGTGCCTG CACCCAAGGT GGAGGCCGAC GTGAGTCTCC CCTCCATGCA GGGGGACCTG AAGACCACTG
006101 ACCTCAGCAT TCAGCCCCCT TCTGCCGACC TGAAGGTCCA GACTGGCCAG GTGGATGTGA AGCTCCCGGA GGGCCACGTG CCCGAGGGAG CTGGCCTCAA
006201 AGGGCACCTG CCCAAGGTGG AGATGCCCAG TTTGAAGATG CCCAAAGTGG ACCTCAAGGG CCCCCAGGTG GACATCAAGG GCCCCAAACT GGACCTAAAA
006301 GACCCCAAGG TGGAAATGAG AGTCCCCGAT GTCGAGGTGT CTCTGCCCAG CATGGAGGTG GACGTCCAGG CCCCAAGAGC CAAGCTGGAT AGTGCGCATC
006401 TGCAGGGGGA CCTGACCCTG GCCAACAAGG ACCTGACTAC CAAAGACAGC AAGTTCAAAA TGCCCAAGTT CAAGATGCCG TCGTTTGGGG TGTCTGCCCC
006501 AGGCAAGTCC ATCGAGGCCT CGGTGGATGT GTCTCCACCC AAGGTGGAGG CCGACATGAG TCTCCCCTCC ATGCAGGGGG ACCTCAAGAC CACTGACCTC
006601 AGCATTCAGC CCCTTTCCGC CGACGTGAAG GTCCAGGCTG GCCAGGTGGA CGTGAAACTC CTGGAGGGCC CTGTGCCCGA GGAAGTCGGC CTCAAAGGGC
006701 ACCTGCCCAA GCTGCAGATG CCCAGTTTCA AGGTGCCCAA AGTGGACCTC AAGGGCCCCG AAATAGACAT CAAGGGCCCC AAGCTGGACC TAAAAGACCC
006801 CAAGGTGGAA GTGACAGCCC CTGATGTGGA GGTGTCTCTG CCCAGCGTGG AGGTGGACGT CAAGGCCCCA GGAGCCAAGC TGGATGGTGC GCGGCTGGAG
006901 GGGGACATGT CCCTGGCCGA CAAGGACGTG ACTGCCAAAG ACAGCAAGTT CAAAATGCCC AAGTTCAAGA TGCTGTCGTT TGGGGTGTCT GCCCTTGGCA
007001 AGTCCATCGA GGCCTCAGCG GATGTGTCTG CGTTGAAGGT GGAGGCCGAC GTGAGCCTCC CCTCCATGCA GGGGGACCTC AAGACCACTG ACCTCAGCGT
007101 TCAGCCCCCT TCCGCTGACC TGGAGGTCCA GGCTGGCCAA GTGGATGTGA AACTCCCAGA GGGCCCCGTG CCGGAGGGAG CCGGCCTCAA AGGGCACCTG
007201 CCCAAGCTGC AGATGCCCAG TTTCAAGATG CCCAAAGTAG ATCTCAAGGG CCCCCAGATA GATGTCAAGG GCCCCAAGCT GGACCTGAAA GGCCCCAAGA
007301 CGGACGTGAT GGCCCCCGAC GTGGAGGTGT CTCAGCCCAG CGTGGAGGTG GATGTCGAGG CCCCGGGAGC CAAGCTGGAT GGTGCGTGGC TGGAGGGGGA
007401 CCTGTCTGTG GCGGACAAGG ATGTGACTAC CAAAGACAGC AGGTTCAAAA TTCCCAAGTT CAAGATGCCG TCATTCGGGG TGTCTGCCCC AGGCAAGTCC
007501 ATCGAGGCCT CGGTGGATGT GTCTGCGCCG AAGGTGGAGG CCGACGGGAG CCTCTCCTCC ATGCAGGGGG ACCTCAAGGC CACTGACCTC AGCATTCAGC
007601 CCCCTTCCGC TGACCTGGAG GTCCAGGCTG GCCAAGTGGA CGTGAAACTC CCAGAGGGCC CTGTGCCGGA GGGAGCCGGC CTCAAAGGGC ACCTGCCCAA
007701 GGTGCAGATG CCCAGTTTCA AGATGCCTGA AATGGACCTC AAGGGCCCCC AGCTAGATGT CAAGGGCCCC AAGCTGGACC TGAAAGGCCC CAAGGCGGAA
007801 GTGACAGCCC CCGATGTGGA GATGTCTCTG TCCAGCATGG AGGTGGACGT CCAGGCCCCG AGAGCAAAGC TGGATGGTGC GCGGCTGGAG GGGGACCTGT
007901 CCCTGGCCGA CAAGGGTGTG ACAGCCAAAG ATAGCAAGTT CAAAATGCCC AAGTTCAAGA TGCCATCATT CAGGGTGTCG GCCCCAGGCG AGTCCATCGA
008001 GGCGTTGGTG GATGTGTCTG AGCTGAAGGT GGAAGCCGAC ATGAGCCTCC CCTCCATGCA AGGGGACCTT AAGACCACTG ACATCAGCAT TCAGCCCCCC
008101 TCTGCCCAAC TGGAGGTCCA GGCTGGCCAG GTGGATGTGA AACTCCCAGA GGGCCACGTT CCCGAGGGAG CCGGCCTCAA AGGGCACCTG CCCAAGCTGC
008201 AGATGCCCAG TTTCAAGATG CCTGAAGTGG ACCTCAAGGG CCCCCAGATA GATGTTAAGG GCCCCAACGT GGACCTGAAA GGCCCCAAGG CGGAAGTGAC
008301 AGCCCCCGAT GTGAAGATGT CTCTGTCCAG CATGGAGGTG GACGTCCAGG CCCCGAGAGC AAAGCTGGAT GGTGCGCGGC TGGAGGGGGA CCTGTCCCTG

```
008401 GCCGACAAGG GCATGACAGC CAAAGACAGC AAGTTCAAAA TGCCCAAGTT CAAGATGCCG TCATTCGGGG TGTCGGCCCC AGGCAAGTCC ATCGAGGCCT
008501 CGGTGGATGT GTCTGAGCTG AAGGTGGAAG CTGACGGGAG CTTCCCCTCC ATGCAAGGGG ATCTTAAGAC CACTGACATC CGCATTCAGC CCCCCTCCGC
008601 CCAACTGGAG GTCCAGGCTG GCCAGGTGGA CGTGAAACTC CCAGAGGGCC ACGTTCCCGA GGGAGCCGGC CTCAAAGGGC ACCTGCCCAA GGTGCAGATG
008701 CCCAGTTTCA AGATGCCCAA AGTGGATCTC AAGGGCCCCC AGATAGACGT CAAGGGCCCC AAGCTGGACC TGAAAGGCCC CAAGGCGGAG GTGACGGCCC
008801 CCGACGTGGA GGTGTCTCTG CCCAGCGTGG AGGTGGACGT CGAGGCCCCG AGAGCAAAGC TGGATGGTGC ACGGCTGGAG GGTGACCTGT CCCTGGCCGA
008901 CAAGGATGTG ACTGCCAAAG ACAGCAAGTT CAAAATGCCC AAGTTCAAGA TGCCGTCGTT CGGGGTGTCT GCCCCAGGCA AGTCCATTGA GGTCTCGGTG
009001 GATGTGTCTG CGCCGAAGGT GGAGGCCGAA GTGAGCCTCC CCTCCATGCA GGGGGACCTG AAGACCACTG ACATCAGCAT TGAGCCCCCC TCTGCCCAAC
009101 TGGAGGTCCA GGCTGGCCAG GTGGACCTGA AGCTCCCAGA GGGCCACGTT CCCGAGGGAG CTGGCCTCAA AGGGCACCTG CCCAAGTTGC AGATGCCCAG
009201 TTTCAAGATG CCCAAAGTAG ATCGCAAGGG ACCCCAGATA GATGTCAAGG GCCCCAAGCT GGACCTGAAA GGCCCGAAGA CGGACGTGAC GGCCCCCGAC
009301 GTGGAGGTGT CTCAGCCCGG CATGGAGGTG GATGTCGAGG CCCCAGGAGC CAAGTTGGAT GGTGCACGGC TGGAGGGGGA CCTGTCCCTG GCCGACAAGG
009401 ATGTGACTGC CAAAGACAGC AAGTTCAAAA TGCCCAAGTT CAAGATGCCG TCGTTCGGGG TGTCTGCCCC AGGCAAGTCC ATTGAGGTCT TGGTGGATGT
009501 GTCTGCGCCA AAGGTGGAGG CCGACCTGAG CCTCCCCTCC ATGCAGGGGG ACCTGAAGAA CACTGACATC AGCATTGAGC CCCCCTCTGC CCAACTGGAG
009601 GTCCAGGCTG GCCAGGTGGA CGTGAAGCTC CCAGAGGGCC ACGTTCTCGA GGGAGCTGGC CTCAAAGGGC ACCTGCCCAA GTTGCAGATG CCCAGTTTCA
009701 AGATGCCCAA AGTAGATCGC AAGGGCCCCC AGATAGACAT CAAGGGCCCC AAGCTGGACC TGAAAGGCCC GAAGATGGAT GTGACGGCCC CCGACGTGGA
009801 GGTGTCTCAG CCCAGCATGG AGGTGGACGT CGAGGCCCCA GGAGCCAAGT TGGATGGTGC ACGGCTGGAG GGGGACCTGT CCCTGGCCGA CAAGGATGTG
009901 ACTGCCAAAG ACAGCAAGTT CAAAATGCCC AAATTCAAGA TGCCGTCGTA CAGGGCGTCT GCCCCAGGCA AGTCCATCCA GGCCTCGGTG GATGTGTCTG
010001 CGCCGAAGGC GGAGGCCGAC GTGAGCCTCC CCTCCATGCA GGGGGACCTC AAGACCACTG ACCTCAGCAT TCAGCTCCCT TCTGTGGACC TGGAGGTCCA
010101 GGCTGGCCAG GTGGACGTGA AGCTCCCGGA GGGCCACGTG CCCGAGGGAG CTGGCCTCAA AGGGCACCTG CCCAAGGTGG AGATGCCCAG TTTCAAGATG
010201 CCCAAAGTGG ACCTCAAGAG CCCCCAGGTG GACATCAAGG GCCCCAAGCT GGACCTAAAA GTCCCCAAGG CGGAAGTGAC AGTCCCTGAT GTGGAGGTGT
010301 CTCTGCCCAG CGTGGAGGTG GACGTCCAGG CCCCGAGAGC CAAGCTGGAT GGTGCGCGGC TGGAGGGGGA CCTGTCCCTG GCTGAAAAGG ATGTGACTGC
010401 CAAAGACAGC AAGTTCAAAA TGCCCAAGTT CAAGATGCCC TCCTTCGGGG TGTCGGCCCC AGGCAGGTCC ATCGAGGCCT CGCTGGATGT GTCTGCGCCG
010501 AAGGTGGAGG CCGACGTGAG CCTCTCCTCC ATGCAGGGGG ACCTCAAGGC CACTGACCTC AGCATTCAGC CCCCTTCCGC TGACCTGGAG GTCCAGGCTG
010601 TCCAAGTGGA TGTGGAACTC CTGGAGGGCC CCGTGCCCGA GGGAGCCGGC CTCAAAGGGC ACCTGCCCAA AGTGGAGATG CCCAGTTTAA AGACGCCCAA
010701 AGTGGACCTC AAGGGCCCCC AGATAGATGT TAAGGGCCCC AAGCTGGACC TGAAAGGCCC CAAGGCAGAA GTGAGAGTCC CCGATGTCGA GGTGTCTCTG
010801 CCCAGCGTGG AGGTGGATGT CCAGGCCCCG AAGGCCAAGC TGGATGCTGG GCGGCTGGAG GGAGACCTGT CCCTGGCTGA CAAGGACGTG ACTGCCAAAG
010901 ACAGCAAGTT CAAAATGCCC AAATTCAAGA TGCCGTCATT CAGGGTATCG GCCCCAGGGA AGTCCATGGA GGCCTCGGTG GATGTGTCTG CACCCAAGGT
011001 GGAAGCCGAT GTGAGTCTCC CCTCCATGCA GGGGGACCTG AAGACCACTG ACCTCAGCAT TCAGCCCCCT TCTGCCGACC TGAAGGTCCA GGCTGGCCAG
011101 ATGGATGTGA AGCTCCCGGA GGGCCAGGTG CCCGAGGGAG CCGGCCTCAA AGAGCACCTG CCCAAGGTGG AGATGCCCAG TTTGAAGATG CCCAAAGTGG
011201 ACCTCAAGGG CCCCCAGGTG GACATCAAGG GCCCCAAGCT GGACCTAAAA GTCTCCAAGG CGGAAGTCAC AGCCCCTGAT GTGGAGGTGT CTCTGCCCAG
011301 CGTGGAGGTG GACGTCCAGG CCCCAAGAGC CAAACTGGAT AGTGCACAGC TGGAGGGGGA CCTGTCCCTG GCCGACAAGG ATGTGACTGC CAAAGACAGC
011401 AAATTCAAAA TGCCCAAGTT CAAGATGCCG TCATTTGGGG TGTCTGCCCC AGGCAAGTCC ATTGAGGCCT CGGTGCACGT GTCTGCACCC AAGGTGGAGG
011501 CCGATGTGAG TCTCCCCTCC ATGCAGGGGG ACCTCAAGAC CACTGACCTC AGCATTCAGC CCCATTCTGC CGACCTGACG GTCCAAGCTC GCCAGGTGGA
011601 CATGAAACTC CTGGAGGGCC ACGTGCCCGA GGAAGCCGGC CTCAAAGGAC ACCTGCCCAA GGTGCAGATG CCCAGTTTCA AGATGCCCAA AGTCGACCTC
011701 AAGGGCCCTG AAATAGACAT CAAGGGCCCC AAGCTGGACC TAAAAGACCC CAAGGTGGAA GTGACAGCCC CTGATGTGGA GGTTTCTCTG CCCAGCGTGG
011801 AGGTGGACGT CGAGGCCCCA GGAGCCAAGC TGGATGGTGC GCGGCTGGAG GGGGACCTGT CCCTGGCCGA CAAGGACATG ACGGCCAAAG ACAGCAAGTT
011901 CAAAATGCCC AAGTTCAAGA TGCCGTCGTT CGGGGTGTCT GCCCCAGGCA AGTCCATGGA GGCATCAGTG GATGTGACCG CGCCAAAGGT GGAGGCCGAC
012001 GTGAGCCTCC CTTCCATGCA GGGGGACCTC AAGGCCACTG ACCTCAGCGT TCAGCCCCCT TCCGCTGACC TGGAGGTCCA GGCTGGCCAA GTGGACGTGA
012101 AACTCCCAGA GGGCCCCGTG CCCGAGGGAG CCAGCCTCAA AGGGCACCTG CCCAAGGTGC AGATGCCCAG TTTCAAGATG CCCAAAGTGG ACCTCAAGGG
012201 CCCCCAGATA GATGTTAAGG GCCCCAAGCT GGACCTGAAA GGCCCCAAGG CGGAAGTGAC AGCCCCTGAT GTGAAGATGT CTCTGTCCAG CATGGAGGTG
012301 GACGTCCAGG CCCCGAGAGC AAAGCTGGAT GGTGTGCAGC TGGAGGGGGA CCTGTCCCTG GCCGACAAGG ATGTGACTGC CAAAGACAGC AAGTTCAAAA
012401 TGCCCAAGTT CAAGATGCCA TCATTCGGGG TGTCGGCCCC AGGCAAGTCC ATGGAGGCGT CCGTGGATGT GTCTGAGCTG AAGGCGAAAG CCGACGTGAG
012501 CCTCCCCTCC ATGCAGGGGG ACCTCAAGAC CACTGACCTC AGCATTCAGT CCCCTTCCGC CGACCTGGAG GTCCAGGCTG GCCAAGTGGA CGTGAAACTC
012601 CCGGAGGGCC CCCTGCCCAA GGGAGCCGGC CTCAAAGGGC ACCTCCCCAA GGTGCAGATG CCCTGTTTGA AGATGCCCAA AGTGGCCCTC AAGGGCCCCC
012701 AGGTGGATGT CAAGGGCCCC AAGCTGGACC TGAAAGGCCC CAAGGCGGAT GTGATGACCC CCGTCGTGGA GGTGTCTCTG CCCAGCATGG AGGTGGACGT
012801 CGAGGCCCCG GGAGCCAAGC TGGACAGTGT GCGGCTGGAG GGTGACCTGT CCCTAGCCGA CAAGGACATG ACTGCCAAAG ACAGCAAGTT CAAAATGCCC
012901 AAGTTCAAGA TGCCGTCGTT CGGGGTGTCT GCCCCAGGCA AGTCCATCGA GGCCTCGTTG GATGTGTCTG CGCTGAAGGT GGAGGCTGAC GTGAGCCTCC
013001 CCTCCATGCA GGGGGACCTG AAGACCACTC ACCTCAGCAT TCAGCCCCCT TCCGCTGATC TGGAGGTCCA GGCTGGCCAA GAGGATGTGA AACTCCCAGA
013101 GGGCCCTGTG CATGAGGGAG CCGGCCTCAA AGGGCACCTG CCGAAGCTGC AGATGCCCAG TTTCAAGGTA CCCAAAGTGG ACCTCAAGGG TCCCCAGATA
013201 GACGTTAATG TCCCCAAGCT GGACCTGAAA GGCCCCAAGG TGGAGGTGAC GTCCCCCAAC CTGGACGTGT CTCTGCCCAG CATGGAGGTG GACATCCAAG
013301 CCCCAGGAGC CAAGCTGGAC AGTACGCGGC TGGAGGGGGA CCTGTCCCTG GCTGACAAGG ACGTGACTGC CAAAGACAGC AAGTTCAAAA TGCCCAAGTT
013401 CAAGATGCCA TCCTTTGGGA TGTTGTCCCC AGGCAAGTCC ATCGAGGTCT CGGTGGATGT GTCTGCGCCA AAGATGGAGG CCGACATGAG CATTCCCTCC
013501 ATGCAGGGGG ACCTCAAGAC CACTGACCTC CGCATTCAGG CCCCTTCCGC CGACCTGGAG GTCCAGGCTG GCCAGGTGGA CTTGAAACTT CCAGAAGGCC
013601 ACATGCCCGA GGTAGCCGGC CTCAAAGGGC ACCTGCCCAA GGTGGAGATG CCCAGTTTCA AGATGCCCAA AGTGGACCTC AAGGGCCCCC AGGTGGACGT
013701 CAAGGGCCCC AAGCTGGACC TGAAAGGCCC AAAGGCAGAG GTGATGGCCC CCGATGTGGA GGTGTCTCTG CCCAGCGTGG AGACGGATGT CCAGGCCCCA
013801 GGATCCATGC TGGATGGTGC GCGGCTTGAG GGGGACCTGT CCCTGGCCCA CGAGGATGTA GCTGGGAAAG ACAGTAAGTT TCAAGGACCA AAACTGAGCA
013901 CGTCTGGTTT TGAATGGTCG TCAAAGAAAG TTTCCATGTC TTCCTCTGAA ATCGAAGGAA ATGTTACATT CCATGAGAAG ACTTCCACAT TTCCCATTGT
014001 GGAATCTGTT GTTCATGAAG GTGATCTTCA TGATCCATCT CGCGATGGTA ACTTGGGGCT TGCTGTTGGA GAAGTTGGAA TGGATTCGAA GTTTAAGAAA
014101 CTGCATTTTA AAGTGCCCAA AGTTTCATTT TCTTCTACCA AAACTCCTAA AGATAGTTTA GTCCCAGGTG CAAAGTCTAG CATAGGTCTT TCCACGATTC
014201 CTTTATCATC TTCAGAATGC TCAAGTTTTG AATTACAACA GGTTTCGGCT TGTTCAGAGC CATCCATGCA GATGCCTAAG GTGGGTTTTG CTGGGTTTCC
014301 ATCATCCCGG CTTGATCTCA CTGGTCCTCA CTTTGAATCT TCTATTCTCT CTCCCTGTGA GGATGTTACA CTTACAAAAT ACCAGGTGAC TGTTCCCAGA
014401 GCTGCCTTGG CCCCTGAGCT TGCTCTGGAA ATTCCTTCTG GGTCTCAGGC TGATATTCCT CTTCCCAAGA CAGAGTGCTC CACTGACCTG CAGCCTCCAG
014501 AGGGAGTTCC AACATCTCAA GCTGAGAGTC ACTCTGGCCC ACTGAATTCC ATGATTCCTG TTTCTCTTGG TCAGGTATCT TTTCCTAAAT TCTATAAACC
014601 AAAGTTTGTG TTTTCAGTCC CCCAAATGGC AGTTCCTGAG GGAGACCTAC ATGCAGCAGT GGGTGCCCCA GTCATGTCTC CTCTTAGCCC TGGAGAAAGA
014701 GTGCAGTGCC CCTTGCCAAG CACCCAGCTG CCATCCCCAG GCACCTGTGT GTCTCAGGGC CCAGAAGAGC TTGTGGCCTC CTTGCAGACA TCAGTAGTGG
014801 CCCCTGGAGA AGCCCCTTCT GAAGATGCTG ACCACGAAGG GAAAGGGAGT CCCTTGAAAA TGCCTAAGAT TAAGCTTCCA TCATTTAGGT GGTCCCCGAA
014901 GAAGGAAACA GGGCCAAAGG TGGACCCAGA ATGCAGCGTG GAGGACTCAA AACTCAGCCT GGTTTTAGAC AAGGATGAAG TGGCCCCGCA GTCTGCCATC
015001 CACATGGATC TGCCTCCTGA GAGGGATGGA GAGAAGGGGA GGAGCACAAA GCCTGGCTTT GCCATGCCAA AACTTGCACT TCCCAAAATG AAGGCTTCTA
015101 AGAGTGGGGT CAGCCTGCCA CAGAGAGACG TGGATCCTTC CCTTTCTAGT GCCACAGCAG GGGGTAGCTT TCAAGACACA GAAAAGGCCA GCAGTGACGG
015201 TGGTAGGGGA GGACTTGGTG CAACAGCAAG TGCCACAGGA AGTGAGGGTG TGAACCTCCA CCGGCCACAG GTCCACATTC CCAGTTTGGG CTTTGCCAAA
015301 CCTGATCTCA GATCCTCCAA GGCCAAGGTG GAGGTGAGCC AGCCTGAAGC TGACCTGCCT CTTCCCAAAC ATGATCTGTC TACCGAAGGT GACAGCAGAG
015401 GATGTGGGCT CGGGGATGTC CCAGTGAGCC AGCCTTGTGG GGAGGGGATA GCCCCCACAC CTGAAGATCC CCTCCAGCCA TCCTGTAGAA AACCAGATGC
015501 TGAAGTCCTC ACAGTGGAAA GCCCAGAGGA GGAAGCCATG ACCAAGTACT CGCAGGAAAG CTGGTTTAAA ATGCCCAAGT TCCGCATGCC CAGCCTTAGG
015601 CGCTCTTTCA GGGACAGAGG CGGGGCTGGA AAGCTGGAAG TGGCTCAGAC ACAGGCACCG GCAGCAACAG GGGGTGAAGC AGCAGCTAAA GTCAAAGAGT
015701 TCCTTGTTTC TGGGTCAAAC GTGGAGGCAG CTATGTCCCT ACAGCTCCCA GAGGCAGATG CAGAAGTGAC AGCTTCTGAG AGCAAATCAT CCACAGATAT
015801 TCTAAGGTGT GATCTTGACA GCACAGGCTT GAAGCTGCAC CTCTCCACTG CTGGGATGAC TGGGGATGAG CTTTCCACTT CTGAGGTCAG GATCCATCCA
015901 TCCAAAGGAC CTCTCCCTTT TCAGATGCCT GGCATGAGGC TTCCAGAAAC CCAGGTTCTT CCAGGAGAAA TAGATGAGAC TCCTCTTTCC AAGCCAGGAC
016001 ATGACCTTGC CAGCATGGAG GATAAAACAG AGAAATGGTC TTCCCAGCCT GAAGGTCCAC TTAAATTGAA AGCTTCAAGT ACTGATATGC CATCCCAGAT
016101 TTCTGTGGTT AATGTGGATC AACTGTGGGA AGATTCTGTC CTAACTGTCA AATTCCCCAA ATTAATGGTA CCAAGGTTCT CCTTCCCTGC CCCCAGCTCA
016201 GAGGATGATG TGTTCATCCC CACTGTGAGG GAAGTGCAGT GTCCAGAGGC CAATATTGAT ACAGCCCTTT GTAAGGAAAG TCCGGGGCTC TGGGGAGCCA
016301 GCATCCTGAA GGCAGGTGCT GGGGTCCCTG GGGAGCAGCC TGTGGACCTT AACCTGCCTT TGGAAGCTCC CCCAATTTCA AAGGTCAGAG TGCATATTCA
016401 GGGTGCTCAG GTTGAAAGTC AAGAGGTCAC TATACACAGC ATAGTGACAC CAGAGTTTGT AGATCTCTCA GTACCCAGGA CTTTTTCCAC TCAGATTGTG
016501 CGGGAATCAG AGATCCCCAC GTCAGAGATT CAAACACCTT CGTACGGATT TTCCTTATTA AAAGTGAAAA TCCCAGAGCC CCACACGCAG GCTAGAGTGT
016601 ACACAACAAT GACTCAACAC TCTAGGACTC AGGAGGGCAC AGAAGAGGCT CCCATACAAG CCACCCCAGG AGTAGACTCC ATTTCTGGAG ATCTCCAGCC
016701 TGACACTGGA GAACCATTTG AGATGATCTC TTCCAGCGTC AATGTACTGG GACAGCAAAC ACTCACATTT GAAGTTCCTT CTGGCCACCA GCTTGCAGAC
016801 AGCTGTTCAG ATGAGGAGCC AGCAGAAATT CTTGAGTTTC CCCCTGATGA TAGCCAAGAG GCAACCACAC CACTGGCAGA TGAAGGCAGG GCTCCAAAAG
016901 ACAAACCAGA AAGTAAAAAA TCTGGTCTGC TCTGGTTTTG GCTTCCAAAC ATTGGGTTTT CCTCTTCTGT TGATGAGACA GGTGTTGATT CCAAAAATGA
017001 CGTCCAGAGA TCTGCTCCCA TTCAAACACA GCCTGAGGCA CGACCAGAGG CAGAACTGCC TAAAAAACAG GAGAAGGCAG GCTGGTTCCG ATTTCCCAAA
017101 TTAGGGTTCT CCTCATCTCC TACCAAGAAA AGCAAAAGCA CCGAAGATGG GGCAGAGCTG GAAGAACAAA AACTTCAAGA AGAAACAATC ACGTTTTTTG
```

```
017201 ATGCCCGAGA AAGTTTCTCC CCTGAAGAGA AGGAAGAGGG TGAACTGATC GGGCCTGTGG GCACTGGGCT GGACTCCAGA GTGATGGTGA CATCCGCGGC
017301 AAGAACAGAG TTAATCCTGC CCGAGCAGGA CAGAAAAGCT GACGATGAAA GCAAAGGGTC AGGCCTGGGA CCAAATGAAG GCTGAGAGGT ATGGCTCATC
017401 AGTACAAGAG AGATGCAAAA AACTAAGTTG GAAAGTAAAG GCTACACACA CATATGGAGC ACCCCATCCC ACAGCACATT ACATCCACCT CACTTCACAG
017501 AACGGAGAAC AGAGCAGAAA TGACCAGAAC ACCTTTGTCA CCATCACACA GCCCTCCTAA AATGGAACCA AAGCTTCCCA GCTCCCTCAA AGCTTTGGAT
017601 GCAAAGAAGG CACCCTGACT TCCACAAGAC ACCAGAATTC ACACGGTACT CAGAGGCACT GCTGGGGAAG TTTGTTGGTC TTTATTAGAT AAATTTCCAG
017701 AGACCTGTCC ATAATACCCA ACAGAACATG ACTGTTTCTT TGAGGAAAGG GTTATAATGT CTGTGGTGTA CAAGTCGTTT TTGGTATAAC TTCTTTCCTG
017801 CTGCTGCTGC TTCCCGGCAA ACATAGTTTT CCTATTTCAG GCAGAGTGCG GTATATTCCA GGAAACACTG TTTCCTACTC ACTTAGCTTA CTTCTTTGTT
017901 GAATGCCTCA CTAATGGCAA GTTTCAAGAT GTTTTGGGTG ACAATGCACA CATGCTGGGC AAAAGGGTGA TGGCCAGTGG CTGGCAGCTG GGCCAGCAGA
018001 AGCTAGGACA TCTGTGAGTT GTCATTCTCA TCTATCCATG TCCACTGGCC TGCCAGCATC CGCCAGTGCC TTGCCAGTGT GCACGGTCCC ACACTGTGGC
018101 CCCTGAGTCC CCTAATGTAC ACGCTGCAGC CAGAATGCAG ATGGAGCTGG CTTGGCTGTT CCCTGGATGG GCAATAAAGA AAGTGCTGCA TCCCA
```

MAP1B

```
000001 ATGGCATCTT GGTGCACGTC TGTGCCATTC CTCTCAATAT TTCCAAAGAT GGACAAAAGA GAACATCGTT AGTTGAATTC CTGGGCAAAC TGGTCTGAAG
000101 ATTGACTTGG AAGGAGTGGA GGACAAAAGA TCCTTCATCA CCGAAGTGAC GTTTTAGAAA CAGTGGTCCT GATCAACCCT TCTGATGAAG CAGTCAGCAC
000201 CGAGGTGCGC TTAATGATCA CTGATGCTGC CCGACACAAG CTGCTCGTGC TGACCGGGCA GTGCTTTGAA AATACCGGAG AGCTCATTCT CCAGTCCGGC
000301 TCTTTCTCCT TCCAGAACTT CATAGAGATT TTCACCGATC AAGAGATCGG GGAGTTACTA AGCACCACCC ATCCTGCCAA CAAAGCCAGC TTAACCCTGT
000401 TCTGTCCTGA AGAAGGGGAC TGGAAGAACT CCAATCTTGA CAGACACAAT CTCCAAGACT TCATCAATAT TAAACTCAAT TCAGCTTCTA TCTTGCCAGA
000501 AATGGAAGGA CTTTCTGAGT TTACCGAGTA TCTCTCAGAA TCAGTGGAAG TCCCATCTCC CTTTGACATC TTGGAACCTC CCACATCGGG TGGATTTCTG
000601 AAGCTCTCCA AGCCCTGCTG TTATATTTTT CCAGGAGGGA GGGGCGATTC TGCCTTGTTT GCAGTGAATG GTTTCAATAT GCTCATCAAT GGCGGATCAG
000701 AGAGAAAATC CTGCTTCTGG AAGCTCATCC GACACTTAGA CCGAGTGGAC TCCATCCTGC TCACCCACAT TGGGGATGAC AATTTGCCTG GAATAAACAG
000801 CATGTTACAG CGGAAAATTG CAGAGCTCGA GGAAGAACAG TCCCAGGGCT CCACCACAAA TAGTGACTGG ATGAAAAACC TCATCTCCCC TGACTTAGGA
000901 GTTGTATTTC TCAATGTACC TGAAAATCTC AAAAATCCAG AGCCAAACAT CAAGATGAAG AGAAGCATAG AAGAAGCCTG CTTCACTCTC CAGTACCTAA
001001 ACAAATTGTC CATGAAACCA GAACCTCTGT TTAGAAGTGT AGGCAATACT ATTGATCCTG TCATTCTTTT CCAAAAAATG GGAGTAGGTA AACTTGAGAT
001101 GTATGTGCTT AATCCAGTCA AGAGCAGCAA GGAAATGCAG TATTTTATGC AGCAGTGGAC TGGTACCAAC AAAGACAAGG CTGAATTCAT TCTGCCTAAT
001201 GGTCAAGAAG TAGATCTCCC GATTTCCTAC TTAACTTCAG TCTCATCTTT GATTGTGTGG CATCCAGCAA ACCCTGCGGA GAAAATCATC CGAGTCCTGT
001301 TTCCTGGGAA CAGCACCCAG TACAACATCC TGGAAGGGTT GGAAAAGCTC AAACATCTAG ACTTTCTGAA GCAGCCACTG GCCACCCAAA AGGATCTCAC
001401 TGGCCAGGTG CCCACTCCTG TGGTGAAACA AACAAAACTG AAACAGAGGG CTGATAGCCG AGAAAGTCTG AAGCCAGCCG CAAAACCACT TCCTAGCAAA
001501 TCCGTGCGCA AGGAGTCAAA AGAAGAAACC CCTGAGGTCA CAAAAGTGAA TCACGTGGAA AAGCCACCCA AAGTTGAAAG CAAAGAAAAG GTAATGGTGA
001601 AAAAAGACAA GCCAATAAAA ACAGAGACCA AACCTTCAGT GACTGAAAAG GAGGTTCCCA GCAAAGAAGA GCCATCTCCA GTGAAAGCCG AGGTGGCTGA
001701 GAAGCAAGCC ACAGATGTCA AACCCAAAGC TGCCAAGGAG AAGACGGTGA AAAAGGAAAC AAAGGTAAAG CCTGAAGACA AGAAAGAGGA GAAAGAAAAG
001801 CCAAAGAAAG AAGTGGCTAA AAAGGAGGAC AAAACACCTA TCAAGAAGGA GGAAAAACCA AAAAGGAAG AGGTGAAAAA AGAAGTCAAA AAAGAGATCA
001901 AGAAAGAAGA GAAAAAAGAA CCCAAGAAAG AGGTTAAGAA AGAAACACCG CCAAAGGAAG TCAAGAAGGA AGTTAAGAAG GAAGAGAAGA AGGAAGTGAA
002001 AAAGGAAGAA AAGGAACCCA AAAAAGAAAT TAAGAAGCTC CCTAAAGACG CAAAGAAATC ATCTACTCCT CTGTCTGAAG CAAAAAAACC AGCTGCTTTA
002101 AAACCAAAAG TACCCAAGAA GGAAGAGTCT GTCAAGAAAG ATTCTGTTGC TGCCGGAAAG CCAAAGGAGA AGGGGAAAAT AAAAGTCATT AAGAAGGAAG
002201 GCAAGGCCGC AGAGGCTGTC GCTGCAGCTG TCGGCACTGG AGCCACCACA GCAGCTGTCA TGGCGGCAGC TGGAATAGCA GCCATTGGCC CTGCCAAAGA
002301 ACTCGAAGCT GAGAGGTCCC TTATGTCATC TCCTGAGGAT CTAACCAAGG ACTTTGAAGA GTTAAAGGCT GAAGAGGTCG ATGTAACAAA GGACATCAAG
002401 CCTCAGCTGG AGCTAATCGA AGACGAAGAG AAACTGAAGG AAACTGAGCC AGTCGAAGCC TACGTCATCC AGAAGGAGAG AGAAGTCACC AAAGGTCCTG
002501 CCGAGTCCCC TGATGAGGGA ATCACTACCA CTGAAGGGGA GGGCGAATGT GAACAGACAC CTGAGGAGCT GGAGCCCGTC GAGAAGCAGG GAGTAGACGA
002601 CATTGAAAAA TTTGAAGATG AAGGAGCCGG TTTTGAAGAA TCTTCAGAGA CTGGAGACTA TGAAGAGAAG GCAGAAACTG AGGAGGCTGA GGAGCCAGAA
002701 GAGGATGGGG AGGAACACGT ATGTGTGAGC GCCTCCAAGC ACAGCCCCAC TGAGGATGAG GAAAGTGCCA AGGCGGAGGC TGATGCATAC ATCAGGGAGA
002801 AGAGGGAGTC TGTGGCCAGT GGGGATGACC GAGCCGAAGA AGACATGGAT GAGGCCATTG AGAAAGGAGA GGCTGAACAA TCTGAAGAGG AGGCTGATGA
002901 GGAGGACAAA GCTGAAGATG CCAGAGAGGA GGAATATGAG CCGGAAAAAA TGGAAGCTGA AGACTATGTG ATGGCTGTGG TCGACAAGGC TGCAGAGGCT
003001 GGTGGTGCCG AGGAGCAGTA TGGATTCCTC ACCACACCAA CCAAGCAACT AGGAGCCCAG TCTCCTGGCC GAGAACCTGC ATCTTCAATT CATGATGAGA
003101 CTTTACCTGG AGGCTCAGAG AGCGAGGCCA CCGCTTCTGA TGAGGAGAAT CGAGAAGACC AGCCTGAGGA ATTCACTGCC ACCTCTGGCT ACACTCAGTC
003201 TACTATTGAG ATATCCAGTG AGCCCACCCC CATGGATGAG ATGTCTACCC CTCGAGACGT GATGAGTGAT GAGACCAACA ATGAAGAGAC GGAGTCCCCT
003301 TCTCAGGAAT TCGTAAATAT CACCAAATAT GAATCTTCAT TGTATTCTCA GGAATACTCT AAACCTGCTG ATGTTACACC GCTCAACGGA TTTTCTGAAG
003401 GATCAAAAAC AGATGCCACT GATGGCAAGG ATTACAATGC TTCAGCCTCT ACCATATCAC CACCCTCTTC CATGGAGGAA GACAAATTCA GCAGATCTGC
003501 TTTACGTGAT GCTTACTGCT CTGAAGTGAA AGCCAGCACC ACTTTGGACA TCAAAGATAG CATCTCAGCT GTTTCAAGTG AAAAGGTCAG CCCATCGAAG
003601 AGCCCGTCCC TGAGTCCATC TCCACCATCA CCCTTAGAAA AGACCCCCCT GGGTGAACGT AGTGTGAACT TCTCTCTGAC GCCCAATGAG ATTAAAGTCT
003701 CTGCAGAGGC AGAAGTAGCC CCGGTGTCTC CTGAGGTGAC CCAAGAAGTA GTTGAAGAAC ATTGTGCTAG TCCTGAGGAC AAGACTCTGG AAGTGGTGTC
003801 ACCATCTCAG TCCGTGACTG GCAGTGCTGG TCACACACCT TACTATCAAT CTCCTACTGA CGAGAAATCC AGTCATCTCC CTACAGAAGT CATTGAAAAA
003901 CCACCAGCAG TTCCAGTGAG TTTTGAATTC AGTGATGCCA AAGATGAGAA TGAAAGGGCT TCAGTAAGCC CCATGGATGA GCCCGTGCCT GACTCAGAGT
004001 CTCCTATTGA AAAAGTTTTG TCTCCTTTAC GCAGCCCGCC CCTCATTGGA TCCGAGTCTG CTTATGAAAG TTTTCTAAGT GCTGATGACA AGGCTTCTGG
004101 CAGAGGTGCC GAAAGTCCTT TTGAAGAAAA GAGTGGAAAA CAAGGCTCTC CAGACCAAGT AAGTCCAGTT TCTGAAATGA CTTCTACTAG TCTTTACCAA
004201 GACAAACAGG AAGGGAAAAG CACAGACTTT GCACCAATAA AAGAAGACTT TGGCCAAGAA AAGAAAACTG ATGATGTTGA AGCCATGAGT TCTCAACCAG
004301 CACTGGCTCT GGATGAAAGG AAATTAGGAG ATGTTTCTCC CACACAAATA GATGTCAGTC AGTTTGGATC TTTTAAAGAA GACACTAAGA TGTCCATTTC
004401 TGAAGGTACT GTCTCAGACA AGTCAGCTAC TCCTGTTGAT GAGGGCGTAG CAGAAGACAC GTACTCTCAT ATGGAGGGTG TGGCCTCAGT GTCCACAGCC
004501 TCAGTGGCTA CGAGCTCATT TCCAGAGCCA ACAACAGATG ATGTGTCTCC ATCTCTGCAT GCTGAGGTTG GCTCCCCACA TTCCACAGAA GTAGATGACT
004601 CCCTTTCAGT GTCTGTTGTG CAAACACCTA CCACATTCCA GGAAACAGAA ATGTCTCCAT CTAAAGAAGA ATGCCCAAGA CCGATGTCAA TTTCTCCACC
004701 AGATTTCTCC CCTAAAACTG CAAAGTCCAG GACACCCGTT CAAGATCACA GATCTGAACA GTCCTCAATG TCTATTGAAT TTGGCCAAGA ATCTCCTGAG
004801 CAATCCCTTG CTATGGACTT CAGTCGACAG TCTCCAGATC ACCCTACAGT GGGTGCAGGC GTGCTTCACA TCACTGAAAA TGGGCCAACT GAAGTGGACT
004901 ACAGTCCTTC TGACATGCAG GACTCCAGTT TATCACATAA GATACCACCT ATGGAGGAGC CGTCCTACAC CCAAGATAAT GATCTTTCTG AGCTCATCTC
005001 AGTATCTCAG GTAGAGGCCT CCCCGTCCAC CTCTTCTGCT CATACCCCTT CTCAGATCGC TTCTCCTCTC CAAGAAGATA CTCTATCCGA TGTTGCTCCT
005101 CCCAGAGATA TGTCCTTATA TGCCTCACTC ACCTCTGAAA AAGTGCAAAG TCTGGAAGGA GAGAAGCTCT CTCCAAAATC TGATATCTCT CCACTCACCC
005201 CACGAGAGTC CTCTCCTTTA TATTCACCTA CTTTTTCAGA TTCTACCTCT GCAGTCAAAG AGAAAACAGC AACTTGCCAC AGTTCCTCTT CTCCACCAAT
005301 AGATGCAGCA TCCGCAGAGC CCTATGGCTT CCGTGCCTCA GTGTTATTCG ATACAATGCA ACACCATCTA GCCTTGAATA GAGATTTGTC CACACCTGGC
005401 CTGGAGAAGG ACAGTGGAGG GAAGACACCT GGTGACTTTA GCTATGCCTA TCAAAAGCCT GAGGAAACAA CCAGGTCCCC AGATGAAGAA GATTATGACT
005501 ATGAGTCTTA TGAGAAGACC ACCCGGACCT CAGATGTGGG TGGCTATTAC TATGAGAAGA TAGAGAGAAC CACAAAATCT CCAAGTGACA GTGGCTACTC
005601 CTATGAGACC ATTGGGAAAA CTACCAAGAC CCCTGAAGAT GGTGACTATT CCTATGAAAT TATTGAGAAG ACCACACGGA CCCCTGAAGA GGGTGGGTAC
005701 TCATATGACA TAAGTGAAAA GACCACCAGC CCCCCCGAAG TGAGTGGTTA CAGCTATGAA AAGACTGAGA GGTCTAGAAG GCTTCTGGAT GACATCAGCA
005801 ATGGCTATGA TGACTCTGAG GATGGTGGCC ACACACTTGG GGACCCCAGC TACTCTTATG AAACCACTGA GAAAATTACC AGTTTCCCTG AGTCTGAAGG
005901 TTATTCCTAT GAGACATCTA CAAAGACAAC ACGAACCCCT GATACTTCCA CATACTGTTA CGAGACTGCA GAGAAAATCA CTAGAACCCC TCAGGCATCC
006001 ACATATTCCT ACGAGACTTC AGACCTATGC TACACTGCAG AAAAGAAGTC CCCCTCAGAA GCCCGTCAGG ATGTCGATTT ATGCCTCGTG TCCTCTTGTG
006101 AATACAAGCA CCCCAAGACA GAGCTTTCAC CCTCTTTCAT TAATCCCAAT CCTCTTGAGT GGTTTGCCAG TGAAGAACCC ACTGAAGAAT CTGAAAAGCC
006201 CCTCACTCAA TCAGGGGGAG CCCCACCGCC TCCAGGAGGA AAGCAACAGG GCCGACAGTG TGATGAAACC CCTCCCACCT CAGTCAGCGA GTCAGCCCCA
006301 TCCCAGACCG ACTCTGATGT TCCCCCGGAG ACTGAAGAGT GCCCCTCCAT CACGGCCGAT GCCAATATCG ACTCTGAAGA CGAGTCGGAA ACCATCCCCA
006401 CAGACAAAAC TGTCACGTAC AAACACATGG ACCCACCTCC AGCTCCCGTG CAAGACCGCA GCCCTTCGCC ACGCCACCCT GATGTGTCCA TGGTGGACCC
006501 AGAGGCCTTG GCCATTGAGC AGAACCTGGG CAAAGCTCTA AAGAAAGATC TGAAAGAGAA GACCAAAACC AAAAAGCCAG GTACAAAGAC CAAGTCATCT
006601 TCACCTGTCA AAAAGAGTGA TGGGAAGTCT AAGCCCTTGG CAGCTTCACC AAAACCAGCG GGCTTGAAAG AATCCTCGGA TAAAGTGTCC AGGGTGGCTT
006701 CTCCTAAGAA GAAAGAATCT GTGGAAAAGG CAGCAAAACC CACCACCACT CCTGAGGTCA AAGCTGCACG TGGGGAAGAG AAAGACAAGG AGACCAAGAA
006801 TGCTGCCAAT GCCTCTGCAT CCAAGTCGGC CAAGACCGCC ACTGCAGGAC CAGGAACTAC CAAGACGACC AAGTCATCTG CTGTGCCCCC AGGCCTCCCT
006901 GTGTATTTGG ACCTGTGCTA CATTCCTAAC CACAGCAATA GTAAGAATGT TGATGTGGAA TTTTTCAAGA GAGTGCGGTC TTCCTACTAC GTGGTGAGTG
007001 GGAATGACCC TGCTGCTGAG GAGCCCAGCC GGGCTGTCCT GGACGCTTTG TTGGAAGGAA AGGCTCAGTG GGGCAGCAAC ATGCAGGTGA CACTGATCCC
007101 AACTCATGAC TCAGAAGTGA TGAGGGAATG GTACCAGGAG ACCCATGAGA AACAGCAAGA TCTCAACATC ATGGTTTTAG CAAGCAGCAG CACAGTGGTT
007201 ATGCAAGATG AATCCTTCCC TGCATGCAAG ATTGAACTGT AAAAACCAAG GCCAGCCACA CCACAGGATC TGAACTTTGT TTCCAGAAAT TCTTCAATTT
007301 GAAATCACCT TTTCTAAAAA GTCAATTCAT CTAGTTAAGT CGCTGAACAA TTACCTGCCA AATGCTATAC TGTGTCATGG TGATGCAAGT CACTAAATTT
007401 CTCAGTTTTT GCTGATTGCT AAGGGAAATA ACAGTATTTC CACAATAGGG TTCAAATTCC TGCAAAATTA CCTACCCCAG TTCATCTCTG CTGAACATTT
```

```
007501 GGAAACCATG CACTAGCCAA CCCAACTGAC TTCTGCTAGG TAGAGGCATT TGTCTTAGAG AGAGAGAGAG CGCGGGAGAG AGTGAGAGAG AGTGAGAGCA
007601 CAAAGATAAC GCAGGAGAGA GAGAGAGAAA GAATGAGAAA GAAAAGGAAT GCAAGAGAAG GAGATGTAAT GACAGAGAGT TCTGGTGAGA TACCCAGAGA
007701 GAAAAAGAGA GAGCAGGGTG GGGTAAGGAG GAGAAAATAA ACCAACAATT AGGTCTGCAT TTTCTCAGGC AGTAGGCATT CTTTAGTCTA CATAGGCAAA
007801 GTTTTCCATT TTTGTCAGTC TGAGTCATCA AAAAGAGTCT TAATTTTCTA AAACAAGTTG GCTAGAAGAA AGTAAAAAGA ACAACACTTG TTATGAGGGC
007901 ATGTGATATT TTCACATCTT AATTAAGCTC CTTCAGTTTG AAGGCTGCAC ACTGACATAA TGTAGTGAGT GTAGACTGGC CATGCAAGTG GTTTGGGCCC
008001 CATTCAGAAC TCTCAGACTC TAAACACACA AGTAGATTGA TCTAAGGCAT GCTCCCAGCA TTTGTCCACC CACTTAGTCC ACTCTGAGTC GATTAACCTG
008101 CATGCAGCAA CACCCAAGTC CACCCCAATT AACTGAAGCA AATACCAAAG CAGTTGGGAG TACATATGGT AGACAATTTG CCTTAGGAAG TGACTTGAAT
008201 GTACAAAGAT ACTTGATGCA CTTATTTTTT AATGTGAGAC AGCAAGTTTA TAAAACATCC ATATAGGATT ATAGATACTT AAAGGAACAC GTGGGTGAGC
008301 GTGTGTGGGG GTACTAGAAG CTGATCTGAT TGGTCCAACA GTTTGATGCT GAGTCATGCG TGTTGAATCC CACTTCAGTG CACCTGTGGC CTCTCAGTCA
008401 AACAAGTTGT GCCTTTCACA GCTTCTTTAC TACTGCAAGT TCAAGACTGA AATGGCTTCT ATGATCAGAA CTGGGAAAAC AGTGAATCTT ATGGTGGAAG
008501 AGGTTCTCAG CAAGTGTACA GTATTTACCT TCCTTTGTCT TACATTGGCT TTTTAAATTT TCCATTAATT TCAACATAAT TATGGGAACA AGTGTACAGA
008601 AGAATTTTTT TTTTAAGATA TGTGAGAACT TTTCATAGAT GAACTTTTTA ACAAATGTTT TCATTTACAG GAAATTGCAA AGAAAATTCT CAAGTGATAG
008701 TCTTTTTTTT TAAGTGTTTC GTAAGACAAA AATTGAATAA TGTTTTTTGA AGTTCTGGCA AGATTGAAGT CTGATATTGC AGTAATGATA TTTATTAAAA
008801 ACCCATAACT ACCAGGAATA ATGATACCTC CCACCCCTTG ATTCCCATAA CATAAAAGTG CTACTTGAGA GTGGGGGAGA ATGGCATGGT AGGCTACTTT
008901 TCAGGGCCTT GACAAGTACA TCACCCAGTG GTATCCTACA TACTTCTTTC AAGATCTTCA ACCATGAGGT AAAAGAGCCA AGTTCAAAGA ACCCTAGCAC
009001 AAATTTGCTT TGGGATTTTC TTTTCTGGAA AAAAAAAATA AAAGAAATAG TACATTGAAA ACAAATGAAT TCTCAACTCC TACGGTTCAT GTAGAGTTTA
009101 GAGAAAATTT CCATCATTGT CATCATTGAA CTGTGAACCT GGGAAGCCAG ATCATGATTA ACACTGACAT CAAGTTTCAA GTTGCAGATC AATGCACCCA
009201 GTGTTCAGAT GAGGCAAACT TCTCCGTGAC AACTGTGCTG TGCTCTGTCA CATTACATTT CCTGCAGACT CTAAGATCTA CGGAGTAGAG AACAATGACC
009301 TCATTTTATT TTCTATGTTA GTTATTTATT TCAAAATTAA CATTTTAGTT GATTTTTGTC TGATAAGTCT ATGTTTTGCA CTGCTAACTA TGATGAGGGT
009401 TTAAAAAAAT GCTTCTTCAG GGTCCTTTCA CTGAGGACCT ATGCAGTCTA CTTAATGCTG TGAATTACAT TTTTCAAATG TTTAATTTTT TAAAGAAAAT
009501 TAATATTCTA TTTTTGTTAG GCTTCTCTAG AAATGCAGCT TTTATTTATT ACCCCATTTC TTTCAAGTCC TTGGAAAATA ACATATTAAG GGTACAAGAA
009601 ATTAACACAT GATGGAAAAG TCATTGTGAC GCCAATGAAT TTCATTGAGT ATAAACTCAT CTACTTCAAA TTTATTTTAT AACACAACCT AAGATACTCA
009701 AGATAATTAT TTAATGGTTA GCTCTTAAGT TGAATTGGTC TACATAATGC GTGGGAAGAA AACCAGATTT TTAGCCTTCT TGCCAAATCC AGACCTCTGG
009801 TTGATTTTTC TTTGACAGAA GATGCAAGTT ATTTTCCAAT TTCACAATTA AATGTATTTA ACCTGAACAT TATTTTGCTT TAAAAACTAT AAACATTGTA
009901 GGAGAATTAT AGCCAGTCTT CAGTTATAAC CACTCCACCC TCCTCACTTT CTCTCTCTCT CTCTCTTTTT TTTTTTTTTT TTTTTTGCTA TGGGATTTAA
010001 TGGGAAAAAT ATGTAAAAAC TGTCACTAGT CAGCTGGCTC TTTTTCCTAT GAAATCTATC AGTACCTTTC TCCATCCGTT GTTCTCAATA TGACCACAGA
010101 GCCTGAGTAT ACCAAGAAAA CCAATATTCG CATTACAGGT TGCTCCTGTC CTTCCAGACA CCTTTCCTGC CTGTGTGACT AACCTAATTT TGCTAGTTCC
010201 ATAAATACAC GATTAGTTTA GTAACAGCCA TCACAATGTA CCATGTACAT TCATGGTGAG AGCTAAAGAT CGACACAGAC TTCTAGGAGC TTTGTTCATA
010301 CTGATTACTT AATCCAATTG TATAGATTGA ATATTTGAGT GGAAGGAATT TACACTCTGT TTAAATGATG GGATTCTATC GAGATAGCAC TCATGATCAT
010401 GACCTTTTTG GTAGTATTCT TAAACAAAAT TCTACAGAGA CTAAATGTTA GCGATGATCC TCCATTTTCA ATTTTAACCA ATTCTGTCCC CTTTCTCAAA
010501 ACCCTGAGCC CTGTGCATGC TTTCTCAGTC TTGTGGTGGG ACTGGATACA ATGACTAACT TCCCCTCCTC CCCTCTTTAA ACACCATTTT CCATGGAGTT
010601 CAAAAAAATT TTTTTTCTTA ACGTTACATA TCATAGTGAA TGGTTTCCCC AGTGTATATG AATGTTTTAA GTGTCTCCAA TAGCTTATGC AGTCTAGGAG
010701 CTTTCCAATA CTCATTTAAT TAAGATTTAA TCATTTGCTA ATGGAAATCT TACCACCTTT CATTTTCCCT CTGTTACCAA ATTTCAGCTC TTAGGAGCTG
010801 CTCTACAATT CTGAATTTGC TTTTCTTGCC TCTCTTTAGT CACCTGTCAC AGGAGGTTCC TGCTCAGTAA TGATATTGTG AGTTAGGATA ATAACTTTTT
010901 TTTTTTGTGC TTCAGATTTA GAAGAAAAGA TCCTGTTTCC ATTTGAAAGG AACTGTAAGC TTTTATCTTT TAACCAACTG AACAATACAC CAAAAGCAGC
011001 CTAGGGATGA GCATTTCTTT GAAAGCAATT AGGTTATTCA CCTGGTATTA AAACTATTTA CTGTTAAAAA ATCTGTGACT TCATGAAGTT GATTTTTAAA
011101 GGCAGCATCA AAAACTGAAA AGGAAGGGAA AAAATAGGCA GCTTCTCTGC ACTTGTTTGG AGCTCCCCAA AACAGGAGCC ATGGAGAAGT GGCATCAAGA
011201 CCGGGCTGCC CTTTCGAGAA CACCCTGTGG CAGTTCAGAG ACACGCTTTT CCTACACTGC ATGCAGCCCC TCTTTCCAGC ACTGGAAAGA AGTGGTCTTG
011301 AGCCCAGCTG AGAAGCACTT CACACTCCTC TCTCTTGTTC TGAATGGTGT TTGTGTCAGT CTGCAGCTGT GTATGGTATT ATGTCTTATA ATCCTGCATC
011401 ACTTCTATCC TATCCAGTCA TATCTAATGT AGAAAATTAG TTTCCAGTGA AAGTAATATG TAGTGCTTTT ATGATATTTG TGTGCAATAT CCCCTCTTCC
011501 ATTGAGGATA TTTGATGTAA AGGAAAAAAA AAAACTCAGT TCCACAATAA AATACAAAAG TGGCAAAA
```

HUWE1

```
000001 GACTGAGGGC TAGCGAGGGG AGCAGGGCTG GAGCAGGGCT GGAGCAGGGC TGGAGCAGGG CTGAAGCAGG GCTGAAGCAG GGCCGCGGAC CCCGCACGCT
000101 CCTGCGGGCC CCGCGGAGCC ATTGCGGCCG AGGCCTCGGC AGGCGCCAGC GGAGAGCTAG CCGCATCTTC GGGGGCAGCC CGGCAGCTGC CGGCGGCGCG
000201 GCGAGAGAGC GGCTGACAGA GGGGATGCGA GGTCCTCCAG CAGCCTGACC TGAGTGGGTT AGTGATCCAG AGAAACCAGC AGGCCAACTT GGTCAGGAAG
000301 GTTCGGGAAG CTGTTGGAGC AGTGTGGGGA ATTTCCCACC AGGATGAGTA TGATTGGCTG TGATTTTAGA TCGTAAAGCT GAAAATTGAA ATCATGAAAG
000401 TAGACAGGAC TAAACTGAAG AAGACACCTA CTGAGGCTCC TGCAGACTGC AGAGCCTTAA TAGACAAACT CAAAGTTTGT AATGATGAGC AACTTCTCTT
000501 GGAACTGCAG CAGATCAAAA CATGGAACAT TGGAAAGTGC GAGTTATATC ACTGGGTGGA CCTGTTGGAC CGCTTCGATG GAATACTGGC AGATGCTGGA
000601 CAGACAGTGG AGAATATGTC ATGGATGCTC GTATGTGATA GGCCAGAAAG AGAGCAACTG AAAATGCTTC TCTTGGCTGT GTTGAACTTC ACAGCCTTGC
000701 TCATTGAGTA CAGCTTTTCC CGGCATCTGT ACAGTTCCAT AGAGCATTTG ACAACTTTAT TGGCTTCCTC TGATATGCAA GTGGTGCTGG CAGTCCTCAA
000801 TCTCCTATAT GTATTTAGCA AAAGATCAAA CTACATCACT CGTCTGGGAT CTGACAAGAG GACCCCGCTG CTAACTCGGC TACAACATTT GGCAGAGAGC
000901 TGGGGTGGAA AGGAGAATGG CTTTGGACTT GCAGAATGTT GCAGAGACTT GCATATGATG AAATATCCAC CCAGTGCAAC TACACTACAC TTTGAATTCT
001001 ATGCAGATCC TGGGGCCGAG GTCAAAATTG AGAAAAGGAC AACTAGTAAC ACACTACATT ATATTCACAT AGAGCAACTT GACAAGATTT CAGAAAGCCC
001101 TTCTGAAATC ATGGAATCTC TTACCAAAAT GTACAGCATT CCTAAGGATA AGCAGATGCT GTTATTTACA CACATACGAC TGGCCCATGG CTTTTCTAAT
001201 CACAGGAAGC GATTGCAGGC AGTTCAGGCC AGACTGCATG CAATATCTAT ATTAGTGTAT TCCAATGCCT TGCAGGAATC AGCAAACAGT ATCTTGTATA
001301 ATGGCTTGAT AGAGGAGTTG GTAGATGTCC TTCAGATAAC GGATAAGCAG CTTATGGAGA TTAAAGCAGC TTCTTTACGA ACATTAACAT CAATTGTCCA
001401 CTTGGAGAGA ACTCCCAAAC TCAGCAGTAT TATTGACTGT ACTGGAACTG CCTCCTACCA TGGATTTTTG CCAGTGCTTG TAAGGAACTG TATCCAGGCC
001501 ATGATTGATC CTTCCATGGA TCCATACCCT CACCAGTTTG CCACTGCTCT CTTCTCTTTT TTATACCATC TGGCCAGCTA CGATGCTGGT GGTGAAGCCT
001601 TGGTCTCCTG TGGAATGATG GAAGCCTTAT TGAAGGTCAT AAAGTTTCTT GGCGATGAAC AGGACCAGAT AACATTTGTC ACCAGAGCCG TCAGAGTGGT
001701 TGACCTTATC ACCAACCTGG ATATGGCAGC TTTTCAATCC CATAGTGGAC TTTCTATCTT CATTTATAGA CTTGAGCATG AAGTAGATTT GTGCCGAAAA
001801 GAATGTCCGT TTGTGATCAA GCCAAAGATC CAGAGACCCA ATACTACACA AGAAGGAGAG GAAATGGAAA CTGATATGGA TGGAGTCCAG TGTATTCCAC
001901 AACGAGCAGC ACTTCTGAAA TCCATGTTGA ATTTCCTCAA GAAGGCCATC CAAGACCCTG CTTTCTCAGA TGGCATACGA CATGTGATGG ATGGTTCTCT
002001 GCCTACCTCC CTGAAACACA TCATCAGCAA TGCAGAATAC TATGGCCCAT CACTCTTCCT CCTAGCTACT GAAGTGGTGA CTGTGTTTGT ATTTCAAGAA
002101 CCATCACTGC TCTCCTCACT CCAGGACAAT GGATTGACAG ATGTCATGCT GCATGCACTG CTTATCAAAG ATGTTCCTGC TACCCGTGAA GTCCTTGGCT
002201 CCCTCCCAAA TGTATTCAGT GCACTCTGTT TGAATGCCCG AGGTCTTCAG TCTTTTGTTC AGTGTCAGCC TTTTGAACGC CTCTTCAAAG TTCTTCTGTC
002301 TCCAGATTAC CTCCCAGCCA TGCGGAGGAG GAGAAGTTCT GATCCCCTTG GGGATACTGC ATCCAACCTG GGGAGTGCTG TCGATGAGCT CATGAGACAT
002401 CAGCCCACCC TTAAAACAGA TGCAACGACT GCCATCATCA AGTTACTTGA AGAAATCTGT AATCTTGGAA GGGACCCCAA ATACATCTGT CAGAAGCCAT
002501 CAATCCAGAA GGCAGATGGC ACTGCCACTG CTCCTCCCCC AAGGTCTAAT CATGCCGCAG AAGAAGCCTC TAGTGAGGAT GAGGAGGAAG AGGAAGTACA
002601 GGCCATGCAG AGCTTTAATT CTACCCAGCA AAATGAAACT GAGCCTAATC AGCAGGTTGT TGGTACAGAG GAACGTATTC CTATTCCCCT CATGGATTAC
002701 ATCCTTAATG TGATGAAATT TGTGGAATCT ATTCTGAGCA ACAATACAAC AGATGACCAC TGCCAGGAAT TTGTGAATCA GAAAGGACTG TTGCCTTTGG
002801 TTACCATTTT GGGTCTTCCC AATCTGCCCA TTGACTTTCC CACATCTGCT GCCTGTCAGG CTGTTGCAGG TGTCTGCAAA TCCATATTGA CACTGTCACA
002901 TGAACCCAAA GTCCTTCAAG AGGGTCTCCT TCAGTTGGAC TCCATCCTCT CCTCCCTGGA GCCCTTACAC CGCCCCATTG AATCCCCTGG GGGCTCAGTG
003001 TTGTTGCGAG AACTGGCTTG CGCAGGCAAT GTTGCTGATG CTACCCTCTC AGCCCAGGCC ACACCTCTGC TGCATGCACT CACTGCTGCC CATGCCTACA
003101 TCATGATGTT TGTTCATACT TGCAGAGTTG GACAGAGTGA AATTCGTTCC ATCTCCGTAA ACCAGTGGGG CTCTCAATTG GGTCTGAGTG TTTTGAGCAA
003201 GCTGAGCCAG TTATACTGTT CCCTGGTGTG GGAAAGCACT GTCCTCCTCT CTCTGTGTAC CCCAAACAGC CTACCATCTG GGTGTGAATT TGGCCAGGCA
003301 GATATGCAGA AACTGGTTCC AAAGGATGAG AAGGCAGGTA CGACCCAGGG CGGAAAAAGA TCAGATGGGG AACAGGATGG AGCAGCTGGA AGTATGGATG
003401 CTTCTACCCA GGGCTTATTA GAAGGCATTG GGCTAGATGG TGACACATTG GCTCCCATGG AGACAGATGA ACCTACTGCT TCAGACTCTA AGGGCAAATC
003501 TAAAATCACA CCAGCAATGG CTGCCAGAAT TAAGCAAATC AAGCCTTTGT TATCAGCTTC CTCCAGATTA GGCCGAGCAC TTGCTGAGCT ATTTGGACTT
003601 CTTGTTAAAC TTTGTGTGGG ATCTCCTGTC CGCCAGAGAA GGAGCCATCA TGCTGCCAGC ACCACTACAG CACCGACACC TGCCGCGCGA TCAACAGCCT
003701 CAGCTCTCAC TAAGCTCTTG ACTAAGGGGT TATCTTGGCA GCCCCCACCA TATACACCTA CTCCCCGATT CAGGCTGACA TTCTTCATCT GTTCAGTTGG
003801 TTTCACATCC CCAATGCTGT TTGATGAGAG GAAGTATCCC TACCACCTCA TGCTGCAAAA ATTTCTCTGC TCCGGAGGCC ACAATGCTCT TTTTGAAACT
003901 TTCAACTGGG CTCTGTCCAT GGGAGGTAAA GTTCCTGTTT CTGAGGGATT GGAACACTCA GACTTGCCTG ATGGCACAGG AGAATTCCTA GATGCCTGGC
004001 TTATGCTGGT GGAGAAGATG GTGAATCCCA CCACGGTGCT TGAATCTCCA CATTCGCTGC CTGCCAAATT GCCTGGAGGT GTCCAGAACT TTCCCCAGTT
004101 CAGTGCACTG CGCTTCCTTG TGGTAACTCA GAAAGCAGCC TTTACTTGCA TCAAAAACTT ATGGAACCGG AAACCCCTGA AGGTATATGG TGGACGAATG
004201 GCTGAATCGA TGCTGGCCAT TCTATGCCAC ATCCTCCGAG GAGAACCTGT GATTCGAGAG AGACTAAGCA AGGAGAAGGA GGGGTCTCGA GGAGAAGAGG
```

004301 ATACAGGGCA AGAGGAAGGT GGCTCCCGCC GGGAACCTCA AGTCAACCAG CAACAACTGC AACAGCTCAT GGACATGGGC TTCACAAGGG AACATGCAAT
004401 GGAGGCACTG TTGAACACCA GCACCATGGA GCAGGCCACA GAGTACCTTT TAACCCACCC TCCTCCAATC ATGGGAGGAG TTGTTCGGGA TCTCAGCATG
004501 TCTGAAGAGG ACCAGATGAT GAGAGCAATT GCTATGTCTC TGGGACAGGA TATTCCAATG GATCAAAGGG CAGAGTCACC TGAGGAAGTT GCTTGCCGGA
004601 AGGAGGAAGA GGAACGGAAA GCTCGGGAAA AGCAGGAGGA GGAAGAGGCT AAATGTCTAG AGAAGTTCCA GGATGCTGAC CCGTTGGAAC AAGATGAGCT
004701 CCACACTTTC ACAGATACTA TGTTGCCAGG CTGCTTCCAC CTTCTTGATG AGCTGCCAGA CACAGTATAC CGTGTGTGTG ACCTGATCAT GACAGCAATC
004801 AAACGTAATG GAGCAGATTA TCGTGACATG ATTCTGAAGC AAGTAGTCAA TCAGGTGTGG GAAGCTGCTG ATGTATTGAT CAAAGCTGCT CTTCCCCTGA
004901 CAACAAGTGA CACAAAAACC GTGTCAGAGT GGATAAGTCA GATGGCCACA CTGCCCCAGG CCTCCAATTT GGCTACTAGA ATCTTGCTTT TAACGCTACT
005001 TTTTGAGGAG TTGAAGCTAC CTTGTGCTTG GGTGGTTGAA TCAAGTGGCA TCCTTAATGT CCTAATCAAA CTCTTGGAAG TGGTTCAGCC CTGCCTCCAG
005101 GCAGCCAAGG AGCAGAAGGA AGTCCAGACC CCAAAGTGGA TCACACCAGT GTTGCTCCTG ATTGATTTCT ATGAAAAGAC AGCCATCTCC TCAAAAAGGA
005201 GAGCCCAGAT GACTAAGTAC CTGCAATCCA ACAGCAACAA CTGGCGCTGG TTTGATGATC GCTCTGGGCG TTGGTGTAGT TACAGTGCAA GCAACAATAG
005301 CACTATTGAT TCTGCCTGGA AATCTGGAGA GACAAGCGTG CGATTCACTG CAGGCCGAAG AAGATACACG GTCCAATTCA CTACAATGGT GCAGGTTAAT
005401 GAGGAAACAG GGAACCGACG CCCTGTGATG CTGACTCTCC TCAGGGTACC TCGGCTGAAT AAAAATTCAA AAAACAGCAA TGGACAGGAA CTAGAGAAGA
005501 CGCTGGAAGA AAGCAAAGAA ATGGATATCA AACGTAAAGA AAATAAAGGC AATGATACCC CTTTGGCCCT AGAGAGTACA AACACTGAAA AGGAGACAAG
005601 CCTGGAGGAA ACAAAAATCG GGGAGATCCT GATCCAGGGC TTGACAGAAG ATATGGTGAC TGTTTTAATC CGGGCCTGCG TGAGCATGCT GGGAGTCCCT
005701 GTGGACCCAG ATACTTTGCA TGCCACCCTT CGTCTCTGTC TGAGGCTCAC CCGGGACCAC AAATATGCCA TGATGTTTGC AGAACTGAAG AGTACCCGCA
005801 TGATCTTGAA TTTGACCCAG AGCTCAGGCT TCAATGGGTT TACTCCCCTG GTCACCCTTC TCTTAAGACA CATCATTGAG GACCCCTGTA CCCTTCGTCA
005901 TACCATGGAA AAGGTTGTTC GCTCAGCAGC TACAAGTGGA GCTGGTAGCA CTACCTCTGG TGTTGTGTCT GGCAGCCTCG GCTCTCGGGA GATCAACTAC
006001 ATCCTTCGTG TCCTTGGGCC AGCCGCATGC CGCAATCCAG ACATATTCAC AGAAGTGGCC AACTGCTGTA TCCGCATCGC CCTTCCTGCC CCTCGAGGCT
006101 CAGGAACTGC TTCAGATGAT GAATTTGAGA ATCTTAGAAT TAAAGGCCCT AATGCTGTAC AGCTGGTGAA GACCACCCCT TTGAAGCCCT CACCTCTGCC
006201 TGTCATCCCT GATACTATCA AGGAAGTGAT CTATGATATG CTGAATGCTC TGGCTGCATA CCATGCTCCA GAGGAAGCAG ATAAATCTGA TCCTAAACCT
006301 GGGGTTATGA CCCAAGAGGT TGGCCAGCTC CTGCAAGACA TGGGTGATGA TGTATACCAG CAGTACCGGT CACTTACGCG TCAGAGCAGT GACTTTGATA
006401 CGCAGTCAGG TTTTTCCATT AATAGTCAGG TCTTTGCTGC AGATGGTGCC TCCACTGAGA CTTCCGCATC TGGGACCTCC CAAGGAGAGG CTTCAACTCC
006501 AGAGGAGTCT CGAGATGGGA AGAAAGATAA AGAAGGGGAC CGGGCCTCTG AGGAAGGCAA ACAGAAAGGC AAGGGCAGCA AACCTTTAAT GCCTACCTCC
006601 ACTATCCTTC GTCTTCTGGC AGAGTTGGTG AGGTCCTATG TTGGTATTGC TACCCTGATT GCCAACTACA GCTACACTGT GGGCCAGTCT GAACTGATCA
006701 AAGAGGACTG CAGTGTGCTA GCTTTTGTTC TGGACCACCT GCTCCCACAT ACCCAGAATG CAGAAGACAA GGACACCCCT GCCTTGGCCC GCCTGTTCCT
006801 CGCAAGCCTG GCTGCTGCAG GGAGTGGCAC AGATGCCCAG GTGGCCCTAG TGAATGAAGT AAAAGCAGCC CTTGGACGGG CACTGGCTAT GGCTGAGAGT
006901 ACAGAGAAAC ATGCCAGGCT TCAGGCAGTG ATGTGTATCA TCAGTACTAT CATGGAGTCC TGCCCCTCCA CCTCCAGCTT CTACAGCAGT GCCACAGCGA
007001 AGACCCAGCA CAATGGCATG AACAACATCA TTCGGCTTTT CCTGAAGAAG GGACTGGTTA ATGACCTGGC CAGAGTACCT CACAGCTTAG ACCTGTCCAG
007101 TCCCAACATG GCCAACACAG TCAATGCTGC TCTGAAGCCT TTGGAAACAC TTTCCCGGAT TGTGAACCAG CCCAGTAGCC TTTTTGGCAG CAAGAGTGCT
007201 TCTAGCAAGA ACAAGTCTGA GCAGGATGCC CAAGGAGCCT CTCAAGATTC CAGTAGCAAC CAGCAGGACC CAGGCGAGCC TGGGGAAGCA GAAGTGCAGG
007301 AGGAGGATCA TGATGTCACT CAGACAGAGG TGGCAGATGG GGATATCATG GATGGGGAGG CTGAAACCGA CTCAGTGGTG ATTGCTGGGC AGCCTGAGGT
007401 GCTCAGTTCA CAAGAGATGC AGGTTGAGAA TGAGCTGGAG GACCTGATAG ATGAGTTGCT TGAGAGGGAT GGCGGATCTG GGAACAGTAC AATTATAGTG
007501 AGCAGAAGTG GAGAGGATGA ATCACAAGAG GACGTGCTGA TGGATGAAGC TCCTTCCAAC CTCAGCCAAG CTTCCACCTT GCAGGCCAAC CGAGAAGATT
007601 CCATGAATAT CCTGGACCCT GAGGATGAGG AGGAGCACAC TCAGGAAGAG GACAGCAGTG GCAGTAACGA GGATGAGGAT GATAGTCAGG ATGAAGAGGA
007701 GGAGGAGGAG GAAGATGAGG AAGATGATCA GGAGGATGAT GAAGGTGAAG AGGGAGATGA AGACGATGAC GACGATGGCT CTGAGATGGA ATTGGATGAG
007801 GATTATCCTG ATATGAACGC TTCTCCCTTG GTCCGATTTG AGCGCTTTGA CCGGGAGGAT GATCTCATCA TTGAGTTTGA CAACATGTTC TCCAGTGCTA
007901 CAGACATCCC CCCATCCCCA GGAAATATCC CTACCACCCA TCCACTGATG GTGCGCCATG CAGACCACAG TTCTCTGACA CTGGGCAGTG GCTCTTCAAC
008001 AACTCGTCTC ACCCAGGGCA TCGGGCGCAG TCAGAGGACC CTAAGGCAGC TGACGGCCAA TACTGGCCAC ACCATTCATG TTCACTACCC TGGGAATCGC
008101 CAGCCCAACC CTCCTCTTAT ACTGCAGAGG TTGCTTGGTC CCTCAGCTGC TGCTGACATC CTTCAGCTGA GCAGCAGCCT TCCCCTACAA AGCCGGGGTC
008201 GGGCCCGCCT CCTGGTAGGC AACGATGACG TCCACATCAT CGCCCGTTCT GATGATGAGC TGCTGGATGA CTTTTTCCAT GATCAGAGCA CAGCTACCAG
008301 CCAAGCAGGA ACCCTGTCCA GCATCCCCAC AGCCCTGACC CGCTGGACAG AAGAATGCAA AGTTCTCGAT GCTGAGAGCA TGCATGACTG TGTTTCAGTG
008401 GTTAAAGTGT CCATTGTCAA TCACCTGGAA TTCCTGAGGG ATGAGGAGCT GGAAGAAAGG CGAGAGAAGC GCAGGAAACA ACTGGCTGAG GAAGAAACAA
008501 AGATAACTGA TAAAGGCAAA GAAGATAAGG AGAACAGGGA TCAGAGTGCA CAGTGTACTG CATCTAAGTC AAATGACTCC ACTGAACAGA ATCTCTCAGA
008601 TGGGACGCCT ATGCCTGACA GCTACCCAAC AACCCCATCT TCAACTGATG CAGCTACATC TGAGTCCAAG GAGACCCTTG GCACTCTGCA ATCCTCACAA
008701 CAGCAACCAA CACTCCCAAC CCCACCAGCT TTGGGAGAGG TTCCTCAGGA GCTGCAGTCT CCAGCTGGAG AAGGGGGCAG CTCTACACAG CTATTGATGC
008801 CTGTAGAGCC AGAGGAATTG GGTCCCACAA GGCCAAGTGG GGAAGCAGAA ACAACTCAGA TGGAGTTATC CCCAGCTCCC ACTATAACCT CACTTTCCCC
008901 AGAGAGAGCT GAGGATTCTG ATGCACTGAC GGCTGTCAGC AGTCAGCTAG AAGGCTCTCC TATGGATACA AGCAGCCTGG CTTCCTGTAC CTTAGAGGAG
009001 GCTGTGGGTG ACACTTCAGC AGCTGGCAGT TCTGAGCAGC CCAGAGCAGG CAGCTCCACT CCTGGGGATG CCCCACCAGC TGTGGCGGAA GTGCAAGGCA
009101 GGAGTGATGG GTCAGGGGAA TCTGCCCAGC CACCTGAGGA CAGCTCCCCA CCTGCATCCT CTGAGAGCTC TTCCACCAGA GATTCTGCCG TGGCCATTTC
009201 TGGAGCAGAT TCCCGAGGAA TCCTAGAAGA GCCGTTGCCT TCAACAAGCA GTGAAGAAGA AGATCCCCTT GCGGGTATCA GTCTCCCTGA AGGTGTGGAC
009301 CCCTCTTTTC TGGCTGCCCT GCCTGATGAC ATCCGTCGGG AAGTTCTACA GAACCAGCTA GGCATTCGTC CACCAACCCG GACTGCCCCC TCCACAAATA
009401 GCTCAGCGCC TGCAGTGGTG GGGAATCCTG GTGTGACTGA AGTGAGCCCT GAGTTTCTGG CTGCCCTGCC TCCAGCCATT CAGGAGGAAG TACTGGCACA
009501 GCAGAGAGCT GAGCAGCAGC GACGAGAACT AGCACAGAAT GCCAGCTCAG ACACCCCTAT GGACCCTGTG ACCTTCATCC AGACTCTGCC CTCAGACCTG
009601 CGCCGTAGTG TCCTAGAGGA TATGGAGGAC AGTGTGTTAG CTGTGATGCC ACCTGACATT GCAGCTGAGG CTCAAGCCCT GAGACGAGAG CAAGAAGCCC
009701 GGCAGCGACA GCTCATGCAT GAGCGTCTGT TTGGGCACAG TAGCACCTCC GCACTCTCTG CTATTCTCCG AAGCCCGGCT TTCACCAGTC GCTTAAGTGG
009801 CAACCGTGGG GTCCAGTATA CTCGCCTTGC TGTGCAGAGA GGTGGCACCT TCCAGATGGG GGGTAGCAGC AGCCATAACA GGCCTTCTGG CAGTAATGTA
009901 GATACTCTCC TCCGCCTCCG AGGACGGCTC CTTCTGGACC ACGAAGCCCT TTCTTGTCTC TTGGTCCTAC TTTTTGTGGA TGAGCCAAAG CTCAATACTA
010001 GCCGTCTACA CCGAGTACTG AGAAATCTCT GCTACCATGC CCAGACCCGC CACTGGGTCA TCCGCAGTCT GCTCTCCATC TTGCAGCGCA GCAGTGAGAG
010101 TGAGCTATGC ATTGAAACAC CCAAACTCAC TACAAGTGAG GAAAAGGGCA AAAAGTCGAG CAAGAGCTGT GGGTCAAGTA GCCATGAGAA CCGTCCCCTG
010201 GACCTGCTAC ACAAGATGGA GTCAAAGAGC TCCAACCAGC TTTCCTGGCT CTCAGTATCC ATGGATGCAG CCCTAGGCTG CAGGACTAAT ATATTTCAGA
010301 TCCAGCGTTC AGGGGGGCGT AAACATACCG AGAAGCATGC AAGCGGTGGC TCCACCGTCC ACATCCATCC CCAAGCTGCT CCTGTTGTCT GCAGACACGT
010401 TTTGGATACA CTCATTCAAT TGGCCAAGGT ATTTCCCAGC CACTTCACAC AGCAGCGGAC CAAAGAAACA AACTGTGAGA GTGATCGGGA AAGGGGCAAT
010501 AAGGCCTGTA GCCCATGCTC CTCACAGTCC TCCAGCAGTG GCATTTGCAC AGACTTCTGG GACTTATTGG TAAAACTGGA CAACATGAAT GTCAGCCGGA
010601 AAGGCAAGAA CTCCGTGAAG TCAGTGCCAG TGAGCGCTGG CGGTGAGGGG GAAACCTCTC CATACAGCCT CGAGGCCTCT CCACTGGGGC AGCTCATGAA
010701 CATGTTGTCA CACCCAGTCA TCCGCCGGAG CTCTCTCTTA ACTGAGAAAC TCCTCAGACT CCTTTCTCTC ATCTCAATTG CTCTCCCAGA AAACAAGGTG
010801 TCAGAAGCAC AGGCTAATTC TGGCAGCGGT GCTTCCTCCA CCACCACTGC CACCTCAACC ACATCTACCA CCACCACCAC TGCCGCCTCC ACCACGCCCA
010901 CACCCCCTAC TGCACCCACC CCTGTCACTT CTGCTCCAGC CCTGGTTGCT GCCACGGCTA TTTCCACCAT TGTCGTAGCT GCTTCGACCA CAGTGACTAC
011001 CCCCACGACT GCTACCACTA CTGTTTCAAT TTCTCCCACT ACTAAGGGCA GCAAATCTCC AGCGAAGGTG AGTGATGGGG GCAGCAGCAG TACAGACTTT
011101 AAGATGGTGT CCTCTGGCCT CACTGAAAAC CAGCTACAGC TCTCTGTAGA GGTGTTGACA TCCCACTCTT GTTCTGAGGA AGGCTTAGAG GATGCAGCCA
011201 ACGTACTACT GCAGCTCTCC CGGGGGGACT CTGGGACCCG GGACACTGTT CTCAAGCTGC TACTGAATGG AGCCCGCCAT CTGGGTTATA CCCTTTGTAA
011301 ACAAATAGGT ACCCTGCTGG CCGAGCTGCG GGAATACAAC CTCGAGCAGC AGCGGCGAGC CCAATGTGAA ACCCTCTCTC CTGATGGCCT GCCTGAGGAG
011401 CAGCCACAGA CCACCAAGCT GAAGGGCAAA ATGCAGAGCA GGTTTGACAT GGCTGAGAAT GTGGTAATTG TGGCATCTCA GAAGCGACCT TTGGGTGGCC
011501 GGGAGCTCCA GCTGCCTTCT ATGTCCATGT TGACATCCAA GACATCTACC CAGAAGTTCT TCTTGAGGGT ACTACAGGTC ATCATCCAGC TCCGGGACGA
011601 CACGCGCCGG GCTAACAAGA AAGCCAAGCA GACAGGCAGG CTAGGTTCCT CCGGTTTAGG CTCAGCTAGC AGCATCCAGG CAGCTGTTCG GCAGCTGGAG
011701 GCTGAGGCTG ATGCCATTAT ACAAATGGTA CGTGAGGGTC AAAGGGCGCG GAGACAGCAA CAAGCAGCAA CGTCGGAGTC TAGCCAGTCA GAGGCGTCTG
011801 TCCGGAGGGA GGAATCACCC ATGGATGTGG ACCAGCCATC TCCCAGTGCT CAAGATACTC AATCCATTGC CTCCGATGGA ACCCCACAGG GGGAGAAGGA
011901 AAAGGAAGAA AGACCACCTG AGTTACCCCT GCTCAGCGAG CAGCTGAGTT TGGACGAGCT GTGGGACATG CTTGGGGAGT GTCTAAAGGA ACTAGAGGAA
012001 TCCCATGACC AGCATGCGGT GCTAGTGCTA CAGCCTGCTG TCGAGGCCTT CTTTCTGGTC CATGCCACAG AGCGGGAGAG CAAGCCTCCT GTCCGAGACA
012101 CCCGTGAGAG CCAGCTGGCA CACATCAAGG ACGAGCCTCC TCCACTCTCC CCTGCCCCCT TAACCCCAGC CACGCCTTCC TCCCTTGACC CATTCTTCTC
012201 CCGGGAGCCC TCATCTATGC ACATCTCCTC AAGCCTGCCC CCTGACACAC AGAAGTTCCT TCGCTTTGCA GAGACTCACC GCACTGTGTT AAACCAGATC
012301 CTACGGCAGT CCACGACCCA CCTTGCTGAT GGGCCTTTTG CTGTCCTGGT AGACTACATT CGTGTCCTCG ACTTTGATGT CAAGCGCAAA TATTTCCGCC
012401 AAGAGCTGGA GCGTTTAGAT GAGGGGCTCC GGAAAGAAGA CATGGCTGTG CATGTCCGTC GTGACCATGT GTTTGAAGAC TCCTATCGTG AGCTGCATCG
012501 CAAATCCCCC GAAGAAATGA AGAATCGATT GTATATAGTA TTTGAAGGAG AAGAAGGGCA GGATGCTGGT GGGCTCCTGC GGGAGTGGTA TATGATCATC
012601 TCTCGAGAGA TGTTTAACCC TATGTATGCC TTGTTCCGTA CCTCACCTGG TGATCGAGTC ACCTACACCA TCAATCCATC TTCCCACTGC AACCCCAACC
012701 ACCTCAGCTA CTTCAAGTTT GTCGGACGCA TTGTGGCCAA AGCTGTATAT GACAACCGTC TTCTGGAGTG CTACTTTACT CGATCCTTTT ACAAACACAT
012801 CTTGGGCAAG TCAGTCAGAT ATACAGATAT GGAGAGTGAA GATTACCACT TCTACCAAGG TCTGGTTTAT CTGCTGGAAA ATGATGTCTC CACACTAGGC
012901 TATGACCTCA CCTTCAGCAC TGAGGTCCAA GAGTTTGGAG TTTGTGAAGT TCGTGACCTC AAACCCAATG GGGCCAACAT CTTGGTAACA GAGGAGAATA
013001 AGAAGGAGTA TGTACACCTG GTATGCCAGA TGAGAATGAC AGGAGCCATC CGCAAGCAGT TGGCGGCTTT CTTAGAAGGC TTCTATGAGA TCATTCCAAA

013101 GCGCCTCATT TCCATCTTCA CTGAGCAGGA GTTAGAGCTG CTTATATCAG GACTGCCCAC CATTGACATC GATGATCTGA AATCCAACAC TGAATACCAC
013201 AAGTACCAGT CCAACTCTAT TCAGATCCAG TGGTTCTGGA GAGCATTGCG TTCTTTCGAT CAAGCTGACC GTGCCAAGTT CCTCCAGTTT GTCACAGGTA
013301 CTTCCAAGGT ACCCCTGCAA GGCTTTGCTG CCCTCGAAGG CATGAATGGC ATTCAGAAGT TTCAGATCCA TCGAGATGAC AGGTCCACAG ATCGCCTGCC
013401 TTCAGCTCAC ACATGTTTTA ATCAGCTGGA TCTGCCTGCC TATGAGAGCT TTGAGAAGCT CCGCCACATG CTACTGTTGG CTATCCAGGA GTGCTCTGAA
013501 GGCTTTGGGC TGGCCTAATA AGGCCCTGCC CAACTCCGTG GGGTTTTTTT TACCATTGTT GGACCTGGGG AGGGGGAGT TAAAAAAAGA ACCAGAAAGA
013601 AATTGTCAAA AACCAATAAA TGAAATCCAC CAACTCACCG TGTGTGTCCC AGCTGCCCCA TCTTCCCCAG CGCATACCTG TTCCTCTTCT CATTCTCTCC
013701 CCGCCGCCTG TTTCCTCACC TTCTCTCCCC TTTCCATGCC GTCCATGATC CCCACCCCAT GTGTTTTAAA AAGGCAGTAG CCTTTGCAGG GACCTGTCTG
013801 TCCCAACTGT TTGAACAGTG TGCTCCTCAG ATTCTGTGTT CAGAAGGATT TGCTGCATTG AGACTTGAAA CCTTTGGATA GGGGAAAAAA TTATATATAT
013901 ATATATTTTT TTGTTCTGTT TGCATTTCTT AATTTGTGCT TGGAATGTGT TGATGTGCAC AGCTAATGAT TCAATGCGAG ACAAGATTGG CGTCTGTGTT
014001 GTGGAGGTTT CAAATAAAGA GCACTCTTCA TAACTCACTT TTCACAATGG AGTTTTTTTC AAACTTAAAA AAAAAAACAA AAAACTCTTA AGCACATGCT
014101 AGGCATCTGG AGAAAAGATG GATTCTCCAG GAAACCATCC CTGCCCTACC CGCTTGCCCA CCTGCCTCCA CCACTGGTGC AACTTCCTCC TTCAGAGTCA
014201 GTTTCTGCAG TCAGGAGAGA TGAGCAGTTC ACCAGGAATT GGGGGTGGGG GCATCTGTTT TTTTTTTTTA GAATGGGAGC TGCACTTGGG GATGGTGATG
014301 GTGTTAGAGA TTGCAGCCCA GGACAAAGCA TCACCTTGCT CAAGGGGAAC AAACTGCAGC AGGTTGTACA AAACATTCAG AGAGCTCACT TGACCCAACC
014401 GAGGGTTTTA CTTGGTGAGC CTTTCTAGTA GTCTTGAGTC TGGGGCTCAG TTTTAGATTT TTATTTCATT AATGTTTGTT TATTTCTAAT AAATTTTAAT
014501 AAGCGAGTAA TTTAACTAGG TCGCCAGAAG ACCATTATTT TTGTTTGTCT ATTTTTGTTG TCGTCACTCC CTCTCTCGTC ATCTGTCTGC CAGTCTGTTC
014601 ACAGATCACC TTGTAGTCCC TTGTGGTTTT TACTCCATCC AGTGCTTAAA GCTGATCTAA GGACTTCGAT GGTTCCAGCC TTCAAAGAAA AAATAATTTA
014701 ATAAAAATTT AGTTAGAAAA AATAACCCCA A

UBR4

000001 GCAGTAGTAC GACGGAAGAT GGCGACGAGC GGCGGCGAAG AGGCGGCGGC AGCGGCTCCG GCGCCGGGGA CCCCGGCAAC GGGGGCGGAC ACGACCCCGG
000101 GCTGGGAGGT GGCTGTGCGG CCCCTGCTGT CCGCGTCCTA CTCCGCCTTC GAGATGAAGG AGTTGCCGCA GCTGGTGGCC TCAGTCATCG AGAGTGAATC
000201 AGAAATCCTG CACCATGAGA AGCAGTACGA GCCATTCTAC TCATCTTTTG TTGCACTTTC CACACACTAT ATTACAACAG TTTGCAGTCT CATTCCCCGG
000301 AACCAACTTC AGTCAGTGGC AGCAGCCTGT AAAGTTCTAA TTGAGTTTTC TCTCCTGCGT CTGGAGAATC CAGATGAGGC TTGTGCTGTG TCCCAGAAAC
000401 ACTTGATTCT CCTAATCAAG GGCCTGTGCA CTGGCTGTAG CCGACTAGAT AGAACTGAAA TTATCACATT TACAGCAATG ATGAAATCCG CCAAGCTGCC
000501 CCAAACAGTG AAGACACTTT CAGACGTGGA AGATCAGAAA GAGCTGGCCT CACCAGTAAG CCCTGAGTTG AGGCAAAAGG AGGTACAGAT GAATTTTTTG
000601 AACCAGCTGA CCTCAGTTTT TAACCCTAGA ACTGTAGCAT CACAACCTAT CAGTACACAG ACTCTGGTGG AAGGAGAAAA TGATGAGCAG TCATCTACAG
000701 ATCAAGCCTC AGCTATCAAA ACCAAGAATG TGTTCATAGC TCAGAACGTG GCTAGTCTTC AAGAGCTTGG TGGCTCGGAG AAGCTACTGC GTGTATGTTT
000801 GAACCTGCCA TATTTCCTAC GCTATATCAA TCGGTTCCAA GATGCAGTTT TAGCTAATTC CTTCTTCATA ATGCCTGCAA CAGTAGCAGA TGCCACTGCT
000901 GTTCGTAATG GCTTTCATTC ATTGGTGATT GATGTAACTA TGGCATTGGA TACCCTTTCT CTACCTGTGT TGGAACCTCT CAATCCTTCT CGTCTACAAG
001001 ATGTGACAGT CCTCAGCCTA AGTTGTCTGT ATGCAGGTGT GAGTGTGGCA ACGTGCATGG CCATCCTCCA TGTGGGTAGT GCCCAGCAAG TGCGGACAGG
001101 GTCCACGAGC TCCAAAGAAG ATGACTATGA AAGTGACGCA GCTACAATTG TCCAGAAATG TCTCGAAATC TATGACATGA TTGGACAAGC AATCAGCAGT
001201 TCTCGCCGGG CTGGTGGTGA GCACTATCAG AATTTCCAAT TGCTGGGTGC TTGGTGCTTG TTAAACAGCC TTTTCCTCAT ACTGAACCTC AGTCCTACTG
001301 CGTTGGCTGA TAAGGGGAAA GAGAAGGACC CACTGGCTGC CCTCCGAGTC AGAGACATCC TTTCTCGTAC TAAAGAGGGA GTGGGCTCCC CTAAACTGGG
001401 GCCTGGAAAA GGGCATCAGG GATTTGGGGT ACTCTCAGTA ATATTGGCAA ACCATGCCAT CAAACTGCTA ACGTCTCTCT TTCAAGACCT ACAAGTGGAG
001501 GCCCTTCACA AGGGTTGGGA GACAGATGGC CCCCCTGCAG CCTTGAGCAT TATGGCCCAG AGCACCTCCA TACAGAGGAT TCAACGGCTG ATTGACTCTG
001601 TCCCACTGAT GAACCTGCTC TTGACGTTAC TTTCAACTTC CTACAGAAAG GCATGTGTCC TGCAGCGGCA GAGGAAGGGC TCCATGAGCA GCGATGCCAG
001701 CGCCTCCACC GACTCCAATA CTTACTATGA GGACGATTTC AGTAGCACGG AGGAGGACAG CAGCCAAGAC GATGACAGTG AGCCTATTTT GGGGCAATGG
001801 TTTGAGGAGA CTATTTCTCC CAGTAAAGAG AAAGCAGCAC CTCCGCCTCC TCCCCCACCT CCTCCACTGG AAAGCTCTCC TCGGGTTAAA AGCCCCAGTA
001901 AGCAGGCCCC TGGTGAGAAG GGCAACATTC TGGCGAGTCG CAAAGATCCT GAGTTGTTCT TAGGTCTGGC TTCCAACATT TTGAACTTCA TCACCTCTTC
002001 CATGCTGAAC TCTCGGAACA ATTTTATCCG AAACTATCTG AGTGTATCTC TTTCAGAACA CCATATGGCC ACCCTAGCCA GTATCATCAA GGAGGTGGAC
002101 AAAGATGGAC TCAAGGGTTC ATCAGATGAA GAGTTTGCTG CAGCTCTCTA TCACTTCAAC CACTCACTGG TAACCTCTGA CCTTCAGTCA CCTAACCTGC
002201 AGAACACACT GTTGCAGCAG CTAGGAGTGG CTCCTTTTTC TGAGGGCCCT TGGCCCTTGT ACATTCACCC TCAAAGCCTC TCTGTGCTTT CACGCCTCCT
002301 GCTCATCTGG CAACATAAAG CCAGTGCTCA AGGTGACCCT GACGTCCCAG AATGCCTTAA AGTTTGGGAC AGGTTTTTGT CTACAATGAA GCAGAATGCC
002401 CTGCAAGGTG TGGTGCCCAG TGAGACAGAG GATCTGAATG TAGAACACCT GCAGATGCTC CTCCTCATTT TCCACAATTT CACCGAGACA GGCCGGCGGG
002501 CCATATTGTC GCTTTTTGTC CAGATCATCC AGGAGTTGAG CGTCAACATG GATGCTCAGA TGCGCTTCGT GCCGCTTATC TTGGCTCGCC TCCTTCTCAT
002601 CTTTGATTAT CTGCTTCATC AGTACTCCAA AGCCCCTGTG TATCTATTTG AGCAGGTACA GCATAACCTG CTAAGTCCTC CCTTTGGGTG GGCAAGTGGA
002701 TCCCAGGACA GCAACAGCCG CCGGGCAACC ACTCCTCTCT ATCATGGATT CAAAGAAGTA GAAGAAAACT GGTCTAAGCA TTTCTCATCA GATGCTGTCC
002801 CACACCCCAG ATTCTACTGT GTCCTGTCCC CAGAAGCCTC AGAGGATGAT TTGAACCGAC TTGATTCTGT GGCATGTGAC GTCCTTTTCT CCAAGCTTGT
002901 CAAGTATGAT GAGCTTTATG CTGCACTGAC AGCCCTGCTT GCAGCTGGGT CCCAGCTTGA TACAGTTAGG AGAAAGGAAA ACAAGAATGT AACAGCCTTG
003001 GAGGCCTGTG CCCTTCAATA TTACTTCTTG ATACTGTGGA GGATCCTAGG AATTTTACCA CCATCAAAGA CTTACATTAA CCAGCTATCC ATGAACTCAC
003101 CTGAGATGAG CGAATGTGAC ATCTTGCACA CTCTGCGATG GTCTTCTCGG CTCCGGATCA GCTCCTATGT CAACTGGATA AAGGATCACC TTATCAAACA
003201 GGGAATGAAG GCTGAGCATG CTAGCTCGCT TCTAGAACTG GCATCCACCA CTAAGTGTAG CTCAGTGAAA TATGATGTTG AAATAGTAGA GGAATACTTC
003301 GCTCGACAGA TCTCATCCTT CTGTAGTATC GACTGTACCA CCATCTTGCA GCTGCATGAA ATTCCCAGTC TGCAGTCCAT CTACACCCTT GATGCCGCGA
003401 TCTCAAAGGT CCAGGTCTCT TTGGATGAGC ATTTTTCTAA GATGGCTGCT GAGACTGATC CTCATAAGTC GTCTGAGATT ACCAAGAACC TACTTCCAGC
003501 CACGCTGCAA CTCATTGACA CCTATGCATC GTTCACCAGA GCCTATTTGC TGCAAAACTT TAATGAAGAG GGAACAACTG AGAAACCTTC CAAGGAGAAA
003601 CTGCAAGGCT TTGCTGCTGT TTTGGCTATT GGCTCTAGCA GGTGCAAGGC AAATACTCTG GGTCCGACAC TGGTTCAGAA TTTGCCATCG TCAGTGCAGA
003701 CTGTGTGTGA GTCCTGGAAC AACATCAATA CCAATGAATT TCCCAATATT GGATCCTGGC GCAATGCCTT TGCCAATGAC ACCATCCCTT CAGAGAGTTA
003801 TATTAGTGCA GTGCAGGCTG CACACCTGGG GACTCTCTGT AGCCAAAGTC TGCCCCTGGC TGCTTCCCTG AAGCATACCC TCCTCTCACT GGTCAGGTTG
003901 ACTGGAGATC TTATTGTTTG GTCAGATGAG ATGAACCCAC CACAGGTAAT TCGGACACTG CTACCTCTTC TTTTGGAATC AAGCACTGAG AGTGTTGCCG
004001 AGATCAGTAG CAACTCCCTG GAACGCATCT TGGGCCCTGC TGAGTCTGAT GAGTTCTTGG CTCGTGTTTA TGAGAAGCTG ATCACTGGTT GTTACAACAT
004101 TCTGGCCAAT CATGCAGATC CTAACAGTGG ACTGGATGAA TCCATCCTGG AGGAATGTCT CCAGTACTTG GAAAAGCAGC TGGAAAGTAG CCAGGCTCGT
004201 AAAGCTATGG AGGAGTTTTT CTCTGACAGT GGAGAACTTG TACAGATCAT GATGGCAACA GCCAATGAGA ACCTCTCTGC TAAATTCTGT AACCGAGTTT
004301 TGAAATTCTT CACCAAACTC TTCCAGCTGA CTGAGAAGAG CCCTAACCCG AGCCTGTTGC ATCTCTGTGG CTCCCTGGCA CAACTGGCCT GTGTGGAACC
004401 TGTGCGCCTG CAGGCCTGGC TCACCCGCAT GACTACATCG CCCCCAAAAG ATTCTGATCA GCTGGATGTA ATTCAGGAGA ACCGGCAGCT GCTGCAGTTA
004501 CTGACCACAT ACATTGTTCG GGAAAACAGC CAAGTTGGGG AAGGTGTGTG TGCTGTTCTT CTGGGCACCC TGACTCCCAT GGCAACAGAG ATGCTGGCCA
004601 ACGGTGATGG GACTGGCTTC CCTGAACTTA TGGTTGTGAT GGCCACTCTG GCCAGTGCAG GTCAAGGTGC TGGTCACCTT CAGCTTCATA ATGCTGCTGT
004701 GGATTGGCTG AGCAGATGCA AGAAATACCT GTCACAGAAG AATGTAGTTG AAAAACTGAA TGCCAATGTA ATGCATGGAA AGCATGTGAT GATCTTGGAG
004801 TGCACATGCC ATATCATGTC TTACTTGGCT GATGTCACGA ATGCCCTGAG CCAGAGTAAT GGTCAAGGCC CAAGTCATCT CTCAGTGGAT GGGGAAGAGC
004901 GGGCCATTGA AGTAGACTCA GACTGGGTGG AGGAGTTGGC GGTGGAAGAG GAAGATTCCC AGGCTGAGGA TTCAGATGAA GATTCTCTTT GCAATAAACT
005001 CTGCACTTTT ACGATCACAC AGAAAGAATT CATGAACCAG CATTGGTACC ACTGTCACAC CTGTAAAATG GTGGATGGCG TGGGTGTCTG CACAGTGTGT
005101 GCTAAGGTGT GCCACAAGGA TCATGAGATT TCCTATGCCA AGTATGGATC CTTCTTCTGT GACTGTGGAG CCAAGGAAGA TGGCAGCTGT TTGGCTCTGG
005201 TGAAGAGAAC TCCTAGCAGT GGCATGAGCT CTACCATGAA GGAGTCGGCA TTTCAGAGTG AACCCAGGAT TTCAGAGAGT CTAGTGCGTC ATGCCAGCAC
005301 CTCCTCGCCA GCTGACAAAG CCAAGGTTAC CATCAGTGAT GGAAAGGTTG CTGACGAAGA GAAGCCCAAG AAGAGCAGCC TCTGCCGCAC AGTAGAGGGC
005401 TGCCGGGAGG AATTACAGAA CCAGGCCAAT TTCTCCTTCG CTCCTCTCGT GTTAGACATG CTTAATTTCC TTATGGATGC CATTCAGACC AACTTCCAGC
005501 AAGCTTCAGC CGTCGGGAGC AGCAGCCGTG CTCAGCAAGC CCTCAGTGAG CTACACACTG TGGAGAAGGC AGTGGAGATG ACAGACCAGC TGATGGTTCC
005601 CACCTTAGGG TCCCAGGAAG GTGCCTTTGA GAATGTGCGG ATGAATTACA GTGGAGACCA GGGCCAGACC ATCCGGCAGC TGATCAGTGC TCATGTGCTC
005701 AGGCGGGTGG CTATGTGTGT GCTCTCCTCT CCCCATGGGC GCCGCCAACA TTTGGCTGTC AGCCATGAGA AGGGCAAGAT CACCGTTCTG CAGCTCTCTG
005801 CACTCCTGAA GCAAGCAGAT TCCAGCAAAA GGAAGTTAAC TCTGACCCGC TTGGCTTCTG CCCCAGTTCC TTTTACTGTG TTGAGCCTCA CAGGAAATCC
005901 CTGCAAGGAA GACTACTTGG CGGTTTGTGG GCTAAAGGAC TGTCATGTGC TCACCTTTAG TAGCTCAGGC TCTGTTTCGG ATCACTTGGT TTTGCACCCT
006001 CAGTTGGCAA CGGGGAACTT CATCATCAAA GCCGTGTGGT TACCTGGTTC ACAGACCGAG TTAGCAATTG TCACCGCAGA CTTTGTTAAG ATTTATGACC
006101 TGTGTGTTGA TGCCTTGAGT CCAACCTTCT ATTTTCTCCT GCCAAGCTCA AAGATAAGAG ATGTTACCTT CCTTTTCAAT GAGGAGGGAA AGAACATCAT
006201 TGTTATAATG TCTTCGGCTG GGTACATCTA TACTCAGCTT ATGGAAGAGG CCAGCAGTGC CCAGCAGGGA CCCTTCTATG TCACTAATGT GTTGGAAATC
006301 AATCATGAGG ACCTGAAGGA CAGTAACAGC CAGGTGGCGG GCGGTGGTGT GTCCGTGTAC TACTCCCACG TGTTGCAGAT GTTGTTCTTC AGCTATTGTC
006401 AAGGCAAATC ATTCGCAGCC ACCATCAGCA GGACAACCCT GGAGGTGTTG CAACTCTTCC CCATCAACAT CAAAAGTTCC AATGGTGGCA GTAAGACTTC
006501 TCCTGCTCTT TGCCAGTGGT CTGAGGTGAT GAACCACCCT GGCTTGGTGT GCTGTGTCCA GCAAACTACA GGGGTGCCGC TGGTAGTTAT GGTGAAACCA
006601 GACACTTTTC TTATCCAGGA GATTAAGACT CTTCCTGCTA AAGCGAAGAT CCAAGACATG GTTGCTATTA GGCACACGGC CTGCAATGAG CAGCAGCGGA
006701 CAACAATGAT TCTGCTGTGT GAGGATGGCA GCCTGCGCAT TTACATGGCC AACGTGGAGA ACACCTCCTA CTGGCTGCAG CCATCCCTGC AGCCCAGCAG
006801 TGTCATCAGC ATCATGAAGC CTGTTCGAAA GCGCAAAACA GCTACAATCA CAACCCGCAC GTCTAGCCAG GTGACTTTCC CCATTGACTT TTTTGAACAC

006901 AACCAGCAGC TGACAGATGT GGAGTTTGGT GGTAACGACC TCCTACAGGT CTATAATGCA CAACAGATAA AACACCGGCT GAATTCCACT GGCATGTATG
007001 TGGCCAACAC CAAGCCCGGA GGCTTCACCA TTGAGATTAG TAACAACAAT AGCACTATGG TGATGACAGG CATGCGGATC CAGATTGGGA CTCAAGCAAT
007101 AGAACGGGCC CCGTCATATA TCGAGATCTT CGGCAGAACT ATGCAGCTCA ACCTGAGTCG CTCACGCTGG TTTGACTTCC CCTTCACCAG AGAAGAAGCC
007201 CTGCAGGCTG ATAAGAAGCT GAACCTCTTC ATTGGGGCCT CGGTGGATCC AGCAGGTGTC ACCATGATAG ATGCTGTAAA AATTTATGGC AAGACTAAGG
007301 AGCAGTTTGG CTGGCCTGAT GAGCCCCCAG AAGAATTCCC TTCTGCCTCT GTCAGCAACA TCTGCCCTTC AAATCTGAAC CAGAGCAACG GCACTGGAGA
007401 TAGCGACTCA GCTGCCCCCA CTACGACCAG TGGAACTGTC CTGGAGAGGC TGGTTGTGAG TTCTTTAGAA GCCCTGGAAA GCTGCTTTGC CGTTGGCCCA
007501 ATCATCGAGA AGGAGAGAAA CAAGAATGCT GCTCAGGAGC TGGCCACTTT GCTGTTGTCC CTGCCAGCAC CTGCCAGTGT CCAGCAGCAG TCCAAGAGCC
007601 TTCTGGCCAG CCTGCACACC AGCCGCTCGG CCTACCACAG CCACAAGGAT CAGGCCTTGC TGAGCAAAGC TGTGCAGTGT CTCAACACAT CTAGCAAAGA
007701 GGGCAAGGAT TTGGACCCTG AGGTGTTCCA GAGGCTAGTG ATCACAGCTC GCTCCATTGC CATCATGCGC CCCAACAACC TTGTCCACTT TACGGAGTCA
007801 AAGCTGCCCC AGATGGAAAC AGAAGGAATG GATGAAGGGA AGGAACCGCA GAAGCAGTTG GAAGGAGATT GCTGTAGTTT CATCACCCAG CTTGTGAACC
007901 ACTTCTGGAA ACTCCATGCA TCCAAACCCA AGAATGCCTT CTTGGCACCT GCCTGCCTTC CAGGACTAAC TCATATTGAA GCTACTGTCA ATGCTCTGGT
008001 GGACATCATC CATGGCTACT GTACCTGTGA GCTGGATTGT ATTAACACAG CATCCAAGAT CTACATGCAG ATGCTCTTGT GTCCTGATCC TGCTGTGAGC
008101 TTCTCTTGTA AACAAGCTCT AATTCGAGTC CTAAGGCCCA GGAACAAACG GAGACATGTG ACTTTACCCT CTTCCCCTCG AAGCAACACT CCAATGGGAG
008201 ACAAGGATGA TGATGACGAT GATGATGCAG ATGAGAAAAT GCAGTCATCA GGGATCCCGA ATGGTGGTCA CATCCGTCAG GAAAGCCAGG AACAGAGTGA
008301 GGTGGACCAT GGAGATTTTG AGATGGTGTC TGAGTCGATG GTCCTGGAGA CAGCTGAAAA TGTCAACAAT GGCAACCCCT CTCCCCTGGA GGCCCTGCTG
008401 GCAGGCGCAG AGGGCTTCCC CCCCATGCTG GACATCCCAC CTGATGCAGA TGACGAGACC ATGGTTGAAC TAGCCATTGC CCTGAGCCTG CAGCAGGACC
008501 AACAAGGCAG CAGCAGCAGT GCCCTGGGCC TGCAGAGCCT GGGACTGTCC GGCCAGGCAC CCAGCTCTTC CTCTCTGGAC GCAGGAACCC TCTCTGACAC
008601 CACAGCATCA GCTCCAGCCT CAGACGACGA GGGCAGTACA GCAGCGACAG ATGGTTCTAC CCTTCGGACC TCTCCTGCTG ACCACGGTGG TAGTGTGGGC
008701 TCGGAGAGCG GGGGCAGTGC AGTGGACTCA GTGGCTGGCG AGCACAGTGT ATCTGGCCGG AGCAGTGCTT ATGGCGATGC TACAGCTGAG GGGCATCCGG
008801 CTGGACCAGG AAGTGTCAGC TCAAGCACTG GAGCCATCAG CACCACCACT GGGCACCAGG AGGGAGATGG CTCCGAGGGA GAAGGAGAAG GAGAAACTGA
008901 AGGAGATGTC CACACTAGCA ACAGGCTGCA CATGGTCCGT CTAATGCTGT TGGAGAGATT ACTGCAGACC CTGCCTCAAT TACGAAACGT TGGCGGTGTC
009001 CGGGCCATCC CATACATGCA GGTCATTCTA ATGCTCACTA CAGATCTGGA TGGAGAAGAT GAGAAAGACA AGGGGGCCCT AGACAACCTG CTCTCCCAGC
009101 TTATTGCTGA GTTGGGTATG GATAAAAAGG ATGTCTCCAA GAAGAATGAG CGCAGCGCCC TGAATGAAGT CCATCTGGTA GTAATGAGAC TCCTGAGTGT
009201 CTTCATGTCC CGCACCAAAT CTGGATCCAA GTCTTCCATA TGTGAGTCAT CTTCCCTCAT CTCCAGTGCC ACAGCAGCAG CTCTACTGAG CTCTGGGGCT
009301 GTGGACTACT GCCTGCACGT GCTCAAATCA CTGCTGGAAT ATTGGAAGAG CCAACAGAAT GACGAGGAGC CTGTGGCTAC CAGCCAGTTG CTGAAACCAC
009401 ATACTACCTC CTCCCCACCT GACATGAGCC CATTCTTTCT CCGCCAGTAT GTGAAGGGTC ATGCTGCTGA TGTGTTTGAG GCCTATACTC AGCTTCTAAC
009501 AGAAATGGTA CTGAGGCTTC CTTACCAAAT CAAAAAGATT ACTGACACCA ATTCTCGAAT CCCACCTCCT GTCTTTGACC ACTCGTGGTT TTACTTTCTC
009601 TCCGAGTACC TCATGATCCA GCAGACTCCA TTTGTGCGCC GTCAAGTCCG CAAACTTCTG CTCTTCATCT GTGGATCCAA AGAGAAGTAC CGCCAGCTCC
009701 GGGATTTGCA CACCCTGGAC TCTCACGTGC GTGGGATCAA GAAGCTGCTA GAAGAGCAGG GGATATTCCT CCGGGCAAGT GTGGTTACAG CCAGCTCAGG
009801 CTCCGCCTTG CAATATGACA CACTCATCAG CCTGATGGAG CACCTGAAAG CCTGTGCAGA GATTGCCGCC CAGCGAACCA TCAACTGGCA GAAATTCTGC
009901 ATCAAAGATG ACTCCGTCCT GTACTTCCTC CTCCAAGTCA GTTTCCTTGT GGATGAGGGC GTGTCCCCAG TGCTGCTGCA ACTGCTCTCC TGTGCTCTGT
010001 GCGGCAGCAA GGTGCTCGCT GCACTGGCAG CCTCTTCGGG ATCCTCCAGT GCTTCTTCCT CCTCAGCCCC TGTGGCTGCC AGTTCTGGAC AAGCCACAAC
010101 ACAGTCCAAG TCTTCCACTA AAAAGAGCAA GAAAGAAGAA AAAGAAAAGG AGAAAGATGG TGAGACCTCT GGCAGCCAGG AGGACCAGCT GTGCACAGCT
010201 CTGGTGAACC AGCTGAACAA ATTTGCCGAT AAGGAAACCC TGATCCAGTT CCTGCGTTGT TTCCTGTTAG AGTCCAATTC TTCCTCGGTG CGCTGGCAGG
010301 CCCACTGTCT GACACTGCAC ATCTACAGAA ATTCCAGCAA ATCTCAACAG GAGCTCCTGC TAGATCTGAT GTGGTCCATC TGGCCAGAAC TCCCAGCCTA
010401 TGGTCGTAAG GCTGCCCAGT TTGTGGACCT ACTAGGATAT TTCTCCCTGA AAACTCCACA AACAGAGAAG AAGTTGAAGG AGTATTCACA GAAGGCTGTG
010501 GAGATTCTGC GGACTCAAAA CCATATTCTT ACCAACCACC CCAACTCGAA CATTTATAAC ACTTTGTCTG GCTTAGTGGA GTTTGATGGC TATTACCTGG
010601 AGAGCGATCC CTGCCTGGTG TGTAATAACC CGGAAGTACC GTTCTGTTAT ATCAAGCTGT CTTCCATTAA AGTGGACACG CGGTACACCA CCACCCAGCA
010701 GGTTGTGAAG CTCATTGGCA GTCACACCAT CAGCAAAGTG ACAGTGAAAA TCGGGGATCT GAAACGGACC AAGATGGTGC GGACCATCAA CCTGTATTAT
010801 AACAACCGAA CCGTGCAGGC CATCGTGGAG TTGAAAAACA AGCCAGCTCG CTGGCACAAA GCCAAGAAGG TTCAGCTGAC CCCTGGACAG ACAGAGGTGA
010901 AGATTGACCT GCCGTTGCCC ATTGTGGCCT CCAATCTGAT GATTGAGTTT GCAGACTTCT ATGAAAACTA CCAGGCCTCC ACAGAGACCC TGCAGTGCCC
011001 TCGCTGTAGT GCCTCGGTCC CTGCCAACCC AGGAGTCTGT GGCAACTGTG GAGAGAATGT GTACCAGTGT CACAAATGCA GATCCATCAA CTACGATGAA
011101 AAGGATCCCT TCCTCTGCAA TGCCTGTGGC TTCTGTAAAT ATGCCCGCTT CGACTTCATG CTCTATGCCA AGCCTTGCTG TGCAGTGGAT CCCATTGAGA
011201 ATGAAGAAGA CCGGAAGAAG GCTGTATCCA ACATCAATAC ACTTTTGGAC AAAGCTGATC GAGTGTATCA TCAGCTGATG GGACACCGGC CACAGCTGGA
011301 GAACCTGCTC TGCAAAGTGA ATGAGGCAGC TCCAGAAAAG CCACAGGATG ACTCAGGAAC AGCAGGGGGC ATCAGCTCCA CTTCTGCCAG TGTGAATCGT
011401 TACATCCTGC AGTTGGCTCA GGAGTATTGT GGAGACTGCA AGAACTCTTT TGATGAACTC TCCAAAATCA TCCAGAAAGT CTTTGCTTCG CGCAAAGAGT
011501 TGTTGGAATA TGACCTACAG CAGAGGGAAG CAGCCACTAA ATCATCCCGG ACCTCCGTGC AGCCCACATT CACTGCCAGC CAGTACCGTG CCTTATCCGT
011601 CCTGGGCTGT GGCCACACAT CCTCCACCAA GTGCTATGGC TGCGCCTCGG CTGTCACAGA ACATTGTATC ACACTACTTC GGGCCCTGGC CACCAACCCA
011701 GCCTTGAGGC ACATCCTTGT CTCCCAGGGC CTTATCCGGG AGCTCTTTGA TTATAATCTT CGCCGAGGGG CTGCGGCCAT GCGGGAGGAG GTCCGCCAGC
011801 TCATGTGCCT CCTAACTCGA GACAACCCAG AAGCCACCCA ACAGATGAAT GACCTGATTA TTGGCAAGGT CTCCACAGCC CTGAAGGGCC ACTGGGCCAA
011901 CCCCGATCTG GCAAGTAGCC TGCAGTATGA AATGCTGCTG CTGACGGATT CTATCTCCAA GGAGGACAGC TGCTGGGAGC TCCGGTTACG CTGTGCTCTC
012001 AGCCTTTTCC TCATGGCTGT GAACATTAAG ACTCCTGTGG TGGTTGAAAA CATTACCCTC ATGTGCCTGA GGATCTTGCA GAAGCTGATA AAACCACCTG
012101 CTCCCACTAG CAAGAAGAAC AAGGATGTCC CCGTTGAGGC CCTCACCACG GTGAAGCCAT ACTGCAATGA GATCCATGCC CAGGCTCAAC TGTGGCTCAA
012201 GAGAGACCCC AAGGCATCCT ATGATGCCTG GAAGAAGTGT CTTCCTATCA GAGGGATAGA TGGCAATGGG AAAGCCCCCA GCAAATCAGA GCTCCGCCAT
012301 CTCTATTTGA CTGAGAAGTA TGTGTGGAGG TGGAAACAGT TCCTGAGTCG TCGGGGGAAG AGGACCTCCC CCTTGGATCT CAAACTGGGG CATAACAACT
012401 GGCTGCGACA AGTGCTTTTC ACTCCAGCAA CGCAGGCCGC ACGGCAGGCA GCCTGTACCA TTGTGGAAGC TCTAGCCACC ATTCCCAGCC GCAAGCAGCA
012501 GGTCCTGGAC CTGCTTACCA GTTACCTGGA TGAGCTGAGC ATAGCTGGGG AGTGTGCAGC TGAGTACCTG GCTCTCTACC AGAAGCTCAT CACTTCTGCG
012601 CACTGGAAAG TCTACTTGGC AGCTCGGGGA GTCCTACCCT ATGTGGGCAA CCTCATCACC AAGGAAATAG CTCGTCTGCT GGCCCTGGAG GAGGCTACCC
012701 TGAGTACCGA TCTGCAGCAG GGTTATGCCC TTAAAAGTCT CACAGGCCTT CTCTCCTCCT TTGTTGAGGT GGAATCCATC AAAAGACATT TTAAAAGTCG
012801 CTTGGTGGGT ACTGTGCTGA ATGGATACCT GTGCTTGCGG AAGCTGGTGG TGCAGAGGAC CAAGCTGATC GATGAGACGC AGGACATGCT GCTGGAGATG
012901 CTGGAGGACA TGACCACAGG TACAGAATCA GAAACCAAGG CCTTCATGGC TGTGTGCATT GAGACAGCCA AGCGCTACAA TCTGGATGAC TACCGGACCC
013001 CGGTGTTCAT CTTCGAGAGG CTCTGCAGCA TCATTTATCC TGAGGAGAAT GAAGTCACTG AGTTCTTTGT GACCCTGGAG AAGGATCCCC AACAAGAAGA
013101 CTTCTTACAG GGCAGGATGC CTGGGAACCC GTATAGCAGC AATGAGCCAG GCATCGGGCC GCTGATGAGG GATATAAAGA ACAAGATTTG CCAGGACTGT
013201 GACTTAGTGG CCCTCCTGGA AGATGACAGT GGCATGGAGC TTCTAGTGAA CAATAAAATC ATTAGTTTGG ACCTTCCTGT GGCTGAAGTT TACAAGAAAG
013301 TCTGGTGTAC CACGAATGAG GGAGAGCCCA TGAGGATTGT TTATCGTATG CGGGGGCTGC TGGGCGATGC CACAGAGGAG TTCATTGAGT CCCTGGACTC
013401 TACTACAGAT GAAGAAGAAG ATGAAGAAGA AGTGTATAAA ATGGCTGGTG TGATGGCCCA GTGTGGGGGC CTGGAATGCA TGCTTAACAG ACTCGCAGGG
013501 ATCAGAGATT TCAAGCAGGG ACGCCACCTT CTAACAGTGC TACTGAAATT GTTCAGTTAC TGCGTGAAGG TGAAAGTCAA CCGGCAGCAA CTGGTCAAAC
013601 TGGAAATGAA CACCTTGAAC GTCATGCTGG GGACCCTAAA CCTGGCCCTT GTAGCTGAAC AAGAAAGCAA GGACAGTGGG GGTGCAGCTG TGGCTGAGCA
013701 GGTGCTTAGC ATCATGGAGA TCATTCTAGA TGAGTCCAAT GCTGAGCCCC TGAGTGAGGA CAAGGGCAAC CTCCTCCTGA CAGGTGACAA GGATCAACTG
013801 GTGATGCTCT TGGACCAGAT CAACAGCACC TTTGTTCGCT CCAACCCCAG TGTGCTCCAG GGCCTGCTTC GCATCATCCC GTACCTTTCC TTTGGAGAGG
013901 TGGAGAAAAT GCAGATCTTG GTGGAGCGAT TCAAACCATA CTGCAACTTT GATAAATATG ATGAAGATCA CAGTGGTGAT GATAAAGTCT TCCTGGACTG
014001 CTTCTGTAAA ATAGCTGCTG GCATCAAGAA CAACAGCAAT GGGCACCAGC TGAAGGATCT GATTCTCCAG AAGGGGATCA CCCAGAATGC ACTTGACTAC
014101 ATGAAAAAGC ACATCCCTAG CGCCAAGAAT TTGGATGCCG ACATCTGGAA AAAGTTTTTG TCTCGCCCAG CCTTGCCATT TATCCTAAGG CTGCTTCGGG
014201 GCCTGGCCAT CCAGCACCCT GGCACCCAGG TTCTGATTGG AACTGATTCC ATCCCGAACC TGCATAAGCT GGAGCAGGTG TCCAGTGATG AGGGCATTGG
014301 GACCTTGGCA GAGAACCTGC TGGAAGCCCT GCGGGAACAC CCTGACGTAA ACAAGAAGAT TGACGCAGCC CGCAGGGAGA CCCGGGCAGA GAAGAAGCGC
014401 ATGGCCATGG CAATGAGGCA GAAGGCCCTG GGCACCCTGG GCATGACGAC AAATGAAAAG GGCCAGGTCG TGACCAAGAC AGCACTCCTG AAGCAGATGG
014501 AAGAGCTGAT CGAGGAGCCT GGCCTCACGT GCTGCATCTG CAGGGAGGGA TACAAGTTCC AGCCCACAAA GGTCCTGGGC ATTTATACCT TCACGAAGCG
014601 GGTAGCCTTG GAGGAGATGG AGAATAAGCC CCGGAAACAG CAGGGCTACA GCACCGTGTC CCACTTCAAC ATTGTGCACT ACGACTGCCA TCTGGCTGCC
014701 GTCAGGTTGG CTCGAGGCCG GGAAGAGTGG GAGAGTGCCG CCCTGCAGAA TGCCAACACC AAGTGCAACG GGCTCCTTCC GGTCTGGGGA CCTCATGTCC
014801 CTGAATCAGC TTTTGCCACT TGCTTGGCAA GACACAACAC TTACCTCCAG GAATGTACAG GCCAGCGGGA GCCCACGTAT CAGCTCAACA TCCATGACAT
014901 CAAACTGCTC TTCCTGCGCT TCGCCATGGA GCAGTCGTTC AGCGCAGACA CTGGCGGGGG CGGCCGGGAG AGCAACATCC ACCTGATCCC GTACATCATT
015001 CACACTGTGC TTTACGTCCT GAACACAACC CGAGCAACTT CCCGAGAAGA GAAGAACCTC CAAGGCTTTC TGGAACAGCC CAAGGAGAAG TGGGTGGAGA
015101 GTGCCTTTGA AGTGGACGGG CCCTACTATT TCACAGTCTT GGCCCTTCAC ATCCTGCCCC CTGAGCAGTG GAGAGCCACA CGTGTGGAAA TCTTGCGGAG
015201 GCTGTTGGTG ACCTCGCAGG CTCGGGCAGT GGCTCCAGGT GGAGCCACCA GGCTGACAGA TAAGGCAGTG AAGGACTATT CCGCTTACCG TTCTTCCCTT
015301 CTCTTTTGGG CCCTCGTCGA TCTCATTTAC AACATGTTTA AGAAGGTGCC TACCAGTAAC ACAGAGGGAG GCTGGTCCTG CTCTCTCGCT GAGTACATCC
015401 GCCACAACGA CATGCCCATC TACGAAGCTG CCGACAAAGC CCTGAAAACC TTCCAGGAGG AGTTCATGCC AGTGGAGACC TTCTCAGAGT TCCTCGATGT
015501 GGCCGGTCTT TTATCAGAAA TCACCGATCC AGAGAGCTTC CTGAAGGACC TGTTGAACTC AGTCCCCTGA CCACCACACA GCAGCTGCGG CGGCGAAGAC
015601 GAAGCTGGCT TGCCTTCCAC CCTCTGTTCT CCCTCCTTGT GCATTAAGTT CCCTCCGCGG GATGCTGCAT TGTTACCCCG CCCTCCCCTC TCTCATTTTT

```
015701 CTTGGTGTGG CTTGGGGTTT TTAGGCTTCC TGTTTTATCT CGTGTGTGTG GTGCACCAGC TATGAGGTTG TCTGTAACCC AAGCCATCAA AGGGCCTGTA
015801 CATACCTAGG AGCCATGAGT TGTCCCGGCC AGCTTCATAC TTGAGTGTGC ACATCTTGAG AAATAAACAA GTGACTTAAC ACACATTGAA AA
```

DYNC1H1

```
000001 AGTCTGCGGT GGGCTAGCGG ACGGTCCGGC TTCCGGCGGC CGTTTCTGTC TCTTGCTGGC TGTCTCGCTG AGTCGCGGCC GCCTTCTCAT CGCTCCTGGA
000101 AGGTCCCGAG CGCGACACCA TGTCGGAGCC CGGGGGCGGC GGCGGCGAGG ACGGCTCGGC CGGATTGGAA GTGTCGGCCG TGCAGAATGT GGCGGACGTG
000201 TCGGTGCTGC AGAAGCACCT GCGCAAGCTG GTGCCGCTGC TGCTGGAGGA CGGCGGCGAG GCGCCGGCCG CGCTGGAGGC GGCGCTGGAG GAGAAGAGCG
000301 CCCTGGAGCA GATGCGCAAG TTCCTTTCGG ACCCGCAGGT CCACACGGTG CTGGTGGAGC GCTCCACGCT CAAAGAGGAC GTCGGTGATG AAGGAGAAGA
000401 AGAAAAAGAA TTCATTTCCT ATAACATCAA CATAGACATT CATTATGGGG TTAAATCCAA TAGCTTGGCA TTCATTAAAC GTACTCCCGT GATTGATGCA
000501 GATAAACCCG TGTCTTCTCA GCTCCGGGTC CTTACACTCA GTGAAGACTC GCCCTACGAA ACTTTGCATT CTTTCATTAG CAATGCAGTG GCTCCTTTTT
000601 TTAAGTCCTA CATTAGAGAG TCTGGCAAGG CAGACAGGGA TGGTGATAAA ATGGCTCCTT CAGTTGAAAA GAAGATTGCA GAACTCGAAA TGGGACTCCT
000701 TCACTTGCAG CAAAATATTG AAATTCCGGA GATCAGCCTG CCGATTCATC CAATGATCAC AAATGTTGCA AAACAGTGTT ATGAGCGTGG AGAAAAGCCA
000801 AAAGTTACAG ACTTTGGTGA TAAGGTTGAA GACCCAACAT TTCTTAATCA GTTACAATCT GGAGTTAACC GCTGGATCCG AGAAATTCAA AAAGTGACCA
000901 AACTGGATCG AGATCCTGCA TCAGGAACTG CCTTACAGGA AATTAGTTTT TGGCTAAACT TGGAACGTGC GTTATACCGC ATCCAGGAGA AACGGGAGAG
001001 CCCGGAAGTT CTCCTGACTC TGGATATCTT GAAACATGGC AAGCGCTTCC ATGCCACCGT CAGTTTTGAC ACTGACACAG GTCTAAAACA GGCTTTGGAA
001101 ACTGTGAATG ACTACAATCC TCTGATGAAA GATTTCCCTC TGAATGATTT GCTGTCTGCC ACGGAGCTGG ACAAAATAAG ACAGGCGCTT GTTGCCATTT
001201 TCACACATTT GAGAAAGATC CGAAACACAA AATATCCTAT TCAGAGGGCA CTGCGTTTGG TGGAGGCAAT TTCAAGAGAC TTGAGTTCTC AATTACTCAA
001301 AGTATTGGGC ACTAGGAAAT TGATGCATGT TGCTTATGAA GAATTTGAAA AAGTTATGGT AGCATGCTTT GAAGTTTTTC AGACTTGGGA TGATGAGTAT
001401 GAGAAACTTC AGGTATTGTT GAGAGACATC GTCAAAAGAA AAAGGGAAGA AAATCTGAAG ATGGTGTGGC GTATCAACCC TGCCCACAGG AAGCTGCAGG
001501 CCCGCCTTGA CCAGATGAGA AAATTTAGAC GCCAGCATGA ACAGCTAAGA GCTGTTATCG TCAGGGTCCT GAGGCCACAG GTCACGGCAG TTGCACAACA
001601 GAATCAAGGA GAGGTCCCTG AACCCCAAGA TATGAAAGTG GCTGAGGTTC TCTTTGATGC TGCAGATGCA AATGCCATTG AGGAAGTAAA CCTTGCTTAT
001701 GAGAACGTCA AGGAAGTGGA TGGACTGGAT GTTTCCAAAG AGGGCACGGA AGCCTGGGAG GCTGCTATGA AGAGGTACGA TGAGAGGATC GACAGAGTGG
001801 AGACCCGGAT CACCGCTCGC CTTCGGGATC AGCTTGGCAC AGCCAAGAAT GCCAACGAGA TGTTTAGGAT TTTCTCCAGG TTTAATGCAC TGTTTGTCAG
001901 GCCTCACATC CGTGGGGCCA TTCGCGAATA CCAGACCCAG CTGATCCAGC GCGTGAAAGA TGACATTGAG TCTCTTCACG ACAAGTTCAA GGTCCAGTAC
002001 CCACAGAGTC AGGCTTGTAA GATGAGTCAC GTTCGTGACT TGCCCCCTGT GTCAGGGTCT ATCATCTGGG CTAAACAGAT CGACAGGCAG CTGACGGCCT
002101 ACATGAAGCG GGTGGAAGAT GTCCTTGGCA AGGGCTGGGA GAATCACGTG GAGGGGCAGA AGCTGAAGCA GGATGGAGAC AGCTTCCGCA TGAAGCTCAA
002201 CACGCAGGAG ATCTTTGATG ACTGGGCAAG GAAGGTGCAG CAGCGCAACC TCGGTGTCTC GGGGCGCATT TTCACCATCG AAAGTACTCG GGTTCGGGGC
002301 CGAACTGGAA ATGTGCTTAA GCTGAAAGTT AACTTTCTTC CTGAGATTAT CACACTATCC AAAGAAGTCC GGAACCTCAA ATGGCTTGGT TTCCGCGTCC
002401 CACTGGCGAT TGTGAACAAA GCCCATCAAG CAAACCAGCT TTACCCGTTT GCCATCTCAC TGATCGAGAG CGTTCGTACC TATGAACGGA CCTGCGAGAA
002501 GGTGGAGGAG CGGAACACCA TTTCCCTTTT GGTGGCTGGC TTGAAAAAGG AAGTGCAGGC CCTGATCGCA GAAGGCATTG CGTTGGTGTG GGAGTCCTAC
002601 AAACTTGACC CATATGTACA GCGCTTAGCA GAGACTGTCT TCAACTTCCA AGAAAAGGTG GATGATCTGC TGATCATTGA AGAAAAAATA GACCTAGAAG
002701 TCCGTTCCTT GGAAACTTGT ATGTATGACC ATAAGACATT CTCGGAAATC TTGAACAGAG TCCAGAAAGC AGTGGATGAC TTAAATCTGC ACTCCTATTC
002801 CAATTTGCCC ATCTGGGTCA ACAAGCTTGA CATGGAGATT GAAAGAATAT TGGGCGTCCG TCTGCAAGCT GGCCTGAGAG CTTGGACGCA GGTTCTTCTT
002901 GGACAAGCTG AAGATAAAGC AGAAGTTGAC ATGGACACAG ATGCTCCACA AGTTAGTCAC AAGCCTGGTG GAGAGCCAAA GATCAAAAAT GTCGTTCATG
003001 AGCTAAGAAT AACCAATCAG GTAATCTACT TGAATCCACC AATTGAAGAG TGCAGATACA AGCTGTATCA GGAAATGTTT GCCTGGAAGA TGGTTGTACT
003101 GTCTCTCCCC AGGATCCAGA GTCAGAGGTA CCAGGTGGGT GTACATTACG AATTGACTGA GGAAGAGAAA TTCTATCGGA ATGCTTTAAC ACGGATGCCT
003201 GATGGCCCTG TTGCCCTGGA AGAGTCGTAT TCTGCTGTCA TGGGCATTGT ATCTGAAGTT GAACAGTATG TCAAGGTTTG GCTTCAGTAT CAGTGTTTAT
003301 GGGATATGCA AGCTGAAAAC ATCTATAACA GACTTGGAGA AGATCTCAAC AAATGGCAGG CTCTCCTGGT CCAAATAAGG AAGGCCAGAG GAACCTTTGA
003401 CAATGCAGAA ACCAAGAAAG AGTTTGGACC AGTAGTTATA GATTATGGCA AGGTACAATC TAAGGTGAAC TTGAAATATG ACTCTTGGCA TAAGGAGGTT
003501 CTTAGCAAAT TTGGGCAGAT GCTAGGATCA AACATGACGG AATTCCATTC CCAGATCTCA AAGTCCCGCC AAGAGTTGGA GCAGCACTCA GTAGACACGG
003601 CCAGCACCTC CGATGCAGTG ACCTTCATCA CCTATGTGCA GTCTTTGAAA CGGAAGATCA AGCAGTTTGA GAAGCAAGTT GAGCTCTACC GCAATGGCCA
003701 GCGCTTACTG GAAAAGCAAA GGTTCCAGTT CCCACCTTCC TGGCTTTATA TTGACAACAT CGAGGGAGAG TGGGGAGCCT TCAATGACAT CATGCGGCGA
003801 AAGGACTCTG CCATTCAGCA GCAGGTGGCA AACCTGCAAA TGAAGATTGT CCAGGAGGAT CGGGCCGTGG AAAGCCGCAC CACCGACCTG CTGACTGACT
003901 GGGAGAAGAC CAAGCCTGTC ACGGGCAACC TTCGCCCAGA AGAGGCACTT CAGGCTCTCA CCATATATGA GGGGAAGTTT GGTAGGCTGA AGGACGACAG
004001 AGAGAAGTGT GCAAAGGCCA AGGAGGCGCT GGAATTGACA GATACTGGGC TTCTCAGTGG CAGTGAAGAG CGCGTGCAGG TGGCCTTAGA AGAATTACAG
004101 GACCTCAAAG GCGTTTGGTC AGAACTTTCT AAGGTTTGGG AGCAAATCGA TCAGATGAAG GAGCAACCCT GGGTTTCAGT ACAGCCTCGA AAGCTTCGAC
004201 AAAATTTGGA TGCCCTCCTG AACCAGCTGA AAAGCTTCCC TGCCCGGTTG CGACAGTATG CGTCCTATGA GTTTGTTCAG AGGCTTCTGA AAGGTTACAT
004301 GAAGATAAAT ATGCTGGTGA TTGAACTGAA ATCCGAAGCA CTTAAAGACC GCCATTGGAA ACAGCTCATG AAAAGGCTTC ACGTTAATTG GGTTGTTTCT
004401 GAGCTAACCC TTGGCCAAAT CTGGGATGTT GACTTGCAGA AAAATGAAGC GATTGTCAAG GATGTACTGC TTGTGGCACA AGGGGAGATG GCTTTGGAAG
004501 AATTTTTGAA GCAGATAAGA GAAGTGTGGA ATACTTATGA ACTAGACTTG GTTAATTATC AGAACAAGTG CCGCTTGATC CGTGGCTGGG ATGACCTCTT
004601 CAACAAGGTC AAAGAACACA TCAACAGCGT CTCGGCCATG AAGCTCTCTC CGTATTACAA GGTTTTTGAA GAGGATGCTC TCAGCTGGGA AGATAAGCTG
004701 AACAGGATCA TGGCTCTCTT TGATGTGTGG ATTGATGTGC AGAGGCGGTG GGTCTACCTG GAAGGTATCT TCACAGGCAG TGCAGATATC AAGCACCTGC
004801 TGCCAGTGGA AACCCAGCGG TTTCAGAGCA TCAGCACTGA GTTTTTGGCT CTAATGAAAA AAGTGTCCAA GTCTCCCCTT GTTATGGATG TTCTGAACAT
004901 CCAGGGAGTA CAGAGGTCTC TGGAAAGATT GGCAGACCTG CTAGGAAAGA TCCAGAAAGC ATTGGGAGAA TATCTGGAAA GAGAGCGGTC ATCTTTCCCC
005001 AGGTTCTATT TTGTGGGTGA TGAAGATTTG CTTGAAATCA TTGGAAACAG CAAGAATGTC GCTAAATTAC AGAAACACTT CAAGAAGATG TTTGCTGGAG
005101 TTTCGAGCAT CATCCTGAAC GAGGATAACT CTGTTGTTTT GGGTATTTCA TCTCGGGAAG GAGAGGAGGT TATGTTTAAA ACTCCTGTGT CAATTACTGA
005201 ACATCCCAAA ATCAATGAGT GGCTCACATT GGTAGAAAAG GAGATGAGAG TCACCCTGGC CAAACTGCTT GCTGAGTCTG TTACGGAAGT TGAGATTTTT
005301 GGTAAAGCAA CTTCAATTGA CCCAAATACC TACATCACTT GGATTGATAA ATACCAGGCC CAGCTTGTGG TTTTGTCAGC CCAGATAGCC TGGTCTGAGA
005401 ACGTGGAGAC CGCACTGAGC AGCATGGGCG GAGGTGGAGA TGCCGCGCCC TTGCACTCTG TGCTGAGCAA TGTGGAGGTC ACCCTCAATG TGTTAGCAGA
005501 CTCTGTCCTC ATGGAGCAGC CCCCACTCCG AAGGCGGAAG CTAGAACACT TGATTACAGA GTTGGTTCAC CAGAGAGATG TTACAAGGTC CTTGATCAAA
005601 AGCAAGATTG ACAACGCCAA ATCTTTTGAA TGGCTCAGCC AGATGCGATT TTACTTTGAC CCTAAGCAAA CTGATGTGTT ACAGCAGTTG TCAATTCAAA
005701 TGGCAAATGC CAAATTTAAC TATGGCTTTG AGTACCTGGG TGTTCAGGAC AAACTGGTCC AGACCCCCCT CACTGACCGC TGCTATTTGA CAATGACACA
005801 AGCCTTGGAG GCCAGGCTGG GGGGTTCCCC ATTTGGACCT GCTGGAACTG GGAAAACAGA GTCTGTCAAA GCTCTTGGCC ATCAGCTTGG ACGGTTTGTT
005901 TTAGTTTTCA ACTGTGATGA AACCTTTGAT TTCCAGGCAA TGGGCCGGAT CTTTGTGGGC CTTTGCCAGG TGGGTGCCTG GGGCTGCTTT GACGAGTTCA
006001 ACCGCCTGGA GGAGCGGATG CTCTCGGCTG TGTCCCAGCA GGTGCAGTGC ATACAGGAAG CACTGCGTGA ACATTCCAAC CCCAACTACG ACAAGACCTC
006101 TGCCCCCATT ACTTGTGAGC TGCTGAACAA ACAAGTCAAG GTGAGCCCGG ACATGGCCAT CTTCATCACC ATGAACCCTG GCTACGCGGG CCGGTCTAAC
006201 CTTCCTGACA ACTTGAAGAA GCTGTTCCGG AGCTTGGCCA TGACCAAGCC CGACCGGCAG TTAATCGCCC AGGTCATGCT GTACTCACAG GGTTTCCGCA
006301 CTGCTGAAGT GCTTGCCAAC AAAATCGTCC CGTTTTTTAA ACTATGCGAT GAGCAGCTCT CTTCCCAAAG CCATTATGAC TTCGGTCTTC GGGCTTTGAA
006401 GAGTGTGCTG GTGAGTGCAG GCAATGTGAA GAGAGAGAGA ATCCAGAAGA TAAAGAGGGA GAAAGAGGAA CGAGGGGAAG CAGTTGATGA AGGAGAAATT
006501 GCTGAAAATC TCCCTGAACA AGAGATTCTG ATACAGAGCG TCTGTGAGAC GATGGTGCCA AAGCTGGTGG CAGAGGACAT CCCGCTGCTC TTCAGCCTCC
006601 TGTCGGACGT GTTCCCTGGA GTCCAGTATC ACAGGGGTGA GATGACTGCC CTTCGAGAGG AGCTGAAGAA AGTGTGTCAG GAGATGTATT TGACATATGG
006701 AGATGGAGAA GAAGTTGGTG GAATGTGGGT TGAAAAGGTT CTCCAGCTCT ATCAGATCAC CCAGATCAAT CATGGCCTGA TGATGGTGGG GCCCTCGGGA
006801 AGTGGGAAGA GCATGGCCTG GCGTGTCCTG CTGAAGGCAT TGGAGAGACT CGAGGGTGTG GAAGGTGTGG CCCATATCAT CGACCCCAAG GCCATCAGCA
006901 AAGACCACCT CTACGGAACC CTGGACCCCA ACACCAGGGA ATGGACAGAT GGGCTCTTCA CACACGTGCT GAGAAAGATC ATCGACAGCG TGAGAGGCGA
007001 GCTGCAGAAG CGCCAGTGGA TCGTCTTCGA TGGCGATGTG GATCCAGAGT GGGTTGAGAA CTTGAACTCA GTGCTGGATG ACAATAAGCT CCTAACTTTG
007101 CCCAATGGAG AGCGCCTCAG TCTTCCACCC AATGTGAGAA TAATGTTTGA GGTACAGGAC TTGAAATACG CGACCTTGGC CACAGTGTCG CGCTGCGGCA
007201 TGGTCTGGTT CAGTGAGGAT GTGCTGAGCA CCGACATGAT CTTCAACAAC TTCCTGGCCA GGCTGCGCAG CATCCCGCTG GATGAAGGGG AGGATGAGGC
007301 ACAGCGGCGG CGTAAGGGCA AAGAGGATGA GGGGGAGGAG GCCGCTTCCC CCATGCTGCA GATCCAAAGA GATGCAGCTA CGATCATGCA ACCGTACTTC
007401 ACGTCCAACG GCCTGGTCAC CAAGGCGCTA GAGCACGCCT TCCAGCTGGA GCACATCATG GACCTAACAC GCCTGCGCTG CCTGGGCTCG CTCTTCTCCA
007501 TGCTGCACCA GGCCTGCCGC AACGTGGCGC AGTATAACGC CAACCATCCC GACTTCCCCA TGCAGATCGA GCAGCTGGAG CGCTACATTC AGCGATATCT
007601 GGTTTATGCC ATACTCTGGT CCCTGTCTGG AGACAGCCGG CTAAAAATGA GAGCAGAGCT GGGTGAATAC ATCAGAAGAA TCACGACCGT GCCTCTGCCC
007701 ACTGCGCCCA ACATACCCAT TATCGATTAT GAGGTGTCCA TCAGCGGAGA ATGGTCTCCG TGGCAGGCCA AGGTGCCTCA GATTGAAGTG GAGACGCACA
007801 AGGTGGCAGC CCCTGATGTC GTCGTGCCAA CGCTGGACAC AGTCCGCCAC GAAGCCCTCT TGTACACTTG GCTGGCCGAA CACAAGCCCC TGGTCTTGTG
007901 TGGCCCTCCT GGGTCTGGCA AGACCATGAC ACTCTTCAGC GCCCTCCGGG CCTTGCCTGA CATGGAGGTG GTGGGTCTCA ACTTCTCCAG TGCTACTACT
008001 CCAGAGCTGC TTCTGAAGAC TTTTGATCAC TACTGCGAGT ACAGGCGCAC ACCTAATGGG GTGGTTTTGG CTCCTGTTCA ACTTGGAAAG TGGCTGGTGT
008101 TGTTCTGTGA TGAAATCAAC TTGCCAGATA TGGATAAATA TGGGACCCAG AGGGTCATAT CCTTCATCAG ACAGATGGTG GAGCACGGAG GCTTTTACCG
008201 TACCTCAGAT CAAACATGGG TGAAGCTGGA GAGAATCCAG TTTGTTGGGG CTTGTAATCC CCCCACAGAC CCTGGAAGAA AGCCCCTCTC ACACAGGTTC
008301 CTGCGCCACG TGCCTGTCGT GTATGTGGAT TACCCGGGCC CCGCCTCCCT CACACAGATC TACGGCACCT TCAACCGCGC CATGCTGAGG CTCATTCCAT
```

008401 CCCTGCGGAC GTATGCAGAG CCGCTCACTG CTGCCATGGT GGAGTTCTAC ACCATGTCTC AGGAGAGATT CACCCAGGAT ACACAACCTC ACTATATCTA
008501 TTCACCCCGT GAAATGACTA GGTGGGTGAG AGGCATCTTT GAAGCGCTGA GACCTCTGGA GACCCTGCCT GTTGAAGGCC TCATTCGGAT TTGGGCACAT
008601 GAAGCTCTGC GTCTCTTCCA AGATAGACTC GTAGAGGATG AGGAGAGGCG TTGGACTGAT GAGAACATCG ACACGGTTGC TCTGAAGCAC TTCCCTAACA
008701 TCGACAGAGA GAAGGCAATG AGCCGACCCA TCTTGTACAG CAACTGGCTG TCAAAGGATT ACATCCCAGT AGACCAAGAA GAGTTAAGAG ATTATGTCAA
008801 AGCTAGGCTG AAGGTCTTTT ATGAAGAAGA ACTTGATGTT CCGCTGGTGC TGTTTAATGA AGTCCTAGAC CACGTGCTGA GGATTGACAG AATATTCCGT
008901 CAACCTCAAG GCCACTTGCT TCTGATTGGT GTTAGTGGAG CAGGAAAAAC TACCCTGTCT CGTTTCGTCG CCTGGATGAA CGGTTTGAGT GTGTACCAGA
009001 TTAAGGTCCA TAGGAAGTAC ACAGGGGAAG ACTTTGATGA AGATCTACGG ACAGTGTTGA GACGTTCTGG CTGTAAAAAT GAAAAGATAG CATTTATAAT
009101 GGATGAATCT AATGTGTTAG ATTCTGGATT CCTGGAGCGA ATGAATACCC TTCTGGCCAA TGGAGAGGTG CCTGGTCTCT TTGAAGGAGA CGAGTATGCC
009201 ACCTTGATGA CGCAGTGCAA AGAGGGGGCA CAGAAGGAAG GCCTGATGCT GGACTCGCAC GAGGAGCTCT ACAAGTGGTT CACTAGCCAG GTTATCCGCA
009301 ACCTCCACGT CGTGTTCACC ATGAACCCGT CCTCGGAGGG ACTCAAGGAC CGGGCAGCTA CATCACCAGC ACTTTTCAAC AGGTGTGTGT TGAATTGGTT
009401 TGGAGACTGG TCCACCGAAG CACTGTATCA GGTTGGCAAA GAATTCACAA GTAAGATGGA TCTGGAGAAG CCAAATTACA TCGTGCCTGA TTACATGCCA
009501 GTTGTGTATG ATAAGCTGCC GCAGCCACCA TCCCATCGGG AAGCCATTGT GAACAGCTGT GTGTTTGTTC ATCAGACTCT TCACCAGGCG AATGCTCGGC
009601 TAGCAAAGCG AGGCGGCAGA ACGATGGCCA TCACCCCTCG CCACTACCTG GACTTCATCA ATCACTATGC CAACCTGTTC CACGAGAAGC GGAGCGAGCT
009701 GGAGGAGCAG CAGATGCACT TGAACGTGGG GCTCAGGAAG ATCAAAGAGA CAGTCGACCA GGTAGAAGAA CTGCGTCGTG ACTTGAGGAT AAAGAGCCAA
009801 GAGCTGGAGG TGAAGAATGC AGCAGCCAAT GACAAGCTGA AAAAGATGGT GAAAGACCAG CAGGAGGCTG AAAAGAAGAA GGTTATGAGC CAAGAAATCC
009901 AGGAACAGCT GCATAAGCAG CAGGAGGTAA TTGCAGACAA ACAGATGAGT GTCAAAGAAG ATCTTGATAA GGTGGAACCT GCCGTCATTG AGGCCCAGAA
010001 TGCTGTGAAG TCGATCAAGA AGCAGCACCT GGTGGAGGTG AGGTCCATGG CCAACCCTCC TGCTGCTGTG AAGCTGGCGC TGGAGTCCAT CTGCCTGCTG
010101 CTGGGGGAAA GCACCACAGA CTGGAAGCAG ATCCGCTCCA TCATCATGCG GGAGAACTTC ATCCCCACCA TCGTCAACTT CTCTGCAGAG GAGATCAGTG
010201 ACGCCATAAG GGAGAAGATG AAGAAAAATT ACATGTCCAA TCCAAGTTAC AATTATGAAA TTGTGAATCG GGCTTCCCTG GCTTGCGGCC CTATGGTGAA
010301 ATGGGCAATT GCACAGCTTA ACTATGCAGA CATGTTAAAG AGAGTGGAGC CCCTACGCAA TGAGCTGCAG AAGCTGGAAG ATGACGCCAA GGACAACCAG
010401 CAGAAGGCCA ACGAGGTGGA GCAGATGATC CGAGACCTGG AAGCCAGCAT CGCCCGCTAC AAGGAGGAAT ACGCCGTCCT GATCTCAGAG GCCCAGGCCA
010501 TCAAGGCAGA CCTGGCAGCT GTCGAGGCAA AAGTAAACCG GAGCACTGCT CTTCTGAAGA GCTTGTCTGC TGAACGTGAA CGATGGGAAA AAACAAGTGA
010601 AACTTTCAAA AACCAGATGT CCACCATTGC TGGGGACTGT CTCTTGTCAG CTGCGTTCAT TGCCTACGCG GGTTACTTTG ACCAGCAGAT GCGTCAGAAC
010701 TTGTTCACTA CCTGGTCCCA TCACCTACAG CAAGCCAACA TCCAGTTCCG TACAGATATT GCCAGGACGG AATACCTTTC CAATGCTGAT GAGCGTCTTC
010801 GCTGGCAGGC CAGCTCCTTG CCTGCTGATG ACCTTTGCAC AGAAAATGCC ATCATGCTGA AACGATTCAA TAGGTATCCG CTGATCATTG ACCCCTCTGG
010901 ACAGGCCACA GAATTCATTA TGAATGAATA TAAGGATCGT AAGATCACAC GGACCAGCTT CCTGGATGAC GCCTTCAGAA AGAACTTAGA GAGTGCACTG
011001 AGATTCGGTA ACCCCCTTCT GGTCCAGGAT GTGGAAAGCT ACGATCCAGT TTTGAACCCG GTGCTGAACC GTGAAGTGCG GCGAACAGGG GGGAGAGTGC
011101 TGATCACTCT CGGGGACCAG GACATAGACC TGTCGCCATC GTTTGTCATC TTCCTGTCCA CCCGGGATCC AACTGTCGAG TTCCCACCAG ATCTCTGTTC
011201 CCGGGTTACT TTTGTAAACT TCACAGTTAC CCGTAGCAGT TTACAAAGCC AGTGTCTAAA TGAAGTACTT AAAGCAGAAA GACCTGATGT GGACGAGAAA
011301 CGATCTGATC TTCTTAAACT TCAAGGGGAA TTTCAGCTCC GTTTGCGTCA GCTGGAAAAA TCTCTACTAC AAGCTCTGAA CGAGGTGAAA GGGCGCATTT
011401 TGGATGACGA CACGATCATA ACCACTCTGG AGAACCTGAA GAGAGAGGCT GCAGAGGTCA CCAGGAAAGT TGAGGAGACG GACATTGTCA TGCAGGAGGT
011501 GGAGACCGTG TCCCAGCAGT ACCTCCCGCT CTCCACCGCC TGCAGCAGCA TCTACTTCAC CATGGAGTCC CTCAAGCAGA TACACTTCTT GTACCAGTAC
011601 TCCCTCCAGT TTTTCCTGGA CATTTATCAC AACGTCCTAT ACGAGAACCC GAACCTGAAG GGTGTCACCG ACCACACACA GCGCCTGTCC ATTATAACAA
011701 AGGACCTCTT CCAGGTGGCG TTTAACCGAG TGGCTCGAGG CATGCTGCAT CAGGACCACA TTACCTTTGC CATGCTGCTG GCAAGAATCA AACTGAAGGG
011801 CACCGTGGGG GAGCCCACCT ACGATGCAGA ATTCCAGCAC TTCTTGAGAG GAAATGAGAT TGTCCTGAGT GCTGGCTCCA CCCCCAGGAT CCAGGGCCTG
011901 ACTGTGGAGC AGGCGGAGGC GGTGGTGAGG CTGAGCTGCC TTCCCGCGTT TAAGGACTTG ATTGCAAAGG TTCAGGCAGA CGAGCAATTT GGCATCTGGC
012001 TGGACAGCAG CTCCCCGGAG CAGACTGTGC CCTACCTCTG GAGTGAAGAA ACACCTGCAA CACCCATTGG CCAGGCCATC CACCGCCTGC TCCTGATCCA
012101 GGCTTTCCGG CCCGATCGCC TGTTGGCCAT GGCCCACATG TTTGTTTCAA CAAACCTTGG GGAGTCTTTC ATGTCCATCA TGGAGCAGCC GCTCGACCTG
012201 ACCCACATTG TGGGCACAGA GGTGAAGCCC AACACTCCTG TCTTAATGTG CTCTGTGCCT GGTTATGATG CCAGTGGACA TGTCGAGGAC CTTGCAGCCG
012301 AGCAGAACAC GCAGATCACT TCAATTGCAA TCGGCTCTGC AGAAGGCTTT AACCAAGCAG ATAAGGCAAT AAACACCGCT GTAAAGTCGG GCAGGTGGGT
012401 GATGCTGAAG AATGTGCATC TGGCCCCAGG GTGGCTGATG CAGCTGGAGA AGAAGTTGCA TTCCCTGCAG CCGCATGCCT GCTTCCGACT CTTCCTCACC
012501 ATGGAGATCA ACCCCAAGGT GCCTGTGAAT CTGCTCCGTG CGGGCCGCAT CTTTGTGTTC GAGCCACCGC CAGGGGTGAA GGCCAACATG CTGAGGACGT
012601 TCAGCAGCAT TCCCGTCTCA CGGATATGCA AGTCTCCCAA CGAGCGTGCC CGCTTGTACT TCCTGCTGGC CTGGTTTCAT GCGATCATCC AAGAACGCTT
012701 ACGATACGCA CCACTGGGGT GGTCAAAGAA GTATGAATTT GGAGAGTCTG ACCTGCGGTC AGCTTGCGAT ACGGTGGACA CGTGGCTGGA TGACACGGCC
012801 AAGGGCAGGC AGAACATCTC ACCGGATAAG ATCCCGTGGT CTGCACTAAA GACCTTAATG GCCCAGTCCA TTTATGGCGG GCGCGTGGAC AACGAGTTTG
012901 ACCAGCGTCT GCTCAACACC TTCCTGGAGC GCCTGTTCAC AACCAGGAGT TTCGACAGTG AGTTTAAGCT GGCATGCAAG GTCGACGGAC ATAAAGACAT
013001 TCAAATGCCA GATGGCATCA GGCGAGAGGA GTTTGTGCAG TGGGTGGAGT TGCTCCCCGA CACCCAGACG CCCTCCTGGC TGGGCCTGCC CAACAACGCC
013101 GAGAGAGTCC TCCTTACCAC ACAGGGTGTG GACATGATCA GTAAAATGCT GAAGATGCAG ATGTTGGAGG ATGAGGACGA CCTGGCCTAC GCAGAGACTG
013201 AGAAGAAGAC GAGGACAGAC TCCACGTCCG ACGGGCGCCC TGCCTGGATG CGGACACTGC ACACCACCGC GTCCAACTGG CTGCACCTCA TCCCCCAGAC
013301 GCTGAGCCAC CTCAAGCGCA CCGTGGAGAA TATCAAGGAT CCTTTGTTCA GGTTCTTTGA GAGAGAAGTG AAGATGGGCG CAAAGCTGCT TCAGGACGTT
013401 CGCCAGGACC TTGCAGATGT CGTCCAGGTG TGCGAAGGAA AGAAGAAGCA GACCAACTAC TTGCGCACGC TGATCAACGA GCTAGTGAAA GGGATCTTGC
013501 CTCGGAGCTG GTCCCACTAC ACGGTGCCTG CCGGCATGAC CGTCATCCAG TGGGTGTCCG ACTTCAGCGA GAGGATCAAA CAGCTGCAGA ACATCTCACT
013601 GGCAGCTGCA TCTGGTGGCG CCAAGGAGCT AAAGAACATC CACGTGTGCC TGGGTGGCCT GTTCGTGCCT GAGGCGTACA TCACTGCCAC CAGGCAGTAT
013701 GTGGCCCAGG CCAACAGCTG GTCCCTGGAG GAGCTCTGCC TGGAAGTCAA CGTCACCACC TCACAGGGCG CCACCCTTGA CGCTTGCAGC TTCGGAGTCA
013801 CGGGTTTGAA ACTTCAAGGG GCCACGTGCA ACAACAACAA GCTGTCACTG TCCAATGCCA TCTCAACCGC CCTTCCCCTG ACGCAGCTGC GCTGGGTCAA
013901 GCAGACAAAC ACCGAGAAGA AGGCCAGTGT GGTAACCTTA CCTGTCTACC TGAACTTCAC CCGTGCAGAC CTCATCTTCA CCGTGGACTT CGAAATTGCT
014001 ACAAAGGAGG ATCCTCGCAG CTTCTACGAG CGGGGTGTCG CAGTCTTGTG CACAGAGTAA ACTTTTCTAG CTGCCCCTTT CTGTAATAGT GAAAGTTGGT
014101 ATTTAACATT TATTCATTTT TAAAATATTT GGAAGGTCTG AGCTTGTGAA AAGAAAGTGG TTGGTCTGAG GTTGGAGGAA GCTGAATGGA ATCTGACGGT
014201 TGGGAGTGGT GGAAATTGGA AGGATACCAG GAGGTATTTG GGAAGGCCAA TGGCGTGGCT CCTTTGAGGA AATAAAACAC TAAGCATGAG CCGGCTCCGC
014301 CTCTTCTGTC TCCGCTTTCA TCCCAGGGCA CAGAGCCTTG CCTTCCATGC TGCCCAGGGA GGGCAGCCCA CGGCAGCCAT GCCCCTCCCC ACCTCGCTTT
014401 CATCATGAGC TCGCTCCCGA GCGGCCACAG CACTCATGAA TGAAGACCTT GGGGCCCTTC ACAGACACAG ATGCAGCCAG CTGTGGCTCT GAAGGCCCTG
014501 GGGCCCGGGC ACCATGGTTC ACACCTTTAA TCGCAGCACT TTGGGAGTCT GGGAGTTAAA GACCAGCCTC GGCAACATAG TGAGACCCCG TCTCTACAGG
014601 AAATTAAATC AGGTGTGGTG GTGCATGCCT GTAGTCCCAG CTACTTGAGG GCTGAGGTGG GAGGATCACC CAAGCCCAAG AGGTCGAGGC TGCAGTGAGC
014701 TGTGATCTCA CCACTGCACT CCAGCCTGGG TGACAGAGCA AGACCCTGTC TCAAAAAAAA AAAAAGCTGG GCGTGGTGGC TCATGCCTGT AATCCCAGCA
014801 CTTTGGGAGG CCGAGGCGGG CGGATCACCA AATTAGCCGG GCATGGTGGC ACATGCCTGT AATCCCAGCT ACTCGGGAGG CTGAGGCAGG AGAATTGCTT
014901 GAACCTGGGA GGCGGAGGTT GCAGTGAGCT GAAAAAAAAA AAGGCAGCCC CCAGCCGCTT GTGTTCTTGA CCAGGGCCCC AGGACTTGGC TCCTCCAGAC
015001 AAGGGAGTTT TGTGCTGTAG ATGAGGGAGT TGCCCATCGC CGCCCTAGCA AGTCCATTCC CACACGACCT TTCCAGTGGT GATGATGACA GTGGCCCATA
015101 CAGCTGACTG TTTGCATCTC ACGTTCACAT TGCTAGAGGT GATGGGTGTG CTACACCCGT GGAAACAGGC TTCTGGCATC TCAGTGTCTT TATTTTATTT
015201 TTGAGATGGA GTTTCGCTCT TGTTGCCCAG GCTGGAGTGC AGTGGCGTGA TCTCAGCTCA CTGCAACCTC CACCTACCGG GTTCAAGTGA TCCTCCTGCC
015301 TCGGCCTCCT GAATAGCTGG GATTACAGGC ATGCACCACC ATGCCCAGCT AATTACTATA TTTTTAGTAG AGACAGGGCT TCACCGTGTT GGCCAGGCTG
015401 GTCTCAAACT CCTGGCCTCA GCCAGTGATC CTCCTGCCTC AGCCTCCCAA AGTGCTGAGA TGACAGGCAT GAGCCACTGC ACCTGGCCAA ATTTTTGTAT
015501 TTTTTGTAGA GATGGAATCT CGCTATTTTC CCTGGGCTGG TCTCAAACTC CTGGCATTAA GGGCTCCTCC TATCTCAGCC TCTCAAAGTG CCGGGGTTCT
015601 AGGTGTGAGT CACCGAGTTG AGCCCCTAAA CACATGTTTT TCTTTTTAGA GACAGGGTCT CACTCGGTTG CCCAGGCTGG AGTGCAGTGG CGTGACCATG
015701 GCTCACTGCA GCCTCAACCT CCCGGGCTCA AGTGATCCTC CCACCTCAGC CTTCTGAGCA GCTGGGACCA CAGACACACA CCACCATGTC GGCTAATTTT
015801 TGTATCTTTT GTAGAGACAA GGTTTCACCA TGTTGCTCGG GCTGGTCTCG AACTCCTGAG CTCAAGTGGT CCTCCCACCT TGGCCTCCCA AAATGCTGGG
015901 ATTACAAGTG TGAGCCACCA CACCCAGCCC CTAAAAACAT TTATGTGCAT CGACATCAGC TTTGATCAGA AGAGCCCCTG CTCCTCTTGG GCTGGGACCC
016001 CTTCCTGGAC TGAGTCTGCT CACCTTGGAT TAGGCCACCT ACTGCTTCTT CCTCTTCCTT TTCTCAGGCT CTGCAAGGAA GAGCTCCAGC TTCCTCTTGG
016101 AGAAGGAGAT TCTGGGCCCC TTTTCTCTCT CCCAAACCTA GGTGGTGGCC ATGCCCCTCG AGCTCTGCTT GGCACGTGTC TGCCAGTCTC AGGGGGCTCC
016201 ATGGGAGTGA GGAAGGGCTC GGCGGCCCTG GGGGTCTGGT CCTCCTCCGC CATCCTCGAG TCCTCGGGCT ACGTGCCCTC ATCTTTGGCT TCCAGGGACA
016301 CGGGCCCAGC TGGCCACTTC TATGACAAGA ATGTGGGTTT TTTGTGACAG GGCGTCTACT ATATGAACTT CCTCTGGACT CTGACTTCAG TGTGTAGGAA
016401 GCCACCTTAC AGCTCATGTC ACCCAGAGAA CAGTCGTTGC AGCTCCAGTT GGAATGACTG GGGGTGTCTT CCGAATCGCC ATCATAAAGA TCTAAAAGCT
016501 GAGGACAAAA GTGTTTCTCA GTCCAAGAGC AGTTCTGCGA GGCGGAATCA AGTCTAACAT GCTCGCATGC GCTGACTCTT CCTCCCGTCA CTGATGCTGG
016601 TTTTTGCAGG CTCTGCCTTG CAAATGAATT TTTCTTTCTT TTTTTTTTTT TTTTTGAGAC GGAATCTTGC TCTGTCGCCC AAGCTGGAGT GCAGTGGCGC
016701 AATCTGGGCT CACTGCAACC TCCACCTCCC GGATTCAAGC GATTCTCCTG CCTCAGCCTC CTGAGTAGCT GGGACTACAG GCGCCCGCTA ATTTTTGTAT
016801 TTTTAGTAGA GACGGGGTTT CACCATATTG GTCAGGCTGG TCTTGAACTC CTGACTCAGG TGATCCACCC GCCTCAGCCT CCCAAGGTGC TGGGATTACA
016901 GGTGTGAGCC ACCACGCCTG GCCGAATTTT TATTTGTTTT TTGTTTGTTT TTGTTTTTTT TAAAAAACTG AGTCTCGCAC TGTTGTCCAG GCTGGAGTGC
017001 AGTGGCGCGA TCTCGGCTCA CTGCAAGCTC CGCCTCCCAG GTTCACGCCA TTCTCCTGCC TCAGCCTCCC AAGTAGCTGG GACTACAGGC GCCCACCACC
017101 AAGCCCGGCT AATTTTTTGG ATTTTTAGTA GAGACAGGGT TTCACTGTGT TAGCCAGGAT GGTCTCGCTC TCCTGACCTC GTGATCCACC CGCCTCCCAA

```
017201 AGTGCTGAGA TTACAGGCGT GAGCCACCAC ACTCGGCCTC TTTGTTTGTT TTTTTTTTTT TTTGAGACAG TCTGGCTCTG TCACTGAGGC TGGAGTGCAG
017301 TGATGCAACC TCATCTCACT GCAGCCTAGA CCTCCTGGGC TTAAGTGATC CTCCCACCTC AGCCTCCCCA GACTACAGGT GCACACCACC ACGCCCGGCT
017401 AATTTTTGTA TTTTTTGTAG AGATGGAGTT TCGCCATGTT GCCCAGGATG GCCTTGAACT CCAGGGCTCA AGCAATCCAC CCACCTCAAC CTCCCAAAGT
017501 GCTGGGATTA CAGGCATGAG CCATTGTGCC CAGCTGCAAA TGAATTTTTA AAAATGTGTT AGATCAATAT TCATATCACC CAAAAACAGC CTGAGAGCCC
017601 GCCACCACTG GGGACTCAGC CTGAGCCTTG TTCCCAGAAA GCCCTCAACG CAGTGCTGGG CACAGACCTG GGAGGCTTGG CTCAAACCGC CCTGCCCTCC
017701 GTTGGCAGGG CAGAGCTCCG TCTTTTCCTC TCAACAGCTC TTCCAAAGGG CAAGGCAGCA TTTTCCTGGT GAGGGGCAAG CGAAGCTGAG GCCAGACCCA
017801 CCCCCGAGTC TTCCCTCTGC CACATCCAGG GCCTGAAGGG CGTTCTGGGG AGCCTCCACC TTCACACCGA AGGCCGCCAA GGTGGTTGGC AAGAAGCTGA
017901 AATGGGCGTT TAAGTCCGAA CCTTGTTTTC AAAGGTGCCC ATTGCCTGTT CTCAAAGCAA TGCACCTGAG AAGCAGTGGC AGGTGGCACA GCCCACCCCC
018001 AACCGCCAGG GCCACCCAAA CACCAGTTAC AACACCATCA CCAACAGAGC CTTTGCAGAA CTTTTTTTTT TTTTTTTTTT TTTGAGACGG AGTCTTGCTC
018101 TGTCGCCCAG GCTGGAGTGC AATGGTGTGA TCTTGGCTGA CTGCAACCTC CGCCTCCCAA GTTCAAATGA TTCTCCTACC TCAGCCTCCC AAGCAGCTGG
018201 GACTACAGGC ACCCACCACC ATGCCCGGCT AATTTTTCTA TTTTTAGTAG AGACAGGGTT TTGCCATGTT GCCCAGGCTG GTCTCGAACT CCTGACCTCA
018301 GGTGATCTGC CCACCTTGGC CTCCCAAAGT GCTGGGATTA CAGGCATGAG CCACCACTCC TGGCCCTCTT GTATTTTTTT GTAGAGACAG GGTTTTGCCA
018401 TGTTGCCCAG GCTGGTCTCC TGGGCTCAAG TGATCCACCC ACCTTGGTCT CCCAAAGTGC TAGGATTATG GGCCTGAGCC ACCCCACCCG GCCCAACCTT
018501 ATTATTACTG AAGTCGCCTT GTTACACACA AACATCTACA GTGCCTCCTG GCCACTCCTT CTGGACTTTT CTTTCCTAGC CCAGGTCAAA GTGGGCCTTC
018601 GGGAGGACCC TGGAGGAGAA GCCCCCTCCT CTTCACTTCC ACCCTCTCTC TTCCACCCCT GGAAGTCAGC TTTCTGACCT CAGAATCCCA GCTATCTTCT
018701 GGACTCAGGT GACAAGTCAC CTAAGTGACA GATCCAGCAT GCACTTTATG ACCATCACTC TGCTCCTCCT GCATCAGCGC CCCACCCAAC AGAGCCGACC
018801 AGGCTGGGAA CACCGAGACC GAGAGCGCGT GGCTGTGTCT ACAAAGGCCT CCTCTTTGAG GGAGACACGC GAGGCACGGG TGGGGCGATG AGACAGGATC
018901 GGCCGGTCCT TGACAGTCTC TGAAGCTGGG AGAACATGAC CCCATGCTCT CCTGTTGGAG TCACCACCTC TTTGCCAACA GCCACCAAAC CTCCACCTCC
019001 AATTAAGCTC CACCCCCATG TCCCCACTGC CCTCCGGACA TCTGTTCAGC ACCCCCCTCG ACCTGGCCAA GGCTCCACGC ACCGCCCTGG TTTCCCGCTC
019101 TGCCCAGCCC CCCGCTGAAG TCTCTAATGG GTTCTTCCGA TTCCACCTGT CCAAGGTAAA ACCCTGAGGT TGTCCCTCCT CCGCGGCACC TCCAGGTATC
019201 TGGTTGCTCA GGCTGAAATG TTTCCCAAGG CCCTGGCTCC TCCTCCTTCC AAACCCCGCA GGCAGCTGAT TAGCAAACTC TGACTCTAAC CTCAAAACAT
019301 GTCCAGAATT CAGCCACATC TCCCCACTTC CCCGCCTGGC CCTGGGCCCC GCTATTCCAT CTCAATCTCT GCCCTGGCCC CCAGCCCAGC ATCCACTCTC
019401 AGACCAGAGC AATGCTTGTG AACTCCGAAA ATCTGAGACA GGTCTCAGTT AATTTAGAAA AGATATTTGT CAAGGTTGAG GACGCGCACC CATGACACAG
019501 CCTCAGGAGG TCCTGACTGG GGGGAGAGAG ACCCTCTCAT ATTGTTTTAT ACTCAGTACC TGTTTTAAGA AAAAAACGAA GTGAAATCAA AGACAGGCAG
019601 CCCGGCGCCA GGCCCAAAAC CAGGCCTGGG CCTGCCTGGC CTAAACCTAG TCGTTAAAAA TCAGCTCATG ACTTAGAACC CGATGTTACC CATAGATTTC
019701 AGGCATTGTA TGGAAGAACA TCGTGAGACT CCCTGCTCTG TTCTGTTTCA CTCTGACTAC CAGTGCATGA AACCCCTGTC ACGTATCCCC CAGACTGCTC
019801 AATCAATCAC GACCCTTTCA CGTAAAATCT TTAGTGTTGT GAGCCCTTAA AAGGGACAGA AATTGTGCAC TTGGGGAGCT GGGATTTTAA GATGGTAACT
019901 TGCCGATGCT CCCAGCTGAA TAAAGCCCTT CCTTCTACAA
```

AHNAK

```
000001 AGAGCCTGGC CCGGCGCCAG CAGCCTGAGG AATGTGGTGG TTGGAGCCGG TGACTAATTC AAACCAGAAC TCAGGGAGGA GTGGTTGTGA CCGAGATTCC
000101 CGACGAGAGA GACTGAGGGG AAGAGAGGAA GGAGGGGCGG GCTCCTGGCA AGGCATTCGC TCCTGAGCGG AATCCTGCAA AGATGGAGAA GGAGGAGACA
000201 ACCCGGGAGC TGCTGCTGCC CAACTGGCAG GGTAGTGGCT CCCACGGGCT GACCATCGCC CAGAGGGACG ACGGCGTCTT TGTGCAGGAG GTGACGCAGA
000301 ACTCCCCTGC GGCCCGCACT GGGGTGGTCA AGGAGGGGGA CCAGATTGTG GGTGCCACCA TCTACTTTGA CAACCTGCAG TCGGGTGAGG TGACCCAGCT
000401 GCTGAACACC ATGGGGCACC ACACGGTGGG CCTGAAGCTG CACCGCAAGG GGGACCGCTC TCCCGAGCCT GGCCAGACCT GGACCCGTGA AGTCTTCAGC
000501 TCCTGCAGCT CTGAAGTGGT TCTGAGCGGG GATGATGAGG AGTACCAGCG CATCTACACC ACGAAGATCA AGCCACGGCT GAAGTCGGAA GATGGAGTGG
000601 AAGGAGACCT CGGGGAGACC CAGAGCCGTA CCATCACAGT GACCAGAAGG GTCACGGCCT ACACTGTGGA TGTGACTGGC CGGGAAGGAG CCAAGGACAT
000701 AGACATCAGT AGCCCTGAAT TCAAGATCAA GATTCCAAGA CATGAACTGA CTGAAATCTC CAATGTGGAT GTGGAGACCC AGTCTGGGAA GACCGTGATC
000801 AGACTGCCCT CGGGCTCGGG GGCAGCCTCT CCGACAGGCT CTGCTGTGGA TATCCGAGCA GGGGCCATTT CTGCTTCAGG ACCAGAGCTC CAAGGTGCTG
000901 GCCACTCGAA GCTCCAGGTC ACCATGCCTG GGATAAAGGT GGGAGGCTCA GGTGTCAATG TCAATGCAAA GGGCTTGGAC TTGGGTGGCA GAGGAGGGGT
001001 CCAAGTTCCA GCAGTGGACA TTTCATCTTC TCTTGGGGGT AGGGCAGTAG AGGTACAGGG CCCATCTCTG GAGAGTGGTG ATCATGGCAA AATTAAATTT
001101 CCCACCATGA AAGTGCCGAA ATTTGGTGTC TCAACAGGGC GTGAGGGCCA GACACCAAAG GCAGGGCTGA GGGTTTCTGC ACCTGAAGTC TCTGTGGGGC
001201 ACAAGGGCGG CAAGCCAGGC TTGACTATCC AAGCCCCTCA GCTGGAAGTC AGTGTGCCCT CTGCCAATAT TGAGGGCCTT GAGGGGAAGC TGAAGGGCCC
001301 CCAAATCACT GGGCCATCAC TTGAGGGTGA CCTAGGCCTG AAAGGTGCCA AGCCACAGGG GCACATTGGG GTGGATGCCT CTGCTCCCCA AATTGGGGGT
001401 AGCATCACTG GCCCCAGTGT GGAAGTTCAG GCCCCTGACA TTGATGTTCA GGGGCCTGGG AGCAAACTGA ATGTGCCCAA GATGAAAGTC CCCAAGTTCT
001501 CTGTATCAGG TGCAAAGGGA GAGGAAACTG GGATTGATGT GACACTGCCT ACAGGTGAAG TGACTGTTCC TGGGGTCTCT GGGGATGTCA GCCTGCCTGA
001601 GATTGCTACT GGTGGGCTGG AAGGAAAGAT GAAAGGTACT AAAGTGAAGA CTCCTGAAAT GATTATTCAG AAACCTAAAA TCTCCATGCA GGATGTGGAT
001701 CTGAGCCTTG GGTCTCCTAA ACTGAAAGGA GATATTAAGG TTTCTGCTCC TGGGGTGCAA GGTGATGTTA AAGGCCCTCA AGTGGCACTT AAAGGCTCCA
001801 GAGTGGACAT AGAGACACCA AACCTAGAGG GAACCTTGAC AGGCCCTAGG CTTGGCAGTC CTTCCGGGAA AACCGGAACC TGTAGGATCT CTATGTCAGA
001901 AGTAGACTTA AATGTGGCCG CACCTAAAGT GAAAGGGGGT GTAGATGTCA CACTCCCCAG AGTAGAAGGG AAAGTCAAAG TCCCTGAAGT TGATGTCAGA
002001 GGCCCCAAAG TGGATGTCAG TGCCCCAGAT GTCGAAGCGC ATGGCCCAGA ATGGAACCTG AAAATGCCCA AGATGAAAAT GCCCACGTTC AGCACTCCAG
002101 GAGCCAAAGG GGAAGGTCCA GATGTTCATA TGACTCTACC CAAAGGAGAT ATCAGTATTT CAGGGCCCAA GGTCAATGTG GAAGCCCCAG ATGTCAACTT
002201 GGAGGGTCTG GGGGGAAAAC TTAAAGGCCC CGATGTTAAG CTGCCTGATA TGAGTGTCAA GACACCAAAG ATCTCCATGC CTGATGTAGA TTTGCACGTG
002301 AAAGGTACAA AGGTGAAGGG AGAGTATGAT GTAACTGTAC CAAAGCTGGA AGGAGAACTC AAAGGCCCAA AAGTGGACAT TGATGCCCCA GATGTGGATG
002401 TTCATGGCCC AGACTGGCAC TTGAAGATGC CCAAGATGAA AATGCCCAAA TTCAGTGTGC CAGGGTTCAA AGCAGAGGGC CCAGAAGTGG ATGTGAACCT
002501 GCCCAAGGCT GATGTGGACA TTTCCGGGCC CAAGATAGAT GTTACTGCTC CTGATGTGAG CATTGAGGAA CCAGAAGGGA AATTGAAAGG GCCCAAGTTT
002601 AAGATGCCTG AGATGAACAT CAAAGTCCCC AAGATCTCCA TGCCTGATGT GGACTTACAT CTGAAAGGCC CTAACGTAAA GGGAGAATAT GATGTCACAA
002701 TGCCAAAGGT TGAAAGTGAG ATTAAAGTTC CTGATGTTGA ACTTAAAAGT GCCAAAATGG ACATTGATGT CCCAGATGTG GAGGTTCAAG GCCCAGACTG
002801 GCACCTGAAG ATGCCCAAGA TGAAAATGCC CAAGTTCAGC ATGCCTGGCT TCAAAGCAGA GGGCCCAGAA GTGGATGTGA ACCTGCCCAA GGCTGATGTG
002901 GACATCTCAG GACCCAAGGT GGGTGTTGAA GTTCCAGATG TGAATATTGA AGGACCTGAA GGAAAGCTGA AGGGCCCCAA GTTCAAGATG CCAGAGATGA
003001 ATATCAAGGC CCCCAAGATC TCCATGCCTG ATGTGGACTT GCATATGAAA GGTCCTAAAG TAAAGGGAGA ATATGATATG ACAGTGCCAA AGCTGGAAGG
003101 GGACCTGAAA GGCCCAAAAG TAGATGTCAG TGCCCCAGAT GTTGAAATGC AGGGTCCTGA CTGGAACTTG AAGATGCCAA AGATTAAAAT GCCCAAATTT
003201 AGCATGCCCA GCCTCAAAGG AGAGGGGCCA GAATTTGATG TGAACCTGTC CAAAGCGAAT GTGGACATTT CTGCACCAAA AGTAGATACT AATGCTCCAG
003301 ATCTGAGCCT TGAAGGACCT GAAGGGAAGT TGAAAGGCCC GAAGTTTAAG ATGCCTGAGA TGCACTTCAG AGCTCCTAAG ATGTCTTTGC CAGATGTTGA
003401 CCTGGATCTT AAAGGACCCA AAATGAAAGG AAATGTAGAT ATCTCTGCAC CAAAGATAGA GGGTGAAATG CAGGTTCCAG ATGTGGACAT CAGAGGTCCC
003501 AAGGTAGATA TTAAAGCACC AGATGTGGAA GGCCAAGGCC TGGACTGGAG CCTGAAAATA CCCAAGATGA AATGCCCAA GTTCAGCATG CCCAGCCTCA
003601 AAGGCGAGGG CCCAGAAGTG GATGTGAACT TGCCTAAGGC TGACGTTGTT GTCTCAGGAC CCAAGGTGGA CATCGAAGCC CCAGATGTGA GCCTCGAAGG
003701 TCCAGAAGGG AAGCTGAAGG GTCCCAAGTT TAAGATGCCT GAGATGCATT TCAAGACCCC CAAGATCTCC ATGCCTGATG TGGACTTACA CTTGAAAGGC
003801 CCCAAAGTCA AAGGGGATGT GGATGTGTCT GTGCCCAAGG TAGAAGGTGA AATGAAAGTG CCAGATGTTG AAATCAAAGG ACCCAAAATG GACATTGATG
003901 CCCCAGATGT GGAGGTTCAA GGCCCAGACT GGCACCTGAA GATGCCCAAG ATGAAAATGC CCAAGTTTAG CATGCCTGGC TTCAAAGGAG AGGGCCGAGA
004001 AGTGGATGTG AACCTGCCCA AGGCTGACAT TGATGTCTCA GGACCCAAGG TGGATGTTGA AGTCCCAGAT GTGAGCCTTG AGGGCCCGGA AGGAAAGCTG
004101 AAGGGCCCCA AGTTTAAGAT GCCTGAGATG CACTTCAAGG CCCCCAAGAT CTCCATGCCT GATGTGGACC TGAATCTTAA GGGGCCAAAA TTGAAGGGAG
004201 ATGTGGATGT GTCCTTGCCT GAGGTAGAAG GTGAAATGAA AGTGCCAGAT GTTGACATTA AAGGGCCCAA AGTTGACATT AGTGCTCCAG ATGTGGATGT
004301 TCATGGCCCA GATTGGCACC TGAAGATGCC CAAGGTGAAA ATGCCCAAGT TCAGCATGCC CGGCTTCAAA GGAGAGGGCC CTGAAGTGGA TGTGAAGCTG
004401 CCCAAAGCTG ACGTTGATGT CTCAGGACCC AAAATGGATG CTGAAGTTCC AGATGTGAAT ATTGAAGGTC CAGACGCAAA ACTAAAAGGT CCCAAATTCA
004501 AGATGCCAGA AATGAGTATA AAGCCTCAGA AGATATCCAT ACCAGATGTT GGTTTGCATT TGAAAGGTCC TAAAATGAAA GGAGATTATG ATGTAACAGT
004601 TCCAAAAGTA GAAGGAGAGA TAAAAGCTCC TGATGTTGAC ATCAAAGGCC CCAAAGTTGA TATTAATGCA CCAGATGTGG AGGTTCATGG CCCAGACTGG
004701 CACCTGAAGA TGCCCAAGGT AAAAATGCCC AAGTTCAGCA TGCCTGGCTT TAAAGGAGAG GGCCCAGAGG TGGATATGAA CCTGCCCAAG GCTGACCTTG
004801 GTGTTTCAGG ACCCAAGGTG GACATTGATG TTCCAGATGT GAATCTTGAA GCTCCAGAGG GGAAACTAAA AGGCCCTAAG TTCAAGATGC CAAGCATGAA
004901 TATACAGACG CACAAAATCT CTATGCCTGA TGTTGGACTT AATTTGAAAG CCCCTAAACT GAAAACTGAT GTAGATGTTT CCCTTCCCAA AGTGGAAGGA
005001 GACTTGAAGG GTCCTGAAAT TGATGTGAAA GCCCCTAAGA TGGATGTGAA TGTTGGTGAT ATTGATATTG AAGGTCCAGA AGGGAAGTTG AAGGGCCCCA
005101 AGTTTAAGAT GCCTGAGATG CATTTCAAGG CCCCCAAGAT CTCCATGCCC GATGTGGACT TACACTTGAA AGGCCCCAAA GTCAAAGGGG ATATGGATGT
005201 GTCTGTGCCC AAGGTAGAAG GTGAAATGAA AGTGCCAGAT GTTGACATTA AAGGGCCCAA AGTGGACATT GATGCCCCAG ATGTGGAGGT TCACGACCCA
005301 GATTGGCACC TGAAAATGCC CAAGATGAAA ATGCCCAAGT TCAGTATGCC TGGCTTCAAA GCAGAGGGCC CTGAAGTGGA TGTGAATCTG CCAAAGGCTG
005401 ACATTGATGT GTCTGGACCC AGTGTGGACA CTGATGCTCC TGATTTGGAT ATTGAGGGAC CAGAAGGAAA GTTGAAAGGC TCCAAATTTA AGATGCCCAA
005501 GTTGAATATA AAAGCTCCCA AGGTCTCCAT GCCAGATGTG GACTTGAATT TGAAGGGACC CAAACTGAAG GGAGAGATAG ATGCTTCTGT GCCAGAACTG
005601 GAAGGTGATC TCAGAGGGCC GCAAGTTGAT GTCAAAGGTC CTTTTGTGGA AGCGGAGGTG CCCGATGTTG ATCTGGAGTG TCCTGATGCA AAGTTGAAAG
005701 GGCCCAAGTT TAAGATGCCT GAGATGCACT TCAAGGCCCC CAAGATCTCC ATGCCTGATG TGGACTTACA CCTGAAAGGC CCCAAAGTCA AAGGGGATGC
```

005801 GGATGTGTCG GTGCCAAAAT TGGAGGGAGA TTTAACAGGC CCCAGTGTGG GTGTGGAGGT GCCTGATGTT GAGCTGGAGT GTCCTGATGC AAAGTTGAAA
005901 GGCCCTAAAT TTAAGATGCC AGACATGCAC TTCAAGGCCC CCAAGATCTC CATGCCTGAT GTGGACTTAC ACTTGAAAGG CCCCAAAGTC AAAGGGGATG
006001 TGGATGTGTC GGTGCCAAAA TTGGAGGGAG ATTTAACAGG TCCCAGTGTG GGTGTGGAGG TGCCTGATGT TGAGCTGGAG TGTCCTGATG CAAAGTTGAA
006101 AGGGCCCAAG TTTAAGATGC CTGAGATGCA CTTCAAGACC CCCAAGATCT CCATGCCTGA TGTGGACTTA CACCTGAAAG GCCCCAAAGT CAAAGGGGAT
006201 ATGGATGTGT CTGTGCCCAA GGTAGAAGGT GAAATGAAAG TGCCAGATGT TGACATCAAA GGACCCAAAA TGGACATTGA TGCCCCAGAT GTGGATGTTC
006301 ATGGCCCAGA CTGGCACCTG AAGATGCCCA AGATGAAAAT GCCCAAGTTC AGCATGCCTG GCTTCAAAGC AGAGGGCCCA GAAGTGGATG TGAACTTGCC
006401 CAAGGCTGAT GTTGTTGTCT CAGGACCCAA GGTGGATGTT GAAGTCCCAG ATGTGAGCCT TGAAGGTCCA GAAGGGAAGC TGAAGGGCCC CAAGCTTAAG
006501 ATGCCTGAGA TGCACTTCAA GGCCCCCAAG ATCTCCATGC CTGATGTGGA CTTACACTTG AAAGGCCCCA AAGTCAAAGG GGATGTGGAT GTGTCTTTGC
006601 CAAAATTGGA GGGAGATTTA ACAGGCCCCA GTGTGGATGT GGAGGTGCCT GATGTTGAGC TGGAGTGTCC TGATGCAAAG TTGAAAGGGC CCAAGTTTAA
006701 GATGCCTGAG ATGCACTTCA AGACCCCCAA GATCTCCATG CCTGATGTGA ACTTAAACTT GAAAGGCCCC AAAGTCAAAG GGGATATGGA TGTGTCTGTT
006801 CCCAAGGTAG AAGGTGAAAT GAAAGTGCCA GATGTTGACA TCAGAGGGCC CAAAGTGGAC ATTGATGCCC CAGATGTGGA TGTTCATGGC CCAGACTGGC
006901 ACCTGAAGAT GCCTAAGATG AAAATGCCCA AGTTCAGCAT GCCTGGCTTC AAAGGAGAGG GCCCAGAAGT GGATGTGAAC TTGCCCAAGG CTGACGTTGA
007001 TGTCTCAGGA CCCAAGGTGG ATGTTGAAGT CCCAGATGTG AGCCTTGAAG GTCCAGAAGG GAAGCTGAAG GGCCCCAAGT TTAAGATGCC TGAGATGCAC
007101 TTCAAGACCC CCAAGATCTC CATGCCTGAT GTTGATTTCA ATTTAAAGGG ACCCAAAATC AAAGGAGATG TTGATGTTTC TGCCCCAAAG CTGGAGGGAG
007201 AGTTAAAAGG TCCAGAATTG GATGTCAAAG GTCCCAAATT AGATGCTGAC ATGCCAGAAG TAGCTGTGGA AGGCCCAAAT GGCAAGTGGA AAACTCCTAA
007301 GTTCAAGATG CCAGATATGC ACTTTAAAGC TCCCAAAATC TCTATGCCAG ACCTCGATCT ACACTTGAAG AGCCCCAAGG CAAAAGGAGA GGTGGATGTA
007401 GATGTTCCCA AATTGGAAGG GGACCTTAAA GGGCCACATG TGGATGTCAG TGGGCCAGAC ATTGACATTG AGGGACCAGA GGGCAAATTG AAAGGCCCTA
007501 AGTTCAAGAT GCCTGATATG CATTTCAAAG CCCCCAATAT TTCTATGCCT GATGTTGATC TAAATCTCAA AGGACCCAAA ATCAAGGGGG ATGTGGATGT
007601 GTCTGTGCCT GAGGTAGAAG GTAAACTTGA AGTACCAGAT ATGAACATCA GGGGCCCCAA AGTTGATGTA AATGCCCCCG ATGTCCAAGC TCCAGACTGG
007701 CACCTGAAGA TGCCCAAGAT GAAAATGCCC AAGTTCAGCA TGCCTGGCTT CAAAGCAGAG GGCCCTGAAG TAGACGTCAA CTTGCCTAAG GCTGACGTTG
007801 ACATCTCAGG ACCCAAGGTG GACATTGAAG GCCCTGATGT TAATATTGAA GGACCAGAGG GAAAGTTGAA AGGGCCTAAG TTAAAGATGC CAGAGATGAA
007901 CATCAAAGCC CCCAAGATCT CCATGCCTGA CTTTGATTTG CATCTGAAAG GTCCCAAGGT GAAGGGCGAT GTGGATGTTT CTCTGCCCAA AGTGGAAGGT
008001 GACCTCAAGG GCCCCGAAGT TGACATCAAG GGGCCCAAAG TGGATATTAA TGCCCCAGAT GTGGGTGTTC AAGGCCCAGA CTGGCACCTG AAGATGCCCA
008101 AGGTGAAAAT GCCAAAGTTC AGCATGCCTG GCTTCAAAGG AGAGGGCCCA GATGGGGATG TGAAGCTGCC CAAGGCTGAC ATTGATGTCT CAGGACCCAA
008201 AGTGGACATT GAAGGCCCTG ATGTTAACAT TGAAGGACCA GAGGGAAAGT TGAAAGGGCC TAAGTTCAAG ATGCCAGAGA TGAATATCAA AGCCCCCAAG
008301 ATCTCCATGC CTGATATTGA CTTAAACCTG AAAGGACCCA AAGTGAAGGG TGATGTGGAT GTTTCCCTTC CTAAAGTGGA AGGTGACCTC AAGGGCCCAG
008401 AAGTTGACAT CAAGGGCCCA AAAGTGGACA TTGACGCACC TGATGTTGAT GTTCATGGCC CAGACTGGCA CCTAAAGATG CCCAAGATAA AAATGCCCAA
008501 GATCAGCATG CCTGGCTTCA AAGGAGAAGG TCCAGATGTG GACGTGAACC TGCCCAAGGC TGACATTGAT GTCTCAGGAC CGAAAGTGGA TGTTGAATGT
008601 CCCGATGTGA ATATCGAAGG ACCTGAAGGA AAGTGGAAAA GTCCAAAGTT TAAGATGCCA GAGATGCATT TTAAGACTCC AAAGATATCC ATGCCAGATA
008701 TTGACCTGAA TCTCACAGGT CCAAAAATAA AAGGAGATGT GGATGTTACA GGCCCTAAGG TAGAGGGAGA TCTGAAAGGT CCTGAAGTTG ACCTCAAAGG
008801 CCCCAAAGTG GACATTGATG TCCCAGATGT TAATGTTCAG GGTCCAGACT GGCACCTGAA GATGCCCAAG ATGAAAATGC CCAAGTTCAG CATGCCTGGC
008901 TTCAAAGCAG AGGGCCCTGA AGTGGATGTG AACCTGCCCA AGGCTGACGT TGATGTCTCA GGCCCCAAAG TGGACGTTGA AGGCCCTGAT GTTAACATTG
009001 AAGGACCAGA GGGAAAGTTG AAAGGGCCCA AGTTCAAGAT GCCAGAGATG AATATCAAAG CCCCCAAGAT CCCCATGCCT GACTTTGATT TGCATCTGAA
009101 AGGTCCCAAG GTGAAGGGCG ATGTGGATAT TTCTCTGCCC AAAGTGGAAG GTGACCTCAA GGGCCCTGAA GTTGACATCA GGGGTCCCCA AGTGGACATT
009201 GATGTCCCGG ATGTGGGCGT TCAAGGCCCA GACTGGCACC TAAAAATGCC CAAAGTGAAA ATGCCCAAAT TCAGCATGCC TGGCTTCAAA GGAGAGGGCC
009301 CAGATGTGGA TGTGAACCTG CCCAAGGCTG ACCTTGATGT CTCAGGACCC AAGGTGGACA TTGATGTTCC AGATGTGAAT ATCGAAGGCC CAGAGGGAAA
009401 GTTGAAAGGT CCCAAATTCA AAATGCCTGA GATGAACATC AAAGCCCCCA AGATCTCCAT GCCTGACATT GATCTTAACC TGAAAGGTCC CAAAGTGAAG
009501 GGTGACATGG ATGTGTCTCT GCCAAAAGTG GAAGGTGACA TGAAAGTTCC TGACGTGGAT ATTAAAGGCC CCAAAGTGGA TATTAATGCC CCAGATGTGG
009601 ATGTTCAAGG CCCAGACTGG CACCTGAAGA TGCCTAAAAT AAAAATGCCC AAGATCAGCA TGCCTGGCTT CAAAGGAGAA GGTCCAGAAG TGGACGTGAA
009701 CCTGCCCAAG GCTGACCTTG ACGTCTCAGG ACCCAAGGTG GACGTTGATG TTCCAGATGT GAATATTGAA GGTCCAGATG CGAAACTGAA GGGCCCTAAA
009801 TTCAAGATGC CAGAGATGAA CATCAAAGCT CCTAAAATAT CAATGCCTGA TTTGGACCTC AATCTTAAAG GCCCTAAAAT GAAAGGAGAG GTGGATGTTT
009901 CACTTGCAAA TGTAGAAGGT GATTTGAAAG GACCTGCTCT TGACATAAAA GGCCCAAAGA TAGATGTAGA TGCTCCAGAT ATTGACATTC ATGGCCCAGA
010001 TGCCAAATTA AAAGGTCCAA AACTGAAGAT GCCTGACATG CATGTAAACA TGCCCAAGAT CTCCATGCCA GAAATTGACT TGAATTTGAA AGGCTCAAAG
010101 CTTAAGGGAG ATGTTGATGT CTCTGGGCCC AAGTTGGAAG GTGACATTAA AGCTCCCAGT TTGGATATAA AGGGCCCAGA AGTGGACGTT TCCGGTCCTA
010201 AGCTTAATAT CGAAGGCAAG TCAAAGAAAT CTCGTTTTAA GCTTCCCAAA TTTAATTTTT CGGGCTCTAA AGTTCAGACA CCTGAAGTGG ATGTCAAAGG
010301 TAAAAAGCCA GATATTGACA TAACAGGTCC AAAAGTTGAT ATTAATGCTC CTGATGTCGA GGTCCAAGGA AAAGTGAAAG GATCCAAGTT TAAAATGCCT
010401 TTCCTGAGTA TTTCATCTCC CAAAGTTTCT ATGCCTGACG TGGAGCTAAA TTTGAAAAGT CCCAAAGTCA AAGGAGACTT AGATATTGCA GGTCCCAATT
010501 TAGAAGGTGA CTTTAAAGGC CCCAAAGTGG ATATTAAGGC ACCAGAAGTC AATCTTAATG CACCTGATGT GGATGTTCAT GGTCCAGACT GGAATCTGAA
010601 AATGCCCAAG ATGAAAATGC CCAAATTCAG TGTGTCTGGC TTAAAAGCAG AAGGGCCAGA TGTAGCTGTG GATCTACCAA AAGGAGACAT CAACATAGAG
010701 GGCCCAAGTA TGAACATTGA GGGCCCAGAT CTCAATGTGG AAGGTCCGGA GGGAGGCTTG AAAGGTCCCA AATTCAAGAT GCCTGACATG AATATCAAAG
010801 CTCCCAAGAT CTCCATGCCT GACATTGACT TAAACTTGAA AGGCCCCAAG GTGAAAGGTG ATGTGGATAT TTCTCTTCCC AAACTTGAAG GGGATCTGAA
010901 AGGGCCAGAG GTTGATATCA AAGGCCCTAA AGTGGACATC AATGCCCCAG ATGTGGATGT TCATGGTCCA GACTGGCATC TGAAGATGCC CAAAGTGAAA
011001 ATGCCCAAGT TCAGCATGCC TGGCTTCAAA GGAGAAGGCC CTGAAGTCGA TGTTACCCTC CCTAAAGCTG ACATTGACAT TTCTGGTCCC AATGTAGACG
011101 TTGATGTTCC AGACGTGAAT ATTGAAGGTC CAGATGCAAA GCTGAAGGGC CCCAAGTTCA AGATGCCTGA GATGAACATC AAAGCCCCCA AGATCTCCAT
011201 GCCTGACTTT GACCTGAACT TGAAGGGACC CAAAATGAAG GGTGATGTGG TTGTGTCTTT GCCCAAAGTG GAAGGTGATC TAAAAGGCCC TGAGGTGGAC
011301 ATCAAGGGCC CCAAAGTGGA CATTGACACT CCTGACATTA ACATCGAAGG CTCAGAGGGT AAATTCAAGG GACCCAAATT TAAGATACCA GAGATGCACC
011401 TGAAGGCTCC CAAAATATCG ATGCCTGACA TTGATTTAAA CCTGAAGGGC CCCAAAGTCA AGGGCGATGT GGATGTTTCT CTGCCCAAAA TGGAAGGTGA
011501 CCTCAAGGGT CCTGAAGTTG ACATCAAGGG CCCCAAAGTG GACATTAATG CTCCAGATGT TGATGTTCAA GGCCCAGACT GGCACCTGAA GATGCCCAAG
011601 GTGAAAATGC CCAAGTTCAG CATGCCTGGC TTCAAAGGAG AGGGCCCAGA TGTGGATGTG AACCTGCCCA AGGCTGACCT TGATGTCTCA GGACCCAAGG
011701 TGGACATTGA TGTTCCAGAT GTGAATATCG AAGGCCCAGA GGGAAAGTTG AAAGGTCCCA AATTCAAGAT GCCTGAGATG AACATCAAAG CCCCCAAGAT
011801 CTCCATGCCT GACATTGATC TTAACCTGAA AGGACCCAAA GTGAAGGGTG ATATGGATGT GTCTCTGCCA AAAGTGGAAG GTGACATGCA AGTTCCTGAC
011901 TTGGATATTA AAGGCCCCAA AGTGGATATT AATGCCCCAG ATGTGGATGT TCGAGGCCCA GACTGGCACC TGAAGATGCC TAAGATAAAA ATGCCCAAGA
012001 TCAGCATGCC TGGCTTCAAA GGAGAAGGTC CAGAAGTGGA TGTGAACCTG CCCAAGGCTG ACCTTGACGT CTCAGGACCC AAGGTGGACG TTGATGTTCC
012101 AGATGTGAAT ATTGAAGGTC CAGATGCGAA ACTGAAGGGC CCTAAATTCA AGATGCCAGA GATGAACATC AAAGCCCCCA AGATCTCCAT GCCTGACTTT
012201 GATTTGCATC TGAAAGGCCC TAAGGTGAAA GGAGATGTGG ATGTTTCTCT GCCTAAGATG GAAGGTGATC TAAAGGCCCC TGAAGTTGAC ATCAAGGGCC
012301 CCAAAGTGGA CATTGATGCC CCAGATGTGG ATGTTCATGG CCCAGACTGG CACCTGAAGA TGCCCAAGGT GAAAATGCCC AAATTCAGCA TGCCAGGATT
012401 TAAAGGAGAG GGCCCAGAAG TGGATGTTAA TTTGCCCAAA GCTGACATTG ATGTCTCAGG ACCCAAAGTG GACATTGACA CTCCTGATAT TGATATTCAT
012501 GGTCCAGAAG GGAAACTGAA GGGCCCCAAA TTTAAAATGC CTGACCTGCA CCTCAAGGCA CCGAAGATCT CTATGCCTGA AGTTGACCTG AATCTGAAAG
012601 GTCCAAAGAT GAAGGGCGAC GTGGACGTTT CTCTGCCCAA AGTGGAAGGC GACCTCAAGG GCCCTGAAGT TGACATCAAG GGCCCCAAAG TGGACATTGA
012701 TGTCCCAGAT GTGGACGTTC AAGGCCCAGA CTGGCACTTA AAAATGCCCA AAGTGAAAAT GCCCAAGTTC AGCATGCCTG GCTTCAAAGG AGAGGGCCCA
012801 GATGTGGATG TGAACCTGCC CAAGGCTGAC CTTGACGTCT CAGGACCCAA GGTGGACATT GATGTTCCTG ATGTGAATAT CGAAGGTCCA GATGCGAAAC
012901 TAAAGGGCCC TAAATTCAAG ATGCCTGAGA TGAACATCAA AGCCCCCAAG ATCTCCATGC CTGACTTTGA TTTGCATCTG AAAGGTCCCA AGGTGAAGGG
013001 TGATGTGGAT GTTTCCCTTC CTAAAGTGGA AGGTGACCTC AAGGGCCCAG AAGTTGACAT CAAGGGCCCC AAAGTGGACA TCGATGCCCC TGATGTAGAT
013101 GTTCATGGCC CAGACTGGCA CCTGAAGATG CCCAAGGTGA AAATGCCCAA ATTCAGCATG CCAGGATTCA AAGGAGAGGG CCCAGATGTG GATGTTACCC
013201 TTCCTAAGGC TGACATTGAG ATTTCTGGCC CCAAAGTGGA CATTGATGCC CCTGATGTCA GTATCGAAGG TCCAGATGCA AAACTCAAGG GTCCAAAGTT
013301 CAAGATGCCA GAGATGAACA TCAAGGCCCC CAAAATCTCC ATGCCTGACA TTGACTTTAA CTTGAAGGGT CCCAAAGTGA AAGGTGATGT GGATGTCTCT
013401 CTGCCCAAAG TGGAAGGTGA TCTCAAGGGC CCTGAAATTG ACATAAAAGG CCCCAGTTTG GACATTGACA CACCTGATGT CAATATTGAA GGTCCGGAAG
013501 GAAAATTGAA GGGGCCCAAA TTTAAGATGC CTGAGATGAA CATCAAAGCT CCCAAAATCT CTATGCCTGA CTTTGATTTG CACCTGAAAG GTCCCAAGGT
013601 GAAGGGTGAT GTGGATGTTT CACTACCTAA GGTGGAAAGT GATCTGAAAG GGCCAGAGGT AGACATTGAA GGTCCTGAAG GGAAGCTCAA AGGTCCCAAG
013701 TTTAAGATGC CTGATGTACA TTTCAAAAGC CCACAAATCT CCATGAGTGA CATTGATTTG AATTTGAAAG GACCTAAGAT AAAAGGAGAT ATGGACATTT
013801 CCGTTCCTAA ACTGGAGGGA GATCTGAAAG GTCCCAAAGT GGATGTCAAA GGCCCTAAAG TGGGCATTGA CACTCCTGAT ATTGACATTC ATGGTCCAGA
013901 AGGGAAACTG AAGGGCCCCA AATTTAAAAT GCCTGACTTA CACCTCAAGG CACCGAAGAT CTCTATGCCT GAAGTTGACC TGAATCTGAA AGGTCCAAAG
014001 GTGAAGGGCG ACATGGACAT TTCTCTGCCC AAAGTGGAAG GCGACCTCAA GGGCCCCGAA GTTGACATCA GGGACCCCAA AGTGGACATT GATGTCCCAG
014101 ATGTGGACGT TCAAGGCCCA GACTGGCACC TAAAAATGCC CAAAGTGAAA ATGCCCAAGT TCAGCATGCC TGGCTTCAAA GGAGAGGGCC CAGATGTGGA
014201 TGTGAACCTG CCCAAGGCTG ACATTGATGT CTCAGGACCC AAAGTGGACG TTGATGTTCC TGATGTGAAT ATCGAAGGTC CAGATGCGAA ACTAAAGGGC
014301 CCCAAGTTCA AGATGCCTGA GATGAGCATC AAAGCCCCCA AGATCTCCAT GCCTGATATT GACTTAAACC TGAAAGGACC CAAAGTGAAG GGCGATGTGG
014401 ATGTTACCCT TCCTAAAGTG GAAGGTGACC TCAAGGGCCC AGAAGCTGAC ATCAAGGGCC CAAAAGTGGA CATCAACACC CCTGATGTGG ATGTTCATGG
014501 CCCAGACTGG CACCTGAAGA TGCCCAAGGT GAAAATGCCC AAATTCAGCA TGCCTGGCTT CAAAGGAGAA GGTCCAGATG TGGATGTGAG CCTGCCCAAG

014601 GCCGACATCG ATGTCTCGGG ACCCAAGGTG GACGTTGATA TTCCAGATGT GAATATCGAA GGTCCAGACG CAAAACTGAA GGGCCCCAAG TTCAAGATGC
014701 CTGAAATAAA TATCAAAGCT CCCAAGATCT CCATACCTGA TGTTGACCTG GATTTGAAAG GACCCAAAGT AAAAGGAGAT TTTGATGTGT CTGTCCCTAA
014801 GGTTGAAGGG ACTTTGAAAG GCCCAGAAGT AGATCTTAAA GGTCCACGTC TGGATTTCGA AGGCCCTGAT GCCAAACTCA GTGGCCCATC TTTGAAGATG
014901 CCATCGCTGG AGATATCTGC TCCTAAAGTA ACTGCTCCTG ATGTTGATTT GCATCTCAAG GCACCAAAAA TTGGATTTTC AGGTCCGAAG TTAGAAGGTG
015001 GTGAAGTGGA CCTCAAGGGA CCCAAAGTTG AAGCTCCAAG CTTAGATGTA CACATGGACA GCCCAGATAT TAACATCGAA GGGCCAGATG TTAAAATCCC
015101 CAAATTTAAG AAACCCAAGT TTGGATTTGG GGCAAAAAGC CCCAAAGCTG ACATCAAGTC ACCTTCACTG GATGTCACTG TTCCTGAGGC AGAGCTGAAC
015201 CTTGAGACTC CTGAAATTAG TGTTGGTGGC AAGGGCAAGA AAAGTAAGTT TAAAATGCCT AAAATTCATA TGAGTGGTCC TAAGATTAAG GCCAAAAAAC
015301 AGGGATTTGA CCTGAATGTT CCTGGGGGTG AAATTGATGC CAGCCTCAAG GCTCCGGATG TAGATGTCAA CATCGCAGGG CCGGATGCTG CACTCAAAGT
015401 CGACGTGAAA TCGCCCAAAA CCAAGAAAAC GATGTTTGGA AAAATGTACT TCCCAGATGT AGAGTTTGAC ATTAAATCAC CTAAATTTAA AGCTGAGGCC
015501 CCTCTCCCTA GCCCCAAACT GGAGGGTGAA CTCCAGGCAC CTGATCTGGA ACTTTCTTTG CCAGCGATTC ACGTCGAAGG TCTTGACATC AAGGCGAAGG
015601 CTCCCAAGGT CAAGATGCCA GATGTGGACA TCTCAGTGCC AAAAATAGAG GGTGACCTGA AAGGCCCCAA AGTGCAGGCA AACTTGGGTG CACCTGACAT
015701 CAACATCGAA GGCCTAGATG CTAAAGTCAA AACACCGTCC TTCGGCATTT CTGCCCCTCA AGTCTCCATC CCTGATGTGA ATGTAAACTT GAAAGGACCA
015801 AAGATAAAGG GTGATGTCCC CAGCGTGGGA CTGGAAGGAC CAGATGTAGA TCTGCAAGGT CCAGAAGCAA AAATTAAGTT CCCCAAGTTT TCCATGCCCA
015901 AGATCGGCAT CCCAGGTGTG AAAATGGAGG GTGGGGGAGC CGAGGTCCAT GCCCAGCTAC CCTCTCTTGA AGGAGACTTG AGAGGACCAG ATGTTAAGCT
016001 CGAAGGGCCC GATGTTTCTC TAAAGGGGCC AGGAGTAGAC TTGCCTTCAG TGAACCTCTC TATGCCAAAA GTCTCTGGGC CTGACCTTGA TCTGAACTTG
016101 AAAGGACCAA GTTTGAAGGG AGACCTGGAT GCATCTGTTC CCAGCATGAA GGTGCATGCT CCAGGGCTCA ACCTCAGTGG TGTCGGTGGC AAAATGCAGG
016201 TGGGAGGAGA CGGTGTGAAA GTGCCAGGGA TCGATGCCAC AACAAAGCTT AACGTTGGGG CACCAGATGT GACACTGAGG GGACCAAGCC TGCAGGGAGA
016301 TCTGGCTGTC TCTGGTGACA TCAAATGCCC TAAAGTATCC GTAGGAGCTC CTGATCTAAG CTTGGAGGCA TCCGAAGGCA GCATTAAACT TCCCAAAATG
016401 AAGCTGCCCC AATTTGGCAT CTCTACTCCG GGGTCCGACT TGCACGTCAA TGCCAAGGGG CCACAGGTTT CTGGCGAACT GAAGGGGCCA GGTGTGGATG
016501 TGAACCTGAA AGGGCCTCGG ATTTCAGCAC CGAATGTGGA CTTTAACTTG GAAGGACCAA AAGTGAAAGG GAGCCTTGGG GCCACTGGTG AGATCAAAGG
016601 CCCCACTGTC GGAGGAGGTC TTCCAGGCAT TGGTGTTCAA GGCCTAGAAG GAAACCTCCA GATGCCTGGA ATTAAGTCCT CTGGATGTGA TGTGAACCTG
016701 CCAGGCGTGA ATGTGAAACT CCCAACTGGG CAGATTTCTG GGCCTGAAAT CAAAGGTGGT CTGAAAGGTT CAGAAGTAGG TTTCCATGGG GCTGCTCCTG
016801 ATATCAGTGT GAAGGGGCCT GCCTTTAATA TGGCATCTCC TGAGTCAGAT TTTGGCATCA ACTTGAAGGG CCCAAAAATC AAAGGAGGTG CGGATGTTTC
016901 AGGGGGTGTC AGTGCCCCAG ACATCAGCCT TGGTGAAGGG CATTTGAGTG TTAAAGGTTC CGGGGGTGAG TGGAAGGGAC CCCAAGTCTC CTCTGCTCTC
017001 AACTTGGACA CATCTAAGTT TGCTGGGGGC CTTCATTTCT CAGGACCAAA GGTGGAAGGA GGTGTGAAAG GAGGTCAGAT TGGACTCCAG GCTCCTGGGC
017101 TGAGTGTGTC TGGGCCTCAA GGTCACTTGG AAAGTGGATC TGGAAAAGTA ACATTCCCTA AAATGAAGAT CCCCAAATTT ACCTTCTCTG GCCGTGAGCT
017201 GGTTGGCAGA GAAATGGGGG TGGATGTTCA CTTCCCTAAA GCAGAGGCCA GCATCCAAGC TGGTGCTGGA GACGGCGAGT GGGAAGAGTC TGAAGTCAAA
017301 CTGAAAAAGT CCAAGATCAA AATGCCCAAG TTTAATTTTT CCAAACCTAA AGGGAAAGGT GGTGTCACTG GCTCACCAGA AGCATCAATT TCTGGGTCCA
017401 AAGGTGACCT GAAAAGTTCA AAGGCCAGCC TGGGCTCTCT GGAAGGAGAG GCAGAGGCCG AAGCCTCTTC ACCGAAAGGC AAATTCTCCT TATTTAAAAG
017501 TAAGAAGCCA CGGCACCGCT CAAATTCATT CAGTGATGAA AGAGAGTTCT CTGGACCTTC CACCCCGACG GGGACGCTGG AGTTTGAAGG TGGGGAAGTG
017601 TCTCTGGAAG GTGGGAAAGT TAAAGGGAAA CACGGGAAGC TGAAATTCGG TACCTTTGGT GGATTGGGGT CAAAGAGCAA AGGTCATTAT GAGGTGACTG
017701 GGAGCGATGA TGAGACAGGC AAGTTACAGG GGAGTGGGGT GTCCCTGGCC TCTAAGAAGT CCCGACTGTC CTCCTCTTCT AGCAATGACA GTGGGAATAA
017801 GGTTGGCATC CAGCTTCCCG AGGTGGAGCT GTCAGTTTCC ACAAAGAAAG AGTAGCAGGC CTTTGTATGT GTGTACATAT ATATATATAT AACAAAACAT
017901 CAGCCTTGGG TGGTGTGTTC CTATATAAAC TCCAAAGGGA AACACACCGA CTGCCTCAGC AATCATGCAA AGACCTTGCC TGGCCCGGTG GCAAGCGCTG
018001 AAAAACCGAC CGCCTGTAGG CTCCTGGAAC TATACAGATA GGTAAAGAGT TCCAAGTTCG TCCAGCCCAT GTGCAAAGTC AACAGTATTT GCCTTAAGAT
018101 TTCATATATA TATATTTTTT TGCATTGACT GCTGAGAGCT CCTGTTTACT AAGCAAGCTT TTGTGTTTAT TATCCTCATT TTTACTGAAC ATTGTTAGTT
018201 TTGGGGTAAT GGAAACCCAC TTTTTCATTG TAATGACTTT GGGGGCTTTT GTTAGTAAGG GTGGGTGGGG TGATGGGTTG CAGACGGAGG TCAGGTCTTC
018301 CTCTTTCCTG AGACTGGATC TGTTCAAACA GCAAACGCCC ACAGATGGCC CAGAGGTGGT GGTAGTCAGG GTGTGTGGGT GTTTTTAGGG TTCTTTAGTG
018401 TTGTTTCTTT CACCCAGGGG TGGTGGTCCC AGCCAGTTTG GTGCTGACGG TGAGAGGAAA TTAGAATCTG TTTGCAAATT GTCCAACCCA CCCCCTCAAC
018501 ATGAGGGGCT TCCATTTTCT GTGTTTTGTA AGGGAACTGT TTCCTTCATG CCGCCATGTT CCTGATATTA GTTCTGATTT CTTTTTAACA AATGTTATCA
018601 TGATTAAGAA AATTTCCAGC ACTTTAATGG CCAATTAACT GAGAATGTAA GAAAATTGAT GCTGTACAAG GCAAATAAAG CTGTTTATTA ACCTTGA

Supplementary Figure Legends

SF 1:

A. Western Blot analyses show protein levels for PRMT5 and PRMT1 in multiple head and neck cancer lines and A549 compared to normal oral keratinocytes and Dysplastic oral keratinocyte cells (DOK). β-actin serves as a loading control.
B. Western Blot analyses show that TGFβ promotes the expression of FXR1 and PRMT5 proteins. Histone H3 serves as a loading control.
C. HEK293 cells expressing empty vector and HA-tag PRMT5 were used for immunoprecipitation with HA-tag antibody and probed for symmetric (SDMA), FXR1, and PRMT5 antibodies. The empty vector does not show any HA-tag expression.

SF 2:

A. The western blot shows two independent guide RNA-mediated knock out (KO) of PRMT1 and PRMT5, showing a reduction in FXR1 protein in A549 cancer cells. No change in FXR2 protein is observed. GAPDH serves as a loading control. N=3.
B. The panel depicts the colony-forming efficiency from clonogenic assays of A549 cells treated with GSK-593 and GSK-712, both drugs and DMSO, for 72 hours.
C. MTT analysis of cell viability in A549 cells treated with GSK-593, GSK-712, both drugs and DMSO for 72 hours. Data presented as the mean ± SD of three experiments.
D. A549 cells were treated with PRMT5i (2µM) and tested for the expression of FXR1, FXR2, and loading control β-Actin by western blot analysis.
E. A549 cells were treated with GSK-593, GSK-712, and both for 72 hours. Protein extraction followed by immunoblotting showed no change in PRMT5, whereas a decrease in FXR1 and increased P21 protein were observed. The marker for PRMT5's function, H4R3Me2S, was found to be reduced upon GSK593 use. β-actin serves as a loading control.
F. Confocal analysis of immunofluorescence assay. 74B cells with and without PRMT5i treatment were probed with rabbit polyclonal-FXR1 antibody followed by Alexa flour 488 goat anti-rabbit antibody and counterstained with DAPI. No change in FXR1 protein localization is observed.

SF 3:

A. Our modeling analysis showed that Node1 formed a complex with G4-RNA using R386 when threaded from C to N terminus direction.
B. SDS-PAGE showing rec. FXR1 (S382-P476) purification from the glutathione S-transferase (GST) column. The bottom of the immunoblot indicates the presence of GST-tag in the proteins. The sequence depicts the R residues that were mutated to K.

SF 4:

A. Immunoblot confirmation of PRMT5 inhibition by shRNA in UMSCC74B cells. Actin serves as a loading control.
B. RNA seq analysis. PCA plot showing the pattern of DEGs in the duplicate samples of FXR1 and PRMT5 KD samples respectively.
C. Heat map of differentially regulated genes in PRMT5 KD compared to the control.
D. Dot plot shows the GO enrichment of differentially up-regulated genes.
E. Dot plot shows the GO enrichment of differentially down-regulated genes.

F. GO enrichment of significantly enriched FXR1 eCLIP mRNA targets. The bar plot shows the involvement in different biological processes for commonly up-regulated genes in PRMT5 and FXR1 KD conditions.
G. GO enrichment of significantly enriched FXR1 eCLIP mRNA targets. The bar plot shows the involvement in different biological processes for commonly down-regulated genes in PRMT5 and FXR1 KD conditions.
H. Oncoprint analysis in TCGA head and neck dataset, showing genetic alterations for the top eighteen eCLIP targets of FXR1.

SF 5:

A. Kaplan-Meier plots show that overall survival is significantly low in HNSCC patients with high PRMT5 (left) and FXR1 (right) mRNA expression. The number of patients affected is indicated in the graph. The log-rank P values are shown.
B. Kaplan-Meier plots show the overall survival is moderately low in Lung cancer patients with PRMT5 (left) expression but very low in FXR1 (right) expression. The number of patients affected is indicated in the graph. The log-rank P values are shown.
