# Supplementary material for: PRMT5-mediated arginine methylation of FXR1 is essential for RNA binding in cancer cells
Source: Nucleic Acids Res. 2024 May 6;52(12):7225–44. doi: 10.1093/nar/gkae319 (PMC11229354; doi:10.1093/nar/gkae319)
Supplement: gkae319_Supplemental_Files [file gkae319_supplemental_files.zip › Supplementary Data Legends-new.pdf]

Supplementary Data Legends

Suppl. Data 1

CLIPper, a peak calling tool, is used to locate clusters. Next, a paired input sample is used to normalize the clusters found in the FXR1 IP samples. A cluster with log2 fold enrichment > 3 and p-value ≤ 0.001 is considered a peak. The details for each peak are displayed in the spreadsheet. The fold enrichment in eCLIP relative to the paired input, as ascertained by either Fisher's Exact Test or Yates' Chi-square Test, is denoted by the log2 fold change.

Suppl. Data 2

The data contains information on FXR1's binding motif sequences as identified by the enriched peaks on its target mRNAs in UMSCC74B cells.

Suppl. Data 3

Analyses of G4 sequences (highlighted in yellow) of FXR1's top eCLIP mRNA targets by G4 mapper tool.

Suppl. Data 4

To identify the existence of G4 sequences, the G4 mapper was used to analyze the highly significant eCLIP-enriched peaks of FXR1's mRNA targets. The G4 sequence details for the top hits are included in the datasheet.

Suppl. Data 5

The pathways that are enriched in the FXR1-eCLIP peaks are displayed by the GO enrichment analysis.
