# Supplementary material for: PRMT5-mediated arginine methylation of FXR1 is essential for RNA binding in cancer cells
Source: Nucleic Acids Res. 2024 May 6;52(12):7225–44. doi: 10.1093/nar/gkae319 (PMC11229354; doi:10.1093/nar/gkae319)
Supplement: gkae319_Supplemental_Files [file gkae319_supplemental_files.zip › Table S1.docx]

**Table S1.** Primers used in the study.

| **Primer** | **Sequence (5’-3’)** |
| --- | --- |
| FXR1-F | CCCTAATTACACCTCCGGTTATG |
| FXR1-R | TCTCCTGCCAATGACCAATC |
| P21-F | CGGAACAAGGAGTCAGACATT |
| P21-R | AGTGCCAGGAAAGACAACTAC |
| GAPDH-F | GGTGGTCTCCTCTGACTTCAACA |
| GAPDH-R | GTTGCTGTAGCCAAATTCGTTGT |
| Beta Actin-F | GGACCTGACTGACTACCTCAT |
| Beta Actin-R | CGTAGCACAGCTTCTCCTTAAT |
| RPS18-F | CTTTGCCATCACTGCCATTAAG |
| RPS18-R | ATCACACGTTCCACCTCATC |
| MycFXR1-R386K-F | CTAGGTCTTATAGCGGAAAAGGCAGAGGTCGTCGGGGA |
| MycFXR1-R386K-R | TCCCCGACGACCTCTGCCTTTTCCGCTATAAGACCTAG |
| MycFXR1-R388K-F | CTTATAGCGGAAGAGGCAAAGGTCGTCGGGGACCTAA |
| MycFXR1-R388K-R | TTAGGTCCCCGACGACCTTTGCCTCTTCCGCTATAAG |
| MycFXR1-R386/388K-F | GGTTCTAGGTCTTATAGCGGAAAAGGCAAAGGTCGTCGGGGACCTAATTAC |
| MycFXR1-R386/388K-R | GTAATTAGGTCCCCGACGACCTTTGCCTTTTCCGCTATAAGACCTAGAACC |
| MycFXR1-R453K-F | GAAGTGTTTCAGGGGGTAAAGGTCGTGGTGGACCACGT |
| MycFXR1-R453K-R | ACGTGGTCCACCACGACCTTTACCCCCTGAAACACTTC |
| MycFXR1-R455K-F | AGTGTTTCAGGGGGTCGAGGTAAAGGTGGACCACGTGGTGGCAAA |
| MycFXR1-R455K-R | TTTGCCACCACGTGGTCCACCTTTACCTCGACCCCCTGAAACACT |
| MycFXR1-R459K-F | GGTCGAGGTCGTGGTGGACCAAAAGGTGGCAAATCCTCCATCAGT |
| MycFXR1-R459K-R | ACTGATGGAGGATTTGCCACCTTTTGGTCCACCACGACCTCGACC |
| MycFXR1-R453/455/459K-F | CAGAAGTGTTTCAGGGGGTAAAGGTAAAGGTGGACCAAAAGGTGGCAAATCCTCCATCAG |
| MycFXR1-R453/455/459K-R | CTGATGGAGGATTTGCCACCTTTTGGTCCACCTTTACCTTTACCCCCTGAAACACTTCTG |
| pET28a-FXR1t-F | TCG CGG ATC CGA ATT CAT GTC TTA TAG CGG AAG AGG CAG AG |
| pET28a-FXR1t-R | GTG CGG CCG CAA GCT TTG ATT ATG GAT TGC TGT CTG GAT CTT TGA G |
| pET28aFXR1t-R386K-F | TCA TGT CTT ATA GCG GAA AAG GCA GAG GTC GTC GGG G |
| pET28aFXR1t-R386K-R | CCC CGA CGA CCT CTG CCT TTT CCG CTA TAA GAC ATG A |
| pGEXFXR1t-R386-388K-F | GAA TTC ATG TCT TAT AGC GGA AAA GGC AAA GGT CGT CGG GGA CCT AAT |
| pGEXFXR1t-R386-388K-R | ATT AGG TCC CCG ACG ACC TTT GCC TTT TCC GCT ATA AGA CAT GAA TTC |
| MAP1B- F | CCCTCAGGCATCCACATATTC |
| MAP1B- R | CAAGAGGACACGAGGCATAAA |
| KMT2A- F | CTCCTCTCTTCCCTTGGTTTAC |
| KMT2A- R | CTCTTGTCAGCATCTCGATCTT |
| ZNF106- F | CACGAGAACGAAGGAACAGTAG |
| ZNF106-R | GATGGAGACACATGGGAAGATG |
| MYO5A-F | GAGAGCTGTGGCTGGTTTAT |
| MYO5A-R | CTCATTCTCATGGCTCCTCTTC |
| RNF213-F | GCTACGCATCCCTGCTATTT |
| RNF213-R | AGGTACTGGTCCTTATCCAGAG |
| PRMT5-F | TATGTGGTACGGCTGCACA3 |
| PRMT5-R | TGGCTGAAGGTGAAACAGG |
| SMG1-F | TATGGTCGGAAGTCGTTGGG |
| SMG1-R | TTGGTGGCTAAAGCACGACT |
| DYNC1H1-F | AGAAGACCAAGCCTGTCACG |
| DYNC1H1-R | CCTTGGCCTTTGCACACTTC |
| PRKDC-F | AGCCATTGCCAGAGTACCAC |
| PRKDC-R | GGATCACTGGAGGTCATGGG |
| AHNAK-F | GGGAGCGATGATGAGACAGG |
| AHNAK-R | AAACTGACAGCTCCACCTCG |
| HUWE1-F | GGAGAGCTAGCCGCATCTTC |
| HUWE1-R | TAACCCACTCAGGTCAGGCT |
| UBR4-F | CATCAGCTCCAGCCTCAGAC |
| UBR4-R | CAGGAGAGGTCCGAAGGGTA |
| AHNAK2-F | GTAGCTTCCTTGTGTCCGGC |
| AHNAK2-R | CCCTTCAGTCACAGAGTGGTC |
